# Cu/Pd-catalyzed arylboration of a 1-silyl-1,3-cyclohexadiene for stereocontrolled and diverse cyclohexane/ene synthesis†

**DOI:** 10.1039/d3sc02536e

**Published:** 2023-09-13

**Authors:** Phillip F. Crook, Alan R. Lear, Suman Das, M. Kevin Brown

**Affiliations:** a Department of Chemistry, Indiana University 800 E. Kirkwood Ave Bloomington IN 47405 USA brownmkb@indiana.edu

## Abstract

The synthesis and Cu/Pd-catalyzed arylboration of 1-silyl-1,3-cyclohexadiene is described. This diene is significant as it allows for synthesis of polyfunctional cyclohexane/enes. To achieve high levels of diastereoselectivity, the use of a pyridylidene Cu-complex was employed. In addition, through the use of a chiral catalyst, an enantioselective reaction was possible. Due to the presence of the silyl and boron substituents, the products can be easily diversified into a range of valuable cyclohexane/ene products.

## Abstract 2

Versatile diene allows for the synthesis of diverse cyclohexane/ene by a Cu/Pd-catalyzed arylboration.

## Graphical abstract

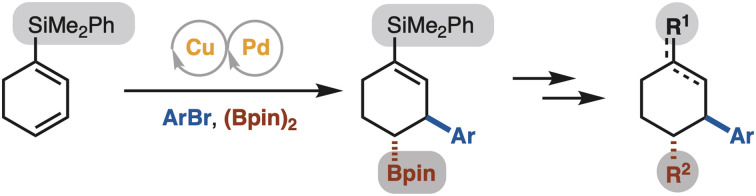

## Introduction

**Scheme 1:**
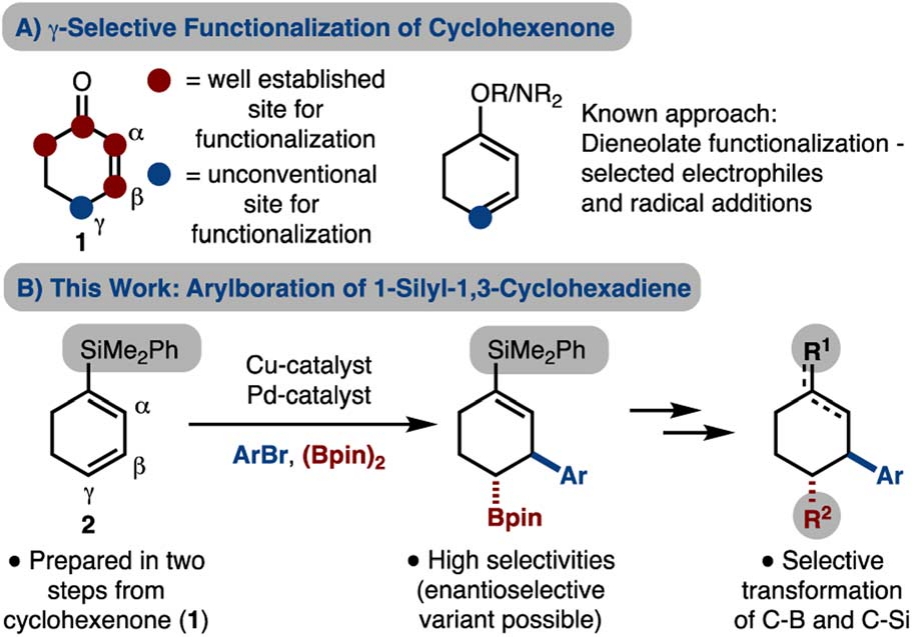
Strategies towards cyclohexane synthesis.

Multisubstituted cyclohexanes are a common motif in natural products and medicinally relevant molecules. A common starting material is cyclohexanone as conjugate addition/enolate trapping strategies have been used extensively.^1^ Cyclohexenone dieneolate is a useful intermediate which can allow for γ-functionalization.^2–4^ Based on prior reports from our lab in the development of alkene arylboration reactions,^5–8^ we became interested in using cyclohexenone dieneolate. Arylboration of this intermediate (or a surrogate) would allow for β,γ-difunctionalization, while retaining functionality at C1 and C2 for further manipulations. In addition, due to the installation of a Bpin unit, elaboration should be facile, thus allowing for diverse cyclohexane synthesis.^9^ Herein we present an arylboration of a 1-silyl-1,3-cyclohexadiene, which is easily prepared, and acts as a surrogate for cyclohexenone dieneolate. In addition, we demonstrate selective manipulation of the C–Si and C–B bonds to generate diverse cyclohexanes (Scheme 1B).

## Results and discussion

**Scheme 2:**
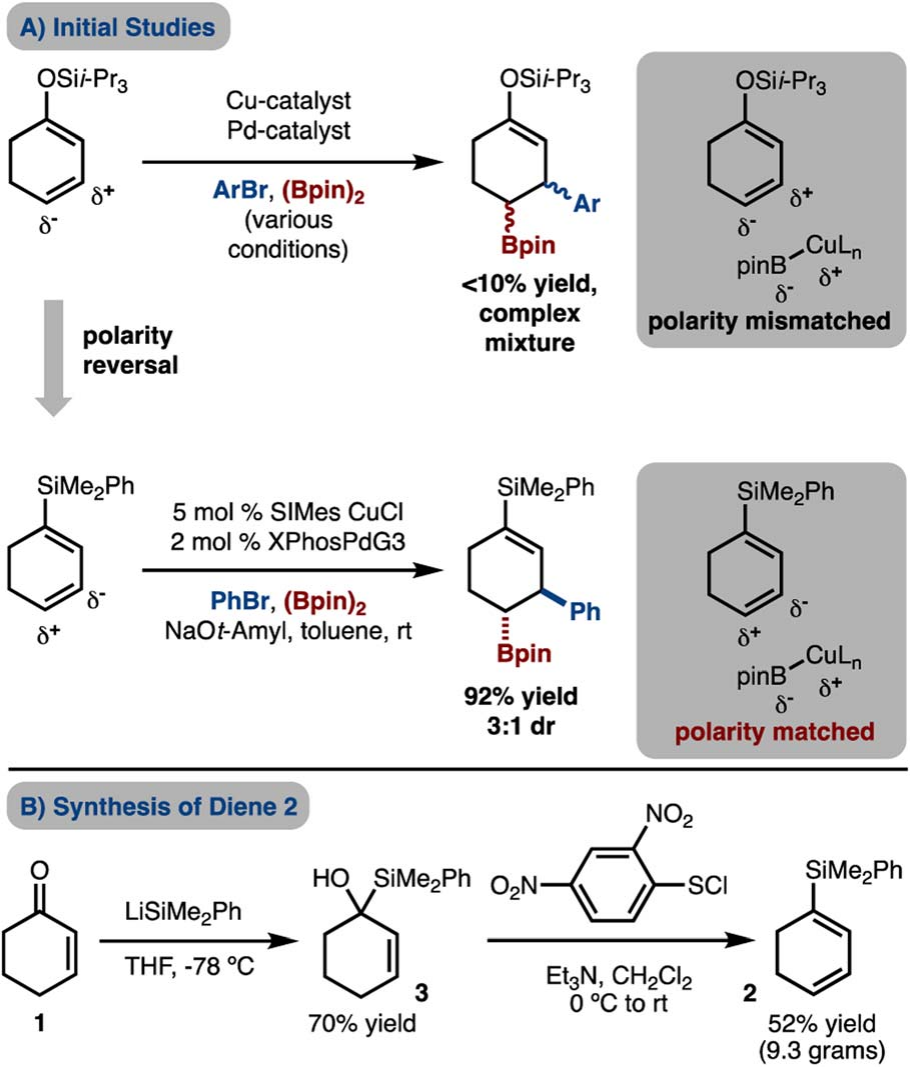
Initial studies and optimization.

Initial efforts were directed towards attempted Cu/Pd-catalyzed arylboration of a silyldieneolate (Scheme 2A). However, under various conditions a complex mixture of products was observed with the desired product being generated in <10% yield. The failure of these reactions may be due, in part, to the γ-carbon being nucleophilic by virtue of electron donation from the oxygen atom and therefore, addition of the nucleophilic Cu–Bpin is disfavored. To overcome the polarity mismatch, we designed a new substrate, 1-silyl-1,3-cyclohexanediene 2. The electropositive Si-atom removes electron density from the π-system and renders C2 and C4 electron-poor and thus allows for borylcupration to occur in a polarity matched scenario.

The requisite silyl diene 2 can easily be prepared on gram scale from 1 through a robust two-step procedure that involves (1) addition of LiSiMe_2_Ph to cyclohexenone (1),^10^ and (2) [2,3]-sigmatropic rearrangement and *syn*-elimination with 2,4-(NO_2_)_2_C_6_H_3_SCl.^11^ Under a standard set of conditions with SIMesCuCl and XPhosPdG3 ^12^ that has been used for arylboration of other alkenes,^7^ product 4a was generated in good yield but moderate diastereomeric ratio (dr), thus supporting the polarity-matched hypothesis.

**Scheme 3:**
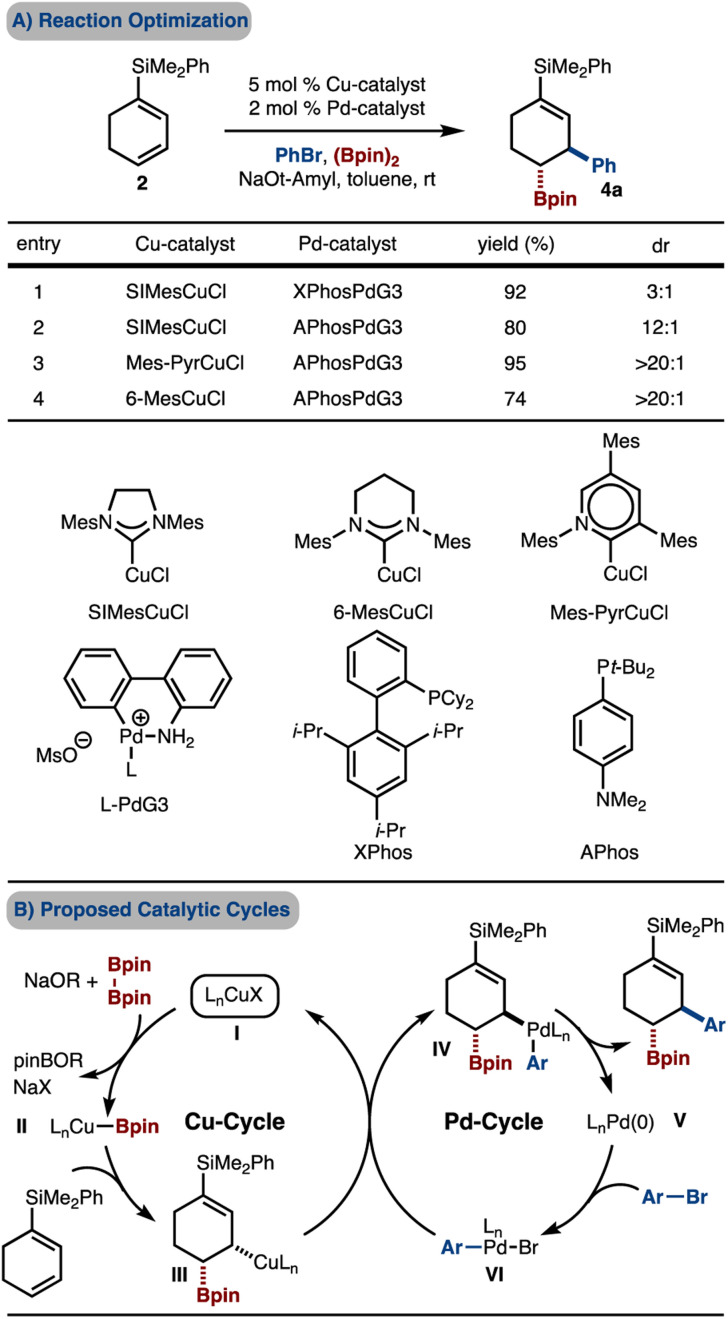
Initial results. Yield and diastereoselectivity determined by ^1^H NMR analysis with an internal standard of the unpurified reaction mixture.

Based on the initial findings, evaluation of Pd-catalysts led to the finding that APhosPdG3 (ref. 13) delivered the product with improved dr (Scheme 3A, compare entries 1–2). Further reaction optimization through examination of various Cu-catalysts revealed that reaction promoted by Mes-PyridylideneCuCl afforded the product in high yield and diastereoselectivity (Scheme 3A, entry 3).^14^ This class of catalyst was recently reported by our lab, and has been shown to, at times, offer superior reactivity compared to SIMesCuCl.^7*g*^ We hypothesized that the increased diastereoselectivity may be due to the more sterically demanding Mes-Pyridylidene as compared to SIMesCuCl (due to the positioning of the Mes groups closer to the Cu-atom as a result of the six- *vs.* -five-membered ring). This hypothesis was tested by use of the related 6-MesCuCl catalyst, which also led to high diastereoselectivity, albeit with reduced yield (Scheme 3A, entry 4). Finally, based on our prior studies, these reactions likely operate by the catalytic cycles illustrated in Scheme 3B. Key aspects of the catalytic cycles are: (1) *syn*-borylcupration of an alkene with L_*n*_Cu–Bpin (II–III), (2) transmetallation with an L_*n*_PdArBr complex that can either proceed with inversion (shown) or retention (not shown) of configuration (III–IV), (3) reductive elimination of an alkyl–Pd-complex (IV–V).

**Scheme 4:**
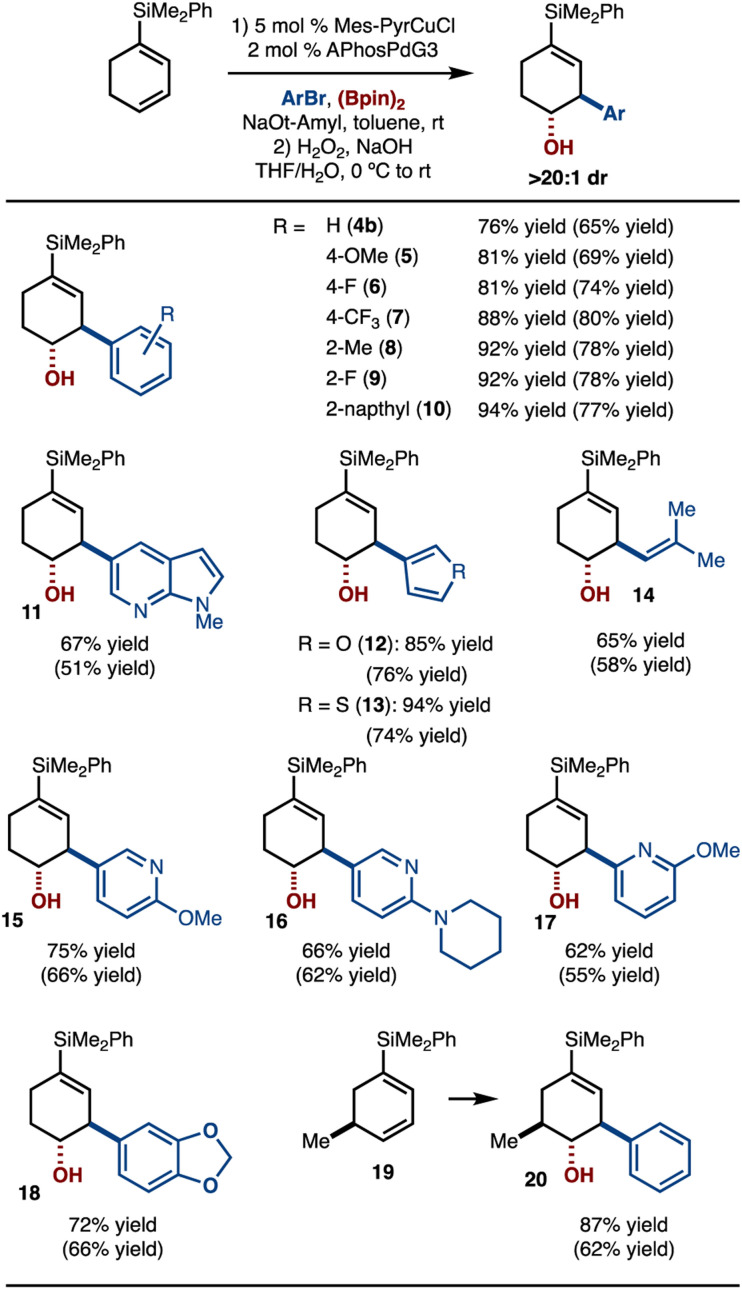
Substrate scope. See the ESI† for details. Yield and diastereoselectivity determined by ^1^H NMR analysis with an internal standard of the unpurified reaction mixture after oxidation. Yield in parentheses is of isolated purified product after oxidation.

Under the conditions outlined in Scheme 3A, entry 3, the scope of the reaction was evaluated (Scheme 4). It was found that arylbromides that bear electron-donating (products 5, 11, 18) and electron-withdrawing substituents (products 6–7, 9) functioned well. In addition, use of sterically demanding 2-MeC_6_H_4_Br did not impede the reaction (product 8). Various heterocycles (products 11–13, 15–17) as well as an alkenyl bromide were also tolerated (product 14). In addition, alkene 19 could be used and allowed for control of stereochemistry with respect to the existing stereogenic center. It should be noted that the products were oxidized prior to isolation to facilitate purification.

**Scheme 5:**
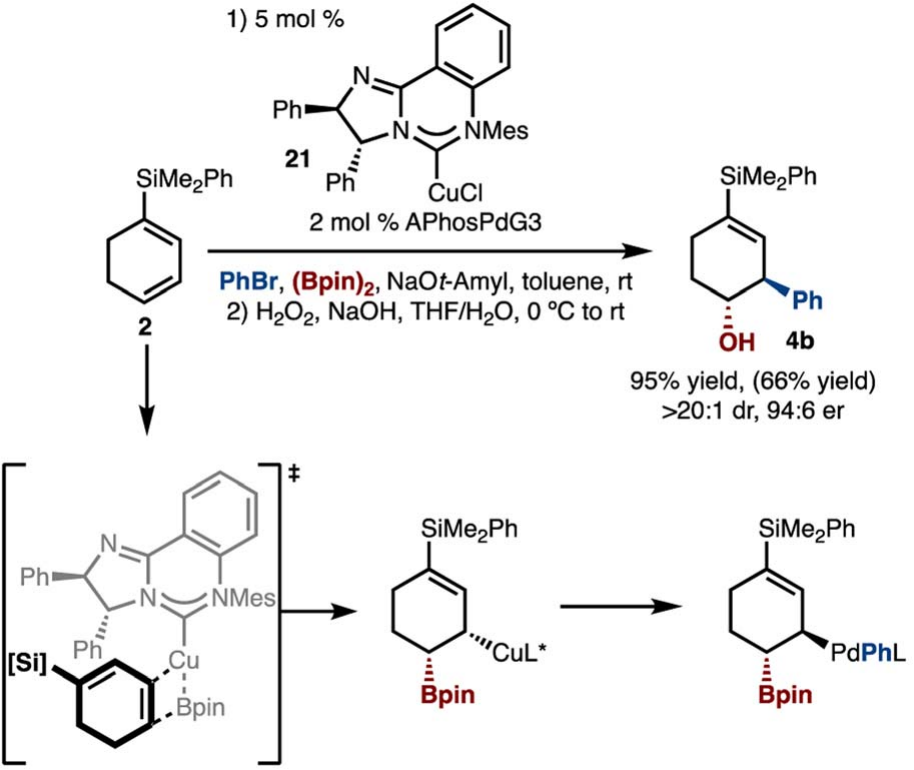
Enantioselective arylboration. Yield and diastereoselectivity determined by ^1^H NMR analysis with an internal standard of the unpurified reaction mixture after oxidation. Yield in parentheses is of isolated purified product after oxidation. Enantiomeric ratio (er) determined by HPLC analysis with a chiral column.

Enantioselective variants of the reaction were also investigated (Scheme 5). It was found that use of McQuadeCuCl (21)^15^ allowed for formation of 4b in high yield, enantioselectivity, and diastereoselectivity.^8*a*^ It is suggested that the borylcupration occurs *via* the model illustrated in Scheme 5, such that the bulk of the diene is positioned distal to the N-Mes group and opposite the proximal Ph-group.

**Scheme 6:**
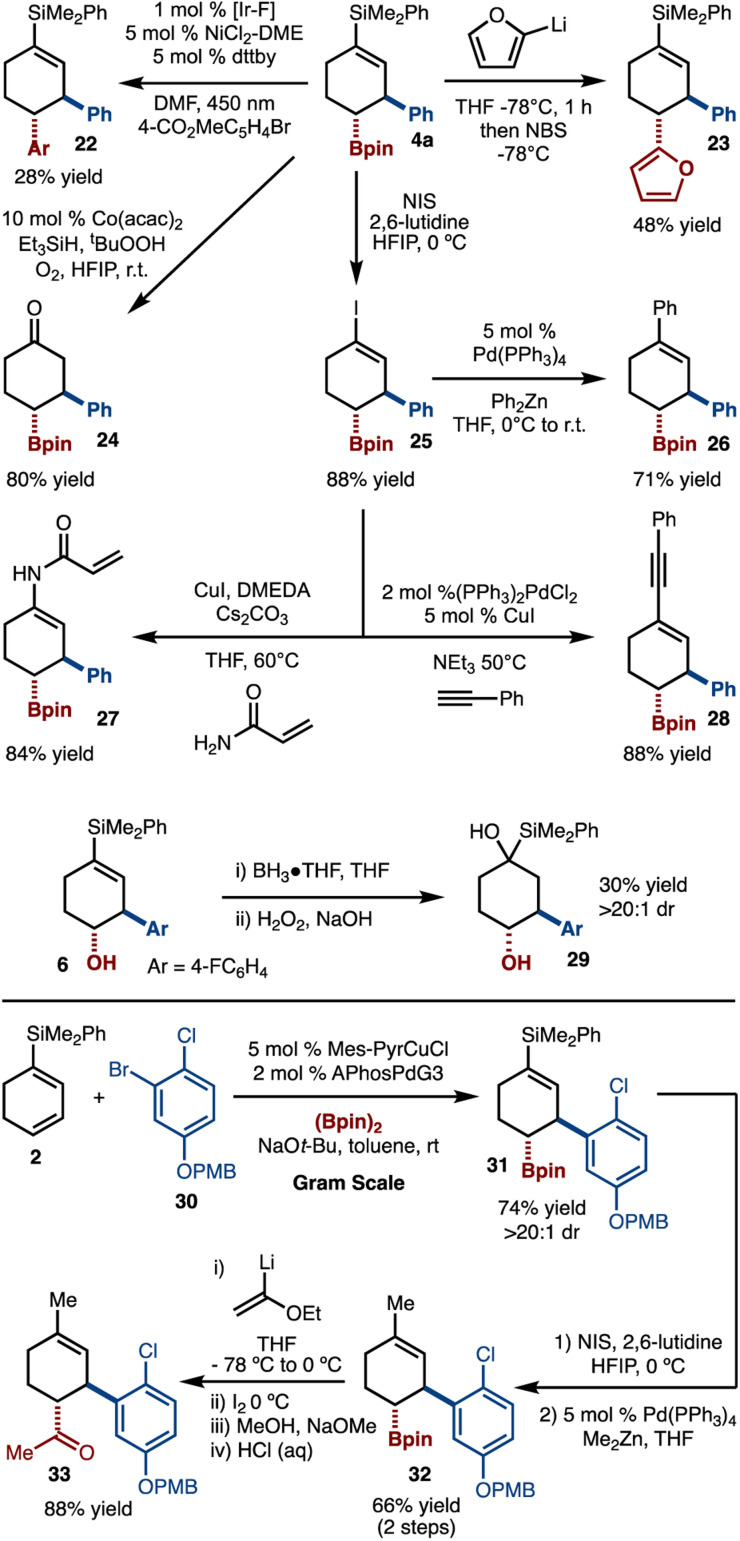
Larger scale and further functionalizations.

A key motivation behind this study was to selectively harness the reactivity of the C–B bond and vinyl-silanes to allow for a variety of products to be generated (Scheme 6). For example, metallophotoredox^16^ and transition-metal-free cross coupling^17^ could be achieved to prepare 22 and 23, respectively. Oxidation of the alkenylsilane could be achieved by Co-catalyzed oxidation to generate 24, thus realizing the β,γ-functionalization of cyclohexenone.^18^ Hiyama coupling of the vinylsilane was attempted; however, cross coupling was not observed. To address this issue a two-step protocol was devised that involved conversion to vinyl iodide 25 with NIS and HFIP.^19^ The iodide could then be subjected to Negishi,^20^ amidation,^21^ and Sonogashira^22^ reactions to provide access to 26, 27, and 28, respectively. Hydroboration of the alkenylsilane was also attempted and gave rise to 29 in 30% yield and >20 : 1 dr.^23^ In addition, we have carried out an alternative series of functionalizations. Starting with gram scale synthesis of 31, conversion by iodination and Negishi cross coupling led to 32. Finally, Zweifel reaction with lithiated ethylvinyl ether and hydrolysis led to formation of ketone 33.

## Conclusions

In summary, through the introduction of Cu/Pd-catalyzed arylboration of 1-silyl-1,3-cyclohexadiene, a new strategy to prepare diverse cyclohexanes is presented. To observe high levels of diastereoselectivity and yield, it was crucial to use a pyridylidene Cu-complex (Mes-PyrCuCl). The demonstrated synthetic transformations of the products serves to highlight the utility of these processes.

## Data availability

The ESI† contains method description, product characterization data, and NMR spectra.

## Author contributions

M. K. B. and A. R. L. conceived and initiated the project. P. F. C., A. R. L. and S. D. collected the data. All authors composed the manuscript.

## Conflicts of interest

There are no conflicts to declare.

## Supplementary Material

SC-014-D3SC02536E-s001
