# Supplementary material for: Cu/Pd-catalyzed arylboration of a 1-silyl-1,3-cyclohexadiene for stereocontrolled and diverse cyclohexane/ene synthesis
Source: Chem Sci. 2023 Sep 13;14(38):10467–70. doi: 10.1039/d3sc02536e (PMC10548526; doi:10.1039/d3sc02536e)
Supplement: SC-014-D3SC02536E-s001 [file SC-014-D3SC02536E-s001.pdf]

Electronic Supplementary Material (ESI) for Chemical Science.
This journal is © The Royal Society of Chemistry 2023

Cu/Pd-Catalyzed Arylboration of a 1-Silyl-1,3-Cyclohexadiene for Stereocontrolled and Diverse Cyclohexane/ene Synthesis

Phillip F. Crook, Alan R. Lear, Suman Das, and M. Kevin Brown*

Department of Chemistry, Indiana University, 800 E. Kirkwood Ave, Bloomington, IN 47405.

Table of Contents

General Information: 2
Reagents 2
Two Step Synthesis of Silyl-Substituted Dienes 5
  Titration of Silyl-Lithium Reagents 6
Procedure A: General Procedure for the Arylboration of Silyl-Substituted Dienes 8
Screening for the Arylboration of Silyl-Substituted Dienes 9
Scope Examples for Arylboration of Silyl-Substituted Dienes 11
Further Functionalization of the Alkyl Boronic Ester Products 17
Further Functionalization of the Vinyl Silane Products 20
Sequential Functionalization of the C-Si and C-B Bonds and Gram-Scale Utility 26
Enantioselective Example 30
Screening for the Arylboration of Silyl-Enol-Ether Diene 31
Limitations of the Method 32
Citations 33
HPLC Data and NMR Spectra 34

General Information:

Infrared (IR) spectra were recorded on Bruker Tensor II FT-IR Spectrometer, v_{max} in cm^{-1}. Bands are characterized as broad (br), strong (s) for >70% transmittance, medium (m) for 40-70% transmittance, and weak (w) for <40% transmittance. ^1H NMR spectra were recorded at room temperature on a Varian I400 (400 MHz), Varian VXR400 (400 MHz), Varian I500 (500 MHz), a Varian I600 (600 MHz) spectrometer, and/or a Bruker Ascend™ 500 MHz (equipped with cryoprobe). Chemical shifts are reported in ppm from tetramethylsilane with the solvent resonance as the internal standard ($CHCl_3$: λ 7.26 ppm). Data are reported as follows: chemical shift, multiplicity (s = singlet, d = doublet, t = triplet, q = quartet, br = broad, m = multiplet, app. = apparent), coupling constants (Hz), and integration. ^{13}C NMR spectra were recorded on a Varian I400 (100 MHz), Varian I500 (125 MHz), and/or a Bruker Ascend™ 500 MHz (125 MHz, equipped with cryoprobe) spectrometer with complete proton decoupling. Chemical shifts are reported in ppm from tetramethylsilane with the solvent resonance as the internal standard ($CDCl_3$: δ77.16 ppm). Unless otherwise noted, all reactions have been carried out with distilled and degassed solvents under an atmosphere of dry N_2 in oven- (135 °C) and flame-dried glassware with standard vacuum-line techniques. Tetrahydrofuran (THF) was purified under a positive pressure of dry argon by passage through two columns of activated alumina. Toluene was purified under a positive pressure of dry argon by passage through columns of activated alumina and Q5 (Grubbs apparatus). All work-up and purification procedures were carried out with reagent grade solvents (purchased from Sigma-Aldrich) in air. Standard column chromatography techniques were carried out using ZEOprep 60/40-63 µm silica gel. For samples that were unstable on silica, purification was done using neutral aluminum oxide (activated, Brockmann I 58 Å pore size, Oakwood). For difficult separations, medium-pressure liquid chromatography (MPLC) was performed using a Teledyne ISCO CombiFlash Rf 150 instrument. Optical rotations were measured on a PerkinElmer 241 polarimeter at 589 nm wavelength (sodium D-line) using a standard 10 cm cell (1 mL). Specific rotations, $[\alpha]_D^{20}$, are reported in degree mL/(g·dm) at the specific temperature. Concentrations (*c*) are given in grams per 100 mL of the specific solvent. Chiral HPLC analysis was performed on an Agilent 1220 Infinity LC system.

Reagents

Acrylamide was purchased from TCI and used as received.
Ad-Pyr-CuCl was prepared according to the literature.[1]
APhos was purchased from STREM and used as received.
APhos-PdG3 was prepared according to the literature.[2]
(rac)-BINAP was purchased from CombiBlocks and used as received.
BINAP-CuCl was prepared according to the literature.[3]
1,1'-Bis(diphenylphosphino)ferrocene (dppf) was purchased from STREM and used as received.
Bis(pinacolato)diboron (B_2pin_2) was purchased from Oakwood and purified by recrystallization in pentane prior to use.
4-bromoanisole was purchased from TCI and purified by neat filtration through a 2 cm pad of dry silica in a 5.75-inch pipette onto activated 4Å MS prior to use.
Bromobenzene was purchased from Sigma Aldrich and purified by neat filtration through a 2 cm pad of dry silica in a 5.75-inch pipette onto activated 4Å MS prior to use.

5-Bromobenzo[d][1,3]dioxole was purchased from Ambeed and purified by neat filtration through a 2 cm pad of dry silica in a 5.75-inch pipette onto activated 4Å MS prior to use.
3-Bromo-4-chlorophenol was purchased from CombiBlocks and used as received.
1-bromo-2-fluorobenzene was purchased from Sigma Aldrich and purified by neat filtration through a 2 cm pad of dry silica in a 5.75-inch pipette onto activated 4Å MS prior to use.
1-bromo-4-fluorobenzene was purchased from Sigma Aldrich and purified by recrystallization in EtOH prior to use.
1-bromo-4-fluorobenzene was purchased from TCI and purified by neat filtration through a 2 cm pad of dry silica in a 5.75-inch pipette onto activated 4Å MS prior to use.
3-bromofuran was purchased from CombiBlocks and purified by neat filtration through a 2 cm pad of dry silica in a 5.75-inch pipette onto activated 4Å MS prior to use.
2-bromo-6-methoxypyridine was purchased from CombiBlocks and purified by neat filtration through a 2 cm pad of dry silica in a 5.75-inch pipette onto activated 4Å MS prior to use.
5-bromo-2-methoxypyridine was purchased from CombiBlocks and purified by neat filtration through a 2 cm pad of dry silica in a 5.75-inch pipette onto activated 4Å MS prior to use.
1-Bromo-2-methylprop-1-ene was purchased from CombiBlocks and purified by neat filtration through a 2 cm pad of dry silica in a 5.75-inch pipette onto activated 4Å MS prior to use.
5-bromo-1-methyl-1H-pyrrolo[2,3-b]pyridine was prepared according to the literature.[4]
2-bromonaphthalene was purchased from Oakwood and purified by recrystallization in EtOH prior to use.
5-bromo-2-(piperidin-1-yl)pyridine was purchased from AOBChem and purified by neat filtration through a 2 cm pad of dry silica in a 5.75-inch pipette onto activated 4Å MS prior to use.
N-Bromosuccinimide was purchased from Oakwood and was purified by recrystallization in H_2O prior to use.
3-bromothiophene was purchased from Sigma Aldrich and purified by neat filtration through a 2 cm pad of dry silica in a 5.75-inch pipette onto activated 4Å MS prior to use.
2-bromotoluene was purchased from Oakwood and by neat filtration through a 2 cm pad of dry silica in a 5.75-inch pipette onto activated 4Å MS prior to use.
Cesium carbonate was purchased from STREM and used as received.
chlorodimethyl(phenyl)silane was purchased from Oakwood and was purified by short path distillation under vacuum before use.
Co(acac)$_2$ was purchased from Sigma Aldrich and dried under vacuum at 70ºC overnight prior to use.
Copper (I) chloride was purchased from STREM and purified by trituration twice with aqueous HCl, twice with EtOH, and twice with Et_2O in that order before drying under vacuum overnight.
Copper (I) iodide was purchased from Alfa Aesar and used as received.
(cyclohexa-1,3-dien-1-yloxy)triisopropylsilane was prepared according to the literature.[5]
Cyclohex-2-en-1-one was purchased from Oakwood and used as received.
N,N′-Dimethylethylenediamine was purchased from CombiBlocks and used as received.
2,4-dinitrophenyl hypochlorothioite was purchased from Sigma Aldrich and used as received.
Dppf-PdG3 was prepared according to the literature.[2]
Ethoxyethene was purchased from Sigma Aldrich and used as received.
Furan was purchased from Sigma Aldrich and used as received.
1,1,1,3,3,3-Hexafluoro-2-propanol was purchased from Oakwood and used as received.
30% H_2O_2 in H_2O was purchased from Macron and used as received.

IMes-CuCl was prepared according to the literature.[6]
Iodine was purchased from Alfa Aesar and used as received.
N-iodosuccinimide was purchased from Oakwood and used as received.
[Ir(dF(CF$_3$)ppy)$_2$(dtbbpy)]PF$_6$ was purchased from STREM and used as received.
Lithium rod was purchased from STREM and used as received.
2,6-Lutidine (2,6-dimethylpyridine) was purchased from Oakwood and used as received.
McQuade-CuCl was prepared according to the literature.[7]
Methanol Anhydrous was purchased from Neta Scientific and used as used as received.
4-Methoxybenzyl chloride was purchased from AK Scientific and used as received.
Methyl 4-bromobenzoate was purchased from CombiBlocks and used as received.
Methylcyclohex-2-en-1-one was prepared according to the literature.[8]
Methylmagnesium bromide solution (3.0 M in Et$_2$O) was purchased from Sigma Aldrich and used as received.
6-Mes-CuCl was prepared according to the literature.[9]
Morpholine was purchased from Alfa Aesar and was purified by distillation under vacuum prior to use.
Naphthyl-Pyr-CuCl was prepared according to the literature.[1]
n-butyl lithium solution (2.5 M in hexanes) was purchased from Sigma Aldrich and used as received.
NiCl$_2$·glyme was purchased from STREM and used as received.
(R,R)-NORPHOS was purchased from STREM and used as received.
NorPhos-CuCl was prepared according to the literature.
PCy$_3$-PdG3 was prepared according to the literature.[2]
Pd(PPh$_3$)$_4$ was purchased from STREM and used as received.
Potassium carbonate was purchased from EMD and dried in an oven at 80ºC overnight prior to use.
Phenylacetylene was purchased from Sigma Aldrich and used as received.
Phenylmagnesium bromide (3.0 M in Et$_2$O) was purchased from Sigma Aldrich and used as received.
(PPh$_3$)$_2$PdCl$_2$ was purchased from STREM and used as received.
QPhos was purchased from STREM and used as received.
QPhos-PdG3 was prepared according to the literature.[10]
RuPhos was purchased from Sigma Aldrich and used as received.
RuPhos-PdG3 was prepared according to the literature.[2]
SIMes-CuCl was prepared according to the literature.[6]
SIPr-CuCl was prepared according to the literature.[6]
Sodium methoxide was purchased from Sigma Aldrich and used as received.
Sodium tert-butoxide was purchased from STREM and used as received.
Sodium tert-pentoxide (NaO^{t}Amyl) was purchased from Alfa Aesar and used as received.
tert-Bu-Pyr-CuCl was prepared according to the literature[1]
tertbutyl hydrogen peroxide (5.5 M in nonane) was purchased from Sigma Aldrich and used as received.
tert-butyllithium solution (1.7M in pentane) was purchased from Sigma Aldrich and used as received.
Tricyclohexylphosphine (PCy$_3$) was purchased from STREM and used as received.
Triethylamine was purchased from EMD and purified by distillation over calcium hydride before use.

Triethylsilane (Et_3SiH) was purchased from TCI and purified by neat filtration through a 2 cm pad of dry silica in a 5.75-inch pipette onto activated 4Å MS prior to use.
(1,3,5-trimesityl-1λ^1-pyridin-2-yl)copper chloride (Mes-Pyridylidene-CuCl or Mes-Pyr-CuCl) was prepared according to the literature.[1]
XantPhos-CuCl was prepared according to the literature.[11]
XPhos was purchased from Sigma Aldrich and used as received.
XPhos-PdG3 was prepared according to the literature.[2]
Zinc (II) Chloride was purchased from Alfa Aesar and used as received.

Two Step Synthesis of Silyl-Substituted Dienes

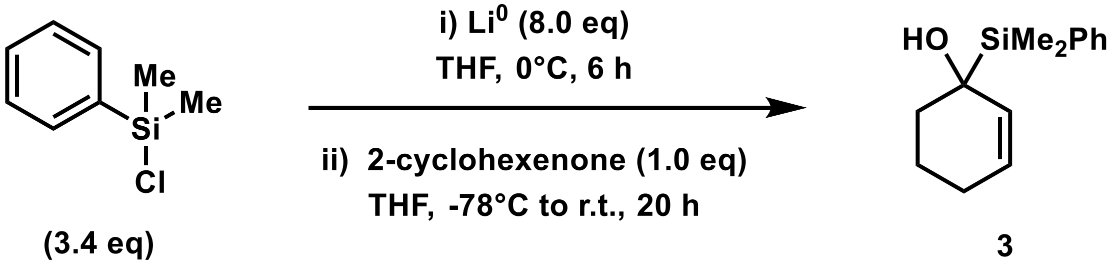

3

1-(dimethyl(phenyl)silyl)cyclohex-2-en-1-ol (3)
This procedure was adapted from the literature.[12] A 100 mL round bottom flask equipped with a magnetic stir bar was flam-dried under vacuum. Li^0 rod (1.00 g, 140 mmol, 8.00 eq) was cut up into small chunks and added to the round bottom before sealing the flask with a rubber septum and purging it with an argon balloon. Anhydrous hexanes (18 mL) was added and the solution was stirred rapidly for 15 minutes before the hexanes was removed *via* syringe. Then, anhydrous THF (18 mL) was added to the flask under argon. The mixture was then cooled to 0°C with an ice bath before adding the $Me_2PhSiCl$ (10 mL, 61 mmol, 3.4 eq). The reaction mixture was sonicated for 1 hour at 0°C, and then stirred rapidly at 0°C for 5 hours in an ice bath. A small aliquot was taken out of the reaction and titrated using the method below. 1.3 eq of the silyl lithium reagent was transferred to a flame-dried 200 mL round bottom flask dropwise over 30 min at -78°C to a solution of 2-cyclohexenone (1.7 mL, 18 mmol, 1.0 eq) in THF (18 mL). The reaction was allowed to warm to room temperature over 20 hours and then quenched slowly with H_2O (40 mL). The two phases were separated, and the aqueous phase was back-extracted twice with EtOAc (2 × 40 mL), and the combined organic layers were washed with brine (100 mL), dried with $MgSO_4$, filtered, and concentrated *in-vacuo*. The crude product was purified by MPLC with 0-8% Et_2O/Hexanes gradient as the eluent to provide 2.91 g of the title compound as a clear, light-yellow oil in 70% yield. **IR (neat):** 3444 (w, br), 3014 (w), 2934 (w), 1706 (w), 1427 (w), 1246 (m), 1108 (m), 929 (w), 905 (w), 828 (m), 805 (s), 773 (s), 731 (s), 699 (s), 655 (m), 473 (m) cm^{-1}. **^{1}H NMR (400 MHz, $CDCl_3$):** δ 7.63 – 7.57 (m, 2H), 7.40 – 7.32 (m, 3H), 5.85 (ddd, *J* = 9.9, 4.9, 2.8 Hz, 1H), 5.72 (d, *J* = 10.1 Hz, 1H), 2.07 – 1.96 (m, 1H), 1.89 – 1.77 (m, 1H), 1.74 – 1.67 (m, 2H), 1.62 – 1.46 (m, 2H), 1.19 (s, 1H), 0.36 (s, 3H), 0.35 (s, 3H) ppm. **^{13}C NMR (101 MHz, $CDCl_3$):** δ 136.5, 134.8, 130.6, 130.4, 129.4, 127.8, 64.4, 32.8, 25.3, 17.6, -5.6, -5.8 ppm. **HRMS (EI+) m/z:** matched on [M]+; Calc for $C_{14}H_{20}OSi$: 232.1278; Found: 232.1274.

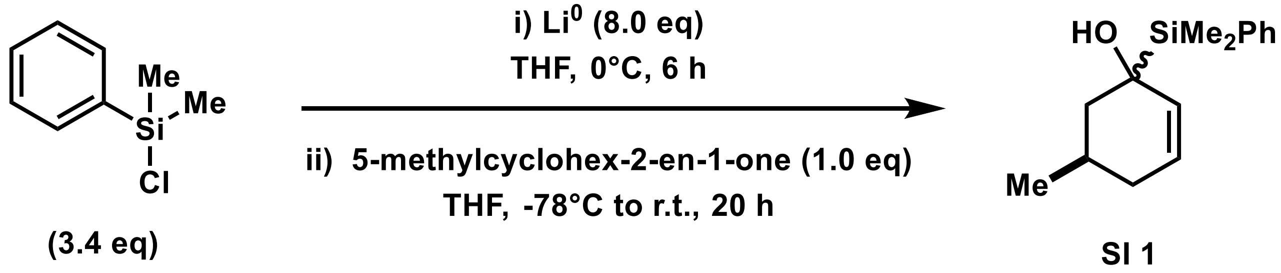

1-(dimethyl(phenyl)silyl)-5-methylcyclohex-2-en-1-ol (SI 1)

This procedure was adapted from the literature.[12] A 100 mL round bottom flask equipped with a magnetic stir bar was flam-dried under vacuum. Li^0 rod (0.60 g, 87 mmol, 8.0 eq) was cut up into small chunks and added to the round bottom before sealing the flask with a rubber septum and purging it with an argon balloon. Anhydrous hexanes (11 mL) was added and the solution was stirred rapidly for 15 minutes before the hexanes was removed *via* syringe. Then, anhydrous THF (11 mL) was added to the flask under argon. The mixture was then cooled to 0°C with an ice bath before adding the $Me_2PhSiCl$ (6.2 mL, 37 mmol, 3.4 eq). The reaction mixture was sonicated for 1 hour at 0°C, and then stirred rapidly at 0°C for 5 hours in an ice bath. A small aliquot was taken out of the reaction and titrated using the method below. 1.3 eq of the silyl lithium reagent was transferred to a flame-dried 200 mL round bottom flask dropwise over 30 min at -78°C to a solution of 5-methylcyclohex-2-en-1-one (1.2 g, 11 mmol, 1.0 eq) in THF (18 mL). The reaction was allowed to warm to room temperature over 20 hours and then quenched slowly with H_2O (20 mL). The two phases were separated, and the aqueous phase was back-extracted twice with EtOAc (2 × 20 mL), and the combined organic layers were washed with brine (50 mL), dried with $MgSO_4$, filtered, and concentrated *in-vacuo*. The crude product was partially purified by MPLC with 0-8% EtOAc/Hexanes gradient as the eluent. The final product was a mixture of two diastereomers and other unknown impurities but was moved forward and fully purified after elimination of the allylic alcohol. **^{1}H NMR (500 MHz, $CDCl_3$):** δ 7.59 (ddd, J = 9.5, 7.5, 2.1 Hz, 5H), 7.37 (h, J = 5.2 Hz, 11H), 5.87 (ddd, J = 9.7, 5.8, 2.0 Hz, 1H), 5.77 – 5.67 (m, 3H), 5.60 (d, J = 10.1 Hz, 2H), 2.13 – 2.01 (m, 4H), 2.01 – 1.94 (m, 2H), 1.78 – 1.41 (m, 10H), 1.30 – 1.17 (m, 5H), 0.99 – 0.93 (m, 5H), 0.91 – 0.85 (m, 6H), 0.38 (dd, J = 7.2, 1.7 Hz, 10H), 0.34 (dd, J = 6.6, 1.7 Hz, 6H).

Titration of Silyl-Lithium Reagents

Due to the mixture of the siyl-lithium reagent with a siloxide, a double titration method is required to determine the concentration of the silyl-lithium. This procedure was adapted from the literature.[13] Approximately 2 mg of phenolphthalein and 3 mL of DI water were added to a 10 mL round bottom flask with a magnetic stir bar. Approximately 2 mg of phenolphthalein and 3 mL of dibromomethane were added to a separate 10 mL round bottom flask with a magnetic stir bar. 0.25 mL of the silyl lithium reagent was quenched in each RBF. 2 mL of water was added to the round bottom flask with dibromomethane and it was shaken vigorously. Each RBF was titrated with 0.1 M HCl in DI water. The first titration is the concentration of the silyl-lithium reagent plus the siloxide reagent. The second titration removes the silyl-lithium reagent through lithium halogen exchange, so this titration determines the concentration of just the siloxide reagent. The difference between the two titrations is the final concentration of the silyl-lithium reagent.

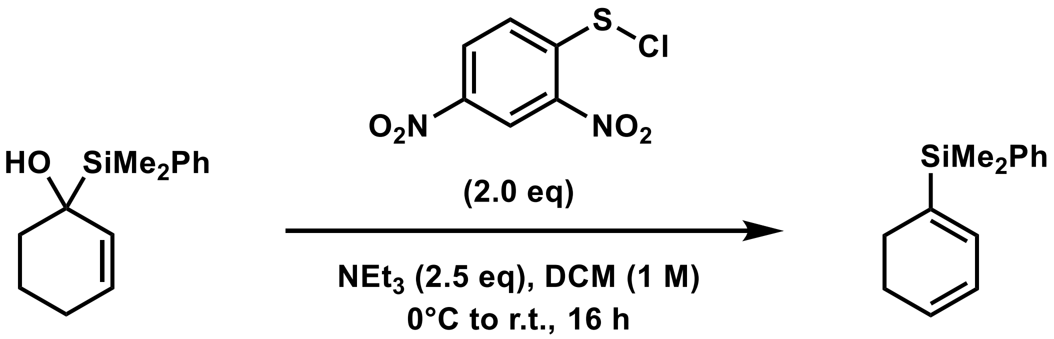

2

cyclohexa-1,3-dien-1-yldimethyl(phenyl)silane (2)

This procedure was adapted from the literature.[14] To a flame-dried, 200 mL round bottom flask equipped with a magnetic stir bar was added 1-(dimethyl(phenyl)silyl)cyclohex-2-en-1-ol (19.5 g, 83.8 mmol, 1.00 eq), triethyl amine (29.2 mL, 210. mmol, 2.50 eq), and DCM (85 mL). The solution was then cooled to 0 °C in an ice bath and 3,4-dinitrophenylhypochlorothioite (39.3 g, 168 mmol, 2.0 eq) was added slowly in portions to the reaction mixture at 0°C. The flask was sealed with a rubber septum, and the solution was then allowed to warm to room temperature and stir overnight under an atmosphere of nitrogen. The reaction mixture was then diluted with pentane (80 mL) and passed through a pad of silica gel with 100% pentane as the eluent to provide 9.3460 g of the title compound as a clear, colorless oil in 52% yield (average of two runs). **IR (neat):** 3067 (w), 3036 (w), 3016 (w), 2955 (w), 2925 (w), 2868 (w), 2819 (w), 1427 (m), 1246 (m), 1072 (m), 1038 (m), 999 (m),770 (s),748 (m), 728 (m), 695 (s), 470 (m) cm^{-1}. **^1H NMR (500 MHz, $CDCl_3$):** δ 7.58 – 7.49 (m, 2H), 7.40 – 7.30 (m, 3H), 6.24 (dq, J = 4.6, 1.5 Hz, 1H), 5.98 (ddt, J = 9.6, 4.9, 1.6 Hz, 1H), 5.91 (ddt, J = 8.5, 4.3, 2.1 Hz, 1H), 2.17 – 2.04 (m, 4H), 0.36 (s, 6H) ppm. **^{13}C NMR (126 MHz, CDCl3):** δ 138.4, 137.1, 134.2, 133.8, 129.1, 128.3, 127.9, 124.9, 24.4, 22.3, -3.5 ppm. **HRMS (EI+) m/z:** matched on [M]+; Calc for $C_{14}H_{18}Si$: 214.1172; Found: 214.1182. Note: the addition of 3,4-dinitrophenylhypochlorothioite is highly exothermic and evolves a gas, so the flask should not be sealed immediately after its addition.

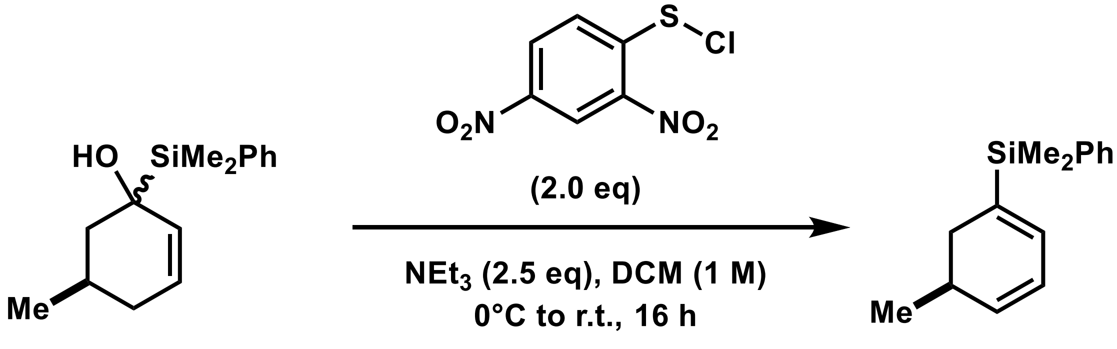

19

dimethyl(5-methylcyclohexa-1,3-dien-1-yl)(phenyl)silane (19)

This procedure was adapted from the literature.[14] To a flame-dried, 200 mL round bottom flask equipped with a magnetic stir bar was added 1-(dimethyl(phenyl)silyl)-5-methylcyclohex-2-en-1-ol (1.23 g, 4.99 mmol, 1.00 eq), triethyl amine (29.2 mL, 210. mmol, 2.50 eq), and DCM (85 mL). The solution was then cooled to 0 °C in an ice bath and 3,4-dinitrophenylhypochlorothioite

(39.3 g, 168 mmol, 2.0 eq) was added slowly in portions to the reaction mixture at 0°C. The flask was sealed with a rubber septum, and the solution was then allowed to warm to room temperature and stir overnight under an atmosphere of nitrogen. The reaction mixture was then diluted with pentane (80 mL) and passed through a pad of silica gel with 100% pentane as the eluent to provide 0.4096 g of the title compound as a clear, colorless oil in 16% yield over two steps. **IR (neat):** 3020 (w), 2954 (w), 2870 (w), 1427 (m), 1246 (m), 1107 (m), 811 (s), 726 (m), 697 (s), 468 (m), 413 (m) cm^{-1}. **^1H NMR (500 MHz, $CDCl_3$):** δ 7.56 – 7.48 (m, 2H), 7.38 – 7.31 (m, 3H), 6.23 – 6.19 (m, 1H), 5.92 (ddd, J = 9.5, 4.8, 2.1 Hz, 1H), 5.74 (dd, J = 9.5, 3.4 Hz, 1H), 2.35 – 2.19 (m, 2H), 1.92 – 1.82 (m, 1H), 0.98 (d, J = 6.8 Hz, 3H), 0.36 (s, 3H), 0.35 (s, 3H) ppm. **^{13}C NMR (126 MHz, $CDCl_3$):** δ 138.33, 136.47, 135.04, 134.17, 133.34, 129.06, 127.87, 123.86, 33.02, 27.93, 19.79, -3.51, -3.63 ppm. **HRMS (EI+) m/z:** matched on [M]+; Calc for $C_{15}H_{20}Si$: 228.1329; Found: 228.1331. Note: the addition of 3,4-dinitrophenylhypochlorothioite is highly exothermic and evolves a gas, so the flask should not be sealed immediately after its addition.

Procedure A: General Procedure for the Arylboration of Silyl-Substituted Dienes

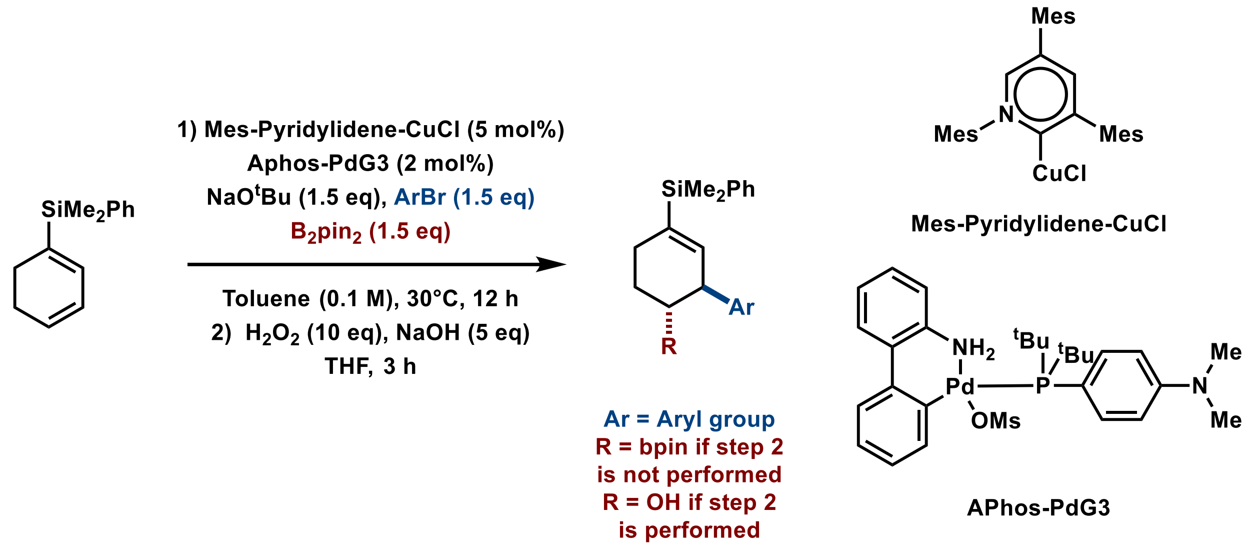

In an N_2-filled glovebox, to a flame-dried 16 × 100 mm screw cap vial with a magnetic stir bar, was added APhos-PdG3 (5.1 mg, 8.0 μmol, 2.0 mol%), Mes-Pyridylidene-CuCl (11 mg, 20. μmol, 5.0 mol%), B_2pin_2 (152 mg, 0.600 mmol, 1.50 eq), NaOtBu (57.7 mg, 0.600 mmol, 1.50 eq), and aryl bromide (0.600 mmol, 1.50 eq, if it is a solid) in that order. The 16 × 100 mm screw cap vial was sealed with a rubber septum, lined with Teflon tape, removed from the glove box, and placed under a positive pressure of N_2. To the screw cap vial was added toluene (3 mL), the silyl diene (0.40 mmol, 1.0 eq), and the aryl bromide (0.600 mmol, 1.50 eq, if it is a liquid) before rinsing the sides of the vial with toluene (1 mL). The septum was then quickly replaced with a Teflon lined screw cap and the reaction was stirred at 30°C for 12 hours. After 12 hours, the reaction was quenched with aq 1M HCl (4 mL), the two phases were separated, and the aqueous phase was back extracted with Et_2O or EtOAc (2 × 4 mL). The combined organic phases were dried with $MgSO_4$, filtered, and concentrated *in-vacuo*. (Note: NaO^{t}Amyl

or NaO^{t}Bu can be used as the base without a significant change to the yield and selectivity of the reaction.)

If R is a hydroxyl group: the crude product was redissolved in THF (2 mL). The solution was cooled to 0°C in an ice bath, and H_2O_2 (0.10 mL, 4.0 mmol, 10 eq, 30%wt in H_2O) and NaOH (2 mL, 2 mmol, 5 eq, 1 M in H_2O) were added before allowing the reaction to warm to room temperature and stir for 3 hours. The reaction was quenched with H_2O (2 mL) and diluted with Et_2O (2 mL). The two phases were separated, and the aqueous phase was back extracted twice with Et_2O (2 × 2 mL). The combine organic phases were dried over $MgSO_4$, filtered, and concentrated *in-vacuo*. The crude product mixture was purified by MPLC as described below.

Screening for the Arylboration of Silyl-Substituted Dienes

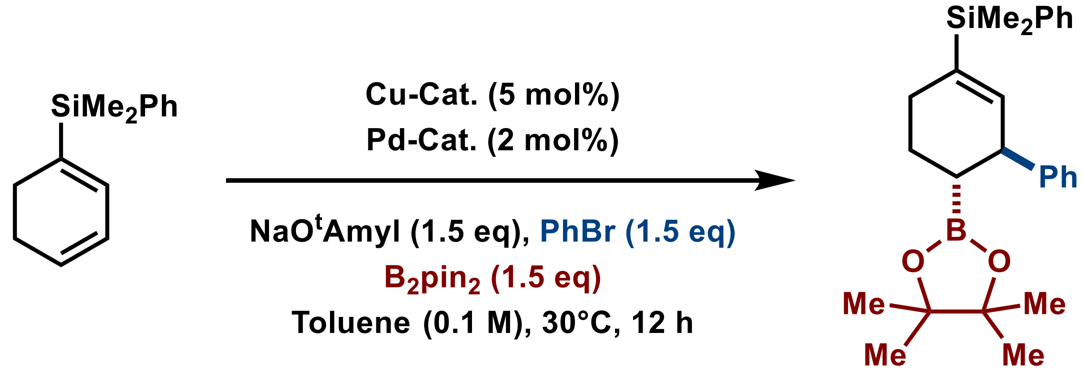

The following reactions were performed using Procedure A above with bromobenzene (0.60 mmol, 1.5 eq), no oxidation of the boronic ester, and the given copper (I) catalysts (0.02 mmol, 5 mol%) and palladium (0) precatalysts (0.008 mmol, 2 mol%). The yields of these runs were determined by GC with dodecane as the internal standard corrected by a burn factor. The best examples from the screen where also analyzed by ^1H NMR with nitromethane as the internal standard:

Entry	Pd-Cat.	Cu-Cat.	Yield	d.r.
1	dppf-PdG3	IMes-Cu-Cl	5%[a]	1:2[a]
2	RuPhos-PdG3	IMes-CuCl	<2%[a]	n.d.
3	PCy_3-PdG3	IMes-CuCl	72%[a]	3:1[a]
4	XPhos-PdG3	IMes-CuCl	63%[a]	3:1[a]
5	XPhos-PdG3	SIMes-CuCl	92%[b]	3:1[b]
6	APhos-PdG3	IMes-CuCl	78%[b]	12:1[b]
7	APhos-PdG3	SIPr-CuCl	28%[a]	6:1[a]
8	APhos-PdG3	XantPhos-CuCl	<2%[a]	n.d.
9	APhos-PdG3	NorPhos-CuCl	<2%[a]	n.d.
10	APhos-PdG3	BINAP-CuCl	<2%[a]	n.d.
11	APhos-PdG3	Mes-Pyr-CuCl	90%[b]	40:1[b]
12	APhos-PdG3	6-Mes-CuCl	74%[b]	43:1[b]
13	APhos-PdG3	^{t}Bu-Pyr-CuCl	<2%[a]	n.d.
14	APhos-PdG3	Ad-Pyr-CuCl	<2%[a]	n.d.
15	APhos-PdG3	Naphthyl-Pyr-CuCl	<2%[a]	n.d.
16	APhos-PdG3	McQuade-CuCl	93%[b]	40:1[b]

a) corrected yield and/or dr determined by GC with dodecane as the internal standard. b) yield and/or dr determined by NMR with dibromomethane as the internal standard.

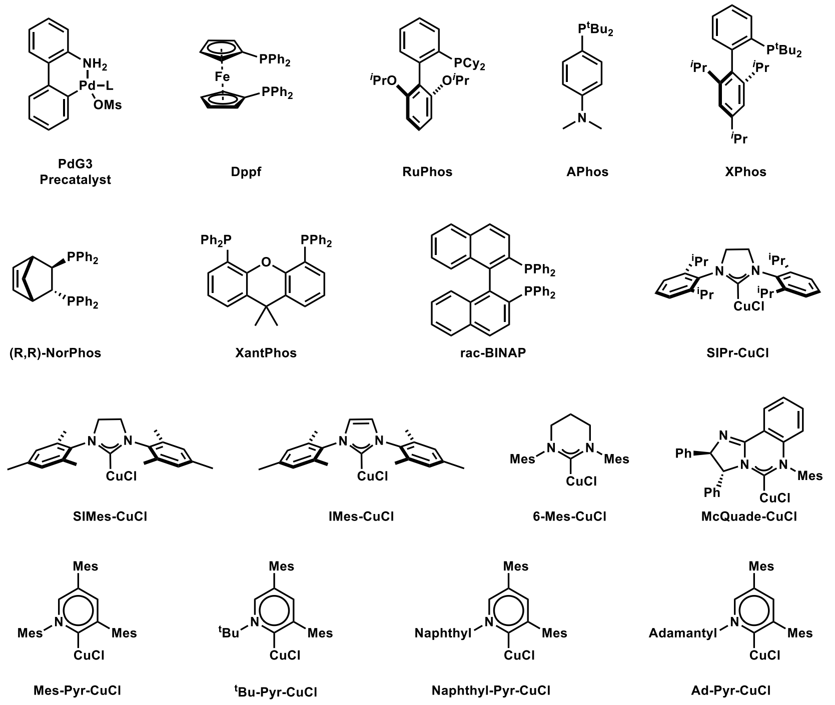

Scope Examples for Arylboration of Silyl-Substituted Dienes

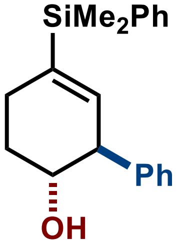

4b

(±)-5-(dimethyl(phenyl)silyl)-1,2,3,4-tetrahydro-[1,1'-biphenyl]-2-ol (4b)
The tile compound was prepared by Procedure A above with **2** (86 µL 0.40 mmol, 1.0 eq) and bromobenzene (63 µL, 0.60 mmol, 1.5 eq). The crude product was purified by MPLC with 0-10% EtOAc/Hexanes gradient as the eluent to provide 108.6 mg of the title compound as a white solid in 65% yield (average of two runs). **IR (neat):** 3259 (br, w), 3084 (w), 3065 (w), 3024 (w), 3002 (w), 2947 (w), 2928 (w), 2887 (w), 2834 (w), 1874 (w), 1809 (w), 1613 (w), 1601 (w), 1488 (w), 1450 (w), 1426 (w), 1352 (w), 1301 (w), 1245 (w), 1167 (w), 1107 (m), 1086 (w), 1069 (w), 1032 (m), 998 (w), 973 (m), 938 (w), 907 (w), 827 (m), 810 (m), 763 (w), 731 (m), 697 (s), 651 (m), 617 (w), 516 (m), 469 (w), 438 (w), 415 (w) cm^{-1}. **^1H NMR (500 MHz, $CDCl_3$):** δ 7.48 – 7.42 (m, 2H), 7.31 – 7.27 (m, 4H), 7.22 – 7.12 (m, 4H), 5.87 – 5.84 (m, 1H), 3.69 – 3.59 (m, 1H), 3.25 (ddt, J = 8.1, 4.0, 2.2 Hz, 1H), 2.29 – 2.22 (m, 1H), 2.22 – 2.12 (m, 1H), 1.96 – 1.88 (m, 1H), 1.66 – 1.55 (m, 2H), 0.29 (s, 6H) ppm. **^{13}C NMR (126 MHz, $CDCl_3$):** δ 143.1, 138.5, 138.3, 137.9, 134.0, 129.2, 128.9, 128.7, 128.0, 127.1, 73.9, 53.3, 30.2, 27.0, -3.3, -3.4 ppm. **HRMS (ESI) m/z:** matched on [M + Na]+; Calc for $C_{20}H_{24}ONaSi$: 331.1489; Found: 331.1490.

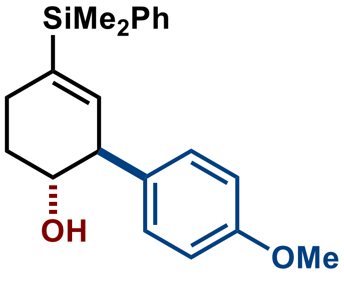

5

(±)-5-(dimethyl(phenyl)silyl)-4'-methoxy-1,2,3,4-tetrahydro-[1,1'-biphenyl]-2-ol (5)
The tile compound was prepared by Procedure A above with **2** (86 µL 0.40 mmol, 1.0 eq) and 4-Bromoanisole (75 µL, 0.60 mmol, 1.5 eq). The crude product was purified by MPLC with 0-10% EtOAc/Hexanes gradient as the eluent to provide 93.3 mg of the title compound as a white solid in 69% yield (average of two runs). **IR (neat):** 3380 (br, w), 3067 (w), 3047 (w), 3001 (w), 2953 (w), 2927 (w), 2834 (w), 1611 (w), 1509 (m), 1427 (w), 1300 (w), 1244 (m), 1034 (m), 828 (s), 805 (s), 752 (s), 729 (s), 698 (s), 655 (m), 537 (m), 473 (m), 441 (m) cm^{-1}. **^1H NMR (500 MHz, $CDCl_3$):** δ 7.55 – 7.50 (m, 2H), 7.39 – 7.33 (m, 3H), 7.17 – 7.11 (m, 2H), 6.92 – 6.87 (m, 2H), 5.94 – 5.89 (m, 1H), 3.81 (s, 3H), 3.68 (ddt, J = 11.2, 8.1, 3.3 Hz, 1H), 3.27 (dq, J = 8.1, 2.5 Hz, 1H), 2.36 – 2.29 (m, 1H), 2.29 – 2.19 (m, 1H), 1.99 (ddt, J = 12.4, 6.4, 3.3 Hz, 1H), 1.72 – 1.62 (m, 2H), 0.36 (br s, 6H) ppm. **^{13}C NMR (126 MHz, $CDCl_3$):** δ 158.8, 138.9, 138.3, 137.7, 135.0, 134.0, 129.6, 129.2, 128.0, 114.3, 74.0, 55.5, 52.4, 30.2, 27.0, -3.3, -3.4 ppm. **HRMS (APCI) m/z:** matched on [M + H]+; Calc for $C_{21}H_{27}O_2Si$: 339.1775; Found: 339.1777.

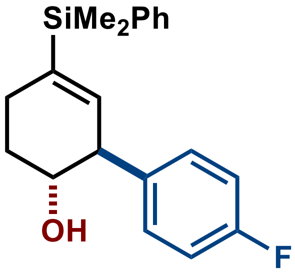

6

(±)-5-(dimethyl(phenyl)silyl)-4'-fluoro-1,2,3,4-tetrahydro-[1,1'-biphenyl]-2-ol (6)
The tile compound was prepared by Procedure A above with **2** (86 µL 0.40 mmol, 1.0 eq) and 1-Bromo-4-fluorobenzene (66 µL, 0.60 mmol, 1.5 eq). The crude product was purified by MPLC with 0-10% EtOAc/Hexanes gradient as the eluent to provide 96.6 mg of the title compound as a white solid in 74% yield (average of two runs). **IR (neat):** 3263 (br, w), 3068 (w), 2947 (w), 1885 (w), 1507 (m), 1452

(w), 1246 (m), 1154 (m), 1031 (m), 974 (m), 895 (w), 830 (m), 808 (m), 731 (m), 695 (m), 651 (m), 523 (m), 468 (m), 468 (m), 441 (m). **^{1}H NMR (500 MHz, $CDCl_3$):** δ 7.55 – 7.47 (m, 2H), 7.41 – 7.31 (m, 3H), 7.22 – 7.13 (m, 2H), 7.08 – 6.98 (m, 2H), 5.91 – 5.84 (m, 1H), 3.68 (tt, J = 11.2, 3.3 Hz, 1H), 3.31 (ddt, J = 8.1, 4.1, 2.3 Hz, 1H), 2.37 – 2.29 (m, 1H), 2.29 – 2.19 (m, 1H), 2.03 – 1.94 (m, 1H), 1.71 – 1.59 (m, 2H), 0.36 (s, 3H), 0.36 (s, 3H). **^{13}C NMR (126 MHz, $CDCl_3$):** δ 163.0, 161.1, 138.8 (d, J = 3.1 Hz), 138.3 (d, J = 3.1 Hz), 134.0, 130.0 (d, J = 7.5 Hz), 129.2, 128.0, 115.7 (d, J = 20.8 Hz), 73.9, 52.4, 30.3, 27.0, -3.3, -3.4 ppm. **^{19}F NMR (400 MHz, $CDCl_3$):** δ -116.19 ppm. **HRMS (EI+) m/z:** matched on [M - H]+; Calc for $C_{20}H_{23}FOSi$: 325.1418; Found: 325.1417.

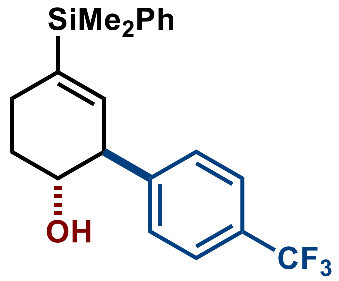

7

(±)-5-(dimethyl(phenyl)silyl)-4'-(trifluoromethyl)-1,2,3,4-tetrahydro-[1,1'-biphenyl]-2-ol (7)

The tile compound was prepared by Procedure A above with **2** (86 µL 0.40 mmol, 1.0 eq) and 1-Bromo-4-chlorobenzene (66 µL, 0.60 mmol, 1.5 eq). The crude product was purified by MPLC with 0-10% EtOAc/Hexanes gradient as the eluent to provide 120.5 mg of the title compound as a light-yellow solid in 80% yield (average of two runs). **IR (neat):** 3346 (br, w), 3068 (w), 3048 (w), 3007 (w), 2954 (w), 2923 (w), 2861 (w), 2838 (w), 1614 (w), 1427 (w), 1416 (w), 1322 (s), 1248 (m), 1161 (m), 1121 (s), 1107 (s), 1066 (m), 1017 (m), 831 (m), 809 (m), 772 (m), 730 (m), 698 (m), 656 (m), 610 (m), 588 (m), 516 (m), 474 (m), 447 (m), 415 (m) cm^{-1}. **^{1}H NMR (500 MHz, $CDCl_3$):** δ 7.61 (d, J = 7.9 Hz, 2H), 7.59 – 7.50 (m, 2H), 7.41 – 7.36 (m, 3H), 7.35 (d, J = 7.9 Hz, 2H), 5.91 – 5.85 (m, 1H), 3.73 (ddd, J = 11.1, 8.1, 3.3 Hz, 1H), 3.45 – 3.36 (m, 1H), 2.40 – 2.31 (m, 1H), 2.31 – 2.21 (m, 1H), 1.99 (ddt, J = 12.4, 6.0, 3.4 Hz, 1H), 1.75 – 1.59 (m, 2H), 0.38 (s, 6H) ppm. **^{13}C NMR (126 MHz, $CDCl_3$):** δ 147.5, 138.9, 138.0, 137.4, 134.0, 129.3 (q, J = 32.4 Hz), 129.2, 129.0, 128.0, 125.7 (q, J = 3.8 Hz), 124.4 (q, J = 271.6 Hz), 73.7, 53.0, 30.5, 27.0, -3.3, -3.4 ppm. **^{19}F NMR (376 MHz, $CDCl_3$):** δ -62.5 (s) ppm. **HRMS (ESI) m/z:** matched on [M - H]+; Calc for $C_{21}H_{22}F_3OSi$: 375.1397; Found: 375.1387.

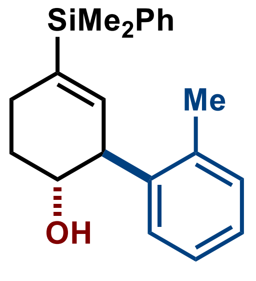

8

(±)-5-(dimethyl(phenyl)silyl)-2'-methyl-1,2,3,4-tetrahydro-[1,1'-biphenyl]-2-ol (8)

The tile compound was prepared by Procedure A above with **2** (86 µL 0.40 mmol, 1.0 eq) and 2-Bromotoluene (72 µL, 0.60 mmol, 1.5 eq). The crude product was purified by MPLC with 0-10% EtOAc/Hexanes gradient as the eluent to provide 100.6 mg of the title compound as a white solid in 78% yield (average of two runs). **IR (neat):** 3360 (br, w), 3066 (w), 2860 (w), 1650 (w), 1586 (w), 1246 (m), 997 (m), 770 (s), 754 (s), 459 (w) cm^{-1}. **^{1}H NMR (500 MHz, $CDCl_3$):** δ 7.57 – 7.49 (m, 2H), 7.39 – 7.34 (m, 3H), 7.23 – 7.10 (m, 4H), 5.93 – 5.86 (m, 1H), 3.84 (ddt, J = 10.4, 6.7, 3.1 Hz, 1H), 3.66 (dt, J = 7.6, 2.8 Hz, 1H), 2.42 (s, 3H), 2.39 – 2.30 (m, 1H), 2.30 – 2.20 (m, 1H), 1.97 (dddd, J = 12.9, 5.6, 4.2, 3.2 Hz, 1H), 1.72 (dtd, J = 12.7, 9.8, 5.9 Hz, 1H), 1.65 (br s, 1H), 0.37 (s, 6H). **^{13}C NMR (126 MHz, $CDCl_3$):** δ 141.1, 138.8, 138.3, 137.4, 137.2, 134.0, 130.7, 129.1, 128.2, 128.0, 126.8, 126.5, 73.5, 48.8, 29.9, 26.4, 20.1, -3.3, -3.4 ppm. **HRMS (EI+) m/z:** matched on [M]+; Calc for $C_{21}H_{26}OSi$: 322.1747; Found: 322.1746.

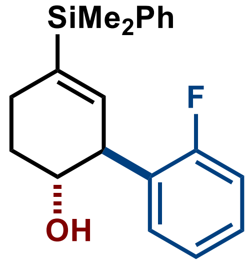

9

(±)-5-(dimethyl(phenyl)silyl)-2'-fluoro-1,2,3,4-tetrahydro-[1,1'-biphenyl]-2-ol (9)

The tile compound was prepared by Procedure A above with **2** (86 μL 0.40 mmol, 1.0 eq) and 2-Bromo-1-fluorobenzene (66 μL, 0.60 mmol, 1.5 eq). The crude product was purified by MPLC with 0-10% EtOAc/Hexanes gradient as the eluent to provide 101.9 mg of the title compound as a white solid in 78% yield (average of two runs). **IR (neat):** 3357 (br ,w), 3067 (w), 2835 (w), 1615 (w), 1584 (w), 1488 (m), 1246 (m), 1225 (m), 971 (m), 828 (s), 807 (s), 771 (s), 754 (s), 729 (s), 471 (m) cm^{-1}. **^{1}H NMR (500 MHz, $CDCl_3$):** δ 7.58 – 7.49 (m, 2H), 7.40 – 7.34 (m, 3H), 7.26 – 7.20 (m, 1H), 7.19 (dd, J = 7.4, 1.9 Hz, 1H), 7.13 (td, J = 7.4, 1.2 Hz, 1H), 7.07 (ddd, J = 9.7, 8.2, 1.3 Hz, 1H), 5.91 – 5.84 (m, 1H), 3.85 (ddd, J = 10.6, 7.6, 3.2 Hz, 1H), 3.72 (dq, J = 7.9, 2.6 Hz, 1H), 2.40 – 2.30 (m, 1H), 2.30 – 2.20 (m, 1H), 1.98 (ddt, J = 12.8, 5.6, 3.7 Hz, 1H), 1.76 – 1.65 (m, 2H), 0.38 (s, 6H) ppm. **^{13}C NMR (126 MHz, $CDCl_3$):** δ 162.5, 160.6, 138.2 (d, J = 7.2 Hz), 137.3, 134.0, 129.9 (d, J = 4.3 Hz), 129.8, 129.2, 128.5 (d, J = 8.2 Hz), 128.0, 124.5 (d, J = 3.4 Hz), 115.7 (d, J = 22.5 Hz), 72.6, 46.0, 30.3, 26.5, -3.3, -3.4 ppm. **^{19}F NMR (376 MHz, $CDCl_3$):** δ -117.98 – -118.03 (m) ppm. **HRMS (APCI) m/z:** matched on [M + H]+; Calc for $C_{20}H_{24}OFSi$: 327.1575; Found: 327.1577.

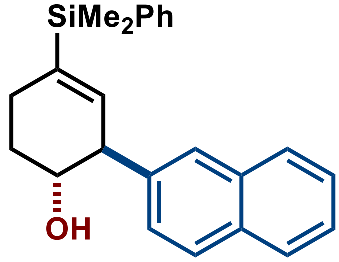

10

(±)-4-(dimethyl(phenyl)silyl)-2-(naphthalen-2-yl)cyclohex-3-en-1-ol (10)

The tile compound was prepared by Procedure A above with **2** (86 μL 0.40 mmol, 1.0 eq) and 2-Bromonaphthalene (124.2 mg, 0.60 mmol, 1.5 eq). The crude product was purified by MPLC with 0-10% EtOAc/Hexanes gradient as the eluent to provide 110.4 mg of the title compound as a white solid in 77% yield (average of two runs). **IR (neat):** 3331 (br, w), 3059 (w), 2850 (w), 1447 (w), 1303 (w), 1067 (w), 854 (m), 804 (m), 744 (m), 699 (m), 477 (m) cm^{-1}. **^{1}H NMR (500 MHz, $CDCl_3$):** δ 7.88 – 7.80 (m, 3H), 7.72 – 7.69 (m, 1H), 7.61 – 7.54 (m, 2H), 7.53 – 7.45 (m, 2H), 7.43 – 7.34 (m, 4H), 6.04 – 5.97 (m, 1H), 3.84 (ddd, J = 11.1, 8.1, 3.3 Hz, 1H), 3.51 (dq, J = 8.5, 2.7 Hz, 1H), 2.45 – 2.36 (m, 1H), 2.36 – 2.24 (m, 1H), 2.04 (ddt, J = 12.3, 5.5, 3.3 Hz, 1H), 1.82 – 1.68 (m, 2H), 0.41 (s, 3H), 0.40 (s, 3H) ppm. **^{13}C NMR (126 MHz, $CDCl_3$):** δ 140.5, 138.4, 138.3, 138.2, 134.0, 133.6, 132.7, 129.2, 128.7, 128.0, 127.8, 127.7, 127.5, 126.5, 126.3, 125.8, 73.6, 53.4, 30.2, 27.0, -3.3 ppm. **HRMS (EI+) m/z:** matched on [M]+; Calc for $C_{24}H_{26}OSi$: 358.1747; Found: 358.1750.

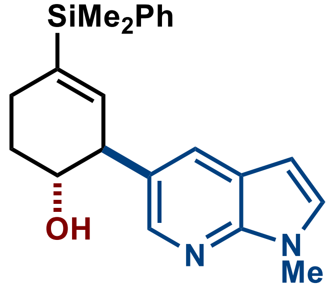

11

(±)-4-(dimethyl(phenyl)silyl)-2-(1-methyl-1H-pyrrolo[2,3-b]pyridin-5-yl)cyclohex-3-en-1-ol (11)

The tile compound was prepared by Procedure A above with **2** (86 μL 0.40 mmol, 1.0 eq) and 2-bromo-6-methoxypyridine (130 mg, 0.60 mmol, 1.5 eq). The crude product was purified by MPLC with 0-50% EtOAc/Hexanes gradient as the eluent to provide 74.0 mg of the title compound as a yellow oil in 51% yield (average of two runs). **IR (neat):** 3412 (br, w), 3034 (w), 2920 (w), 1710 (s), 1516 (w), 1404

(m), 1354 (m), 1219 (m), 1082 (w), 962 (w), 813 (m), 729 (m), 700 (m), 529 (m) cm^{-1}. **^{1}H NMR (500 MHz, $CDCl_3$):** δ 8.20 (s, 1H), 7.73 (d, J = 2.1 Hz, 1H), 7.57 – 7.49 (m, 2H), 7.41 – 7.32 (m, 3H), 7.16 (t, J = 2.6 Hz, 1H), 6.40 (d, J = 3.4 Hz, 1H), 5.99 – 5.91 (m, 1H), 3.87 (s, 3H), 3.77 (ddd, J = 11.1, 8.0, 3.2 Hz, 1H), 3.47 – 3.40 (m, 1H), 2.41 – 2.23 (m, 2H), 2.17 – 1.96 (m, 2H), 1.77 – 1.67 (m, 1H), 0.37 (s, 6H) ppm. **^{13}C NMR (126 MHz, $CDCl_3$):** δ 147.4, 143.6, 138.6, 138.2, 138.1, 134.0, 130.0, 129.7, 129.2, 128.6, 128.0, 120.7, 99.2, 74.3, 50.9, 31.4, 30.3, 27.0, -3.4 ppm. **HRMS (APCI) m/z:** matched on [M + H]+; Calc for $C_{22}H_{27}ON_2Si$: 363.1887; Found: 363.1888.

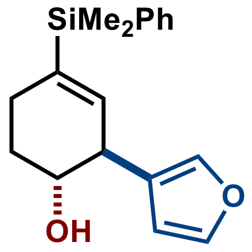

12

(±)-4-(dimethyl(phenyl)silyl)-2-(furan-3-yl)cyclohex-3-en-1-ol (12)
The tile compound was prepared by Procedure A above with **2** (86 µL 0.40 mmol, 1.0 eq) and 3-Bromofuran (54 µL, 0.60 mmol, 1.5 eq). The crude product was purified by MPLC with 0-10% EtOAc/Hexanes gradient as the eluent to provide 90.7 mg of the title compound as a light-yellow oil in 76% yield (average of two runs). **IR (neat):** 3357 (br, w), 3134 (w), 2836 (w), 1614 (w), 1427 (w), 1246 (m), 959 (m), 808 (s), 771 (s), 728 (s), 698 (s), 599 (m), 474 (m) cm^{-1}. **^{1}H NMR (500 MHz, $CDCl_3$):** δ 7.53 – 7.48 (m, 2H), 7.41 (t, J = 1.7 Hz, 1H), 7.36 (dd, J = 4.9, 1.9 Hz, 3H), 7.32 (dt, J = 1.5, 0.7 Hz, 1H), 6.32 (dd, J = 1.9, 0.9 Hz, 1H), 5.89 (s, 1H), 3.68 (ddd, J = 10.7, 7.9, 3.2 Hz, 1H), 3.25 (dq, J = 8.0, 2.5 Hz, 1H), 2.35 – 2.25 (m, 1H), 2.25 – 2.13 (m, 1H), 1.98 (ddt, J = 12.6, 5.7, 3.5 Hz, 1H), 1.78 (br s, 1H), 1.64 (dtd, J = 12.6, 10.3, 5.8 Hz, 1H), 0.36 (s, 6H) ppm. **^{13}C NMR (126 MHz, $CDCl_3$):** δ 143.6, 140.0, 138.2, 137.8, 137.6, 134.0, 129.2, 128.0, 126.5, 110.1, 72.3, 43.4, 29.9, 26.6, -3.3, -3.4 ppm. **HRMS (EI+) m/z:** matched on [M]+; Calc for $C_{18}H_{22}O_2Si$: 298.1384; Found: 298.1376.

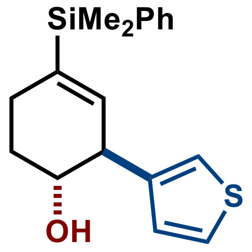

13

(±)-4-(dimethyl(phenyl)silyl)-2-(thiophen-3-yl)cyclohex-3-en-1-ol (13)
The tile compound was prepared by Procedure A above with **2** (86 µL 0.40 mmol, 1.0 eq) and 3-Bromothiophene (56 µL, 0.60 mmol, 1.5 eq). The crude product was purified by MPLC with 0-10% EtOAc/Hexanes gradient as the eluent to provide 93.1 mg of the title compound as a yellow oil in 74% yield (average of two runs). **IR (neat):** 3216 (br, w), 3132 (w), 2837 (w), 1485 (w), 1352 (w), 1243 (w), 1164 (w), 828 (m), 803 (m), 771 (m), 733 (m), 697 (m), 478 (m) cm^{-1}. **^{1}H NMR (400 MHz, $CDCl_3$):** δ 7.56 – 7.49 (m, 2H), 7.41 – 7.35 (m, 3H), 7.33 (dd, J = 4.9, 3.0 Hz, 1H), 7.08 (dd, J = 3.0, 1.3 Hz, 1H), 6.99 (dd, J = 5.0, 1.3 Hz, 1H), 5.99 – 5.93 (m, 1H), 3.73 (tt, J = 10.7, 3.0 Hz, 1H), 3.46 (dt, J = 8.1, 2.7 Hz, 1H), 2.38 – 2.28 (m, 1H), 2.28 – 2.16 (m, 1H), 1.99 (ddt, J = 12.5, 5.5, 3.5 Hz, 1H), 1.83 (br s, 1H), 1.66 (dtd, J = 13.0, 10.5, 6.1 Hz, 1H), 0.37 (s, 6H) ppm. **^{13}C NMR (101 MHz, $CDCl_3$):** δ 143.7, 138.2, 137.9, 137.6, 134.0, 129.2, 128.0, 127.5, 126.3, 121.9, 72.9, 48.3, 29.9, 26.7, -3.4 ppm. **HRMS (ESI) m/z:** matched on [M + Na]+; Calc for $C_{18}H_{22}ONaSSi$: 337.1053; Found: 337.1053.

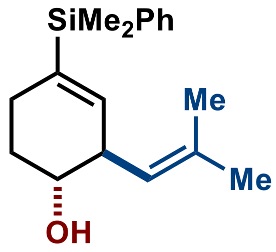

14

(±)-4-(dimethyl(phenyl)silyl)-2-(2-methylprop-1-en-1-yl)cyclohex-3-en-1-ol (14)

The tile compound was prepared by Procedure A above with **2** (86 µL 0.40 mmol, 1.0 eq) and 1-bromo-2-methylprop-1-ene (62 µL, 0.60 mmol, 1.5 eq). The crude product was purified by MPLC with 0-15% EtOAc/Hexanes gradient as the eluent to provide 66.7 mg of the title compound as a yellow oil in 58% yield (average of two runs). **IR (neat):** 3373 (br, w), 3067 (w), 2920 (w), 1609 (w), 1427 (m), 1246 (m), 1110 (m), 1043 (m), 960 (m), 828 (m), 805 (s), 770 (m), 728 (m), 698 (s), 638 (m), 471 (m) cm^{-1}. **^{1}H NMR (400 MHz, $CDCl_3$):** δ 7.55 – 7.46 (m, 2H), 7.41 – 7.31 (m, 3H), 5.74 – 5.63 (m, 1H), 5.03 – 4.91 (m, 1H), 3.50 (ddd, J = 11.1, 8.0, 3.3 Hz, 1H), 2.99 (tq, J = 10.1, 2.4 Hz, 1H), 2.30 – 2.19 (m, 1H), 2.19 – 2.07 (m, 1H), 1.98 (ddt, J = 12.3, 6.1, 3.2 Hz, 1H), 1.86 (br s, 1H), 1.79 (s, 3H), 1.73 (s, 3H), 1.63 – 1.53 (m, 1H), 0.34 (br s, 6H) ppm. **^{13}C NMR (101 MHz, $CDCl_3$):** δ 138.7, 138.4, 136.7, 135.7, 134.0, 129.0, 127.9, 125.9, 72.3, 46.2, 29.9, 27.0, 26.2, 18.5, -3.3, -3.4 ppm. **HRMS (APCI) m/z:** matched on [M - H]+; Calc for $C_{18}H_{25}OSi$: 285.1669; Found: 269.1721.

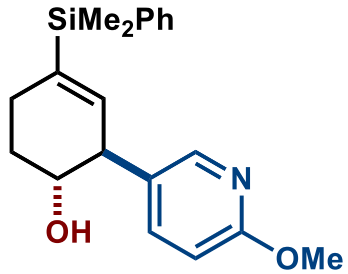

15

(±)-4-(dimethyl(phenyl)silyl)-2-(6-methoxypyridin-3-yl)cyclohex-3-en-1-ol (15)

The tile compound was prepared by Procedure A above with **2** (86 µL 0.40 mmol, 1.0 eq) and 5-bromo-2-methoxypyridine (78 µL, 0.60 mmol, 1.5 eq). The crude product was purified by MPLC with 0-30% EtOAc/Hexanes gradient as the eluent to provide 74.8 mg of the title compound as a yellow oil in 66% yield (average of two runs). **IR (neat):** 3337 (br, w), 3008 (w), 2947 (w), 1605 (m), 1490 (s), 1390 (m), 1291 (m), 1246 (m), 1111 (m), 1028 (m), 970 (m), 829 (m), 808 (s), 729 (s), 698 (s), 655 (w), 473 (m) cm^{-1}. **^{1}H NMR (600 MHz, $CDCl_3$):** δ 8.03 (d, J = 2.4 Hz, 1H), 7.54 – 7.49 (m, 2H), 7.42 (dd, J = 8.5, 2.5 Hz, 1H), 7.36 (dd, J = 4.9, 1.9 Hz, 3H), 6.74 (d, J = 8.4 Hz, 1H), 5.88 – 5.83 (m, 1H), 3.94 (s, 3H), 3.67 (ddd, J = 10.9, 8.0, 3.3 Hz, 1H), 3.29 (dt, J = 8.4, 2.8 Hz, 1H), 2.37 – 2.30 (m, 1H), 2.28 – 2.20 (m, 1H), 1.99 (ddt, J = 12.4, 5.6, 3.4 Hz, 1H), 1.75 – 1.63 (m, 2H), 0.37 (br s, 6H) ppm. **^{13}C NMR (151 MHz, $CDCl_3$):** δ 163.4, 146.6, 138.8, 138.7, 137.9, 137.7, 133.9, 131.2, 129.2, 127.9, 111.0, 73.5, 53.5, 49.7, 30.4, 26.9, -3.4, -3.5 ppm. **HRMS (ESI) m/z:** matched on [M + H]+; Calc for $C_{20}H_{26}O_2NSi$: 340.1727; Found: 340.1729.

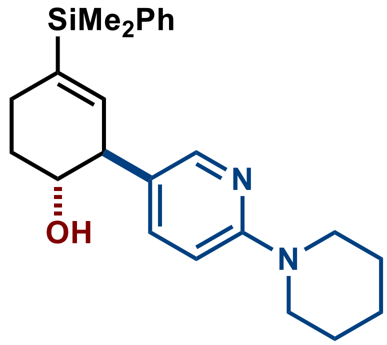

16

(±)-4-(dimethyl(phenyl)silyl)-2-(6-(piperidin-1-yl)pyridin-3-yl)cyclohex-3-en-1-ol (16)

The tile compound was prepared by Procedure A above with **2** (86 µL 0.40 mmol, 1.0 eq) and 5-bromo-2-(piperidin-1-yl)pyridine (103 µL, 0.600 mmol, 1.50 eq). The crude product was purified by MPLC with 0-15% EtOAc/Hexanes gradient as the eluent to provide 97.3 mg of the title compound as a yellow oil in 62% yield (average of two runs). **IR (neat):** 3346 (br, w), 2997 (w), 2929 (w), 2851 (w), 1604 (w), 1491 (m), 1404 (w), 1311 (w), 1241 (m), 1110 (w), 1024 (w), 932 (w), 809 (m), 770 (m), 698

(m), 471 (w) cm^{-1}. **^{1}H NMR (400 MHz, $CDCl_3$):** δ 8.03 (d, J = 2.5 Hz, 1H), 7.56 – 7.45 (m, 2H), 7.39 – 7.33 (m, 3H), 7.30 (dd, J = 8.7, 2.5 Hz, 1H), 6.63 (d, J = 8.7 Hz, 1H), 5.89 – 5.84 (m, 1H), 3.64 (ddd, J = 10.9, 8.0, 3.2 Hz, 1H), 3.49 (t, J = 4.7 Hz, 4H), 3.19 (dq, J = 8.2, 2.5 Hz, 1H), 2.37 – 2.15 (m, 2H), 2.08 (br s, 1H), 1.97 (ddt, J = 12.5, 6.6, 3.4 Hz, 1H), 1.72 – 1.58 (m, 7H), 0.35 (s, 6H) ppm. **^{13}C NMR (101 MHz, $CDCl_3$):** δ 159.0, 147.8, 138.3, 138.1, 138.0, 137.6, 133.9, 129.1, 127.9, 126.5, 107.4, 75.1, 73.5, 49.7, 46.6, 30.1, 26.8, 25.6, 24.9, 24.8, -3.4 ppm. **HRMS (ESI) m/z:** matched on [M + H]+; Calc for $C_{24}H_{33}ON_2Si$: 393.2357; Found: 393.2361.

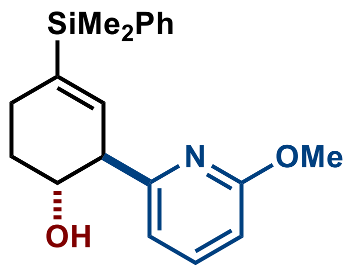

17

(±)-4-(dimethyl(phenyl)silyl)-2-(6-methoxypyridin-2-yl)cyclohex-3-en-1-ol (17)

The tile compound was prepared by Procedure A above with **2** (86 μL 0.40 mmol, 1.0 eq) and 2-bromo-6-methoxypyridine (74 μL, 0.60 mmol, 1.5 eq). The crude product was purified by MPLC with 0-15% EtOAc/Hexanes gradient as the eluent to provide 74.8 mg of the title compound as a yellow oil in 55% yield (average of two runs). **IR (neat):** 3394 (br, w), 3067 (w), 2948 (w), 1577 (m), 1464 (m), 1246 (m), 1038 (m), 802 (s), 728 (s), 698 (s), 472 (w) cm^{-1}. **^{1}H NMR (600 MHz, $CDCl_3$):** δ 7.56 (t, J = 7.3 Hz, 1H), 7.55 – 7.50 (m, 2H), 7.40 – 7.32 (m, 3H), 6.77 (d, J = 7.3 Hz, 1H), 6.65 (d, J = 8.2 Hz, 1H), 6.11 – 6.07 (m, 1H), 4.36 (s, 1H), 3.92 (s, 3H), 3.86 (ddd, J = 11.8, 8.5, 3.5 Hz, 1H), 3.45 (ddt, J = 8.4, 4.0, 2.1 Hz, 1H), 2.35 – 2.28 (m, 1H), 2.22 – 2.15 (m, 1H), 2.11 – 2.04 (m, 1H), 1.70 (qd, J = 11.8, 5.4 Hz, 1H), 0.40 (s, 3H), 0.39 (s, 3H) ppm. **^{13}C NMR (101 MHz, $CDCl_3$):** δ 163.4, 160.6, 139.7, 139.1, 138.1, 135.8, 134.0, 129.2, 127.9, 113.6, 109.0, 72.3, 53.4, 52.2, 30.5, 26.8, -3.3, -3.4 ppm. **HRMS (ESI) m/z:** matched on [M + H]+; Calc for $C_{20}H_{26}O_2NSi$: 340.1727; Found: 340.1729.

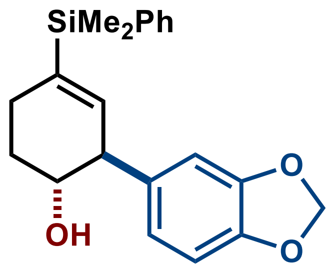

18

(±)-2-(benzo[d][1,3]dioxol-5-yl)-4-(dimethyl(phenyl)silyl)cyclohex-3-en-1-ol (18)

The tile compound was prepared by Procedure A above with **2** (86 μL 0.40 mmol, 1.0 eq) and 5-bromobenzo[d][1,3]dioxole (72 μL, 0.60 mmol, 1.5 eq). The crude product was purified by MPLC with 0-15% EtOAc/Hexanes gradient as the eluent to provide 93.1 mg of the title compound as a yellow oil in 66% yield (average of two runs). **IR (neat):** 3365 (br, w), 3008 (w), 2889 (w), 1608 (w), 1484 (s), 1427 (m), 1232 (s), 1111 (w), 1037 (s), 934 (m), 806 (s), 728 (m), 698 (s), 636 (w), 474 (w) cm^{-1}. **^{1}H NMR (600 MHz, $CDCl_3$):** δ 7.56 – 7.48 (m, 2H), 7.36 (dd, J = 4.3, 2.1 Hz, 3H), 6.81 – 6.76 (m, 1H), 6.70 (d, J = 6.6 Hz, 2H), 5.96 (d, J = 1.5 Hz, 1H), 5.95 (d, J = 1.7 Hz, 1H), 5.90 – 5.86 (m, 1H), 3.66 (ddd, J = 11.1, 8.2, 3.3 Hz, 1H), 3.25 (ddt, J = 8.2, 4.1, 2.3 Hz, 1H), 2.36 – 2.28 (m, 1H), 2.28 – 2.19 (m, 1H), 2.00 (ddt, J = 12.3, 6.1, 3.3 Hz, 1H), 1.74 – 1.61 (m, 2H), 0.37 (s, 3H), 0.37 (s, 3H) ppm. **^{13}C NMR (101 MHz, $CDCl_3$):** δ 148.1, 146.6, 138.5, 138.2, 138.0, 136.9, 134.0, 129.2, 127.9, 121.8, 108.6, 108.5, 101.2, 73.8, 52.9, 30.2, 27.0, -3.3, -3.4 ppm. **HRMS (EI+) m/z:** matched on [M]+; Calc for $C_{21}H_{24}O_3Si$: 352.1489; Found: 352.1496.

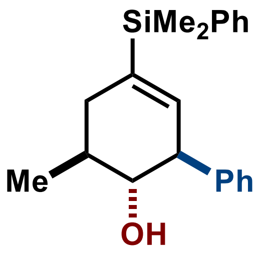

20

(±)-5-(dimethyl(phenyl)silyl)-3-methyl-1,2,3,4-tetrahydro-[1,1'-biphenyl]-2-ol (20)

The tile compound was prepared by Procedure A above with **19** (91 mg, 0.40 mmol, 1.0 eq) and bromobenzene (63 µL, 0.60 mmol, 1.5 eq). The crude product was purified by MPLC with 0-10% EtOAc/Hexanes gradient as the eluent to provide 80.0 mg of the title compound as a light-yellow oil in 62% yield (average of two runs). **IR (neat):** 3268 (br, w), 3064 (w), 2900 (w), 1615 (w), 1492 (w), 1435 (m), 1255 (m), 1108 (m), 1020 (m), 851 (m), 807 (m), 726 (m), 698 (s), 655 (m), 548 (m), 478 (w) cm^{-1}. **^1H NMR (500 MHz, $CDCl_3$):** δ 7.56 – 7.47 (m, 2H), 7.40 – 7.31 (m, 5H), 7.30 – 7.18 (m, 3H), 5.89 (s, 1H), 3.33 (d, J = 5.4 Hz, 2H), 2.31 (d, J = 14.3 Hz, 1H), 1.95 – 1.81 (m, J = 5.5, 4.0 Hz, 2H), 1.64 (br s, 1H), 1.04 (d, J = 5.5 Hz, 3H), 0.34 (s, 6H) ppm. **^{13}C NMR (126 MHz, $CDCl_3$):** δ 143.4, 139.3, 138.3, 137.6, 134.0, 129.2, 128.9, 128.7, 128.0, 127.1, 79.2, 53.8, 36.7, 35.7, 17.9, -3.3, -3.4 ppm. **HRMS (APCI) m/z:** matched on [M - OH]+; Calc for $C_{21}H_{25}Si$: 305.1720; Found: 305.1722. Note: unlike the other substrates that have a d.r.>20:1, this substrate has a d.r. of 13:1 relative to the methyl group. Stereochemistry is assumed to be trans relative to the existing stereocenter due to steric repulsion of the boryl cupration step.

Further Functionalization of the Alkyl Boronic Ester Products

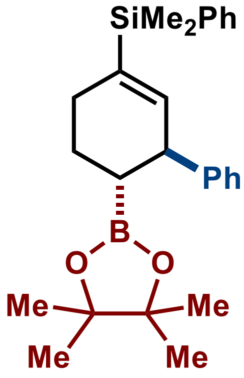

4a

dimethyl(phenyl)((±)-6-(4,4,5,5-tetramethyl-1,3,2-dioxaborolan-2-yl)-1,4,5,6-tetrahydro-[1,1'-biphenyl]-3-yl)silane (4a)

The tile compound was prepared by Procedure A above without oxidation of the boronic ester using **2** (86 µL, 0.400 mmol, 1.0 eq) and bromobenzene (63 µL, 600 µmol, 1.5 eq). The crude product was purified by MPLC with 0-2.5% EtOAc/Hexanes gradient as the eluent to provide 136.9 mg of the title compound as a colorless oil in 82% yield (average of two runs). **IR (neat):** 3050 (w), 2976 (w), 2913 (w), 1740 (w), 1600 (w), 1371 (m), 1317 (m), 1245 (m), 1142 (m), 1110 (m), 966 (w), 810 (m), 698 (s), 473 (w) cm^{-1}. **^1H NMR (600 MHz, $CDCl_3$):** δ 7.55 – 7.50 (m, 2H), 7.36 – 7.32 (m, 3H), 7.29 – 7.25 (m, 2H), 7.25 – 7.21 (m, 2H), 7.19 – 7.15 (m, 1H), 6.18 – 6.04 (m, 1H), 3.56 (dq, J = 8.9, 2.8 Hz, 1H), 2.17 – 2.11 (m, 1H), 2.11 – 2.03 (m, 1H), 1.81 – 1.74 (m, 1H), 1.62 (dddd, J = 13.0, 10.9, 9.4, 5.4 Hz, 1H), 1.25 (ddd, J = 11.6, 9.2, 2.9 Hz, 1H), 1.15 (s, 6H), 1.12 (s, 6H), 0.34 (s, 3H), 0.32 (s, 3H) ppm. **^{13}C NMR (126 MHz, $CDCl_3$):** δ 146.8, 141.3, 138.9, 137.0, 134.1, 128.9, 128.4, 128.3, 127.8, 126.1, 83.1, 44.8, 26.9, 24.9, 24.7, 23.7, -3.3, -3.4 ppm (the carbon directly bonded to boron was not seen due to quadrupole relaxation). **HRMS (APCI) m/z:** matched on [M + H]+; Calc for $C_{26}H_{36}O_2BSi$: 419.2572; Found: 419.2577.

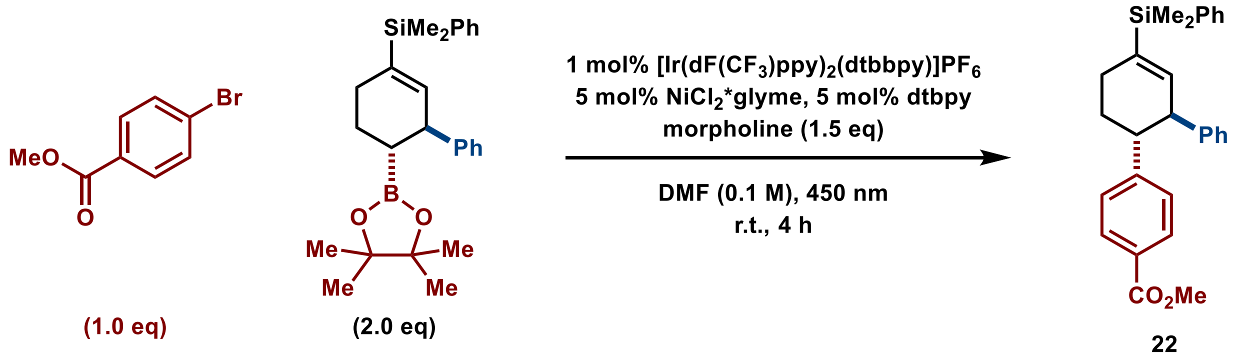

methyl(±)-4'-(dimethyl(phenyl)silyl)-1',2',5',6'-tetrahydro-[1,1':2',1''-terphenyl]-4-carboxylate (22)

This procedure was adapted from the literature.[15] In an argon filled glovebox, to a 3-dram vial with a magnetic stir bar was added **4a** (170 mg, 0.40 mmol, 2.0 eq), $[Ir(dF(CF_3)ppy)_2(dtbbpy)]PF_6$ (2.2 mg, 2.0 µmol, 5.0 mol%), and methyl-4-bromobenzoate (43 mg, 0.20 mmol, 1.0 eq). The vial was sealed with a rubber septum and removed from the glove box. Anhydrous DMF (1 mL, 0.1 M) and morpholine (26 µL, 0.30 mmol, 1.5 eq) were added to the vial. In a N_2-filled glove box, to a separate 1-dram vial was added $NiCl_2$·glyme (2.2 mg, 10. µmol, 5.0 mol%) and 4,4′-Di-tert-butyl-2,2′-dipyridyl (2.7 mg, 10. µmol, 5.0 mol%). Anhydrous DMF (1 mL) was added to the 1-dram vial, it was sonicated for 15 minutes, and then heated to 100°C with a heat gun to ensure complete complexation before transferring the solution from the 1-dram vial to the reaction mixture. The reaction was then irradiated for 4 h with 450 nm light in an IPR. The reaction was then quenched with aq LiCl (2 mL, 10% by volume) and extracted three times with Et_2O (3 × 2 mL). The combined organic layers were dried over $MgSO_4$, filtered, and concentrated *in-vacuo*. The crude product was purified by flash column chromatography with % EtOAc/Hexanes as an isocratic eluent to provide 17.1 mg of the title compound as a light-yellow oil in 20% yield. **IR (neat):** 3066 (w), 3024 (w), 2922 (w), 1720 (s), 1609 (w), 1434 (w), 1275 (s), 1180 (w), 1111 (m), 1073 (w), 815 (w), 768 (m), 699 (s), 475 (w) cm^{-1}. **^{1}H NMR (400 MHz, $CDCl_3$):** δ 7.83 (d, J = 8.2 Hz, 2H), 7.61 – 7.53 (m, 2H), 7.43 – 7.33 (m, 3H), 7.18 – 7.09 (m, 3H), 7.02 (d, J = 8.3 Hz, 2H), 6.92 – 6.83 (m, 2H), 6.17 – 6.11 (m, 1H), 3.87 (s, 3H), 3.51 (dd, J = 9.9, 2.6 Hz, 1H), 2.77 (td, J = 10.1, 4.3 Hz, 1H), 2.34 – 2.25 (m, 2H), 1.95 (dt, J = 9.7, 4.6 Hz, 1H), 0.39 (s, 3H), 0.38 (s, 3H) ppm. **^{13}C NMR (126 MHz, $CDCl_3$):** δ 167.2, 151.0, 144.1, 140.4, 138.3, 137.5, 133.9, 129.4, 129.0, 128.3, 128.1, 127.9, 127.8, 127.6, 126.2, 51.9, 51.2, 49.4, 30.0, 27.5, -3.4, -3.5 ppm. **HRMS (APCI) m/z:** matched on [M + H]+; Calc for $C_{28}H_{31}O_2Si$: 427.2088; Found: 427.2088.

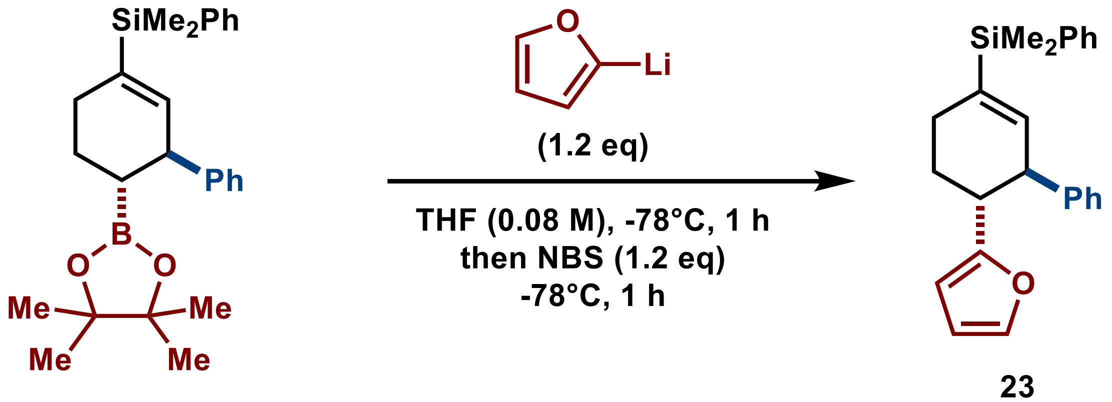

23

((±)-6-(furan-2-yl)-1,4,5,6-tetrahydro-[1,1'-biphenyl]-3-yl)dimethyl(phenyl)silane (23)

This procedure was adapted from the literature.[16] A solution of furan (7.1 mg, 95 µmol, 1.2 eq) in THF (0.25 mL) was cooled to –78 °C and treated with n-BuLi (38 µL, 95 µmol, 1.2 eq, 2.5 M in hexanes). The cooling bath was removed, and the mixture was stirred at room temperature for 1 hour. The mixture was cooled to –78 °C and **4a** (33.2 mg, 79.3 µmol, 1.00 eq) was added dropwise as a solution in THF (0.50 mL). The mixture was stirred at –78 °C for 1 h. A solution of N-bromosuccinimide (17 mg, 95 µmol, 1.2 eq) in THF (0.25 mL) was added dropwise. After 1 hour at –78 °C, a saturated aqueous solution of $Na_2S_2O_3$ (1 mL) was added and the reaction mixture was allowed to warm to room temperature. The reaction mixture was diluted with Et_2O (3 mL) and H_2O (3 mL). The layers were separated, and the aqueous layer was back extracted twice with Et_2O (2 × 4 mL). The combined organic layers were dried over $MgSO_4$, filtered, and concentrated *in-vacuo*. The crude product was purified by MPLC with 0-2% EtOAc/Hexanes gradient as the eluent to provide 13.6 mg of the title compound as a yellow oil in 48% yield. **IR (neat):** 3066 (w), 3025 (w), 2924 (w), 1600 (w), 1491 (w), 1427 (w), 1247 (w), 1111 (w), 998 (w), 814 (m), 728 (m), 699 (s), 598 (w), 472 (w) cm^{-1}. **^{1}H NMR (400 MHz, $CDCl_3$):** δ 7.58 – 7.51 (m, 2H), 7.40 – 7.33 (m, 3H), 7.26 – 7.21 (m, 3H), 7.20 – 7.14 (m, 1H), 7.07 – 7.01 (m, 2H), 6.18 (dd, J = 3.2, 1.8 Hz, 1H), 6.12 – 6.06 (m, 1H), 5.78 (d, J = 3.2 Hz, 1H), 3.70 – 3.60 (m, 1H), 2.88 (td, J = 10.3, 3.2 Hz, 1H), 2.27 – 2.18 (m, 2H), 1.98 (dq, J = 13.4, 4.7 Hz, 1H), 1.94 – 1.84 (m, 1H), 0.37 (s, 6H), 0.36 (s, 3H) ppm. **^{13}C NMR (126 MHz, $CDCl_3$):** δ 158.5, 144.8, 140.7, 140.0, 138.5, 137.4, 134.1, 129.1, 128.3, 128.2, 127.9, 126.4, 110.0, 104.9, 48.5, 42.3, 28.0, 26.9, -3.3, -3.4 ppm. **HRMS (APCI) m/z:** matched on [M + H]+; Calc for $C_{24}H_{27}OSi$: 359.1826; Found: 359.1829.

Further Functionalization of the Vinyl Silane Products

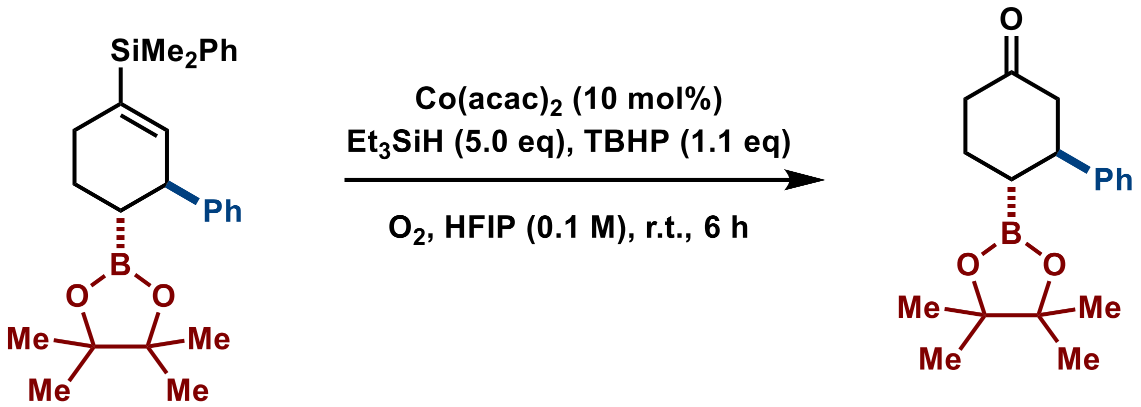

24

(±)-3-phenyl-4-(4,4,5,5-tetramethyl-1,3,2-dioxaborolan-2-yl)cyclohexan-1-one (24)

This procedure was adapted from the literature.[17] A flame-dried 16 × 100 mm screw cap vial with a magnetic stir bar was charged with $Co(acac)_2$ (2.1 mg, 8.0 µmol, 10 mol%) in a N_2-filled glove box. The vial was sealed with a rubber septum and lined with Teflon tape. The vial was then removed from the glove box and placed under a positive pressure of N_2. The vial was then charged sequentially with **4a** (33.3 mg, 80.0 µmol, 1.00 equiv), 1,1,1,3,3,3-Hexafluoro-2-propanol (1 mL, 0.1 M), triethylsilane (64 µL, 0.40 mmol, 5.0 equiv), and a solution of tert-butyl hydroperoxide in nonane (16 µL, 0.25 mmol, 1.1 equiv, ~5.5 M). The vial was then purged three times with a balloon of O_2. The reaction mixture was stirred at room temperature for 6 hours with a O_2 balloon. The product mixture was concentrated to dryness, the residue obtained was filtered through a short column of silica gel, and the short column was rinsed with 50% EtOAc/Hexanes (100 mL). The filtrates were combined, and the combined filtrates were concentrated. The crude product was purified by flash column chromatography with 10% EtOAc/Hexanes as an isocratic eluent to provide 19.1 mg of the title compound as a white solid in 80% yield. **IR (neat):** 3028 (w), 2976 (w), 2924 (w), 1713 (m), 1372 (m), 1324 (m), 1141 (s), 975 (m), 853 (m), 758 (m), 698 (m), 669 (m), 543 (w), 496 (w), 443 (w) cm^{-1}. **^{1}H NMR (500 MHz, $CDCl_3$):** δ 7.31 – 7.25 (m, 2H), 7.25 – 7.21 (m, 2H), 7.21 – 7.16 (m, 1H), 3.04 (td, J = 12.1, 4.1 Hz, 1H), 2.57 (ddd, J = 14.1, 4.2, 1.9 Hz, 1H), 2.50 – 2.44 (m, 2H), 2.36 (td, J = 12.9, 6.2 Hz, 1H), 2.15 (ddt, J = 12.7, 6.1, 3.1 Hz, 1H), 1.78 (qd, J = 12.9, 4.4 Hz, 1H), 1.65 (td, J = 11.9, 3.1 Hz, 1H), 0.99 (s, 6H), 0.96 (s, 6H) ppm. **^{13}C NMR (126 MHz, $CDCl_3$):** δ 211.1, 144.4, 128.6, 127.4, 126.8, 83.3, 50.8, 47.1, 42.0, 27.7, 24.5 ppm (the carbon directly bonded to boron was not seen due to quadrupole relaxation, one carbon is missing likely due to methyl groups on the pinacol having the same chemical shift). **HRMS (EI+) m/z:** matched on [M]+; Calc for $C_{18}H_{25}O_3B$: 300.1895; Found: 300.1901.

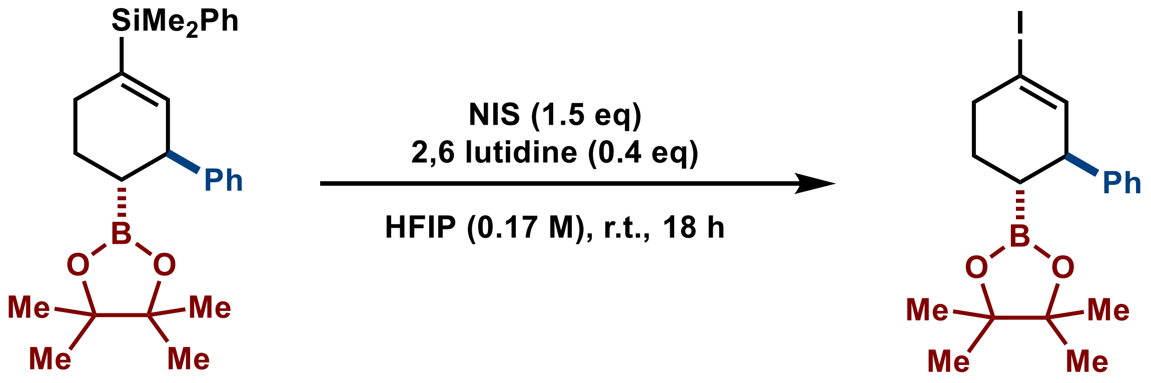

25

2-((±)-5-iodo-1,2,3,4-tetrahydro-[1,1'-biphenyl]-2-yl)-4,4,5,5-tetramethyl-1,3,2-dioxaborolane (25)

This procedure was adapted from the literature.[18] To a flame-dried round bottom flask with a magnetic stir bar was added **4a** (0.299 g, 714 μmol, 1.00 eq), 1,1,1,3,3,3-Hexafluoro-2-propanol (4.2 mL), and 2,6-dimethylpyridine (33 μL, 286 μmol, 0.40 eq). The solution was then cooled to 0°C, and N-iodosuccinimide (241 mg, 1.07 mmol, 1.50 eq) was added in one portion. The reaction flask was purged with N_2 and allowed to warm to room temperature while stirring for 18 hours. After 18 hours, the reaction mixture was diluted with DCM (5 mL), and the organic phase was washed with saturated sodium thiosulfate (5 mL), then saturated sodium bicarbonate (5 mL), and finally brine (5 mL). The organic layer was dried with magnesium sulfate, filtered, and concentrated *in-vacuo.* The crude product was purified by MPLC with 0 to 2% EtOAc/Hexanes gradient as the eluent to provide 262.9 mg of the title compound as a light-yellow oil in 88% yield (average of two runs). **IR (neat):** 3061 (w), 3027 (w), 2977 (w), 2926 (w), 2883 (w), 2246 (w), 1991 (w), 1722 (w), 1669 (w), 1631 (w), 1601 (w), 1451 (w), 1411 (w), 1377 (m), 1320 (m), 1257 (w), 1166 (w), 1141 (s), 1016 (w), 970 (m), 908 (m), 876 (w), 852 (m), 756 (w), 730 (s), 699 (s), 670 (m), 647 (m), 606 (w), 578 (w), 550 (w), 537 (w), 504 (w), 483 (w), 454 (w) cm^{-1}. **^{1}H NMR (500 MHz, $CDCl_3$):** δ 7.29 – 7.23 (m, 2H), 7.23 – 7.15 (m, 3H), 6.41 – 6.37 (m, 1H), 3.59 (dq, J = 9.1, 3.0 Hz, 1H), 2.66 – 2.55 (m, 2H), 1.82 – 1.75 (m, 2H), 1.34 – 1.28 (m, 1H), 1.14 (s, 6H), 1.11 (s, 6H) ppm. **^{13}C NMR (126 MHz, $CDCl_3$):** δ 144.9, 141.3, 128.4, 128.1, 126.5, 97.7, 83.4, 47.4, 39.4, 26.0, 24.8, 24.7 ppm (the carbon directly bonded to boron was not seen due to quadrupole relaxation). **HRMS (APCI) m/z:** matched on [M + H]+; Calc for $C_{18}H_{25}O_2BI$: 411.0987; Found: 411.0986.

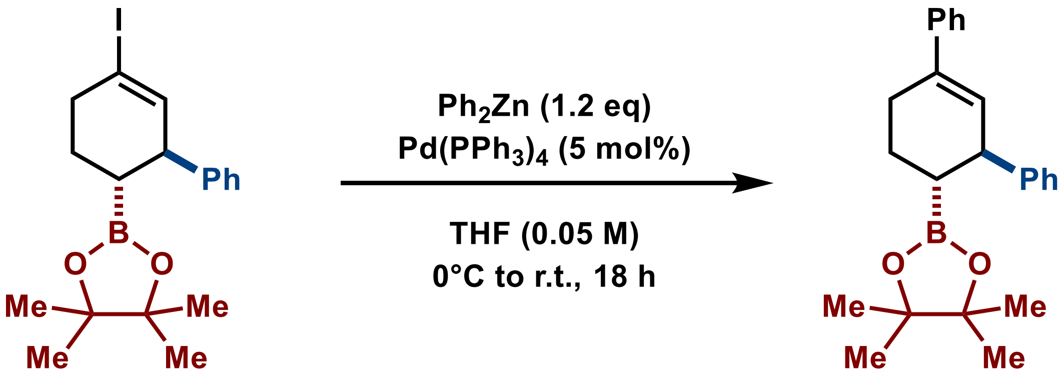

26

4,4,5,5-tetramethyl-2-((±)-3',4',5',6'-tetrahydro-[1,1':3',1''-terphenyl]-4'-yl)-1,3,2-dioxaborolane (26)

To a flame-dried 2-dram vial with a magnetic stir bar was added activated $ZnCl_2$ (55.0 mg, 404 μmol, 2.0 eq) in a N_2-filled glovebox. To a separate flame-dried 10 mL round bottom flask with a magnetic stir bar was added $Pd(PPh_3)_4$ (11.7 mg, 10.1 μmol, 5.00 mol%) in a N_2-filled glovebox. Both the 2-dram vial and round bottom flask were capped with rubber septa, lined with Teflon tape, and removed from the glovebox. Anhydrous THF (0.40 mL) was added to the 2-dram vial, and it was cooled to 0°C in an ice bath. PhMgBr (0.808 mL, 808 μmol, 4.00 eq, 1 M in THF) was added to the 2-dram vial and the solution was heated and stirred for 2 hours at 60°C. To the 10 mL round bottom flask was added anhydrous THF (4 mL) and **25** (82.8 mg, 202 μmol, 1.00 eq). The solids were allowed to settle in the 2-dram vial before transferring the supernatant dropwise to the 10 mL round bottom flask. The solid in the 2-dram vial was rinsed with another portion of anhydrous THF (0.40 mL) before letting the solid settle and transferring the supernatant dropwise to the 10 mL round bottom flask. The reaction was covered with aluminum foil and allowed to stir at room temperature overnight. After 18 hours, the reaction was quenched with water (4 mL), the two phases were separated, and the aqueous phase was back extracted with Et_2O (2 × 4 mL). The combined organic phases were washed with brine (12 mL), dried with magnesium sulfate, filtered, and concentrated *in-vacuo*. The crude product purified by MPLC with 0-2% EtOAc/Hexanes gradient as the eluent to provide 51.7 mg of the title compound as a light-yellow oil in 71% yield (average of two runs). **IR (neat):** 3057 (w), 3027 (w), 2977 (w), 2924 (w), 2857 (w), 1599 (w), 1493 (w), 1450 (w), 1409 (w), 1380 (m), 1315 (m), 1269 (w), 1214 (w), 1140 (s), 1108 (w), 1074 (w), 1028 (w), 1003 (w), 975 (m), 908 (w), 880 (w), 856 (m), 750 (m), 697 (s), 671 (m), 579 (w), 537 (w), 455 (w) cm^{-1}. **^{1}H NMR (500 MHz, $CDCl_3$):** δ 7.44 – 7.40 (m, 2H), 7.32 – 7.26 (m, 3H), 7.26 – 7.19 (m, 4H), 7.16 (ddd, J = 8.6, 5.7, 2.7 Hz, 1H), 6.16 – 6.12 (m, 1H), 3.68 (dq, J = 9.2, 3.0 Hz, 1H), 2.57 – 2.44 (m, 2H), 2.02 – 1.95 (m, 1H), 1.82 – 1.72 (m, 1H), 1.32 – 1.24 (m, 1H), 1.16 (s, 6H), 1.12 (s, 6H) ppm. **^{13}C NMR (126 MHz, $CDCl_3$):** δ 146.8, 142.4, 137.0, 128.9, 128.4, 128.3, 128.2, 126.9, 126.2, 125.3, 83.3, 44.4, 27.5, 24.9, 24.7, 24.1 ppm (the carbon directly bonded to boron was not seen due to quadrupole relaxation). **HRMS (APCI) m/z:** matched on [M + H]+; Calc for $C_{24}H_{30}O_2B$: 361.2333; Found: 361.2337.

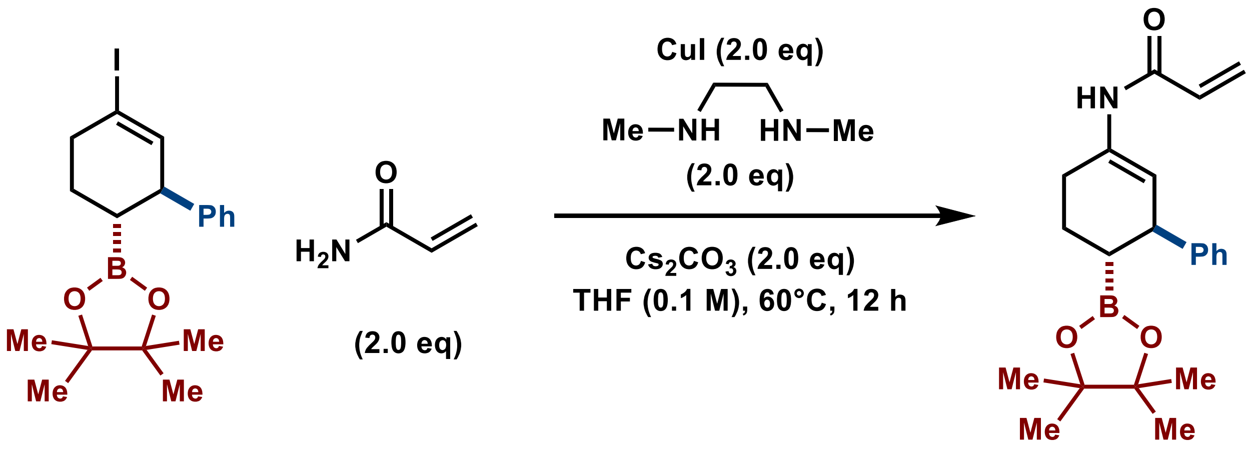

27

***N*-((±)-6-(4,4,5,5-tetramethyl-1,3,2-dioxaborolan-2-yl)-1,4,5,6-tetrahydro-[1,1'-biphenyl]-3-yl)acrylamide (27)**

This procedure was adapted from the literature.[19] To a flame-dried 16 × 100 mm screw cap vial with a magnetic stir bar, in an N_2-filled glove box, was added CuI (38.1 mg, 200. µmol, 2.00 eq), acrylamide (14.2 mg, 200. µmol, 2.00 eq), and Cs_2CO_3 (65.1 mg, 200. µmol, 2.00 eq). The vial was sealed with a rubber septum and Teflon tape and then removed from the glove box and placed under a positive pressure of N_2. **25** (41.0 mg, 100. µmol, 1.00 eq) in anhydrous THF (1 mL) and *N,N'*-Dimethylethylenediamine (21.5 µL, 200. µmol, 2.00 eq) were added. The rubber septum was quickly replaced with a Teflon-lined screw cap and the reaction was allowed to stir at 60°C for 12 h. The reaction was cooled to room temperature and filtered through celite. The reaction was then diluted with Et_2O (1 mL) and washed with water twice (2 × 2 mL). The organic phase was dried with magnesium sulfate, filtered, and concentrated *in-vacuo*. The crude product was purified by flash column chromatography with 30% EtOAc/Hexanes as an isocratic eluent to provide 29.7 mg of the title compound as a yellow oil in 84% yield (average of two runs). **IR (neat):** 3267 (w), 2975 (w), 2928 (w), 2859 (w), 2363 (w), 2334 (w), 2160 (m), 1978 (m), 1869 (w), 1698 (w), 1661 (w), 1618 (w), 1541 (w), 1377 (m), 1230 (w), 1141 (m), 969 (w), 855 (w), 799 (w), 758 (w), 700 (m), 699 (w), 578 (w), 538 (w), 457 (w), 419 (m) cm^{-1}. **^1H NMR (500 MHz, $CDCl_3$):** δ 7.26 – 7.21 (m, 4H), 7.18 – 7.13 (m, 1H), 6.43 (s, 1H), 6.31 (d, J = 16.8 Hz, 1H), 6.10 (s, 2H), 5.66 (dd, J = 10.2, 1.4 Hz, 1H), 3.63 (dq, J = 9.1, 3.0 Hz, 1H), 2.47 – 2.29 (m, 2H), 1.89 (ddt, J = 12.7, 6.1, 3.3 Hz, 1H), 1.78 – 1.68 (m, 1H), 1.29 – 1.19 (m, 1H), 1.15 (s, 6H), 1.12 (s, 6H). **^{13}C NMR (126 MHz, $CDCl_3$):** δ 163.5, 146.4, 133.6, 131.5, 128.2, 127.1, 126.2, 117.8, 83.3, 43.0, 29.8, 28.1, 24.9, 24.7, 23.6 (the carbon directly bonded to boron was not seen due to quadrupole relaxation). **HRMS (ESI) m/z:** matched on [M + Na]+; Calc for $C_{21}H_{28}O_3NBNa$: 376.2054; Found: 376.2058.

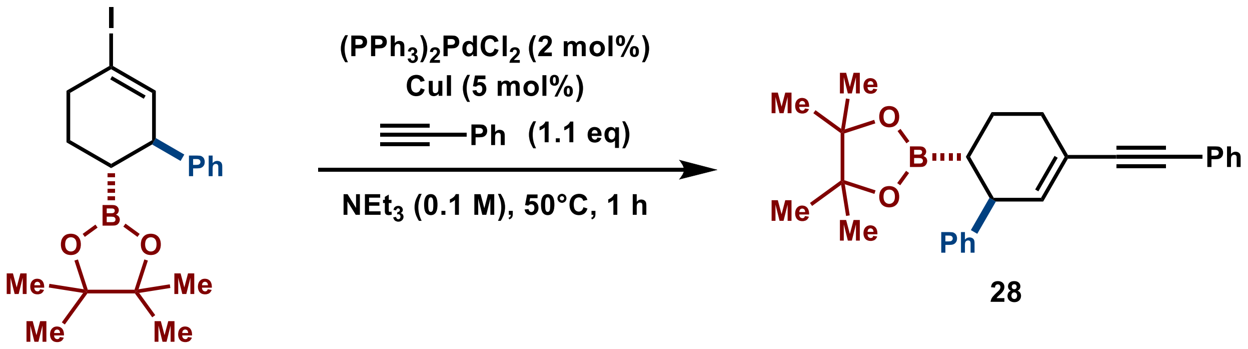

28

4,4,5,5-tetramethyl-2-((±)-5-(phenylethynyl)-1,2,3,4-tetrahydro-[1,1'-biphenyl]-2-yl)-1,3,2-dioxaborolane (28)

This procedure was adapted from the literature.[20] To a flame-dried 16 × 100 mm screw cap vial with a magnetic stir bar was added **25** (35.1 mg, 85.6 µmol, 1.00 eq) as a solution in anhydrous DCM. The DCM was removed under vacuum and the vial was then taken into a N_2-filled glove box where CuI (0.8 mg, 4 µmol, 5 mol%), and $(PPh_3)_2PdCl_2$ (1.2 mg, 1.7 µmol, 2.0 mol%) were added. The vial was then sealed with a rubber septum and Teflon tape, removed from the glove box, and put under a positive pressure of N_2. Phenylacetylene (10 µL, 94. µmol, 1.1 eq) and triethylamine (1 mL, freshly distilled over CaH_2) were added to the vial, and the reaction was stirred at 50°C for 1 hour. The reaction was then cooled back to room temperature and filtered with Et_2O. The solvent was removed *in-vacuo*, and the crude product was redissolved in Et_2O (2 mL). Water (2 mL) was added, and the two phases were separated. The aqueous phase was then back extracted twice with Et_2O (2 × 2 mL). The combined organic layers were then washed once with Brine (6 mL), dried over $MgSO_4$, filtered, and concentrated *in-vacuo*. The crude product was purified by flash column chromatography with 2.5% EtOAc/Hexanes as an isocratic eluent to provide 29.0 mg of the title compound as a clear, colorless oil in 88% yield (average of two runs). **IR (neat):** 3026 (w), 2979 (w), 2923 (w), 1597 (w), 1490 (w), 1443 (w), 1410 (w), 1378 (m), 1322 (m), 1264 (m), 1232 (w), 1213 (w), 1166 (w), 1141 (m), 1107 (w), 1071 (w), 1027 (w), 968 (w), 914 (w), 882 (w), 856 (w), 735 (s), 701 (m), 691 (m), 578 (w), 523 (w), 484 (w), 450 (w) cm^{-1}. **^{1}H NMR (500 MHz, $CDCl_3$):** δ 7.45 – 7.39 (m, 2H), 7.32 – 7.26 (m, 4H), 7.26 – 7.23 (m, 3H), 7.20 – 7.16 (m, 1H), 6.22 (dd, J = 4.7, 2.0 Hz, 1H), 3.61 (dq, J = 9.4, 3.1 Hz, 1H), 2.38 – 2.25 (m, 2H), 1.89 (dtd, J = 11.9, 4.6, 2.9 Hz, 1H), 1.77 – 1.67 (m, 1H), 1.29 – 1.23 (m, 1H), 1.15 (s, 6H), 1.13 (s, 6H) ppm. **^{13}C NMR (126 MHz, $CDCl_3$):** δ 145.7, 138.9, 131.6, 128.4, 128.3, 127.9, 126.4, 123.8, 121.2, 91.2, 87.5, 83.3, 44.3, 29.4, 24.9, 24.7, 23.7 ppm (the carbon directly bonded to boron was not seen due to quadrupole relaxation, one carbon is missing likely due to methyl groups on the pinacol having the same chemical shift). **HRMS (ESI) m/z:** matched on [M + H]+; Calc for $C_{26}H_{30}O_2B$: 385.2333; Found: 385.2334.

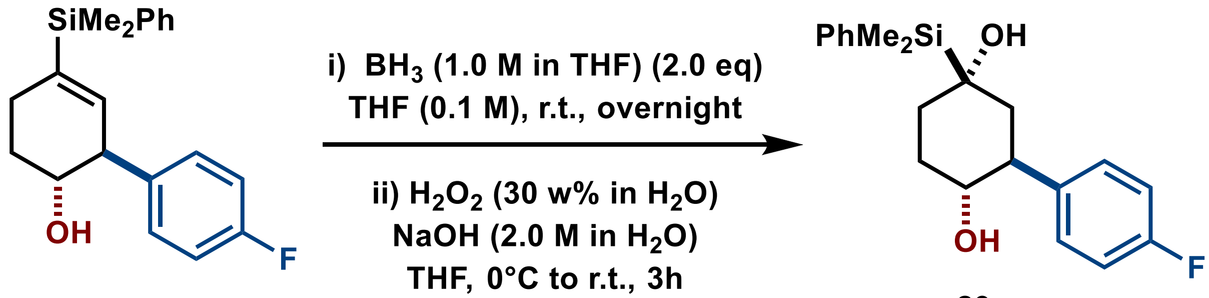

29

(3*S*,4*R*)-1-(dimethyl(phenyl)silyl)-3-(4-fluorophenyl) cyclohexane-1,4-diol (29)
The title compound was prepared according to the literature procedure.[21] In a flame dried reaction tube, **6** (85 mg, 0.26 mmol, 1.0 eq.) dissolved in 3 mL of THF under N_2 atmosphere at 0 °C, BH_3/THF (0.52 mL, 0.52 mmol, 2.0 eq. 1M solution in THF) was added drop wise. The reaction was warmed room temperature and stirred for overnight. Then solvent was evaporated under reduced pressure and crude material was directly used for next step.

Next, the crude material from previous step was dissolved in 2 mL of THF followed by addition of 2M NaOH solution. Then, the reaction mixture was cooled to 0°C, and 1.5 mL of 30% aqueous H_2O_2 solution was added. The reaction mixture was allowed to warm at room temperature and stir for 3 hours. The reaction was diluted with H_2O (5 mL) then extracted with diethyl ether (3 x 5 mL). The combined organic layers were dried over Na_2SO_4, filtered, and concentrated *in-vacuo*. The crude mixture was purified by flash column chromatography (R_f= 0.25 in 40% ethyl acetate-Hexane) afforded 27.5 mg of 1,4-diol product with 30% yield over two steps. **IR:** 3396(br), 2927(m), 1509(s), 1427(m), 1221(m), 735(m). **^{1}HNMR (500 MHz, $CDCl_3$):** δ 7.57 – 7.52 (m, 2H), 7.44 – 7.34 (m, 3H), 7.20 (dd, *J* = 8.3, 5.4 Hz, 2H), 7.02 (t, *J* = 8.6 Hz, 2H), 3.60 (td, *J* = 9.9, 5.7 Hz, 1H), 2.96 (ddd, *J* = 13.6, 10.1, 3.6 Hz, 1H), 1.90 – 1.77 (m, 4H), 1.73 – 1.55 (m, 2H), 1.54 – 1.41 (m, 1H), 1.27 (d, *J* = 7.2 Hz, 1H), 0.36 (s, 6H). **^{13}CNMR (125 MHz, $CDCl_3$):** δ 162.89, 160.94, 138.47 (d, *J* = 3.3 Hz), 135.51, 134.66, 129.73, 129.62, 129.55, 128.14, 115.86, 115.69, 74.22, 65.25, 45.43, 40.50, 32.08, 28.08, -6.18, -6.30. **HRMS (ESI, *m/z*):** Calcd for $C_{20}H_{25}FO_2SiNa$ [M+Na] : 367.1500, found 367.1498

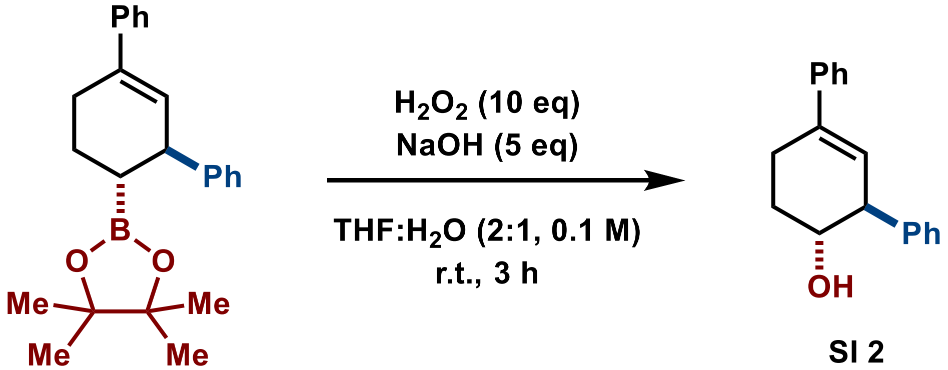

SI 2

(±)-3',4',5',6'-tetrahydro-[1,1':3',1''-terphenyl]-4'-ol (SI 2)
In a 2-dram vial, **26** (52.4 mg, 145 μmol, 1.00 eq) was dissolved in THF (1 mL). The solution was cooled to 0°C in an ice bath, and H_2O_2 (0.05 mL, 2 mmol, 10 eq, 30%wt in H_2O) and NaOH (1 mL, 2 mmol, 5 eq, 1 M in H_2O) were added before allowing the reaction to warm to room

temperature and stir for 3 hours. The reaction was quenched with H_2O (1 mL) and diluted with Et_2O (1 mL). The two phases were separated, and the aqueous phase was back extracted twice with Et_2O (2 × 2 mL). The combine organic phases were dried over $MgSO_4$, filtered, and concentrated *in-vacuo*. The crude product mixture was purified by MPLC with 0-15% EtOAc/Hexanes gradient as the eluent to provide 28.0 mg of the title compound as a white solid in 77% yield (average of two runs). Characterization data concurs with that found in the literature.[22] **^{1}H NMR (500 MHz, $CDCl_3$)**: δ 7.42 – 7.37 (m, 2H), 7.33 – 7.28 (m, 3H), 7.28 – 7.18 (m, 5H), 5.99 – 5.93 (m, 1H), 3.83 – 3.74 (m, 1H), 3.47 – 3.40 (m, 1H), 2.72 – 2.60 (m, 2H), 2.17 – 2.09 (m, 1H), 1.92 – 1.83 (m, 1H), 1.78 (s, 1H).

Sequential Functionalization of the C-Si and C-B Bonds and Gram-Scale Utility

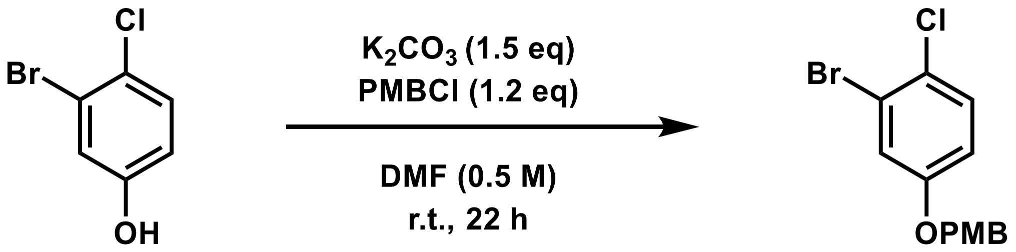

30

2-bromo-1-chloro-4-((4-methoxybenzyl)oxy)benzene (30)

This procedure was adapted from the literature.[23] In a nitrogen-filled glove box, to a flame-dried 100 mL round bottom flask with a magnetic stir bar, was added 3-bromo-4-chlorophenol (4.00 g, 19.3 mmol, 1.00 eq) and K_2CO_3 (4.00 g, 28.9 mmol, 1.50 eq). The flask was sealed with a rubber septum and removed from the glove box to be placed under a positive pressure of N_2. Anhydrous DMF (40 mL) was added to flask. 4-methoxybenzylchloride (3.2 mL, 24 mmol, 1.2 eq) was added dropwise and the reaction was stirred at room temperature for 22 hours. The reaction was then diluted with Et_2O (200 mL) and washed twice with aqueous LiCl (2 × 40 mL, 10% by volume). The organic phase was then dried with $MgSO_4$, filtered, and concentrated *in-vacuo*. The crude product was recrystallized in ethanol to produce 5.37 g of the title compound as a crystalline-white solid in 85% yield (average of two yields). **IR (neat):** 3093 (w), 3006 (w), 2952 (w), 2932 (w), 2835 (w), 1879 (w), 1610 (w), 1584 (m), 1511 (m), 1462 (m), 1375 (m), 1284 (m), 1224 (s), 1173 (m), 1119 (m), 1009 (s), 863 (m), 807 (s), 657 (m), 558 (m) cm^{-1}. **^{1}H NMR (400 MHz, $CDCl_3$):** δ 7.36 – 7.29 (m, 3H), 7.24 (d, J = 2.9 Hz, 1H), 6.97 – 6.89 (m, 2H), 6.86 (dd, J = 8.9, 2.9 Hz, 1H), 4.95 (s, 2H), 3.82 (s, 3H) ppm. **^{13}C NMR: (126 MHz, $CDCl_3$):** δ 159.8, 157.9, 130.6, 129.5, 128.2, 126.2, 122.7, 120.0, 115.8, 114.4, 70.5, 55.5 ppm. **HRMS (APCI) m/z:** matched on [M - H]+; Calc for $C_{14}H_{11}O_2BrCl$: 324.9625; Found: 324.9626.

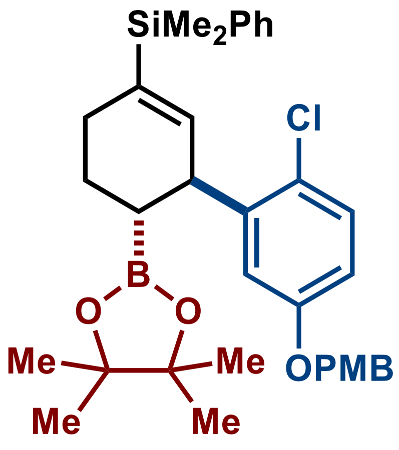

31

((±)-2'-chloro-5'-((4-methoxybenzyl)oxy)-6-(4,4,5,5-tetramethyl-1,3,2-dioxaborolan-2-yl)-1,4,5,6-tetrahydro-[1,1'-biphenyl]-3-yl)dimethyl(phenyl)silane (31)

The title compound was prepared by Procedure A above with **2** (1.07 mL, 5.00 mmol, 1.00 eq) and **30** (2.46 g, 7.50 mmol, 1.50 eq). The crude product was purified by MPLC with 0-8% EtOAc/Hexanes gradient as the eluent to provide 2.179 g of the title compound as a crystalline, colorless solid to a clear, colorless oil in 74% yield (average of two runs). **IR (neat):** 3069 (w), 2975 (w), 2931 (w), 2834 (w), 2361 (w), 2335 (w), 2161 (w), 2025 (w), 1974 (w), 1880 (w), 1614 (w), 1593 (w), 1571 (w), 1514 (m), 1463 (m), 1406 (w), 1376 (m), 1316 (m), 1301 (m), 1246 (s), 1217 (m), 1167 (m), 1142 (s), 1110 (m), 1036 (m), 1008 (m), 968 (w), 913 (w), 874 (w), 852 (w), 809 (s), 773 (m), 731 (m), 699 (m), 646 (m), 578 (w), 519 (w), 477 (m), 450 (m) cm^{-1}. **^{1}H NMR (500 MHz, $CDCl_3$):** δ 7.57 – 7.50 (m, 2H), 7.34 – 7.27 (m, 5H), 7.22 (d, J = 8.7 Hz, 1H), 6.94 – 6.87 (m, 2H), 6.82 (d, J = 3.0 Hz, 1H), 6.73 (dd, J = 8.7, 3.0 Hz, 1H), 6.04 (dt, J = 4.1, 2.1 Hz, 1H), 4.91 (s, 2H), 4.06 – 4.00 (m, 1H), 3.81 (s, 3H), 2.14 – 2.04 (m, 2H), 1.70 – 1.52 (m, 2H), 1.41 – 1.34 (m, 1H), 1.16 (s, 12H), 0.35 (s, 3H), 0.33 (s, 3 H) ppm. **^{13}C NMR (126 MHz, $CDCl_3$):** δ 159.6, 157.2, 144.5, 139.4, 138.8, 138.4, 134.1, 130.1, 129.4, 129.0, 128.9, 127.9, 125.5, 116.6, 114.2, 114.0, 83.2, 70.0, 55.5, 40.5, 26.5, 24.8, 24.7, 21.5, -3.4, -3.5 ppm (the carbon directly bonded to boron was not seen due to quadrupole relaxation). **HRMS (ESI) m/z:** matched on [M + Na]+; Calc for $C_{34}H_{42}O_4BClNaSi$: 611.2526; Found: 611.2532.

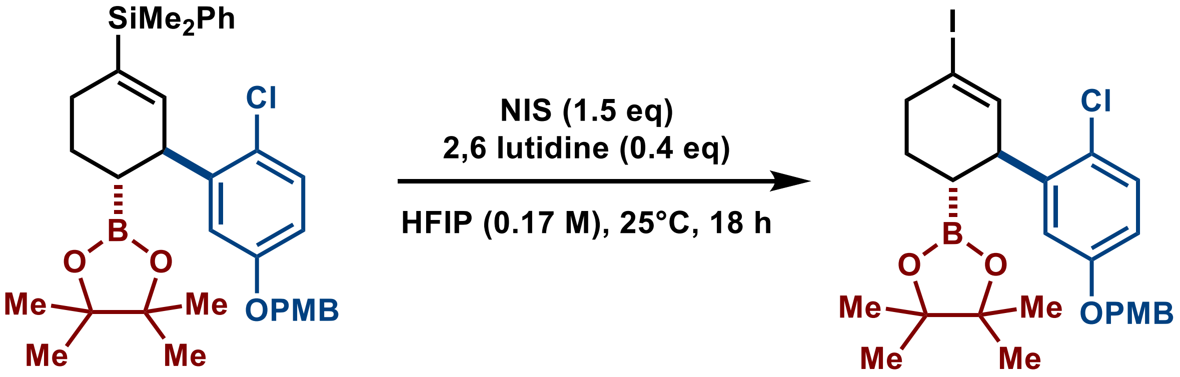

SI 3

2-((±)-2'-chloro-5-iodo-5'-((4-methoxybenzyl)oxy)-1,2,3,4-tetrahydro-[1,1'-biphenyl]-2-yl)-4,4,5,5-tetramethyl-1,3,2-dioxaborolane (SI 3)

This procedure was adapted from the literature.[18] To a flame-dried round bottom flask with a magnetic stir bar was added **31** (2.039 g, 3.461 mmol, 1.000 eq), 1,1,1,3,3,3-Hexafluoro-2-propanol (20 mL), and 2,6-dimethylpyridine (0.16 mL, 1.4 mmol, 0.40 eq). The solution was then cooled to 0°C, and N-iodosuccinimide (1.17 g, 5.19 mmol, 1.50 eq) was added in one portion. The reaction flask was purged with N_2 and allowed to warm to room temperature while stirring for 18 hours. After 18 hours, the reaction mixture was diluted with DCM (20 mL), and

the organic phase was washed with saturated sodium thiosulfate (20 mL), then saturated sodium bicarbonate (20 mL), and finally brine (20 mL). The organic layer was dried with magnesium sulfate, filtered, and concentrated *in-vacuo.* The crude product was purified by MPLC with 0-8% EtOAc/Hexanes gradient as the eluent to provide 1.5877 g of the title compound as a clear, light-yellow oil in 79% yield (average of two runs). **IR (neat):** 3042 (w), 2976 (w), 2930 (w), 2835 (w), 1737 (w), 1594 (w), 1514 (m), 1464 (m), 1406 (w), 1372 (m), 1321 (m), 1247 (m), 1167 (m), 1140 (s), 1025 (m), 970 (m), 849 (m), 819 (m), 736 (m), 669 (m), 520 (w) cm^{-1}. **^{1}H NMR (500 MHz, $CDCl_3$):** δ 7.38 – 7.33 (m, 2H), 7.22 (d, J = 8.7 Hz, 1H), 6.95 – 6.90 (m, 2H), 6.84 (d, J = 3.0 Hz, 1H), 6.75 (dd, J = 8.7, 3.0 Hz, 1H), 6.31 – 6.27 (m, 1H), 4.98 (d, J = 11.2 Hz, 1H), 4.96 (d, J = 11.2 Hz, 1H), 4.07 – 4.01 (m, 1H), 3.82 (s, 3H), 2.65 – 2.47 (m, 2H), 1.75 – 1.64 (m, 2H), 1.48 – 1.40 (m, 1H), 1.18 (appt d, 12H) ppm. **^{13}C NMR (126 MHz, $CDCl_3$):** δ 159.7, 157.3, 142.6, 139.5, 130.3, 129.5, 128.8, 125.1, 116.9, 114.2, 113.9, 98.8, 83.5, 70.2, 55.5, 43.3, 39.1, 24.8, 24.7, 24.1 ppm (the carbon directly bonded to boron was not seen due to quadrupole relaxation). **HRMS (ESI) m/z:** matched on [M + Na]+; Calc for $C_{26}H_{31}O_4BClINa$: 603.0941; Found: 603.0945.

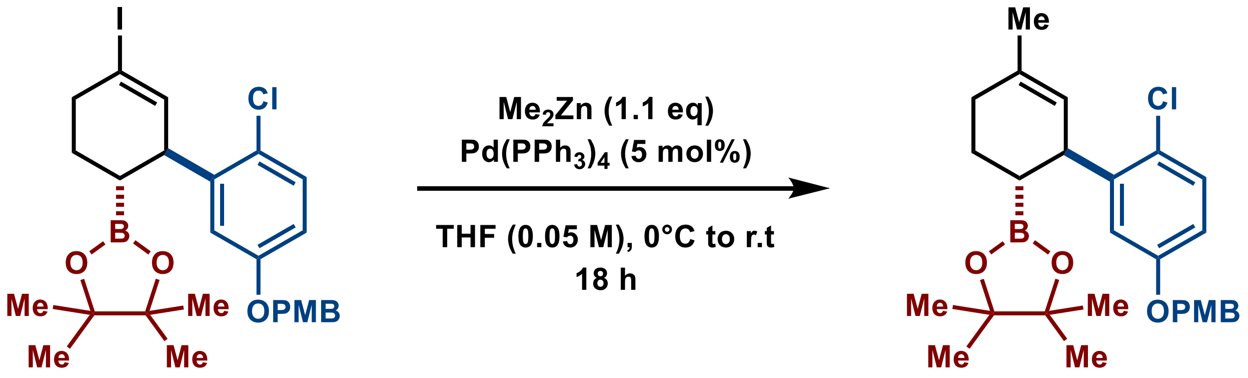

32

2-((±)-2'-chloro-5'-((4-methoxybenzyl)oxy)-5-methyl-1,2,3,4-tetrahydro-[1,1'-biphenyl]-2-yl)-4,4,5,5-tetramethyl-1,3,2-dioxaborolane (32)

To a flame-dried 25 mL round bottom flask with a magnetic stir bar was added activated $ZnCl_2$ (390 mg, 2.8 mmol, 1.1 eq) in a N_2-filled glovebox. To a separate flame-dried 200 mL round bottom flask with a magnetic stir bar was added $Pd(PPh_3)_4$ (150 mg, 0.13 mmol, 5.0 mol%) in a N_2-filled glovebox. Both round bottom flasks were capped with rubber septa, lined with Teflon tape, and removed from the glovebox. Anhydrous THF (6 mL) was added to the 25 mL round bottom flask, and it was cooled to 0°C in an ice bath. MeMgBr (1.9 mL, 2.2 eq, 3.0 molar in Et_2O) was added to the 25 mL round bottom flask and the solution was stirred for 15 minutes at 0°C. To the 200 mL round bottom flask was added anhydrous THF (60 mL) and **SI 3** (1.50 g, 2.58 mmol, 1.00 eq). The solids were allowed to settle in the 25 mL round bottom flask before transferring the supernatant dropwise to the 200 mL flask. The solid in the 25 mL flask was rinsed with another portion of anhydrous THF (6 mL) before letting the solid settle and transferring the supernatant dropwise to the 200 mL flask. The reaction was covered with aluminum foil and allowed to stir at room temperature overnight. After 18 hours, the reaction was quenched with water (60 mL), the two phases were separated, and the aqueous phase was back extracted with Et_2O (2 × 60 mL). The combined organic phases were washed with brine

(150 mL), dried over $MgSO_4$, filtered, and concentrated *in-vacuo*. The crude product purified by MPLC with 0-6% EtOAc/Hexanes gradient as the eluent to provide 1.0139 g of the title compound as a white solid in 84% yield (average of two runs). **IR (neat):** 3042 (w), 2976 (w), 2928 (w), 2872 (w), 2834 (w), 1613 (w), 1514 (m), 1463 (m), 1377 (m), 1304 (m), 1247 (m), 1141 (s), 1009 (m), 968 (w), 859 (m), 821 (m), 734 (m), 665 (m), 519 (w) cm^{-1}. **^{1}H NMR (500 MHz, $CDCl_3$):** δ 7.37 – 7.31 (m, 2H), 7.21 (d, J = 8.6 Hz, 1H), 6.93 – 6.88 (m, 2H), 6.85 (d, J = 3.1 Hz, 1H), 6.72 (dd, J = 8.7, 3.0 Hz, 1H), 5.35 – 5.30 (m, 1H), 4.95 (d, J = 11.3 Hz, 1H), 4.92 (d, J = 11.1 Hz, 1H), 3.98 – 3.93 (m, 1H), 3.81 (s, 3H), 1.96 (dq, J = 17.8, 9.2 Hz, 2H), 1.71 (s, 3H), 1.70 – 1.63 (m, 2H), 1.35 – 1.29 (m, 1H), 1.18 (s, 6H), 1.17 (s, 6H) ppm. **^{13}C NMR (126 MHz, $CDCl_3$):** δ 159.6, 157.2, 145.2, 135.7, 129.9, 129.5, 129.0, 125.5, 123.8, 117.1, 114.1, 113.1, 83.2, 70.1, 55.4, 39.4, 29.8, 24.8, 24.7, 24.1, 21.7 ppm (the carbon directly bonded to boron was not seen due to quadrupole relaxation). **HRMS (APCI) m/z:** matched on [M + H]+; Calc for $C_{27}H_{35}O_4BCl$: 469.2311; Found: 469.2307.

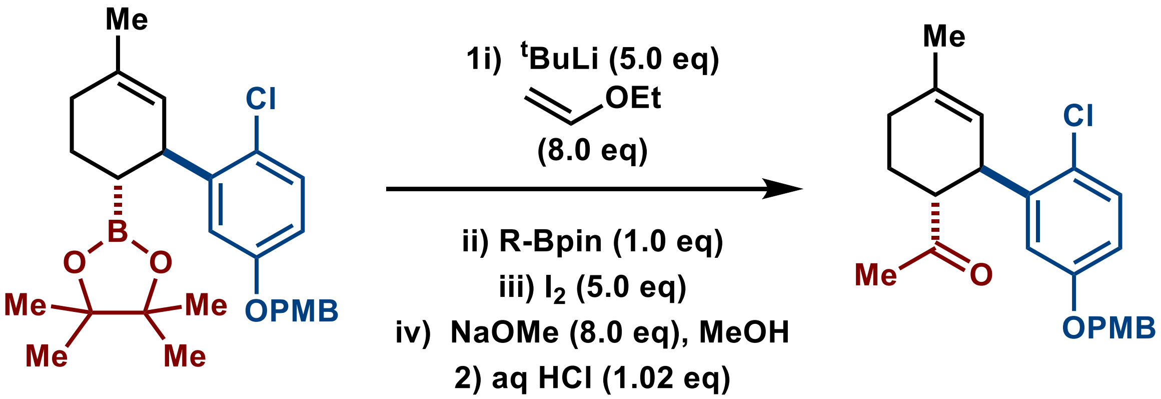

33

1-((±)-2'-chloro-5'-((4-methoxybenzyl)oxy)-5-methyl-1,2,3,4-tetrahydro-[1,1'-biphenyl]-2-yl)ethan-1-one (33)

This procedure was adapted from the literature.[24] To a flame dried 100 mL round bottom flask with a magnetic stir bar was added ethoxyethene (0.83 mL, 8.7 mmol, 8.0 eq) and anhydrous THF (14 mL). The flask was then cooled to -78°C, and ^{t}BuLi (3.85 mL, 5.46 mmol, 5.00 eq, 1.42 M in pentane) was added dropwise. The solution was stirred at -78°C for 1 hour. The reaction was then allowed to warm to 0°C and stir for an additional 1 hour. The flask was then cooled back to -78°C, and **32** (512 mg, 1.09 mmol, 1.00 eq) was added dropwise as a solution in anhydrous THF (5.7 mL). The resulting solution was stirred at -78°C for 1 hour, and then warmed to room temperature and stirred for 1 hour. The solution was then cooled to -78°C and I_2 (1.39 g, 5.46 mmol, 5.00 eq) was added as a solution in anhydrous THF (11 mL). The reaction was stirred at -78°C for 1 hour, and then warmed to room temperature and stirred for 1 hour. A solution of anhydrous NaOMe (472 mg, 8.74 mmol, 8.00 eq) in anhydrous MeOH (14 mL) was then added to the reaction at room temperature, and the resulting solution was stirred overnight. After 18 hours, the reaction was quenched with saturated aqueous $Na_2S_2O_3$ (50 mL). The two phases were separated, and the aqueous phase was back extracted twice with Et_2O (2 × 50 mL). The combined organic phases were dried over $MgSO_4$, filtered, and concentrated *in-vacuo*. The crude product was redissolved in anhydrous THF (10 mL) and HCl, (1.11 mL, 1.11 mmol, 1.02 eq, 1M solution in H_2O). The reaction was stirred at room temperature for 30 minutes, and then quenched with H_2O (10 mL). The two phases were separated, and the

aqueous phase was back extracted twice with Et_2O (2 × 10 mL). The combine organic phases were dried over $MgSO_4$, filtered, and concentrated *in-vacuo*. The crude product was purified by MPLC with 0-10% gradient as the eluent to provide 365.1 mg of the title compound as a white solid in 88% yield (average of two runs). **IR (neat):** 3085 (w), 3036 (w), 3012 (w), 2967 (w), 2937 (w), 2896 (w), 2839 (w),1710 (m), 1601 (w), 1568 (w), 1514 (m), 1467 (m), 1251 (m), 1176 (m), 1014 (m), 865 (m), 829 (m), 806 (m), 720 (w), 666 (m), 534 (w), 474 (w), 435 (w) cm^{-1}. **^{1}H NMR (400 MHz, $CDCl_3$):** δ 7.36 – 7.31 (m, 2H), 7.24 (d, J = 8.7 Hz, 1H), 6.94 – 6.88 (m, 2H), 6.83 (d, J = 3.0 Hz, 1H), 6.76 (dd, J = 8.7, 3.0 Hz, 1H), 5.30 – 5.24 (m, 1H), 4.96 (d, J = 11.3 Hz, 1H), 4.93 (d, J = 11.1 Hz, 1H), 4.19 – 4.11 (m, 1H), 3.82 (s, 3H), 2.74 – 2.66 (m, 1H), 2.11 (s, 3H), 2.01 – 1.94 (m, 2H), 1.88 (dq, J = 12.9, 6.5 Hz, 1H), 1.78 – 1.69 (m, 4H) ppm. **^{13}C NMR (126 MHz, $CDCl_3$):** δ 210.4, 159.7, 157.5, 143.0, 135.5, 130.4, 129.5, 128.8, 125.4, 121.9, 117.4, 114.2, 113.7, 70.2, 55.5, 52.4, 39.5, 28.5, 28.1, 23.8, 22.6 ppm. **HRMS (ESI) m/z:** matched on [M + Na]+; Calc for $C_{23}H_{25}O_3ClNa$: 407.1384; Found: 407.1386.

Enantioselective Example

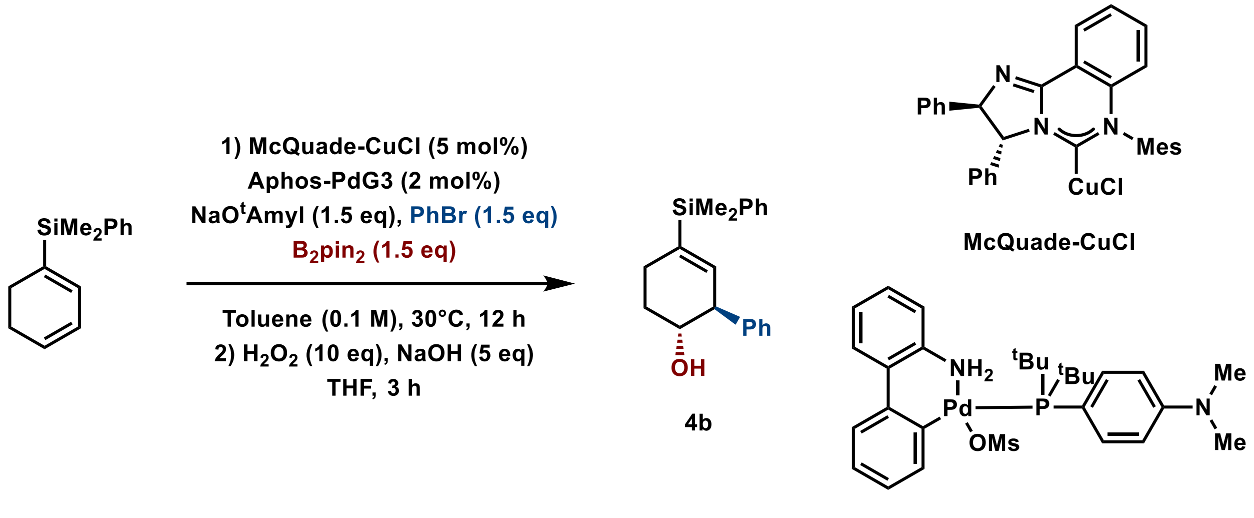

APhos-PdG3

(1S,2R)-5-(dimethyl(phenyl)silyl)-1,2,3,4-tetrahydro-[1,1'-biphenyl]-2-ol (4b)

In an N_2-filled glovebox, to a flame-dried 16 × 100 mm screw cap vial with a magnetic stir bar, was added APhos-PdG3 (5.1 mg, 8.0 μmol, 2.0 mol%), McQuade-CuCl (11 mg, 20. μmol, 5.0 mol%), B_2pin_2 (152 mg, 0.600 mmol, 1.50 eq), and NaO*t*Amyl (57.7 mg, 0.600 mmol, 1.50 eq) in that order. The 16 × 100 mm screw cap vial was sealed with a rubber septum, lined with Teflon tape, removed from the glove box, and placed under a positive pressure of N_2. To the screw cap vial was added toluene (3 mL), the **2** (86 μL, 0.40 mmol, 1.0 eq), and bromobenzene (63 μL, 0.60 mmol, 1.5 eq) before rinsing the sides of the vial with toluene (1 mL). The septum was then quickly replaced with a Teflon lined screw cap and the reaction was stirred at 30°C for 12 hours. After 12 hours, the reaction was quenched with aq 1M HCl (4 mL), the two phases were separated, and the aqueous phase was back extracted with Et_2O (2 × 4 mL). The combined organic phases were dried with $MgSO_4$, filtered, and concentrated *in-vacuo*. The crude product was redissolved in THF (2 mL). The solution was cooled to 0°C in an ice bath. H_2O_2 (0.10 mL, 4.0 mmol, 10 eq, 30%wt in H_2O) and NaOH (2 mL, 2 mmol, 5 eq, 1 M in H_2O) were added before allowing the reaction to warm to room temperature and stirred for 3 hours. The reaction

was quenched with H_2O (2 mL) and diluted with Et_2O (2 mL). The two phases were separated, and the aqueous phase was back extracted twice with Et_2O (2 × 2 mL). The combine organic phases were dried over $MgSO_4$, filtered, and concentrated *in-vacuo*. The crude product mixture was purified by MPLC with 0-10% EtOAc/Hexanes gradient as the eluent to provide 65 mg of the title compound as a white solid in 53% yield. The Enantiomeric mixture was then analyzed by HPLC.

Screening for the Arylboration of Silyl-Enol-Ether Diene

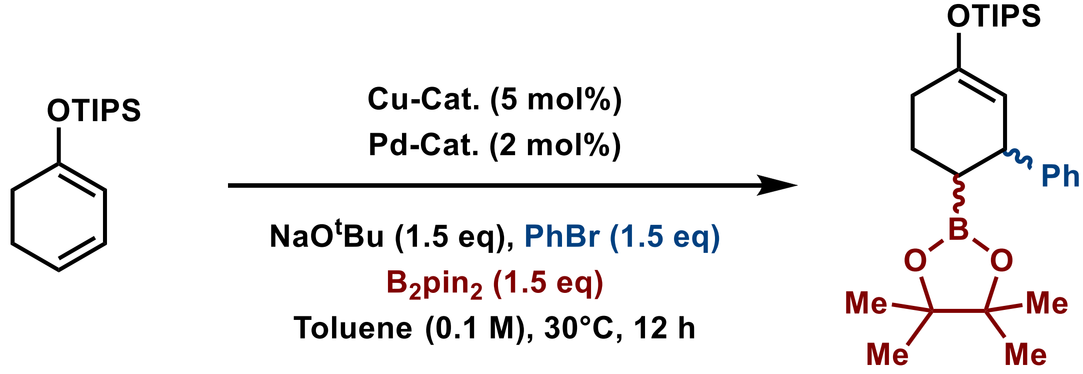

The following reactions were performed using Procedure A above with bromobenzene (0.60 mmol, 1.5 eq), no oxidation of the boronic ester, and the given copper (I) catalysts (0.02 mmol, 5 mol%) and palladium (0) precatalysts (0.008 mmol, 2 mol%) and . The yields of these runs were determined by GC with dodecane as the internal standard corrected by a burn factor. The best examples from the screen where also analyzed by ^1H NMR with nitromethane as the internal standard:

Entry	Cu Cat	Pd G3 PreCat	GC Yield	d.r.
1	SIMes-CuCl	APhos-PdG3	<2%	n.d.
2	SIMes-CuCl	XPhos-PdG3	8%	2:1
3	SIMes-CuCl	QPhos-PdG3	<2%	n.d.
4	SIPr-CuCl	APhos-PdG3	<2%	n.d.
5	SIPr-CuCl	XPhos-PdG3	8%	4:1
6	SIPr-CuCl	QPhos-PdG3	<2%	n.d.
7	Mes-Pyr-CuCl	APhos-PdG3	n.d.	4:1
8	Mes-Pyr-CuCl	XPhos-PdG3	17%[a]	6.5:1.7:1
9	Mes-Pyr-CuCl	QPhos-PdG3	2%	5:1

GC yields and d.r. were determined using dodecane as an internal standard. These yields were uncorrected. a) Entry 8 was also analyzed by ^1H NMR with nitromethane as the internal standard to determine a more accurate yield of 13%.

Limitations of the Method

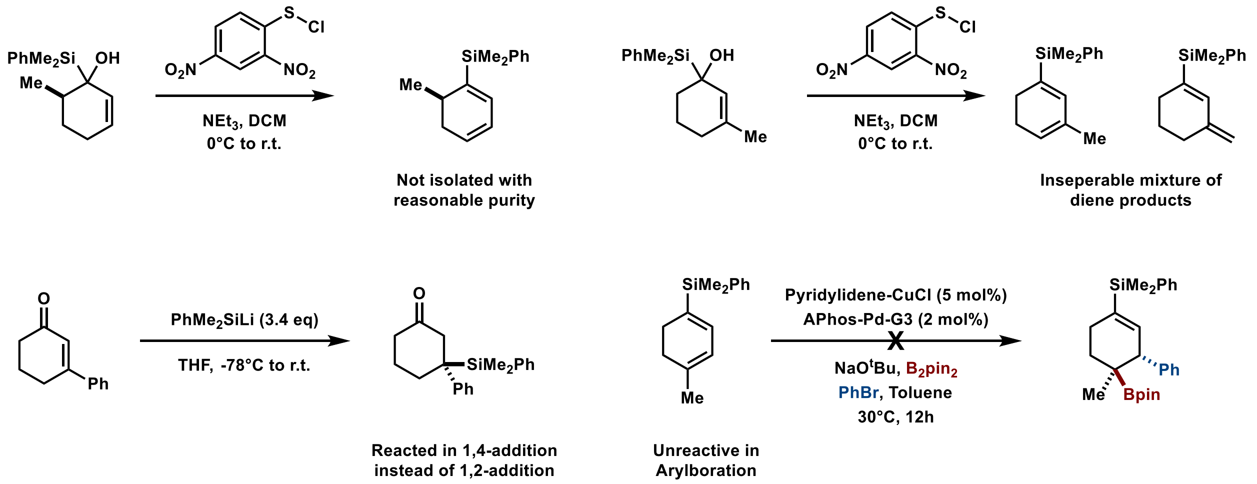

Some limitation of the method included the synthesis and employment of other silyl-dienes. Substitution of the silyl-diene with a methyl group in the C-6 position underwent silyl-lithium addition smoothly, but the elimination product was not able to be isolated with reasonable purity due to the highly non-polar nature of these dienes. Methyl substitution on C-3 lead to a mixture of two diene isomers that were not separable by column chromatography. Exchanging the methyl group with a phenyl group on C-3 because it lacks a proton for deprotonation did not generate the desired 1,2-addition product. We hypothesize that 1,4-addition was the primary side product. Finally, we successfully synthesized the silyl-diene with methyl substitution on C4, but this substrate was unreactive in the optimized arylboration conditions. We hypothesize that this is due to steric repulsion between the boryl-copper intermediate with the reactive alkene.

Citations

[1] Y. Huang, M. K. Brown, *Angew Chem Int Ed.,* 2019, **58**, 6048.
[2] N. C. Bruno, M. T. Tudge, S. L. Buchwald, *Chem Sci,* 2013, **4**, 916.
[3] X. Li, M. Jiang, X. L. Zhu, X. Y. Song, Q. R. Deng, J. Lv, D. S. Yang, *Org Chem Front,* 2022, **9**, 386.
[4] S. Verdonck, S. Y. Pu, F. J. Sorrell, J. M. Elkins, M. Froeyen, L. J. Gao, L. I. Prugar, D. E. Dorosky, J. M. Brannan, R. Barouch-Bentov, S. Knapp, J. M. Dye, P. Herdewijn, S. Einav, S. De Jonghe, *J Med Chem,* 2019, **62**, 5810.
[5] S. Botov, E. Stamellou, S. Romanski, M. Guttentag, R. Alberto, J. M. Neudorfl, B. Yard, H. G. Schmalz, *Organometallics,* 2013, **32**, 3587.
[6] S. Diez-Gonzalez, E. C. Escudero-Adan, J. Benet-Buchholz, E. D. Stevens, A. M. Z. Slawin, S. P. Nolan, *Dalton T,* 2010, **39**, 7595-7606.
[7] K. M. Logan, M. K. Brown, *Angew Chem Int Ed.,* 2017, **56**, 851.
[8] B. D. Chong, Y. I. Ji, S. S. Oh, J. D. Yang, W. Baik, S. Koo, *J Org Chem,* 1997, **62**, 9323.
[9] J. W. Hall, D. Bouchet, M. F. Mahon, M. K. Whittlesey, C. S. J. Cazin, *Organometallics,* 2021, **40**, 1252.
[10] J. X. Xie, X. Liu, N. Zhang, S. Y. Choi, G. B. Dong, *J Am Chem Soc,* 2021, **143**, 19311.
[11] K. Semba, T. Fujihara, T. H. Xu, J. Terao, Y. Tsuji, *Adv Synth Catal,* 2012, **354**, 1542.
[12] D. Ni, M. K. Brown, *ACS Catal,* 2021, **11**, 1858.
[13] I. Fleming, R. S. Robers, S. C. Smith, *J. Chem. Soc., Perkin Trans. 1,* **1198**, 1209.
[14] H. J. Reich, S. Wollowitz, *J Am Chem Soc,* 1982, **104**, 7051.
[15] E. Speckmeier, T. C. Maier, *J Am Chem Soc,* 2022, **144**, 9997.
[16] M. Odachowski, A. Bonet, S. Essafi, P. Conti-Ramsden, J. N. Harvey, D. Leonori, V. K. Aggarwal, *J Am Chem Soc,* 2016, **138**, 9521.
[17] X. S. Ma, S. B. Herzon, *J Org Chem,* 2016, **81**, 8673.
[18] E. A. Ilardi, C. E. Stivala, A. Zakarian, *Org Lett,* 2008, **10**, 1727.
[19] W. W. Zhang, S. L. Zhang, B. J. Li, *Angew Chem Int Ed.,* 2020, **59**, 6874.
[20] X. Du, H. Chen, Y. Liu, *Chemistry,* 2008, **14**, 9495.
[21] Soderquist, A. John. and Brown, C. Herbert. *J. Org. Chem.*, 1980, **45**, 3571-3578.
[22] S. R. Sardini, M. K. Brown, *J Am Chem Soc,* 2017, **139**, 9823.
[23] A. Ilangovan, K. Anandhan, M. P. Kaushik, *Tetrahedron Lett,* 2015, **56**, 1080.
[24] R. P. Sonawane, V. Jheengut, C. Rabalakos, R. Larouche-Gauthier, H. K. Scott, V. K. Aggarwal, *Angew Chem Int Ed Engl,* 2011, **50**, 3760.

HPLC Data and NMR Spectra

Data File C:\CHEM32\2\DATA\ARL\ARL-II-21300013.D
Sample Name: ARL-II-213-HPLC-A

```
=====================================================================
Acq. Operator   : SYSTEM                        Seq. Line :    1
Acq. Instrument : 1220 HPLC                      Location : Vial 81
Injection Date  : 11/6/2019 12:22:44 PM               Inj :    1
                                               Inj Volume : 10.000 µl
Acq. Method     : C:\CHEM32\2\METHODS\MLC_VARIABLE.M
Last changed    : 11/6/2019 12:10:57 PM by SYSTEM
Analysis Method : C:\CHEM32\2\METHODS\DEF_LC.M
Last changed    : 8/17/2020 3:31:15 PM by SYSTEM
                  (modified after loading)
Sample Info     : Lux 3u Cellulose-2, 95.0:5.0 HEX:IPA, 0.5 ml/min, 254 n
                  m

Additional Info : Peak(s) manually integrated
```

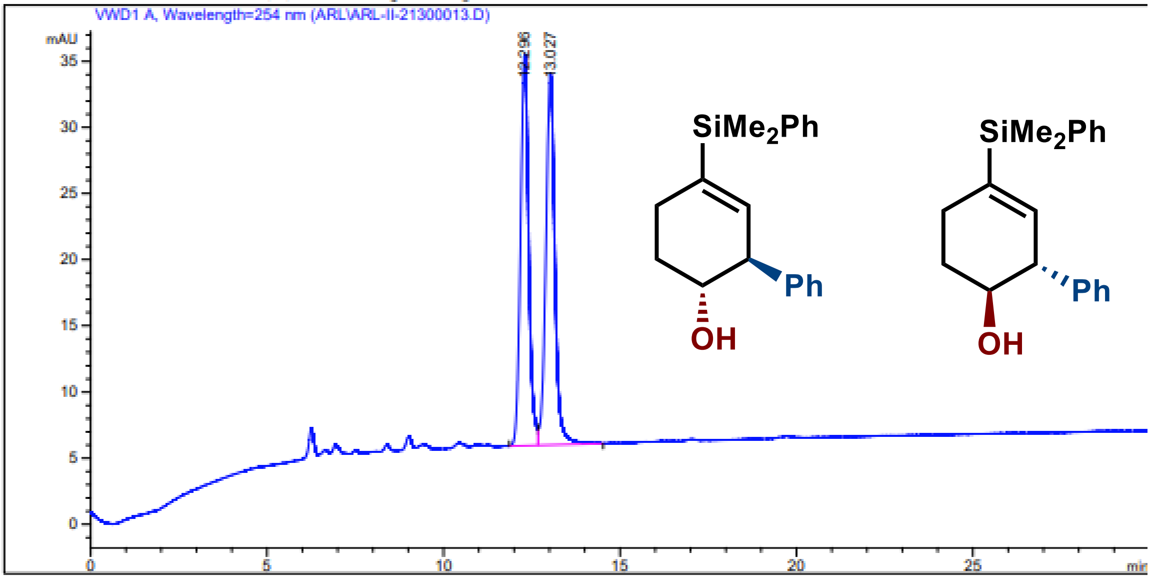


```
=====================================================================
                         Area Percent Report
=====================================================================

Sorted By             :      Signal
Multiplier:                  :       1.0000
Dilution:                    :       1.0000
Use Multiplier & Dilution Factor with ISTDs


Signal 1: VWD1 A, Wavelength=254 nm
```

Peak #	RetTime [min]	Type	Width [min]	Area [mAU*s]	Height [mAU]	Area %
1	12.296	BV	0.2459	476.03647	29.55855	48.8845
2	13.027	VB	0.2690	497.76160	28.06006	51.1155
Totals :				973.79807	57.61860	

Data File C:\CHEM32\2\DATA\ARL\ARL-II-213_F002.D
Sample Name: ARL-II-213-HPLC-F

===

Acq. Operator : SYSTEM Seq. Line : 1
Acq. Instrument : 1220 HPLC Location : Vial 81
Injection Date : 11/7/2019 12:18:01 PM Inj : 1
Inj Volume : 10.000 µl
Acq. Method : C:\CHEM32\2\METHODS\MLC_VARIABLE.M
Last changed : 11/6/2019 12:10:57 PM by SYSTEM
Analysis Method : C:\CHEM32\2\METHODS\DEF_LC.M
Last changed : 8/17/2020 3:31:15 PM by SYSTEM
(modified after loading)
Sample Info : Lux 3u Cellulose-2, 95.0:5.0 HEX:IPA, 0.5 ml/min, 254 nm

Additional Info : Peak(s) manually integrated

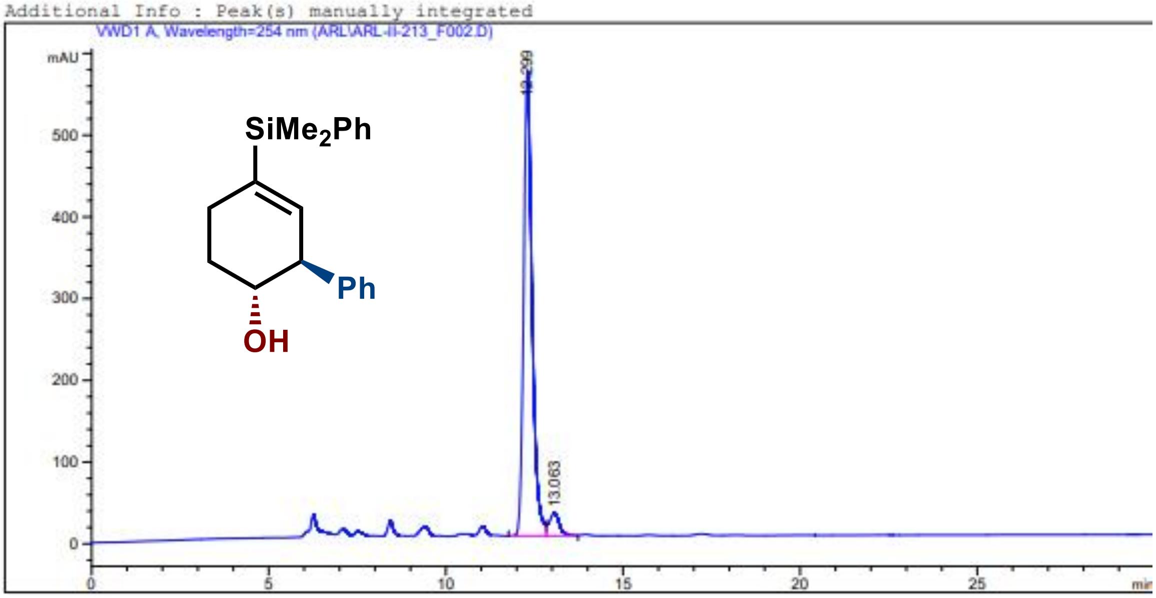

===
Area Percent Report
===

Sorted By : Signal
Multiplier: : 1.0000
Dilution: : 1.0000
Use Multiplier & Dilution Factor with ISTDs

Signal 1: VWD1 A, Wavelength=254 nm

Peak #	RetTime [min]	Type	Width [min]	Area [mAU*s]	Height [mAU]	Area %
1	12.299	BV	0.2464	9354.02832	567.06604	94.1810
2	13.063	VV	0.2946	577.93622	28.73856	5.8190

Totals : 9931.96454 595.80460

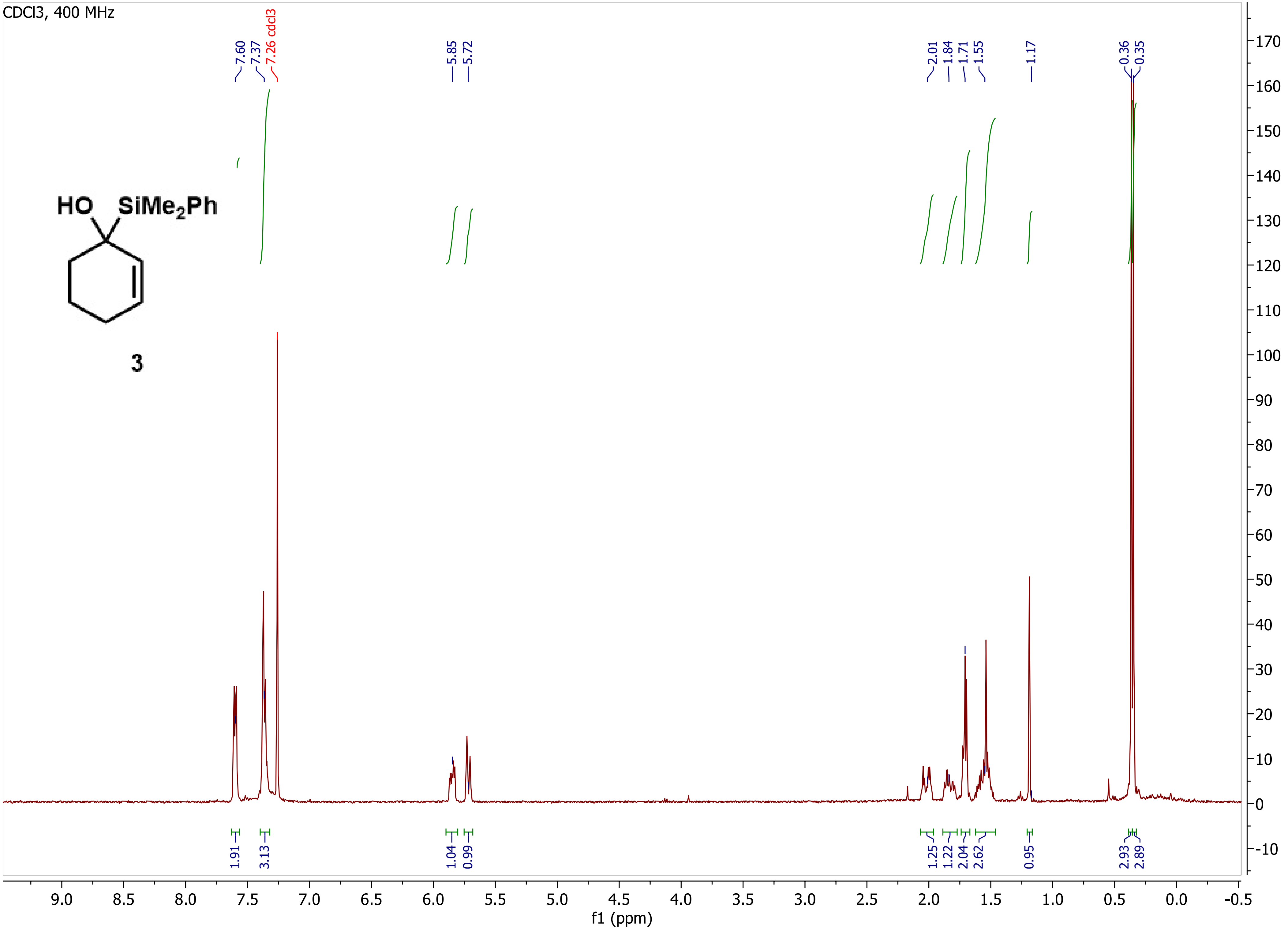

CDCl3, 400 MHz
7.60
7.37
7.26 cdcl3
5.85
5.72
2.01
1.84
1.71
1.55
1.17
0.36
0.35
HO
SiMe2Ph
3
1.91
3.13
1.04
0.99
1.25
1.22
2.04
2.62
0.95
2.93
2.89
f1 (ppm)

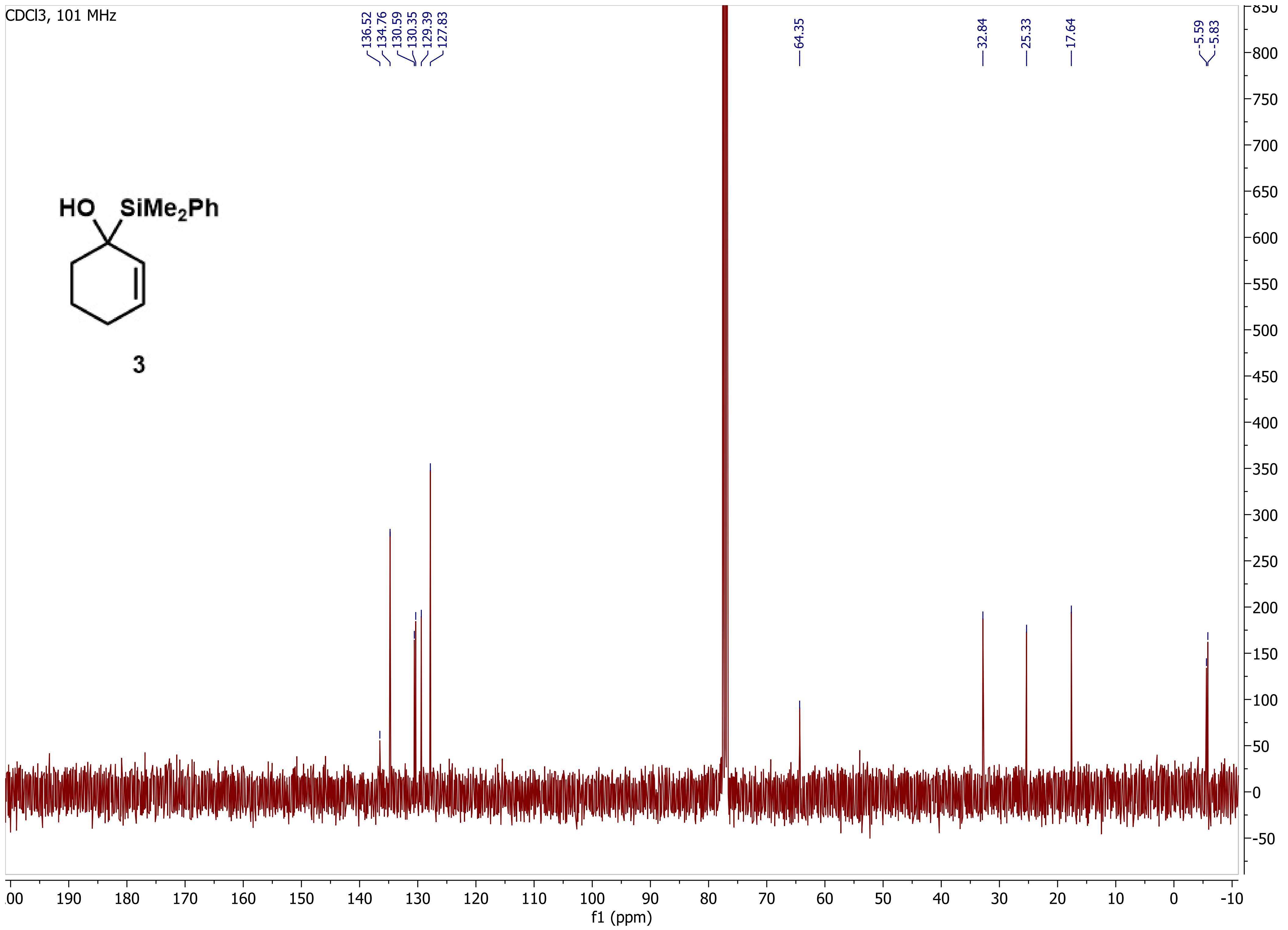

CDCl3, 101 MHz
136.52
134.76
130.59
130.35
129.39
127.83
64.35
32.84
25.33
17.64
-5.59
-5.83
HO
SiMe2Ph
3
f1 (ppm)

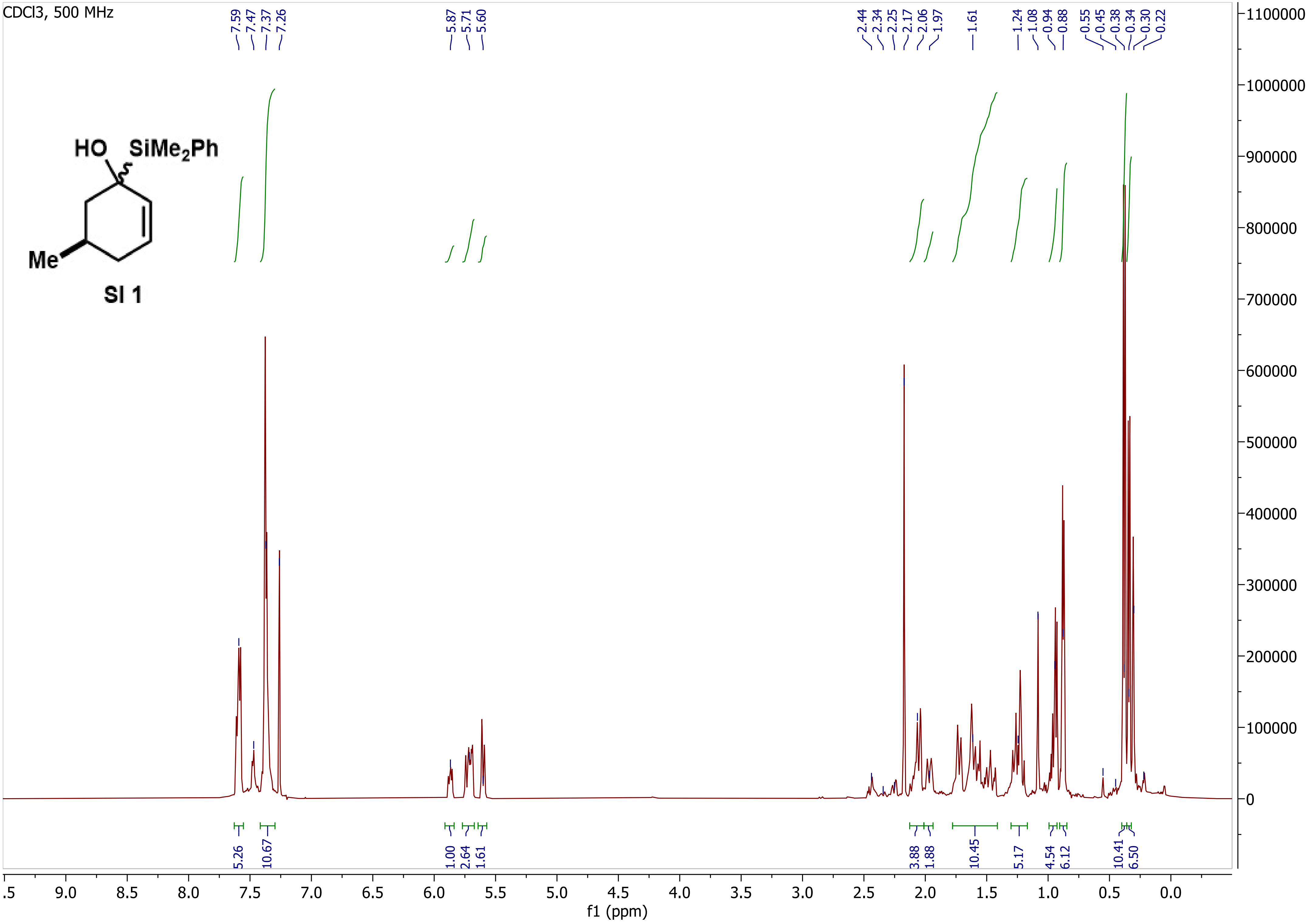

CDCl3, 500 MHz
HO SiMe2Ph
Me
SI 1
7.59 7.47 7.37 7.26
5.87 5.71 5.60
2.44 2.34 2.25 2.17 2.06 1.97
1.61
1.24 1.08 0.94 0.88
0.55 0.45 0.38 0.34 0.30 0.22
5.26 10.67
1.00 2.64 1.61
3.88 1.88
10.45
5.17
4.54 6.12
10.41 6.50
1100000
1000000
900000
800000
700000
600000
500000
400000
300000
200000
100000
0
.5 9.0 8.5 8.0 7.5 7.0 6.5 6.0 5.5 5.0 4.5 4.0 3.5 3.0 2.5 2.0 1.5 1.0 0.5 0.0
f1 (ppm)

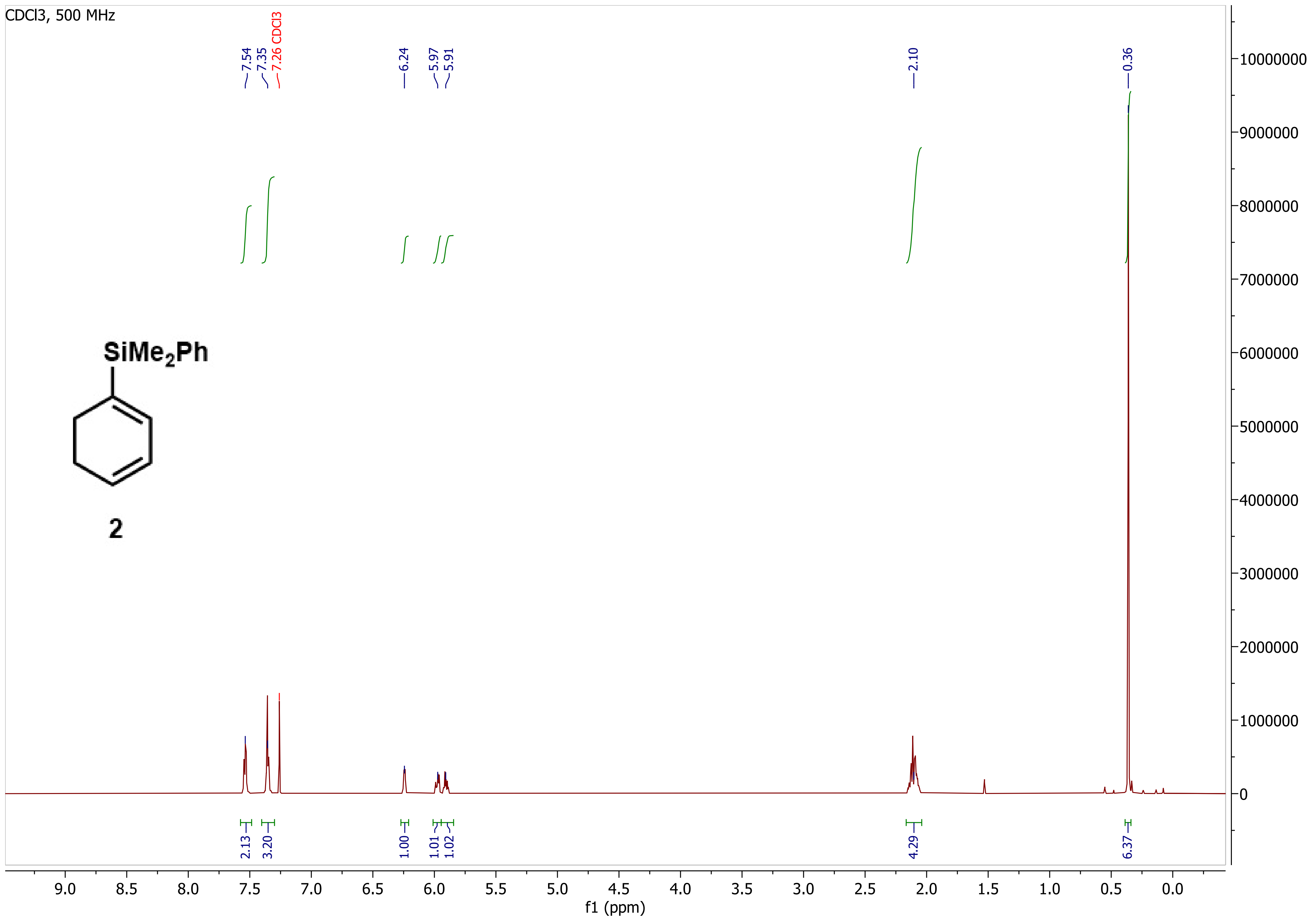
CDCl3, 500 MHz
7.54
7.35
7.26 CDCl3
6.24
5.97
5.91
2.10
0.36
SiMe2Ph
2
2.13
3.20
1.00
1.01
1.02
4.29
6.37
f1 (ppm)

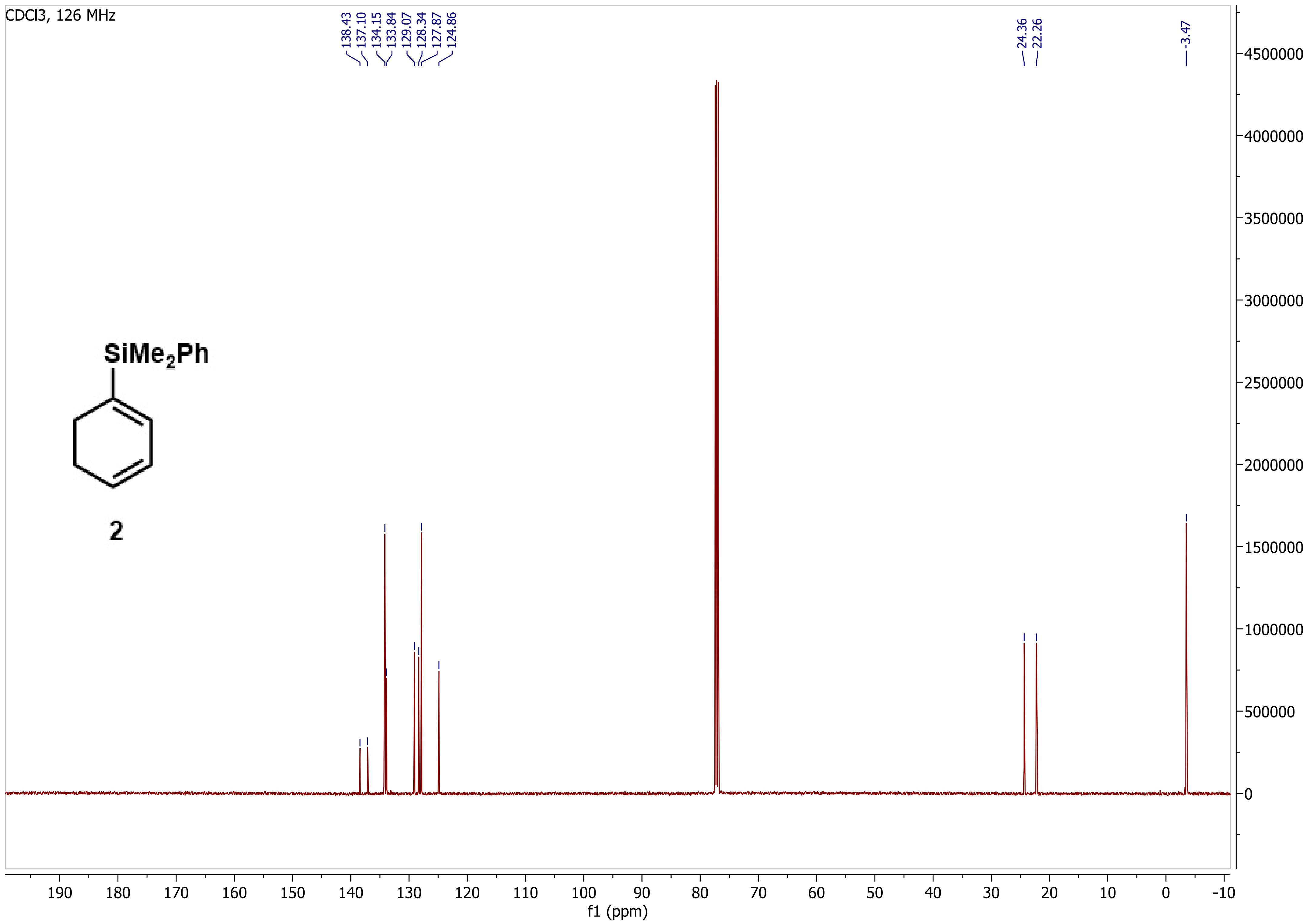
CDCl3, 126 MHz
138.43
137.10
134.15
133.84
129.07
128.34
127.87
124.86
24.36
22.26
-3.47
SiMe2Ph
2
4500000
4000000
3500000
3000000
2500000
2000000
1500000
1000000
500000
0
190
180
170
160
150
140
130
120
110
100
90
80
70
60
50
40
30
20
10
0
-10
f1 (ppm)

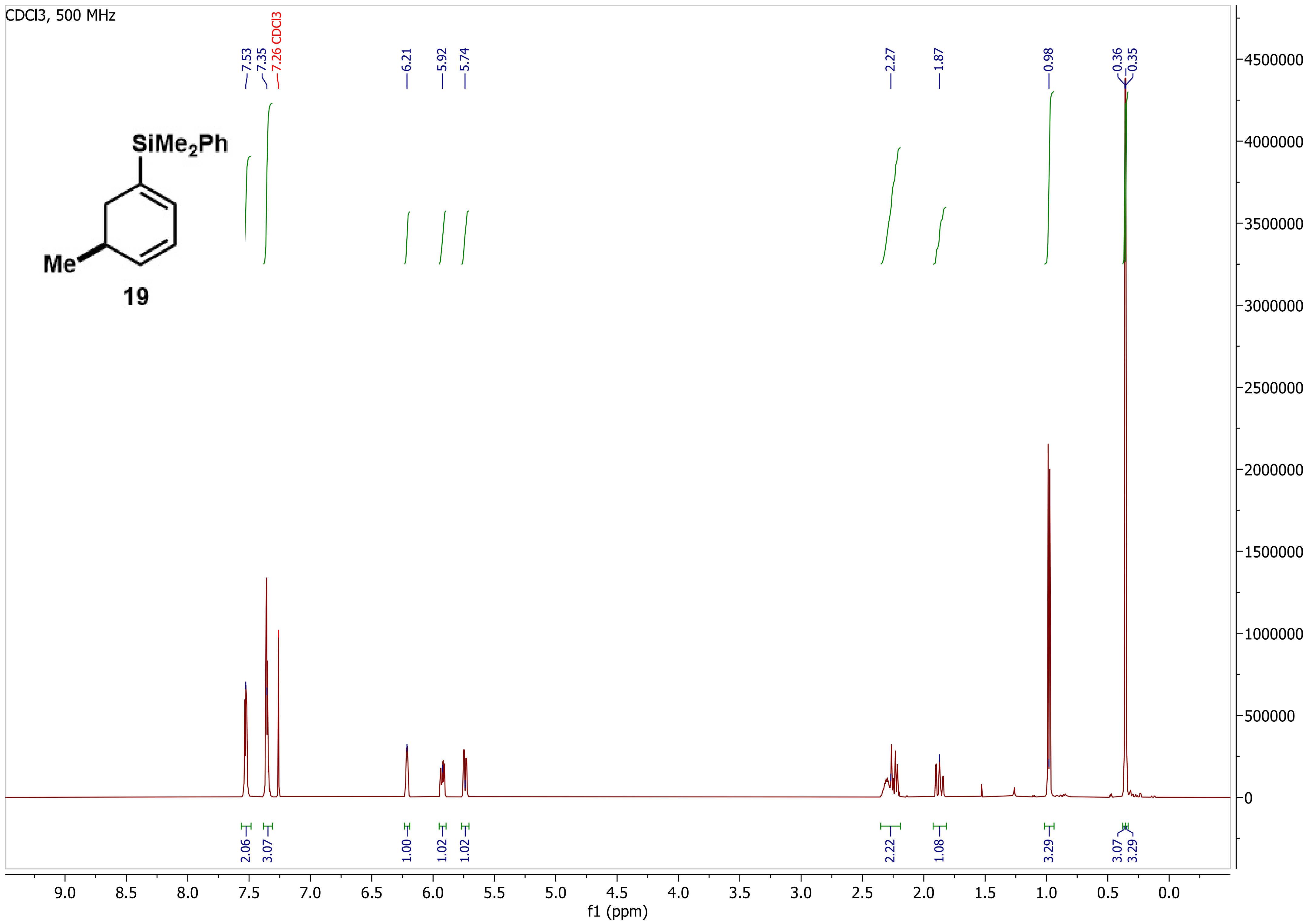

CDCl3, 500 MHz
SiMe2Ph
Me
19
7.53
7.35
7.26 CDCl3
6.21
5.92
5.74
2.27
1.87
0.98
0.36
0.35
2.06
3.07
1.00
1.02
1.02
2.22
1.08
3.29
3.07
3.29
f1 (ppm)

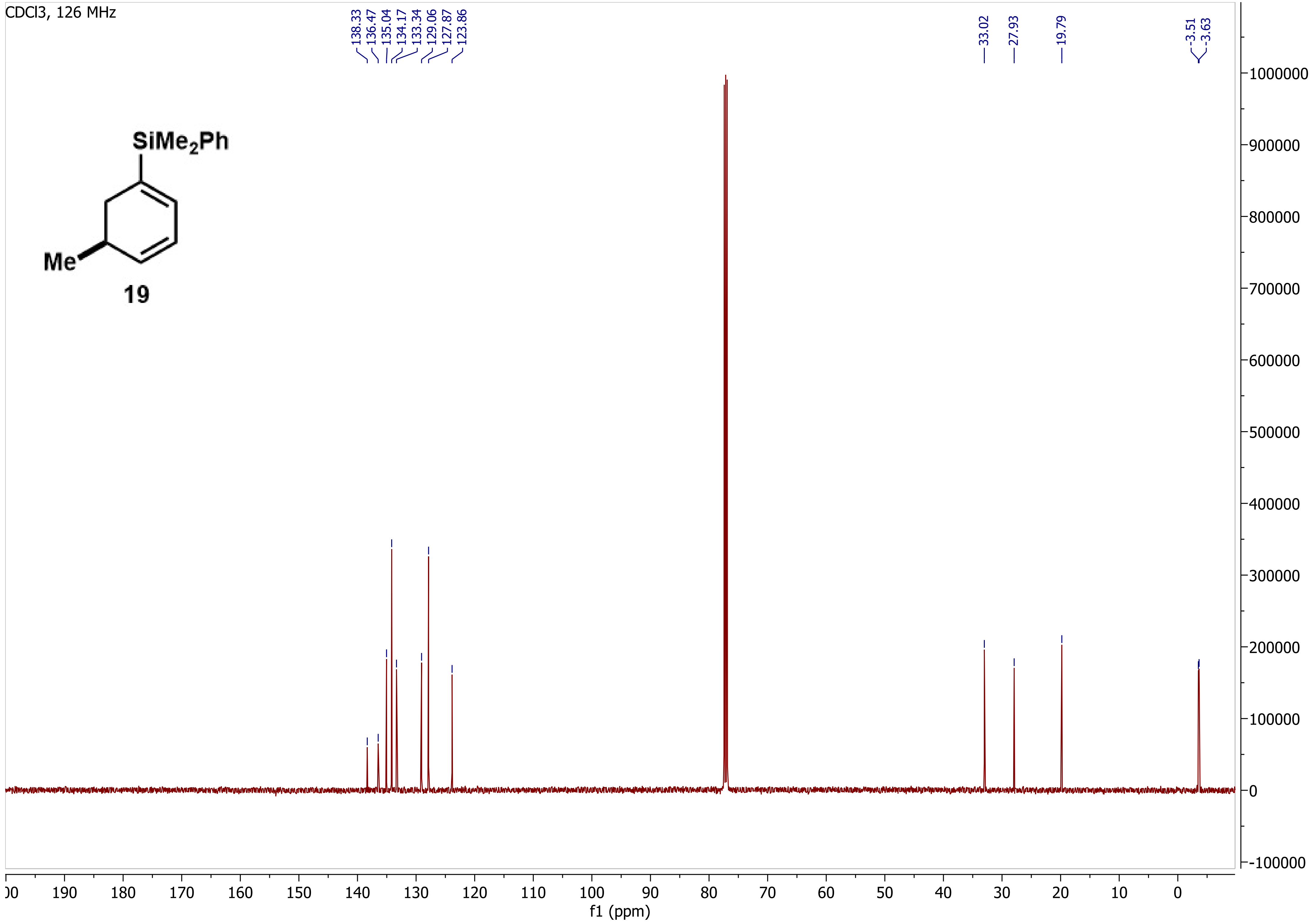
CDCl3, 126 MHz
138.33
136.47
135.04
134.17
133.34
129.06
127.87
123.86
33.02
27.93
19.79
-3.51
-3.63
SiMe2Ph
Me
19
f1 (ppm)

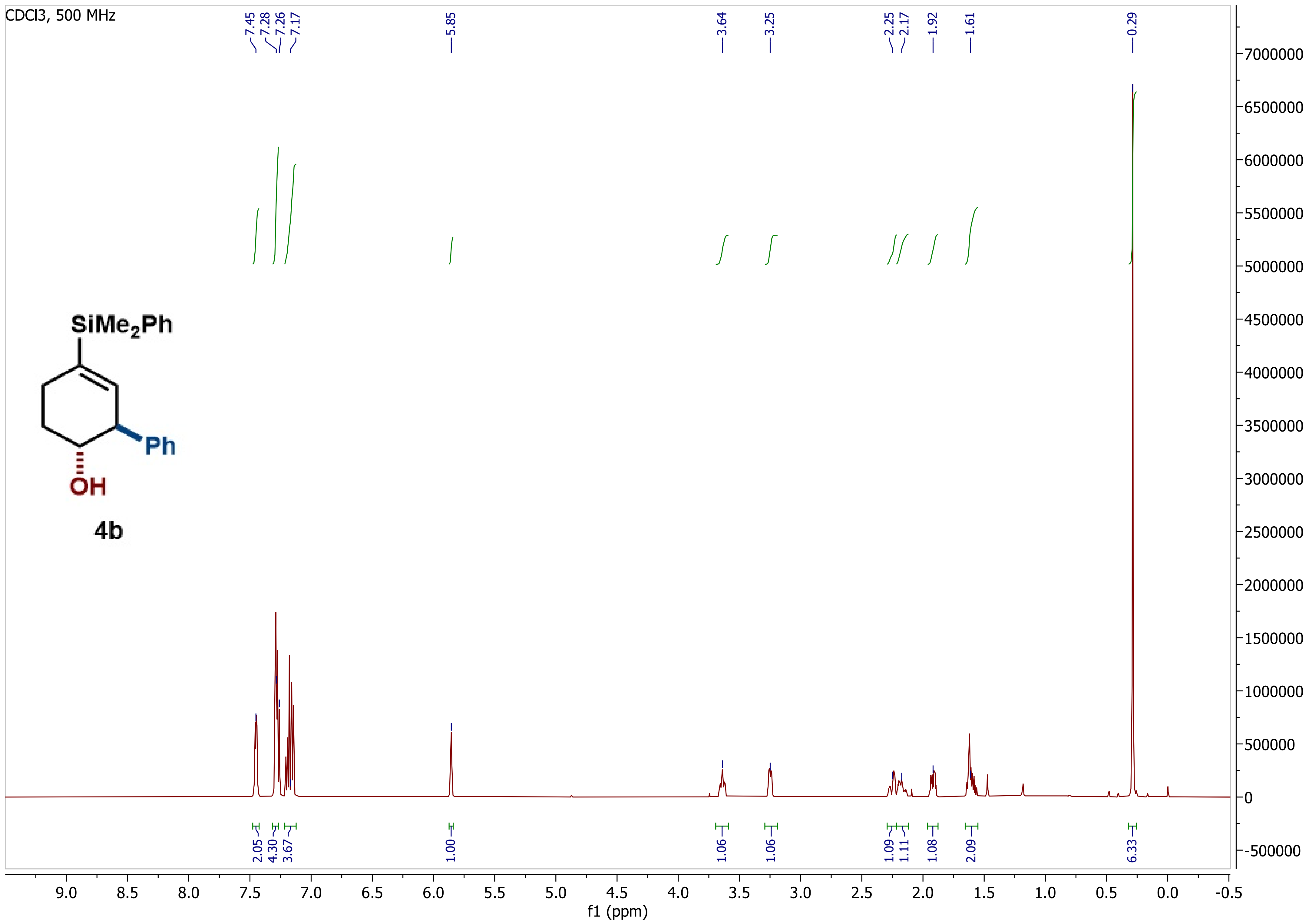

CDCl3, 500 MHz
7.45
7.28
7.26
7.17
5.85
3.64
3.25
2.25
2.17
1.92
1.61
0.29
SiMe2Ph
Ph
OH
4b
2.05
4.30
3.67
1.00
1.06
1.06
1.09
1.11
1.08
2.09
6.33
f1 (ppm)

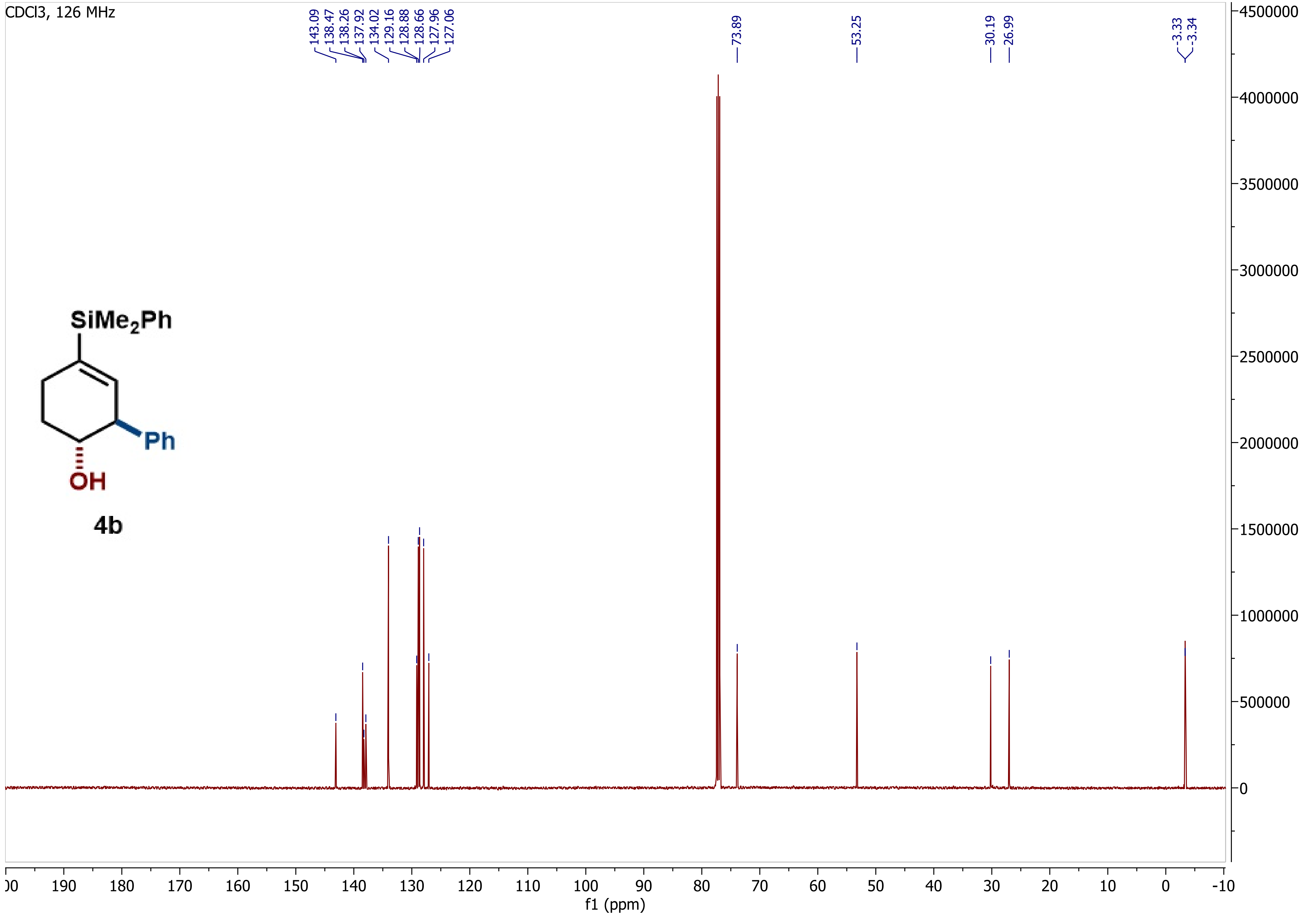
CDCl3, 126 MHz
143.09
138.47
138.26
137.92
134.02
129.16
128.88
128.66
127.96
127.06
73.89
53.25
30.19
26.99
-3.33
-3.34
SiMe2Ph
Ph
OH
4b
f1 (ppm)

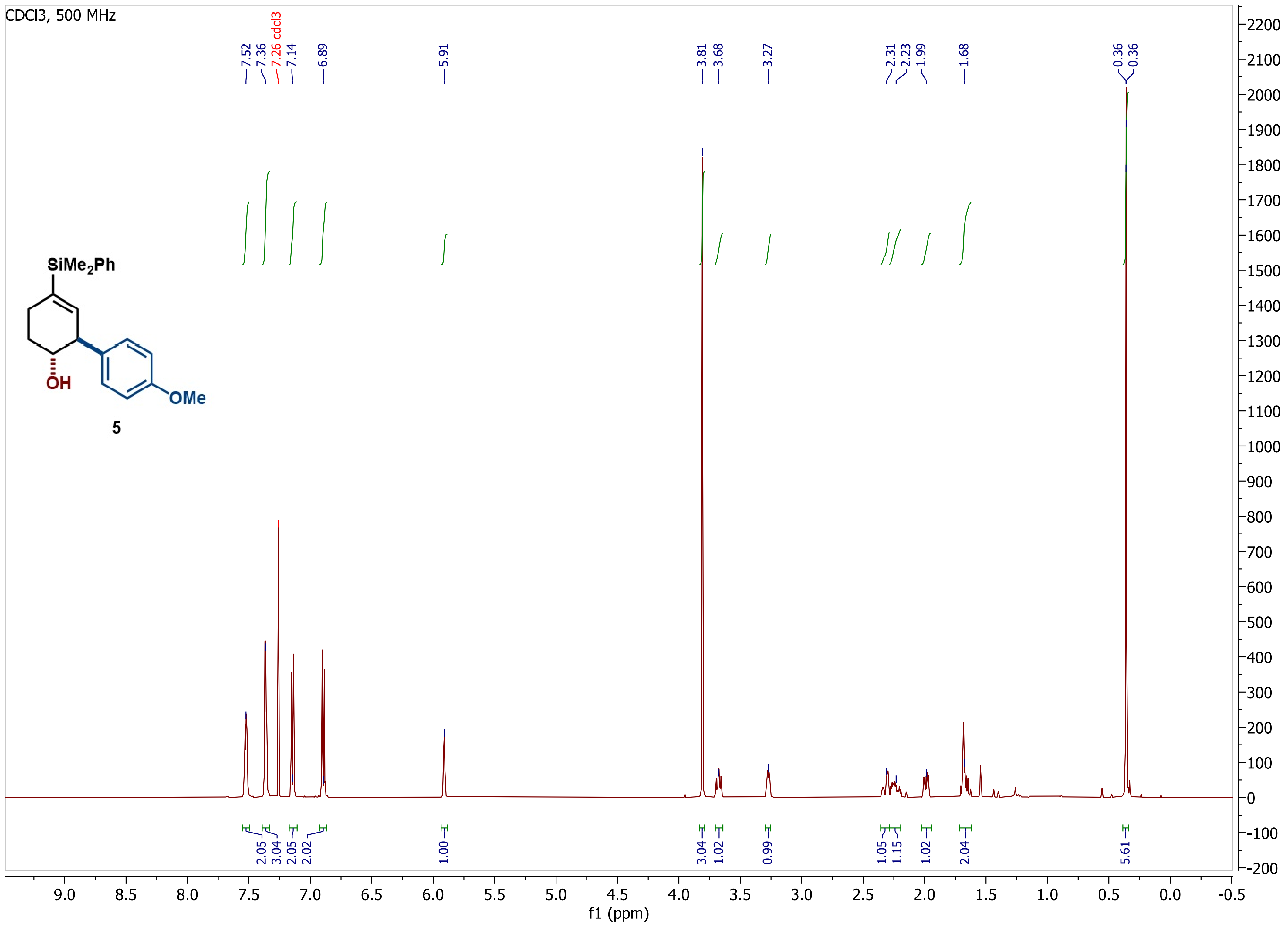
CDCl3, 500 MHz
7.52
7.36
7.26 cdcl3
7.14
6.89
5.91
3.81
3.68
3.27
2.31
2.23
1.99
1.68
0.36
0.36
SiMe2Ph
OH
OMe
5
2.05
3.04
2.05
2.02
1.00
3.04
1.02
0.99
1.05
1.15
1.02
2.04
5.61
f1 (ppm)

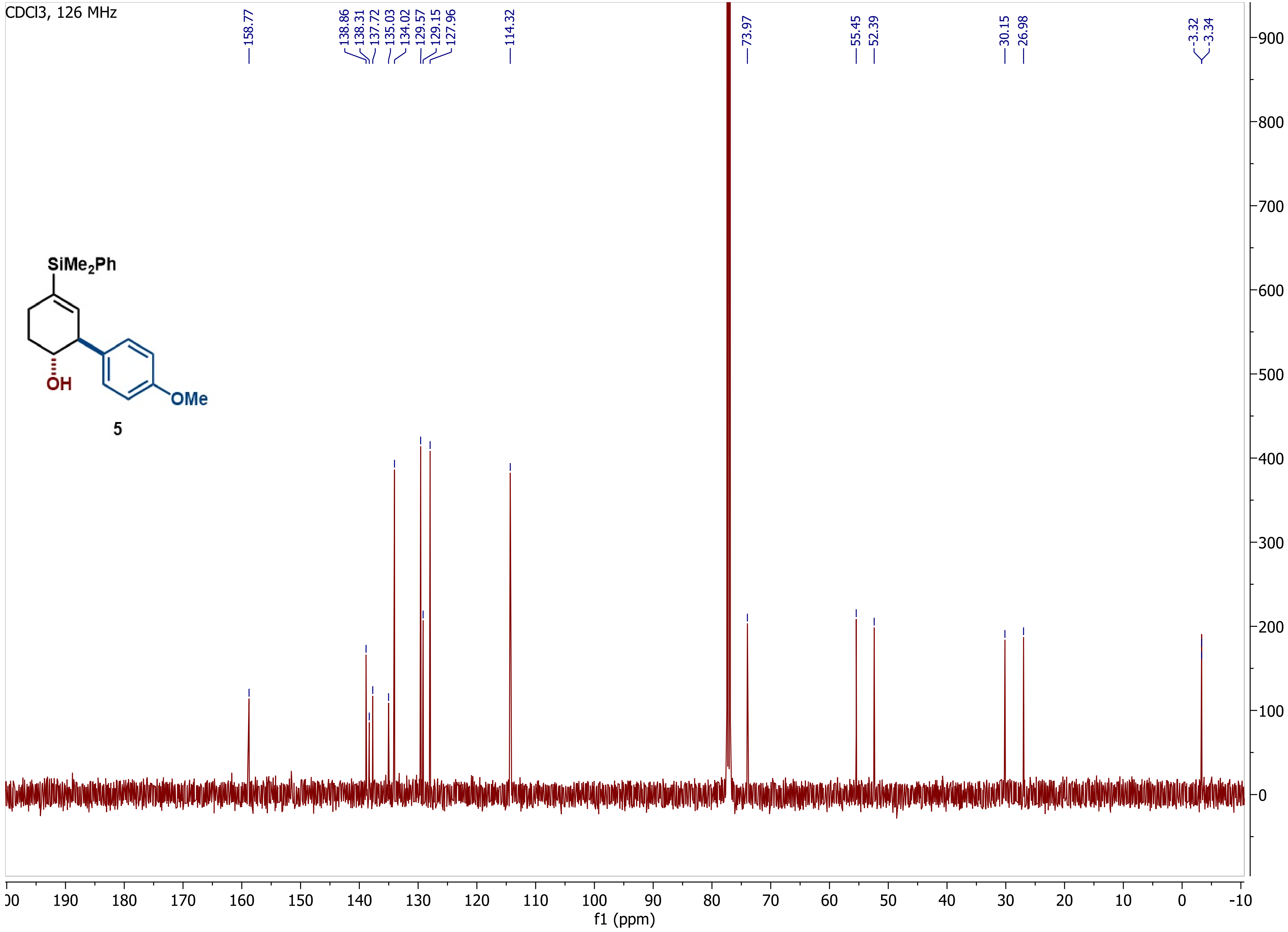

CDCl3, 126 MHz
158.77
138.86
138.31
137.72
135.03
134.02
129.57
129.15
127.96
114.32
73.97
55.45
52.39
30.15
26.98
-3.32
-3.34
SiMe2Ph
OH
OMe
5
f1 (ppm)

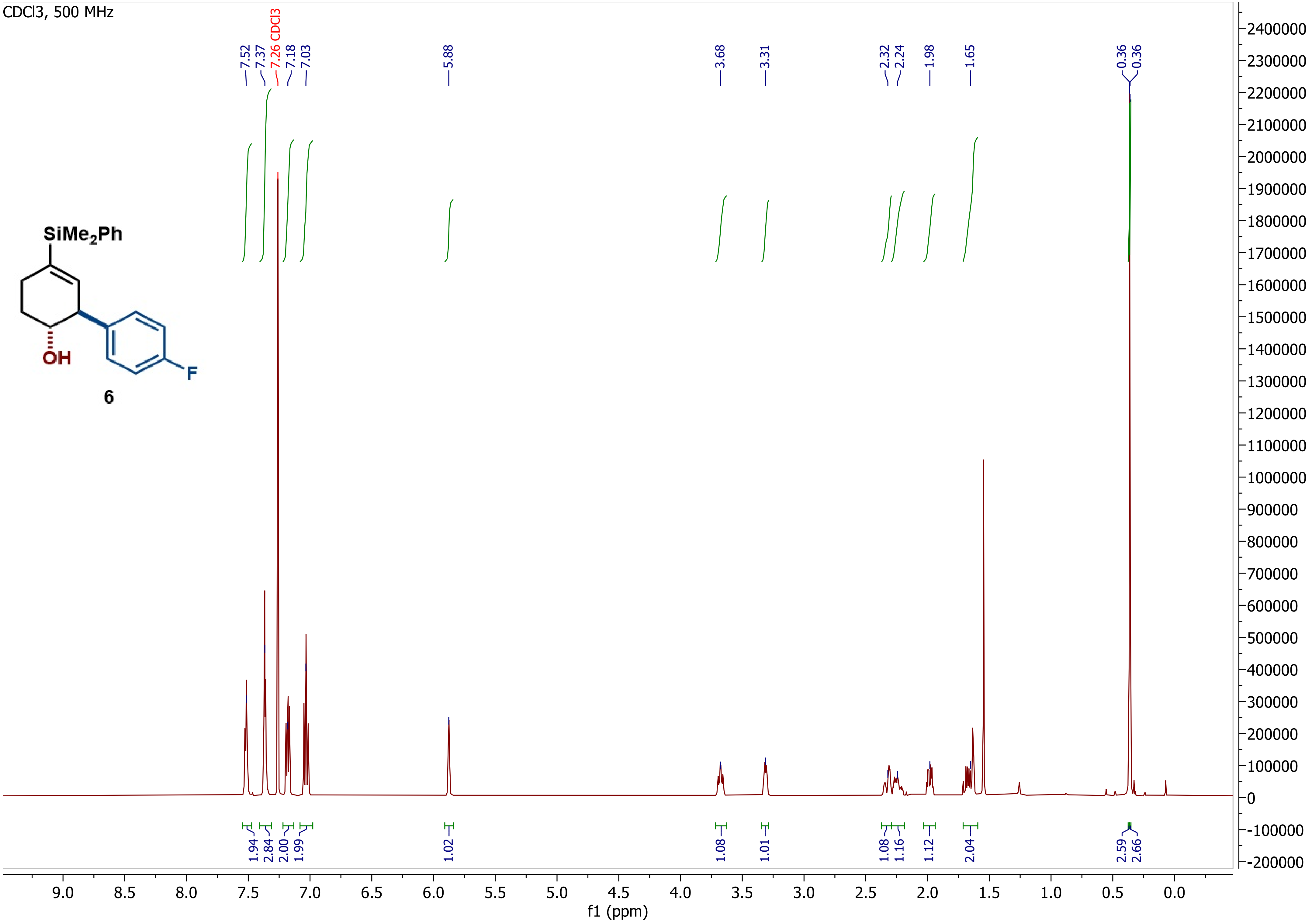

CDCl3, 500 MHz
7.52
7.37
7.26 CDCl3
7.18
7.03
5.88
3.68
3.31
2.32
2.24
1.98
1.65
0.36
0.36
SiMe2Ph
OH
F
6
1.94
2.84
2.00
1.99
1.02
1.08
1.01
1.08
1.16
1.12
2.04
2.59
2.66
f1 (ppm)

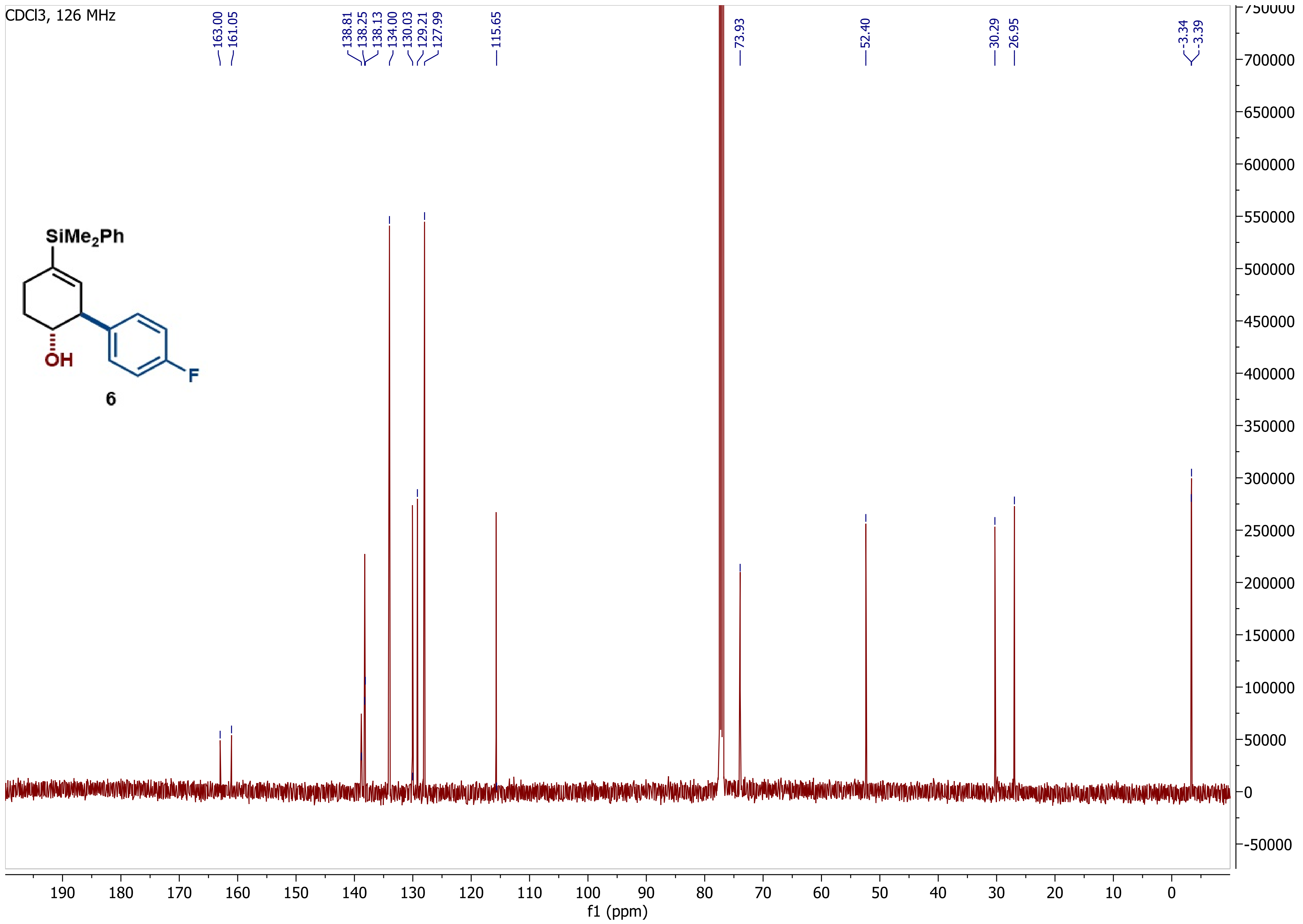

CDCl3, 126 MHz
163.00
161.05
138.81
138.25
138.13
134.00
130.03
129.21
127.99
115.65
73.93
52.40
30.29
26.95
-3.34
-3.39
SiMe2Ph
OH
F
6
f1 (ppm)

CDCl3, 400 MHz

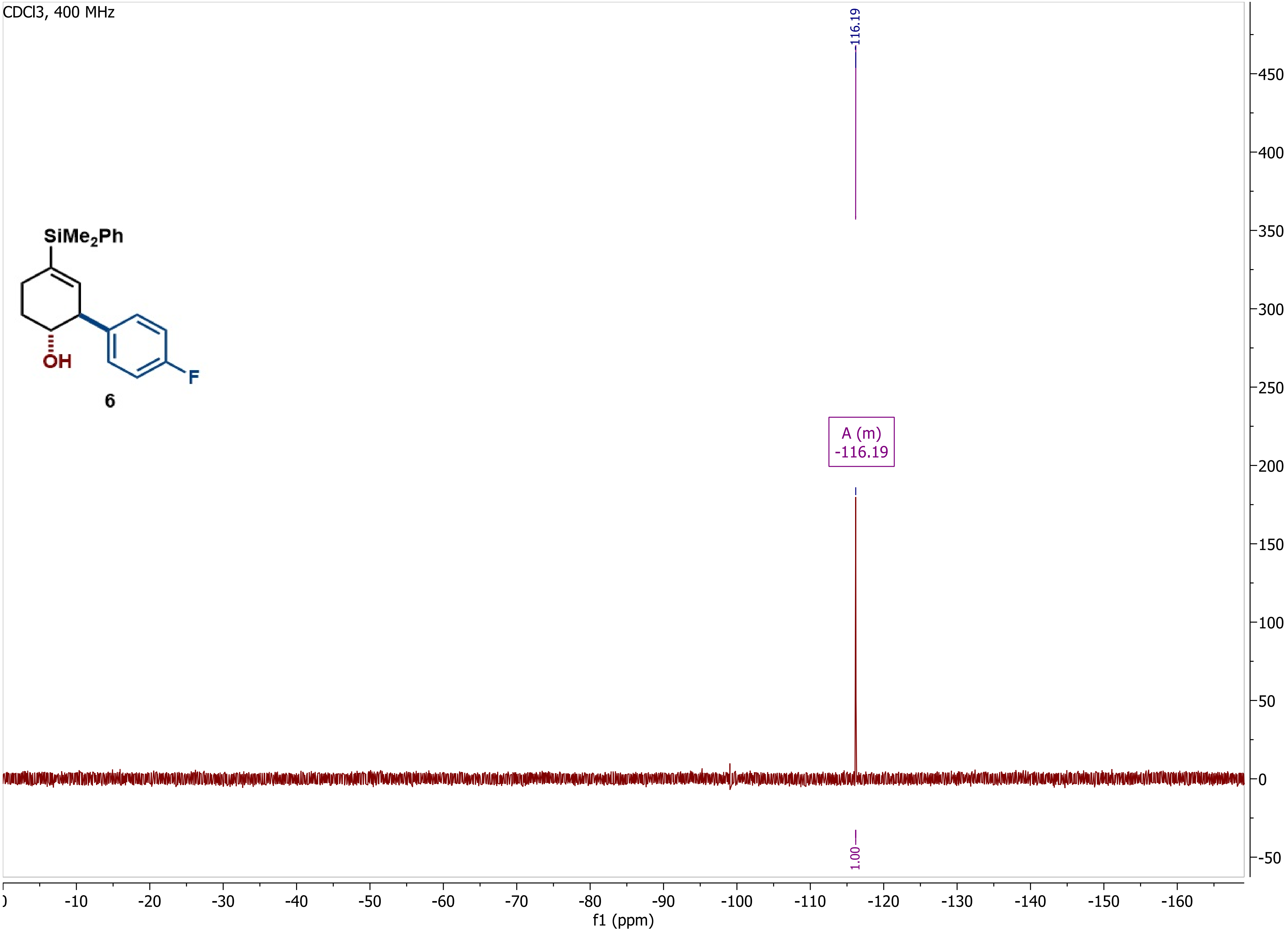

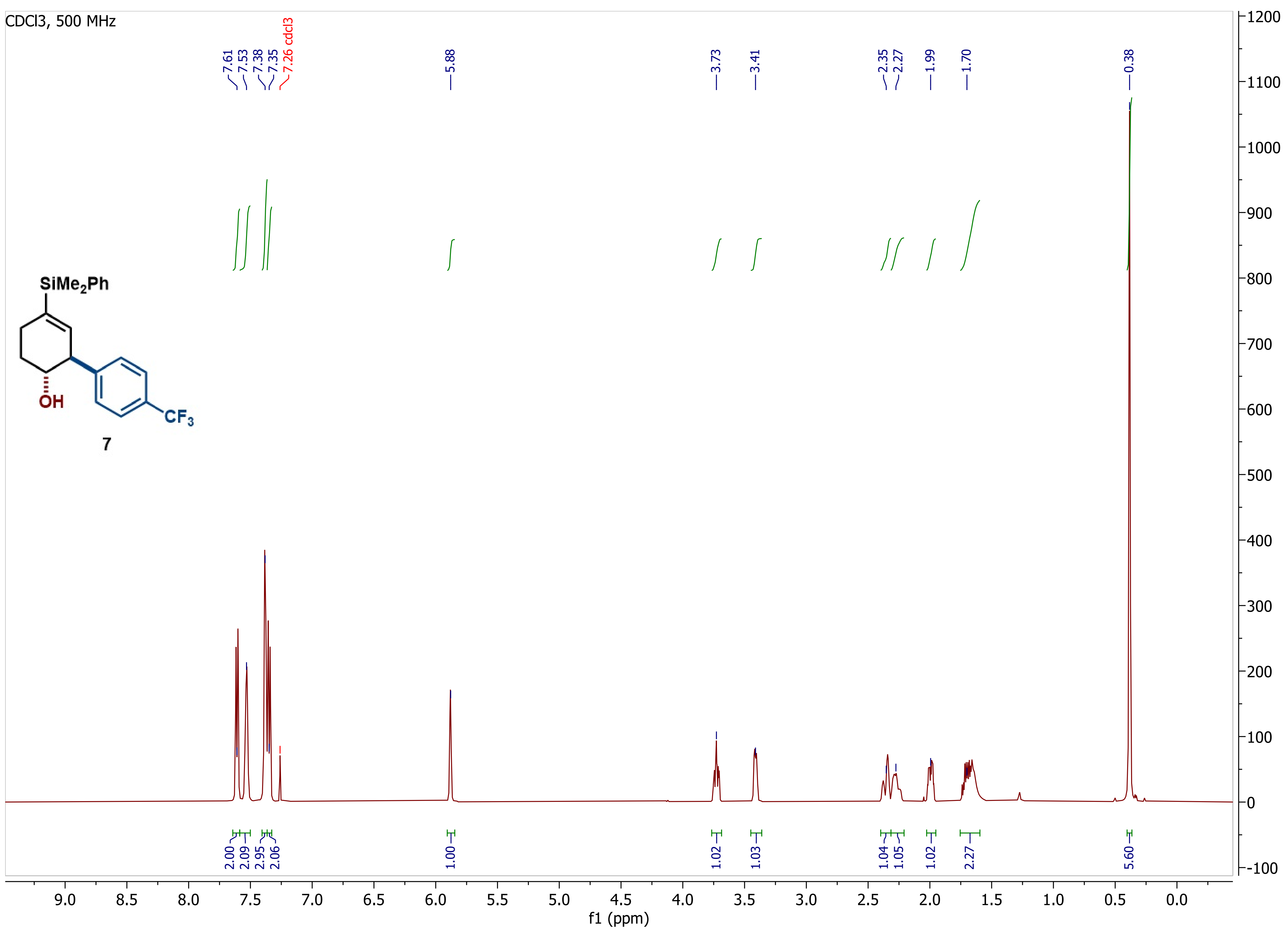
CDCl3, 500 MHz
7.61
7.53
7.38
7.35
7.26 cdcl3
5.88
3.73
3.41
2.35
2.27
1.99
1.70
0.38
SiMe2Ph
OH
CF3
7
2.00
2.09
2.95
2.06
1.00
1.02
1.03
1.04
1.05
1.02
2.27
5.60
f1 (ppm)

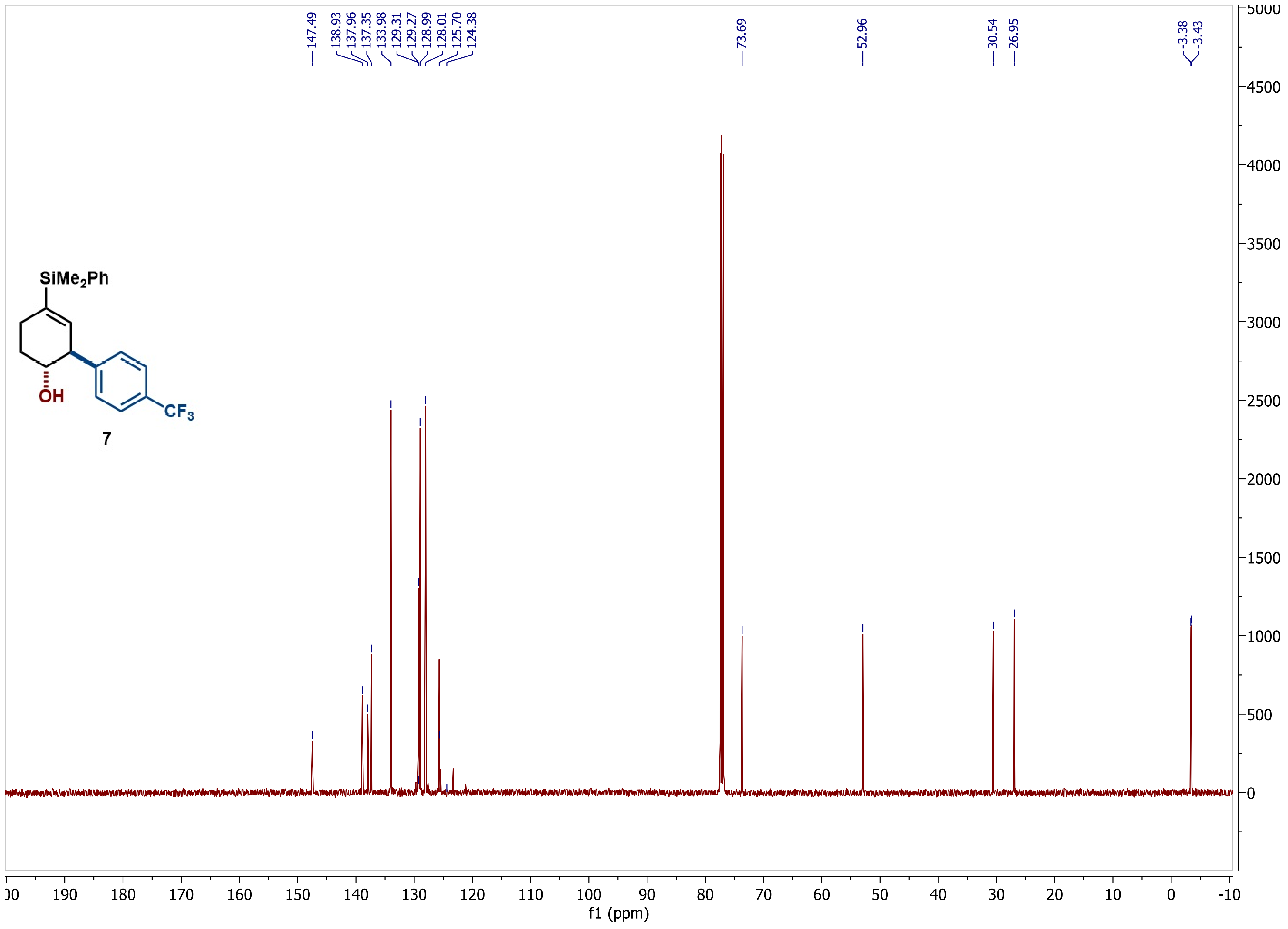

147.49
138.93
137.96
137.35
133.98
129.31
129.27
128.99
128.01
125.70
124.38
73.69
52.96
30.54
26.95
-3.38
-3.43
SiMe2Ph
OH
CF3
7
f1 (ppm)

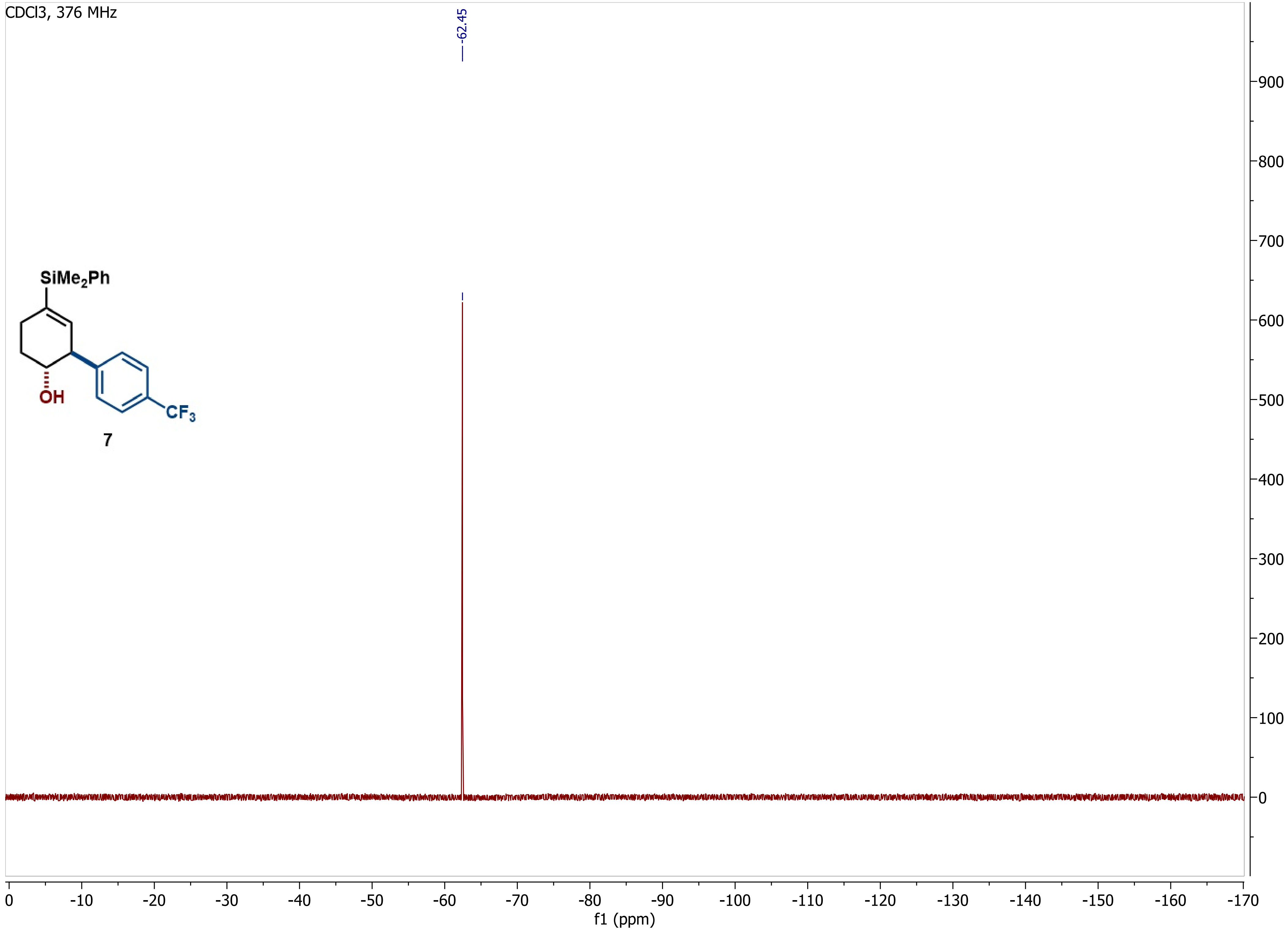
CDCl3, 376 MHz
-62.45
SiMe2Ph
OH
CF3
7
900
800
700
600
500
400
300
200
100
0
0
-10
-20
-30
-40
-50
-60
-70
-80
-90
-100
-110
-120
-130
-140
-150
-160
-170
f1 (ppm)

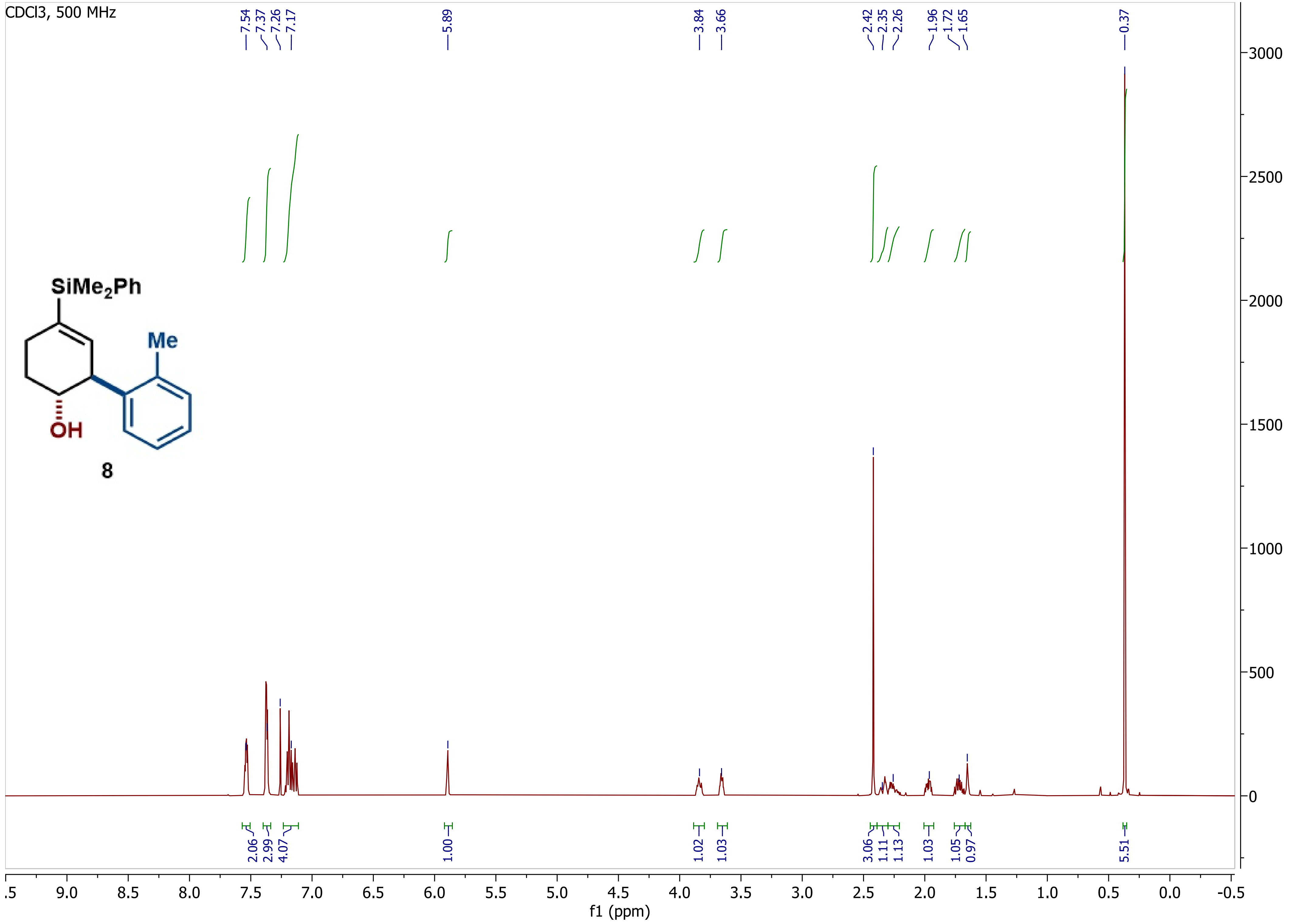

CDCl3, 500 MHz
7.54
7.37
7.26
7.17
5.89
3.84
3.66
2.42
2.35
2.26
1.96
1.72
1.65
0.37
SiMe2Ph
Me
OH
8
2.06
2.99
4.07
1.00
1.02
1.03
3.06
1.11
1.13
1.03
1.05
0.97
5.51
3000
2500
2000
1500
1000
500
0
f1 (ppm)

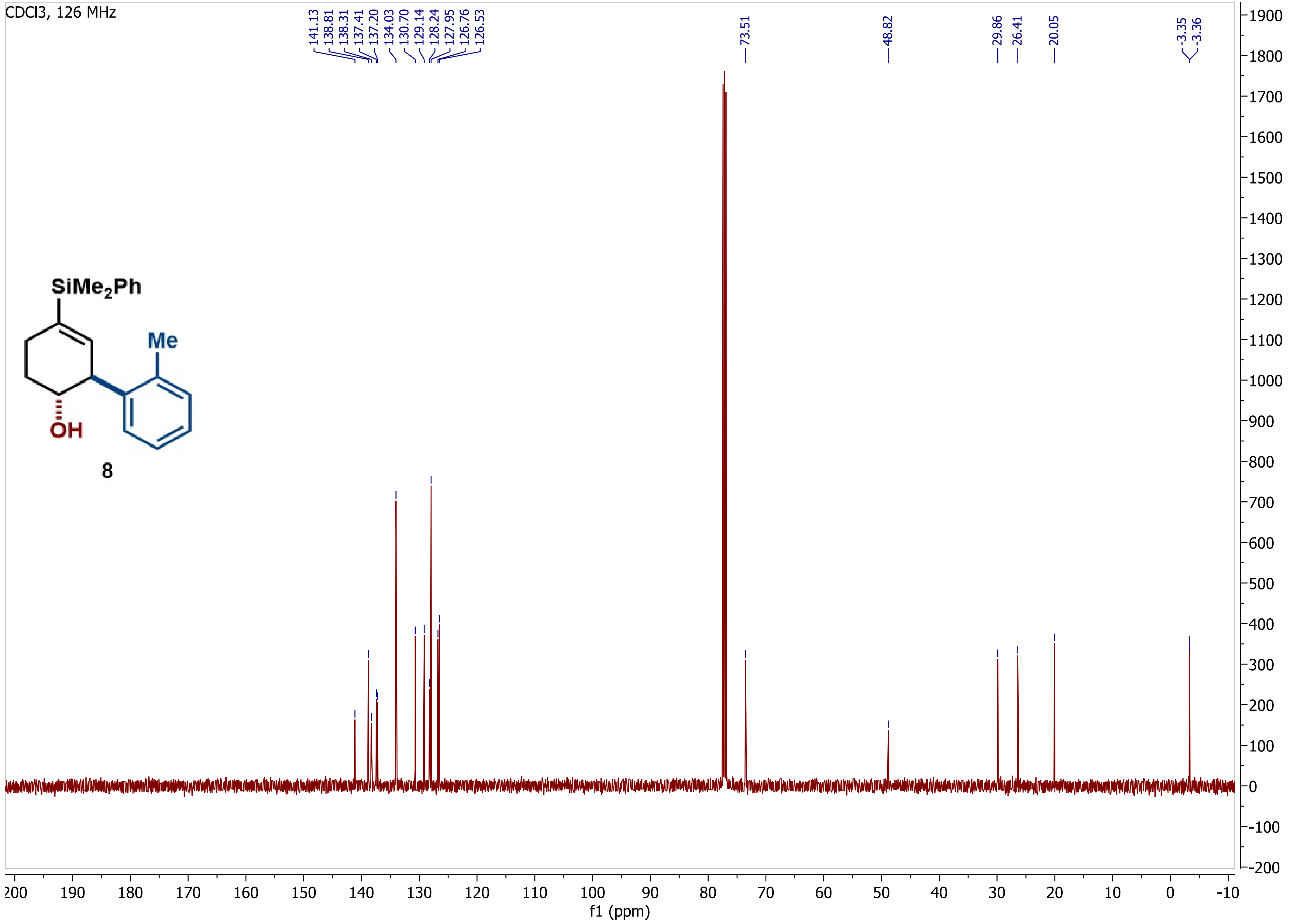
CDCl3, 126 MHz
141.13
138.81
138.31
137.41
137.20
134.03
130.70
129.14
128.24
127.95
126.76
126.53
73.51
48.82
29.86
26.41
20.05
-3.35
-3.36
SiMe2Ph
Me
OH
8
f1 (ppm)

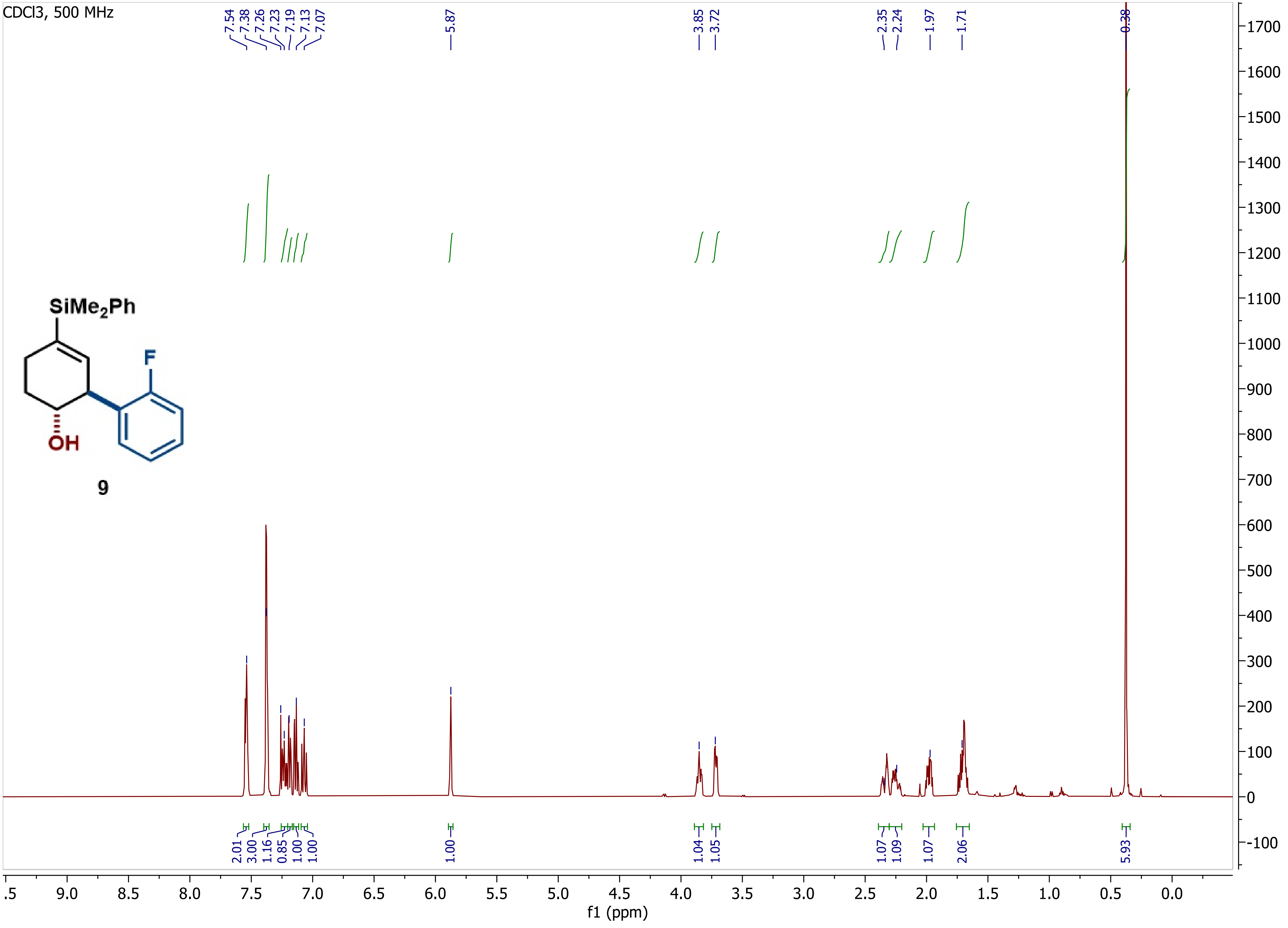

CDCl3, 500 MHz
7.54
7.38
7.26
7.23
7.19
7.13
7.07
5.87
3.85
3.72
2.35
2.24
1.97
1.71
0.38
2.01
3.00
1.16
0.85
1.00
1.00
1.00
1.04
1.05
1.07
1.09
1.07
2.06
5.93
f1 (ppm)
SiMe2Ph
F
OH

9

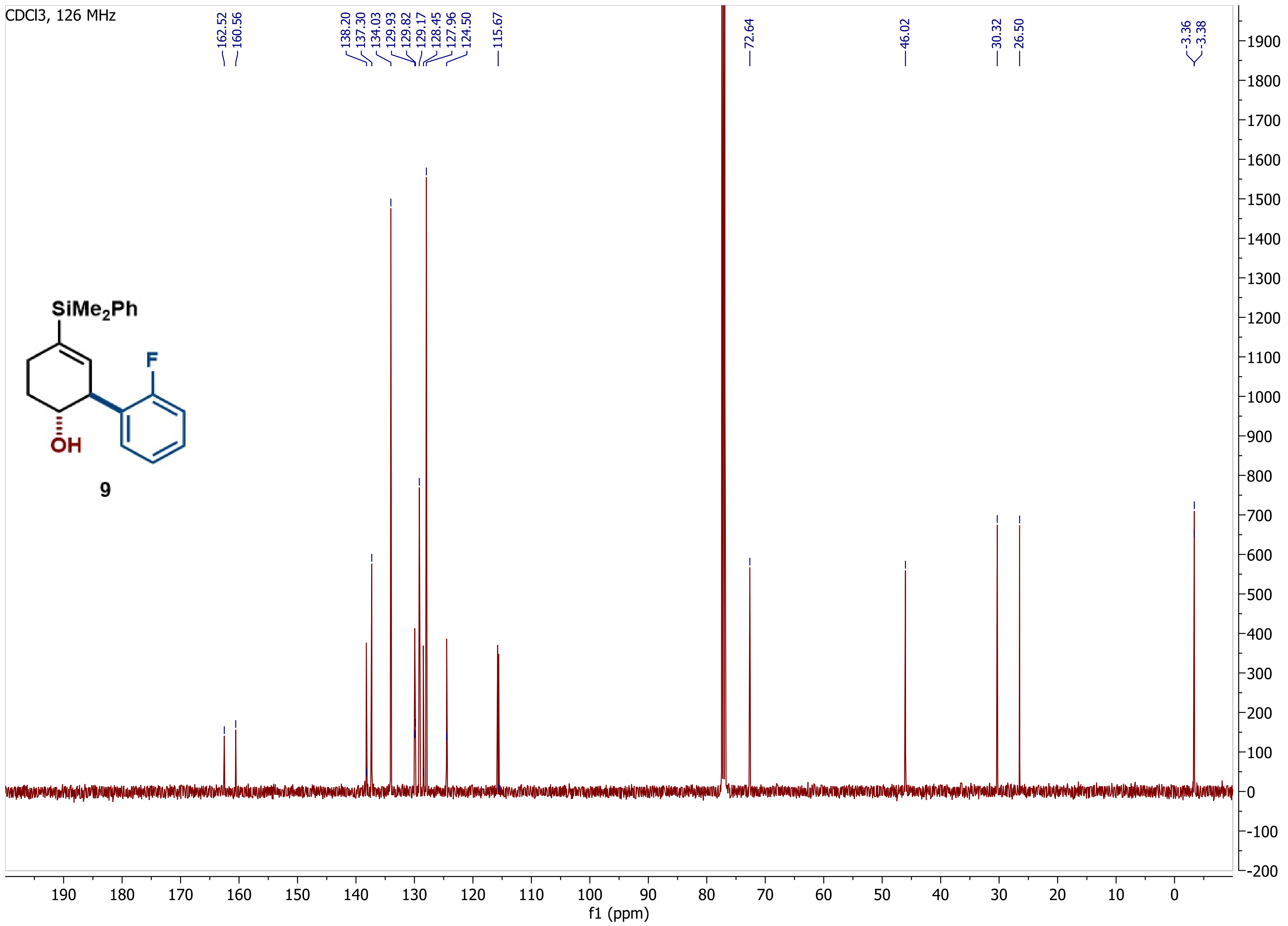
CDCl3, 126 MHz
162.52
160.56
138.20
137.30
134.03
129.93
129.82
129.17
128.45
127.96
124.50
115.67
72.64
46.02
30.32
26.50
-3.36
-3.38
SiMe2Ph
F
OH
9
f1 (ppm)

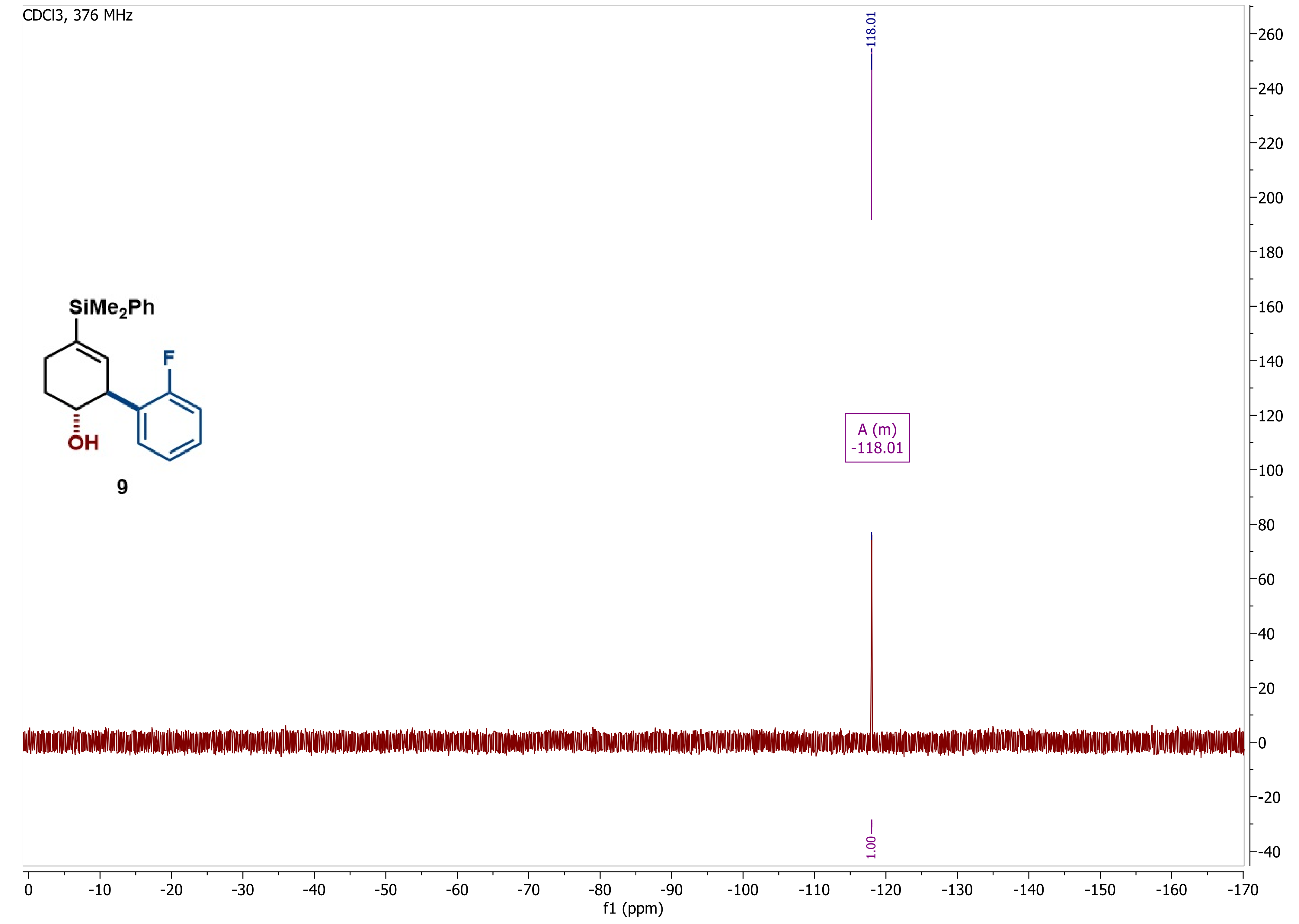
CDCl3, 376 MHz
-118.01
SiMe2Ph
F
OH
9
A (m)
-118.01
1.00
f1 (ppm)

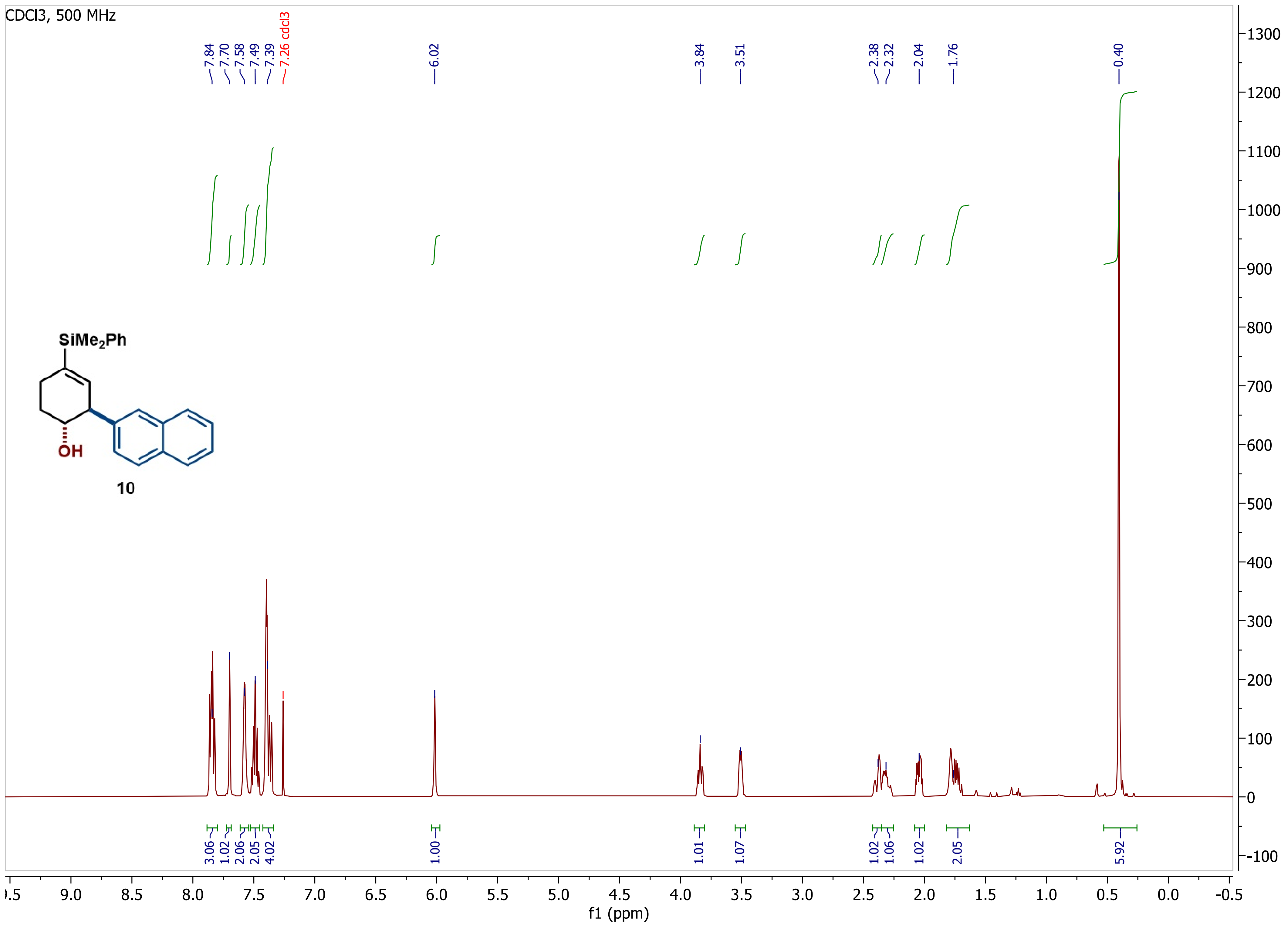
CDCl3, 500 MHz
7.84
7.70
7.58
7.49
7.39
7.26 cdcl3
6.02
3.84
3.51
2.38
2.32
2.04
1.76
0.40
SiMe2Ph
OH
10
3.06
1.02
2.06
2.05
4.02
1.00
1.01
1.07
1.02
1.06
1.02
2.05
5.92
f1 (ppm)

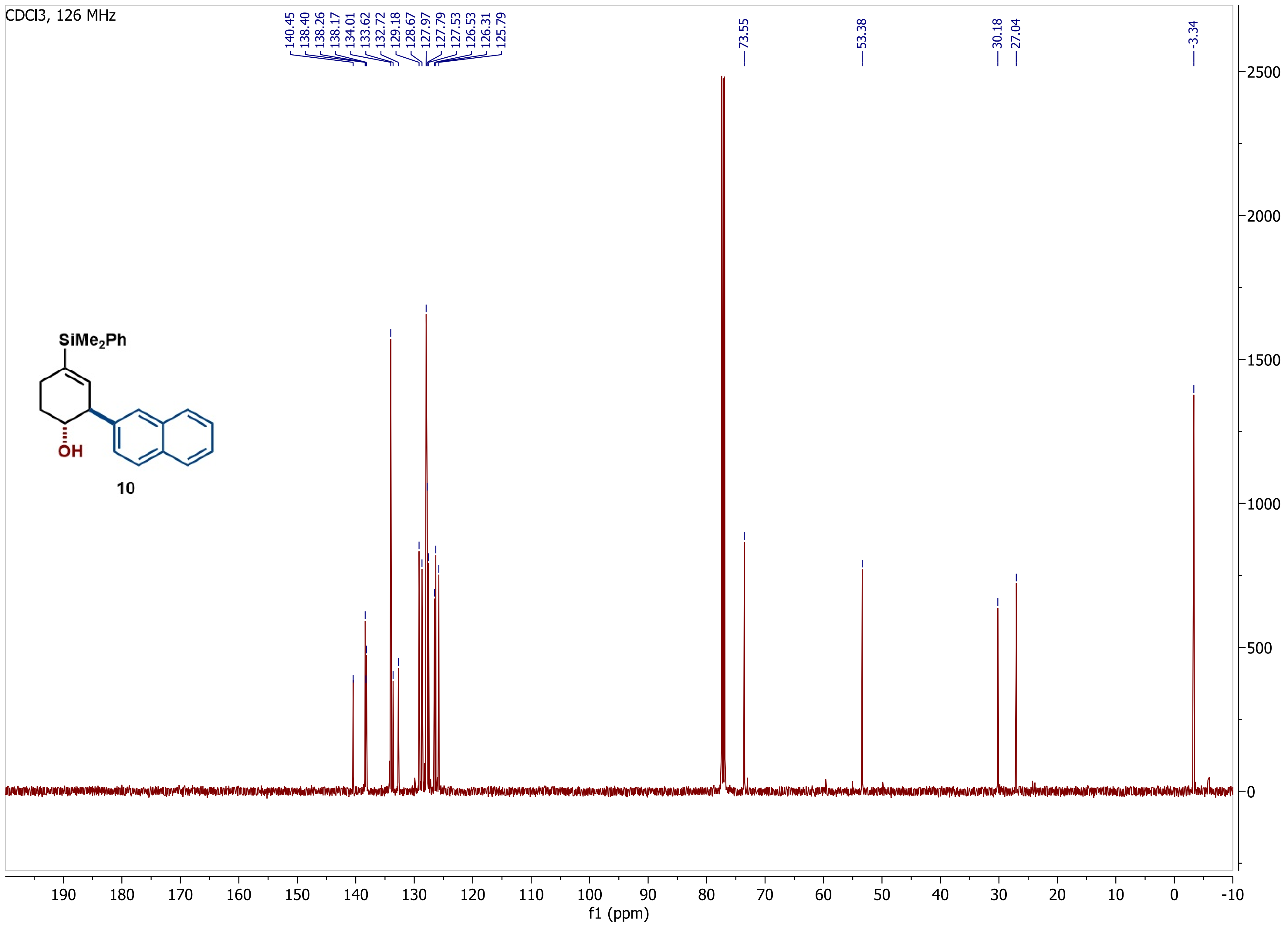

CDCl3, 126 MHz
140.45
138.40
138.26
138.17
134.01
133.62
132.72
129.18
128.67
127.97
127.79
127.53
126.53
126.31
125.79
73.55
53.38
30.18
27.04
-3.34
SiMe2Ph
OH
10
f1 (ppm)

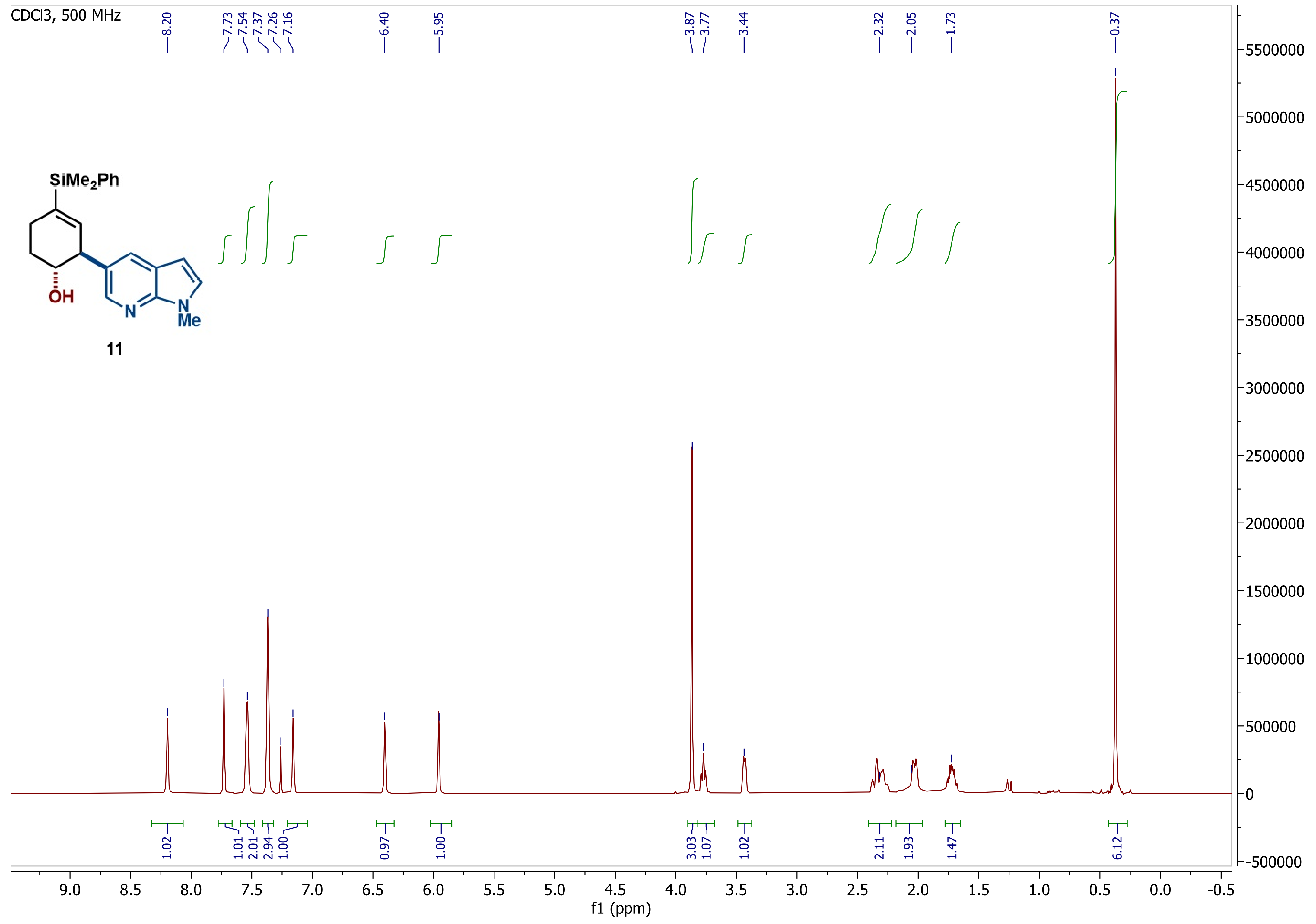

CDCl3, 500 MHz
8.20
7.73
7.54
7.37
7.26
7.16
6.40
5.95
3.87
3.77
3.44
2.32
2.05
1.73
0.37
SiMe2Ph
OH
N
N
Me
11
1.02
1.01
2.01
2.94
1.00
0.97
1.00
3.03
1.07
1.02
2.11
1.93
1.47
6.12
5500000
5000000
4500000
4000000
3500000
3000000
2500000
2000000
1500000
1000000
500000
0
-500000
9.0
8.5
8.0
7.5
7.0
6.5
6.0
5.5
5.0
4.5
4.0
3.5
3.0
2.5
2.0
1.5
1.0
0.5
0.0
-0.5
f1 (ppm)

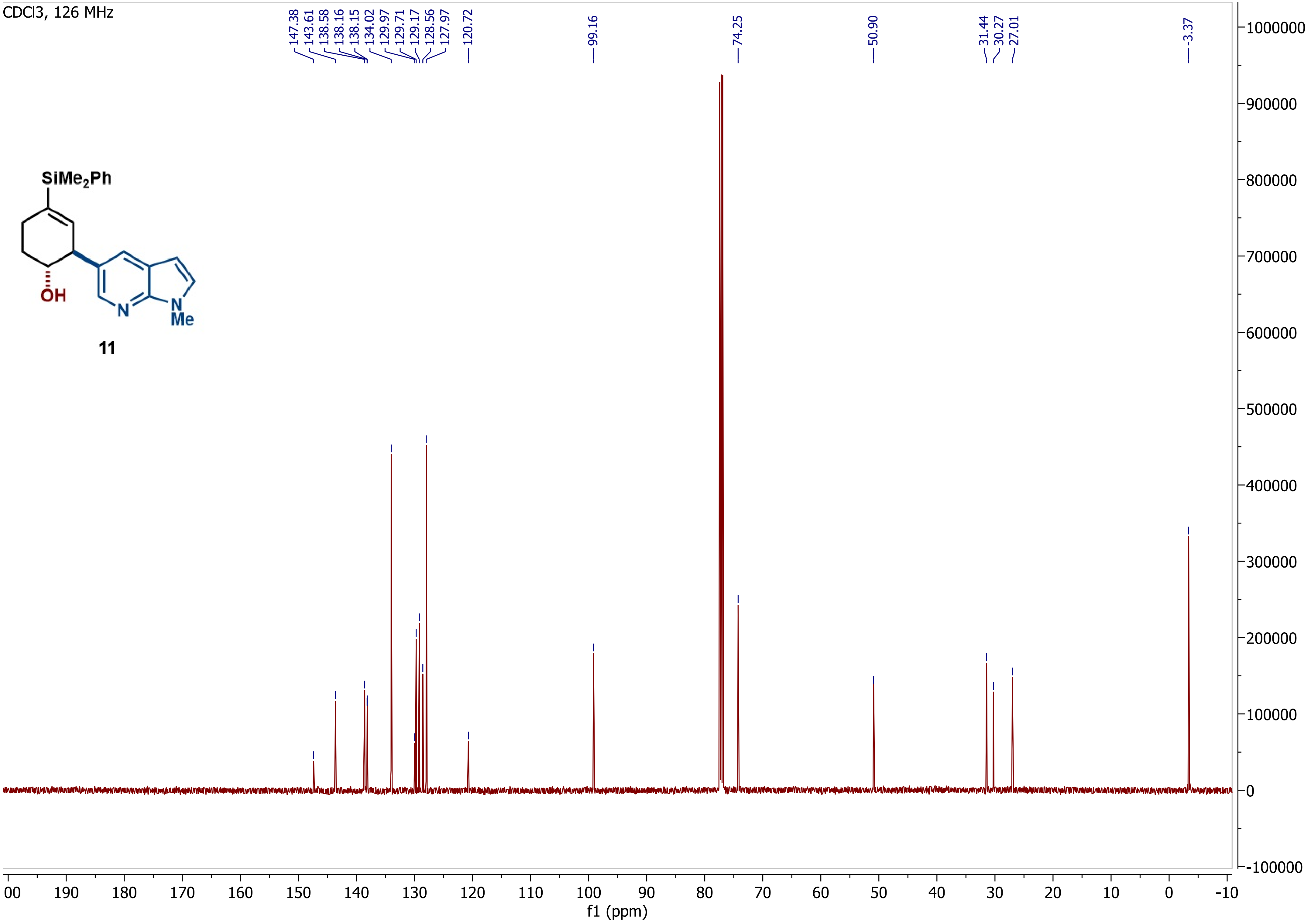
CDCl3, 126 MHz
147.38
143.61
138.58
138.16
138.15
134.02
129.97
129.71
129.17
128.56
127.97
120.72
99.16
74.25
50.90
31.44
30.27
27.01
-3.37
SiMe2Ph
OH
N
N
Me
11
f1 (ppm)

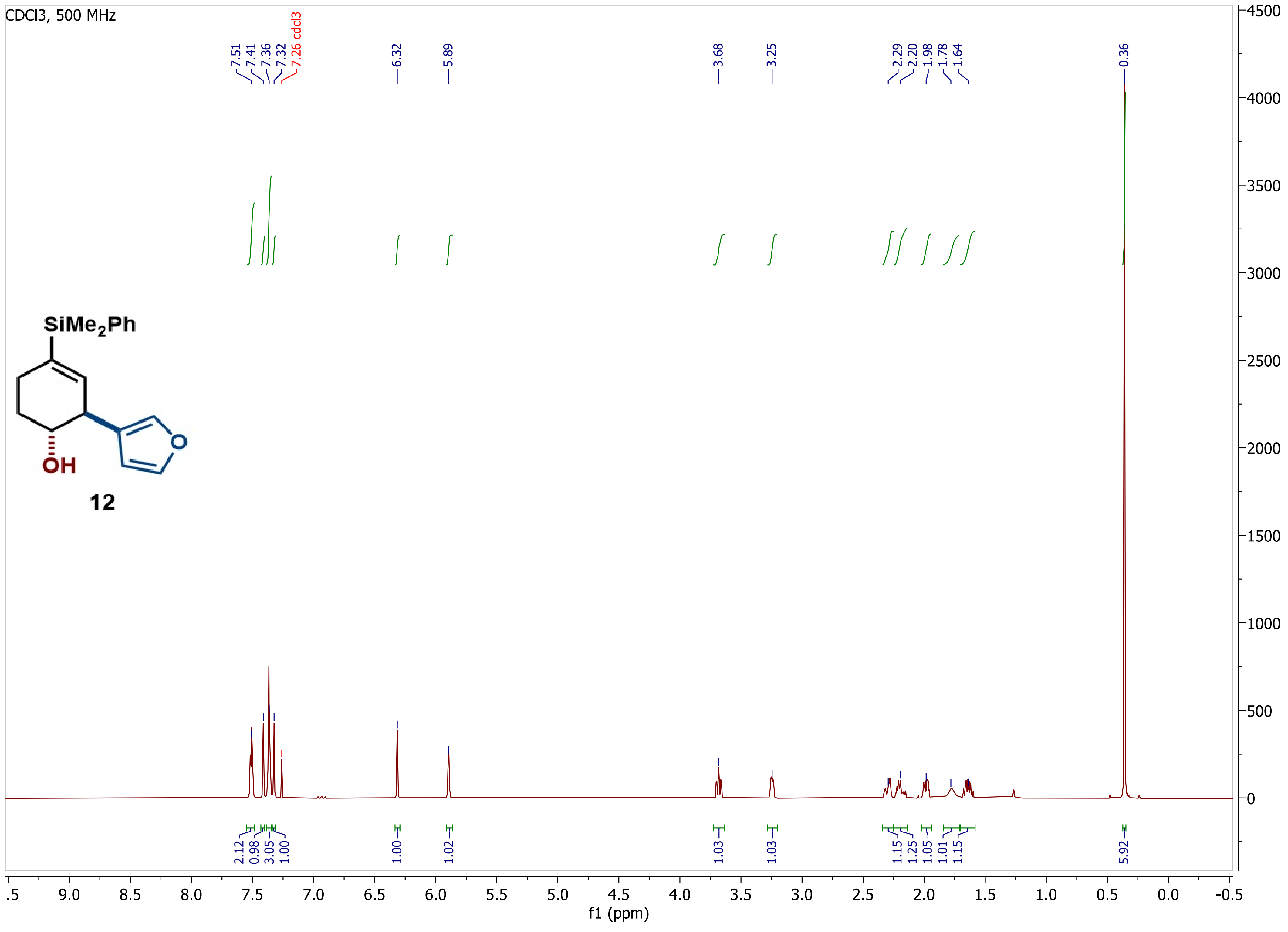
CDCl3, 500 MHz
7.51
7.41
7.36
7.32
7.26 cdcl3
6.32
5.89
3.68
3.25
2.29
2.20
1.98
1.78
1.64
0.36
SiMe2Ph
OH
O
12
2.12
0.98
3.05
1.00
1.00
1.02
1.03
1.03
1.15
1.25
1.05
1.01
1.15
5.92
f1 (ppm)

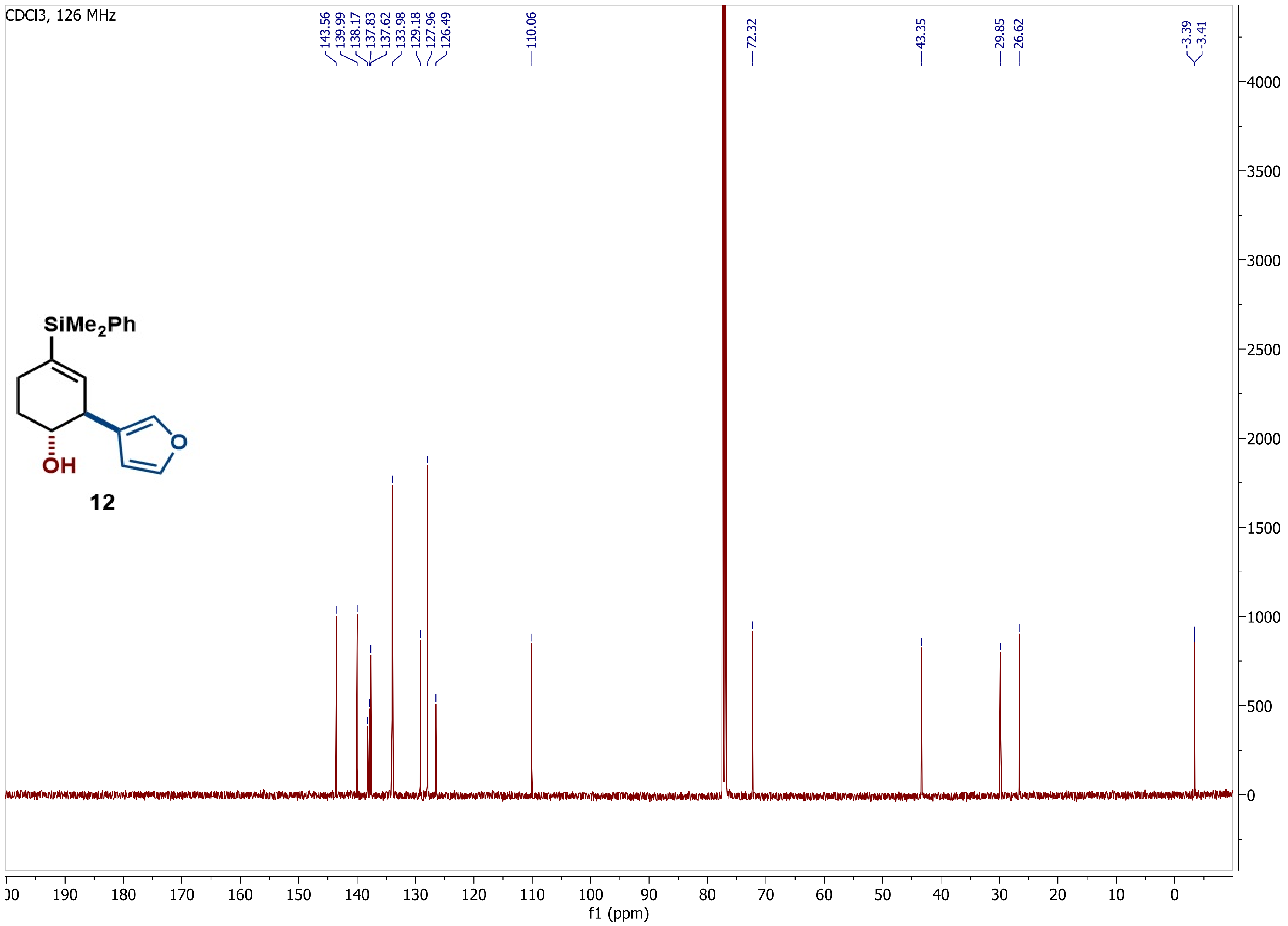

CDCl3, 126 MHz
143.56
139.99
138.17
137.83
137.62
133.98
129.18
127.96
126.49
110.06
72.32
43.35
29.85
26.62
-3.39
-3.41
SiMe2Ph
OH
O
12
4000
3500
3000
2500
2000
1500
1000
500
0
190
180
170
160
150
140
130
120
110
100
90
80
70
60
50
40
30
20
10
0
f1 (ppm)

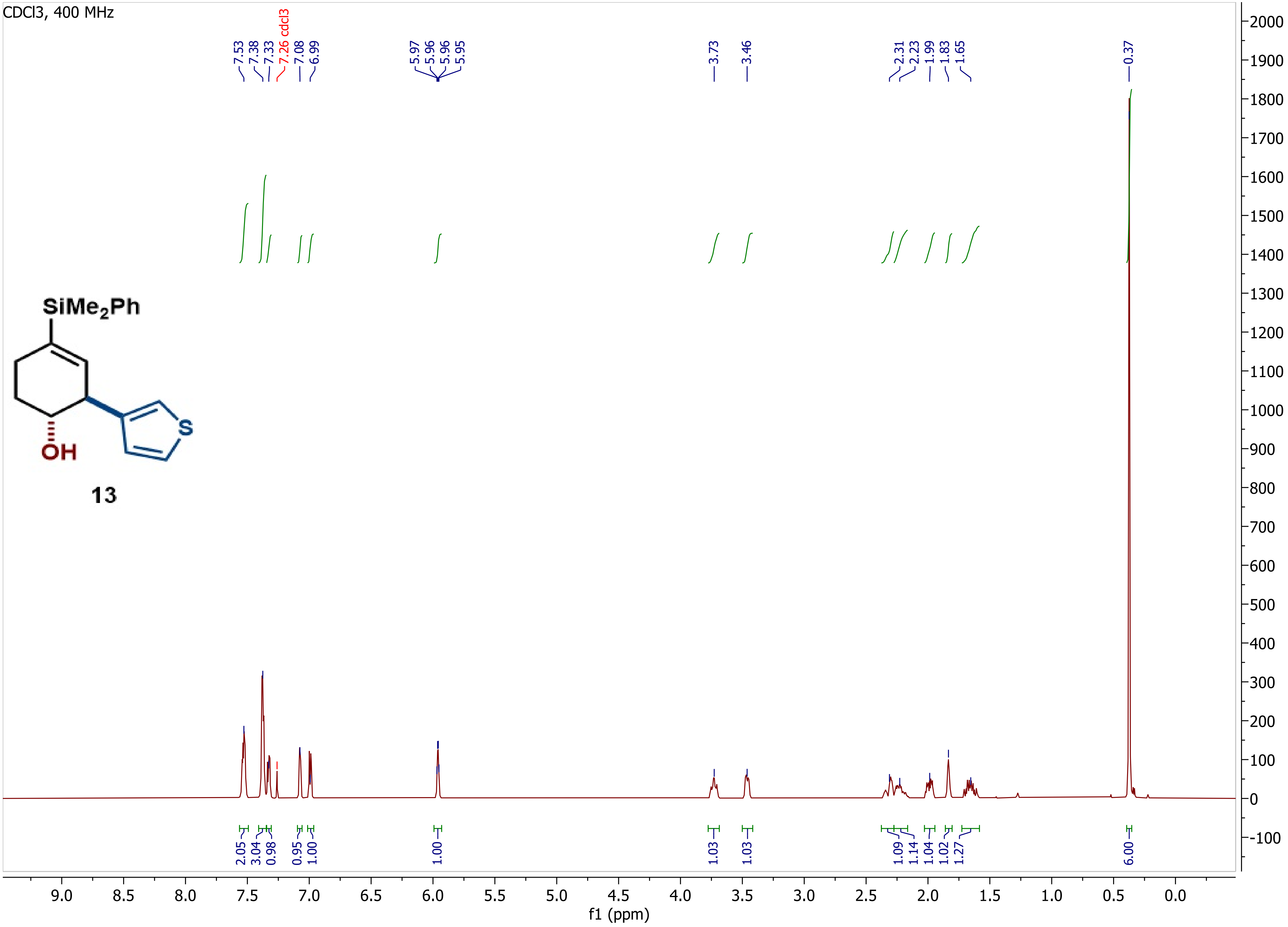

CDCl3, 400 MHz
7.53
7.38
7.33
7.26 cdcl3
7.08
6.99
5.97
5.96
5.96
5.95
3.73
3.46
2.31
2.23
1.99
1.83
1.65
0.37
SiMe2Ph
S
OH
13
2.05
3.04
0.98
0.95
1.00
1.00
1.03
1.03
1.09
1.14
1.04
1.02
1.27
6.00
f1 (ppm)

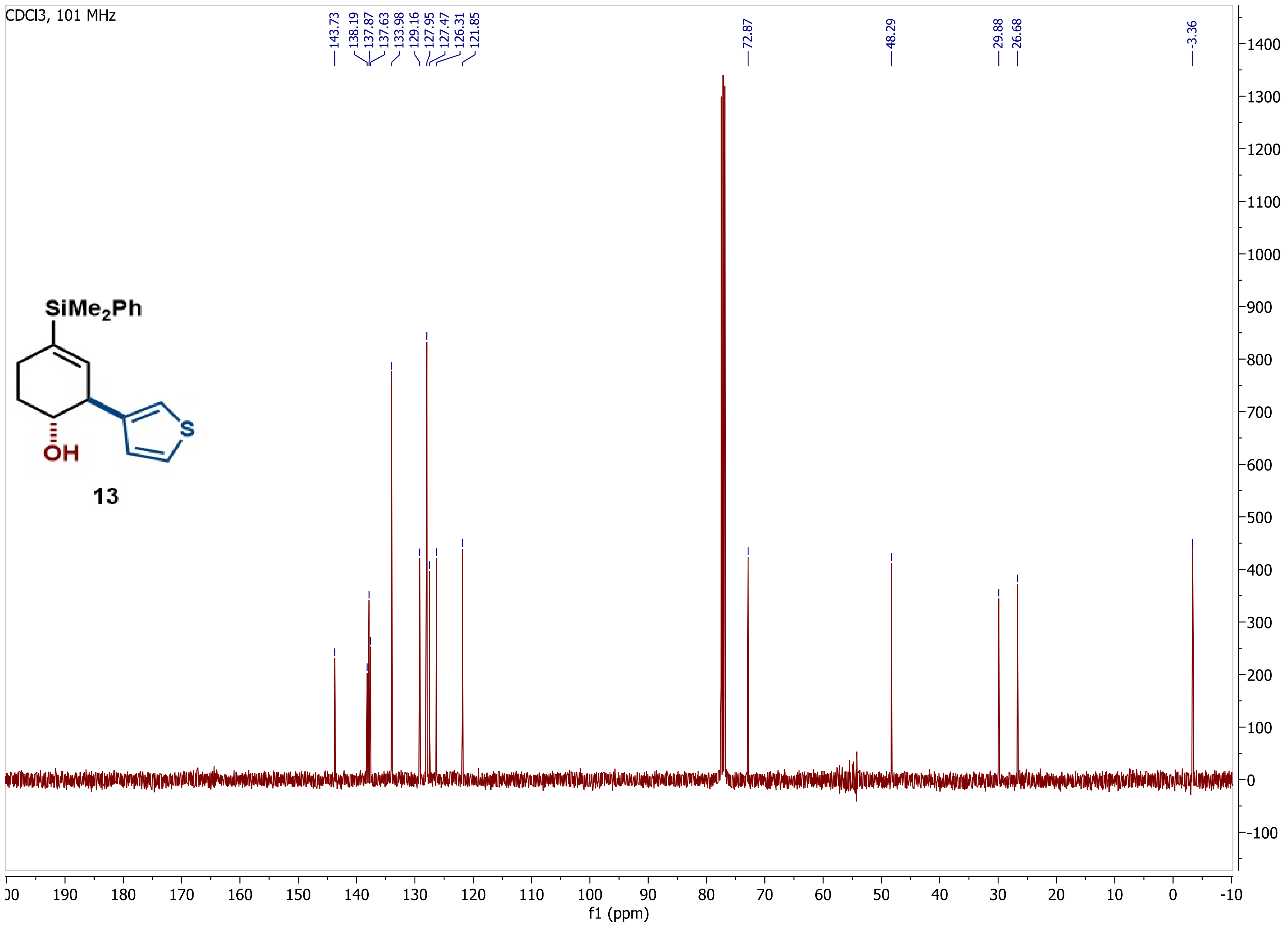

CDCl3, 101 MHz
143.73
138.19
137.87
137.63
133.98
129.16
127.95
127.47
126.31
121.85
72.87
48.29
29.88
26.68
-3.36
SiMe$_2$Ph
S
OH
13
f1 (ppm)

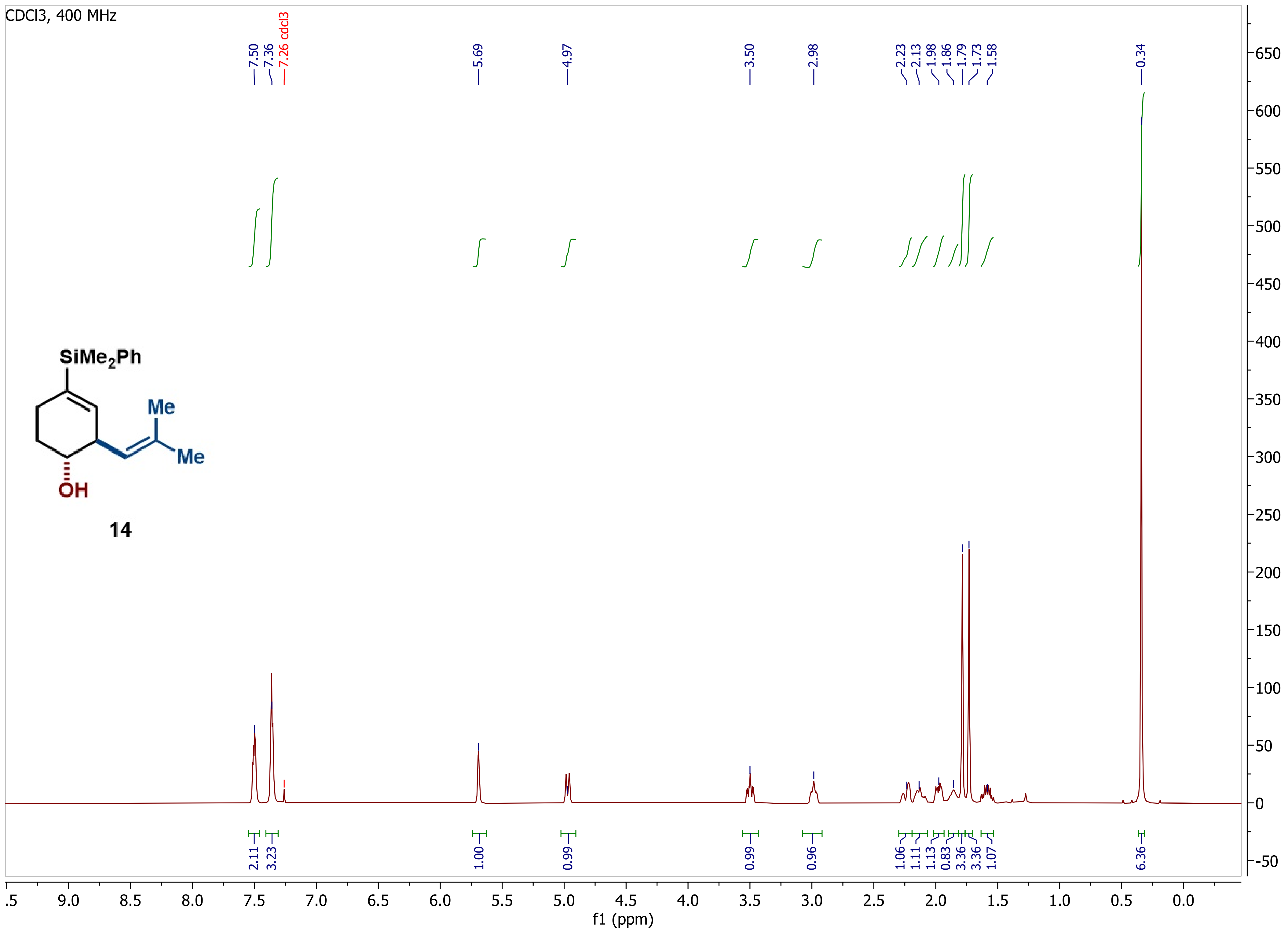

CDCl3, 400 MHz
7.50
7.36
7.26 cdcl3
5.69
4.97
3.50
2.98
2.23
2.13
1.98
1.86
1.79
1.73
1.58
0.34
SiMe2Ph
Me
Me
OH
14
2.11
3.23
1.00
0.99
0.99
0.96
1.06
1.11
1.13
0.83
3.36
3.36
1.07
6.36
f1 (ppm)

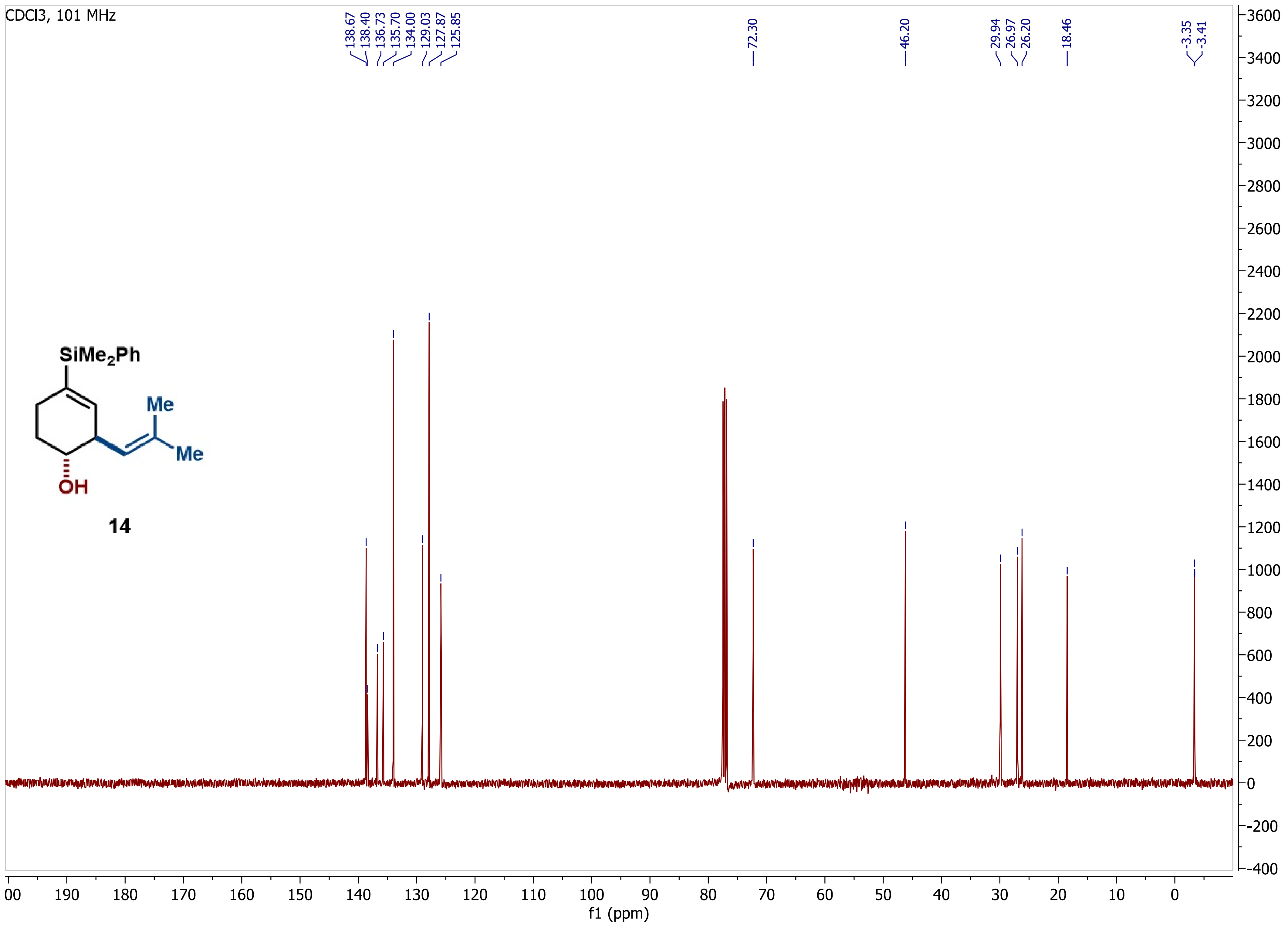

CDCl3, 101 MHz
138.67
138.40
136.73
135.70
134.00
129.03
127.87
125.85
72.30
46.20
29.94
26.97
26.20
18.46
-3.35
-3.41
SiMe2Ph
Me
Me
OH
14
f1 (ppm)

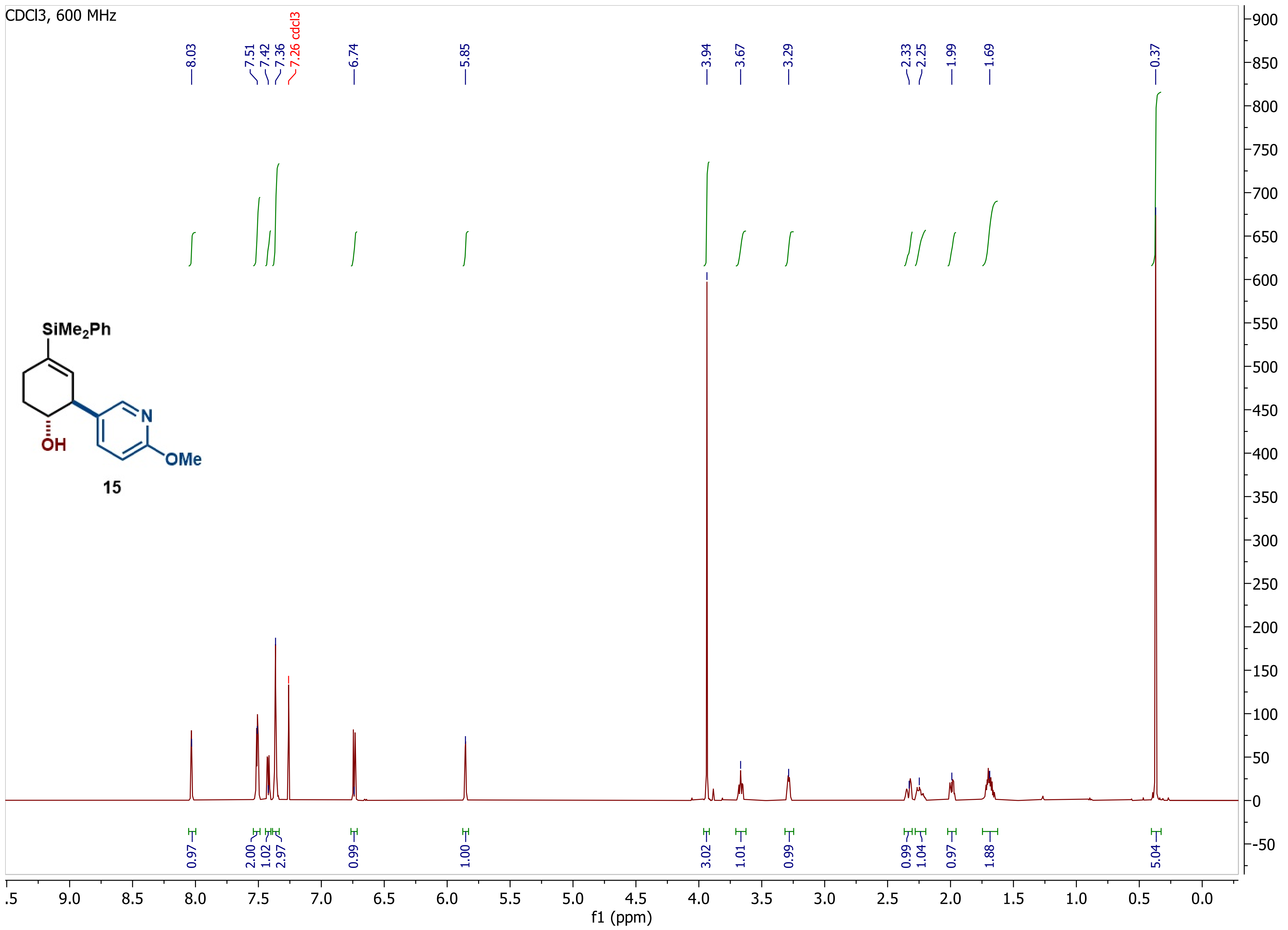

CDCl3, 600 MHz
8.03
7.51
7.42
7.36
7.26 cdcl3
6.74
5.85
3.94
3.67
3.29
2.33
2.25
1.99
1.69
0.37
SiMe2Ph
OH
N
OMe
0.97
2.00
1.02
2.97
0.99
1.00
3.02
1.01
0.99
0.99
1.04
0.97
1.88
5.04
f1 (ppm)

15

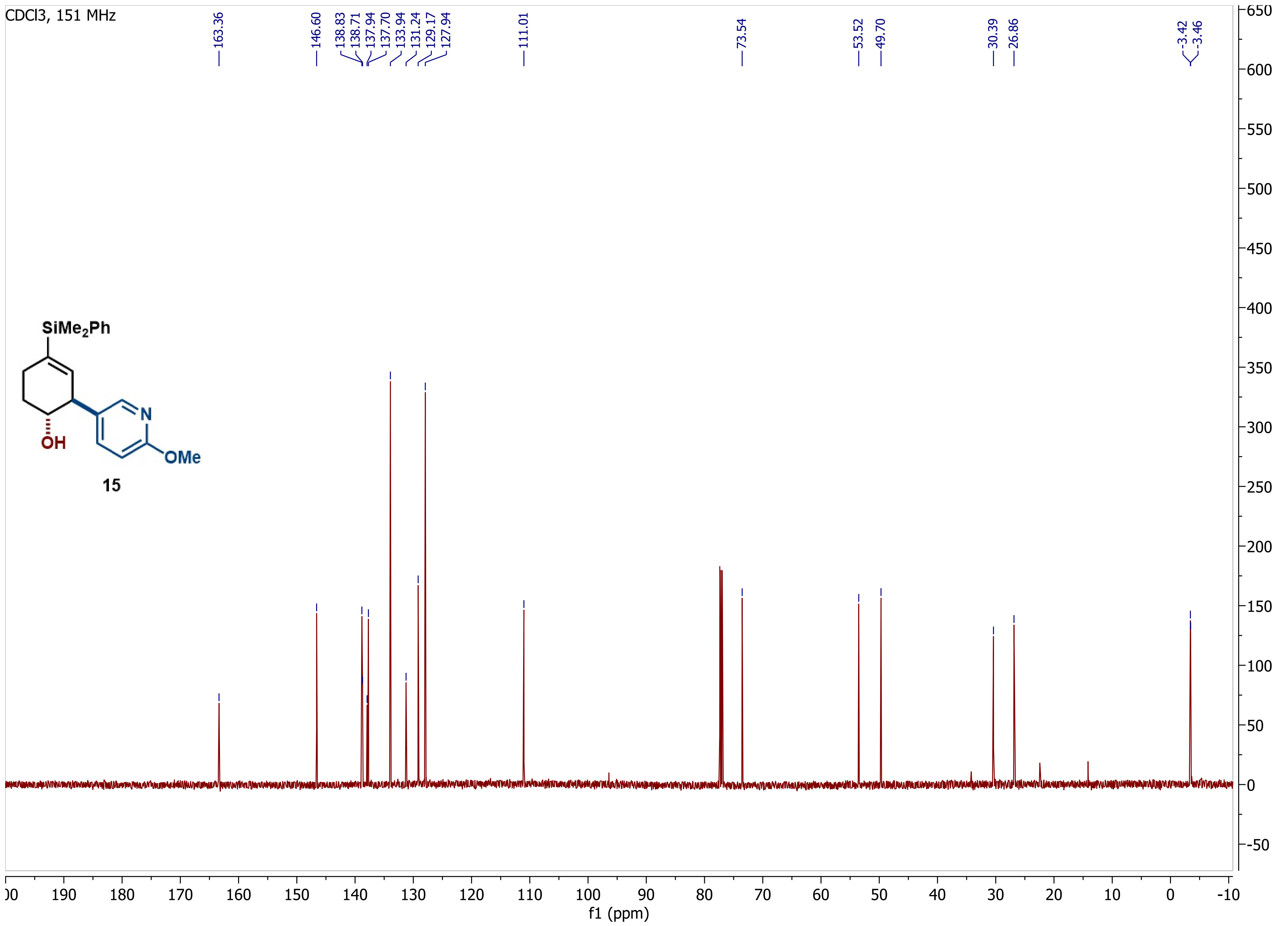
CDCl3, 151 MHz
163.36
146.60
138.83
138.71
137.94
137.70
133.94
131.24
129.17
127.94
111.01
73.54
53.52
49.70
30.39
26.86
-3.42
-3.46
SiMe2Ph
N
OH
OMe
15
f1 (ppm)

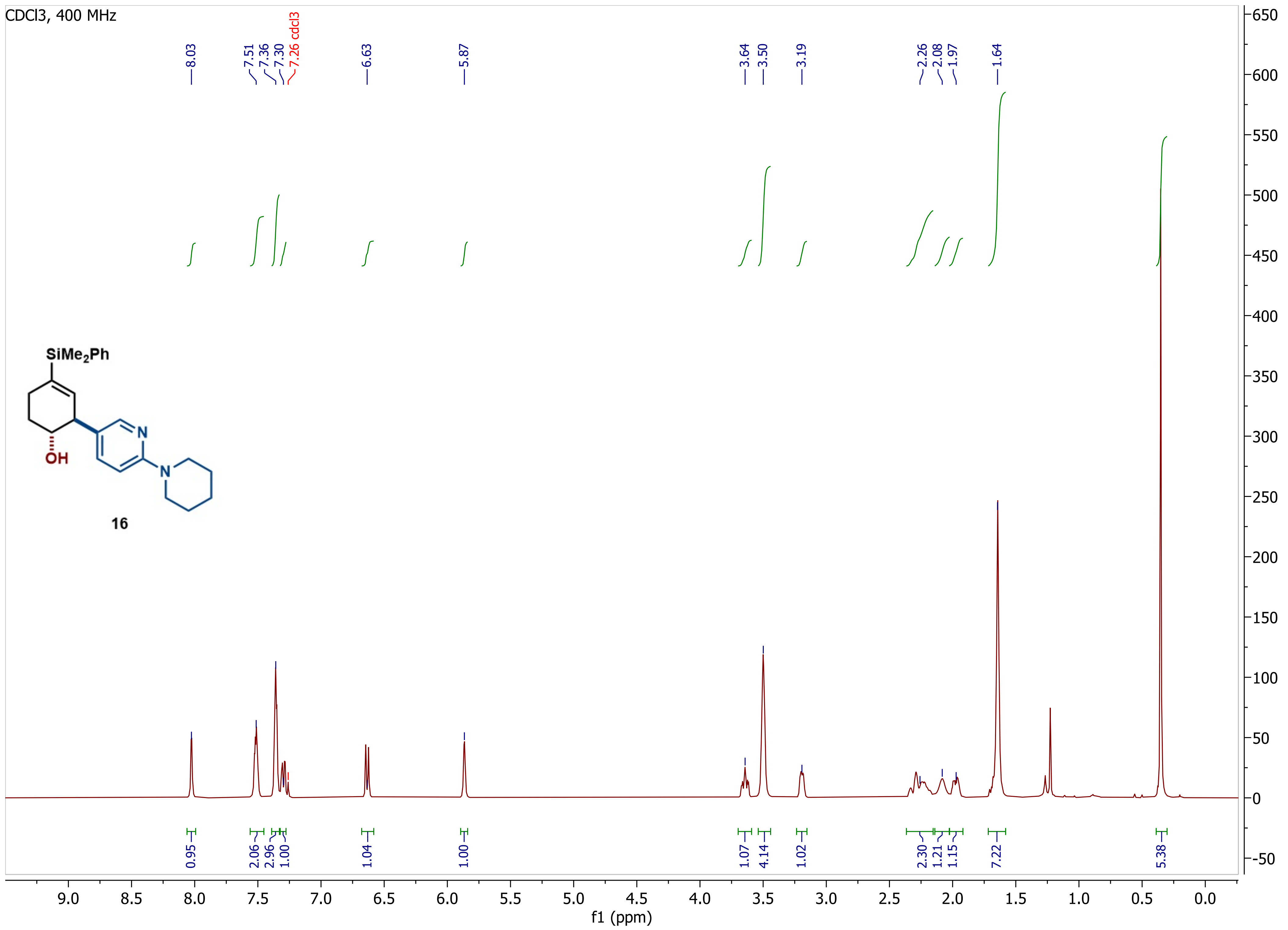
CDCl3, 400 MHz
8.03
7.51
7.36
7.30
7.26 cdcl3
6.63
5.87
3.64
3.50
3.19
2.26
2.08
1.97
1.64
SiMe2Ph
N
OH
N
16
0.95
2.06
2.96
1.00
1.04
1.00
1.07
4.14
1.02
2.30
1.21
1.15
7.22
5.38
f1 (ppm)

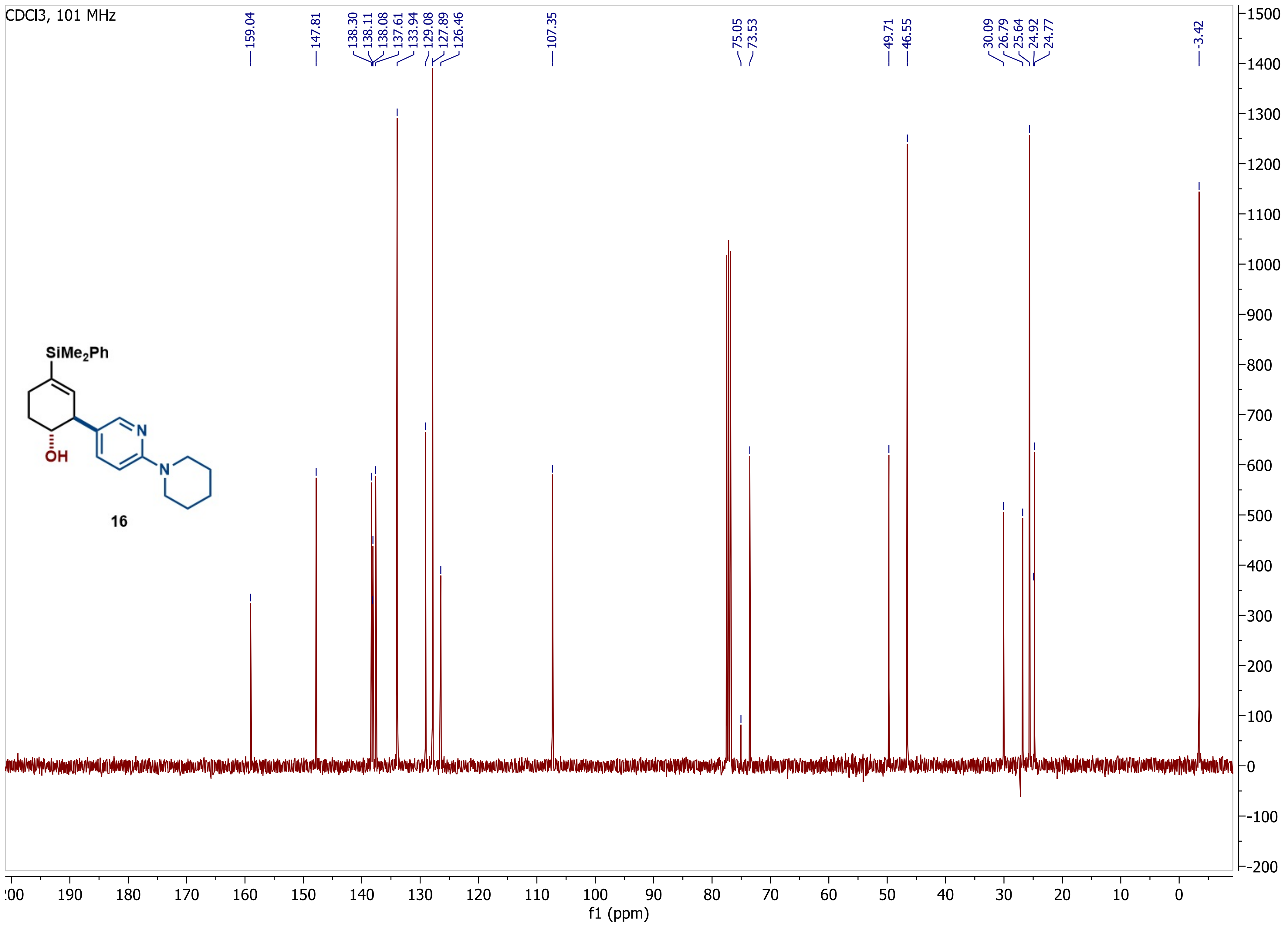

CDCl3, 101 MHz
159.04
147.81
138.30
138.11
138.08
137.61
133.94
129.08
127.89
126.46
107.35
75.05
73.53
49.71
46.55
30.09
26.79
25.64
24.92
24.77
-3.42
SiMe2Ph
N
OH
N
16
f1 (ppm)

CDCl3, 600 MHz

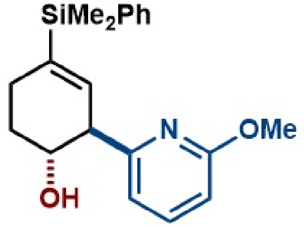

17

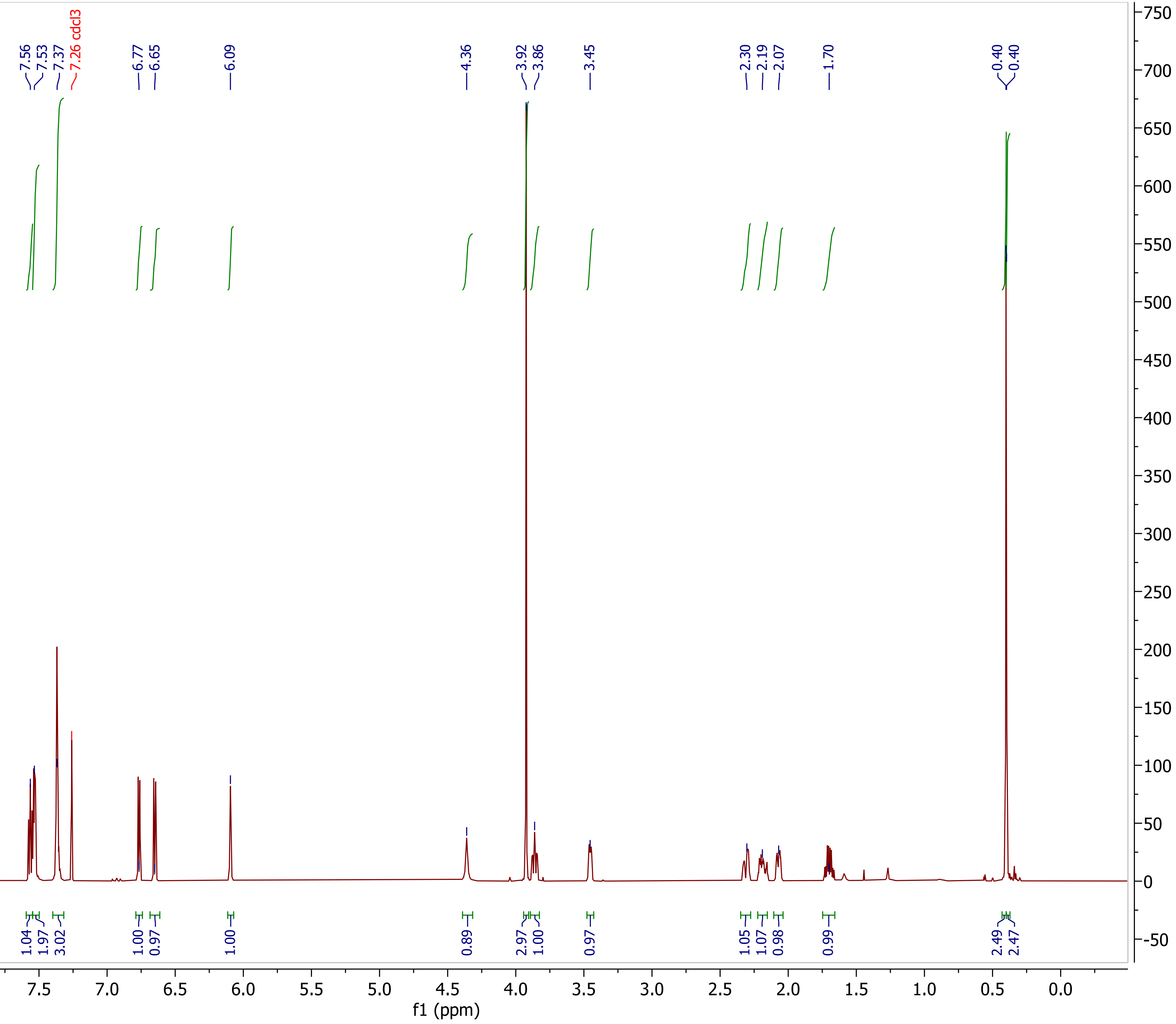

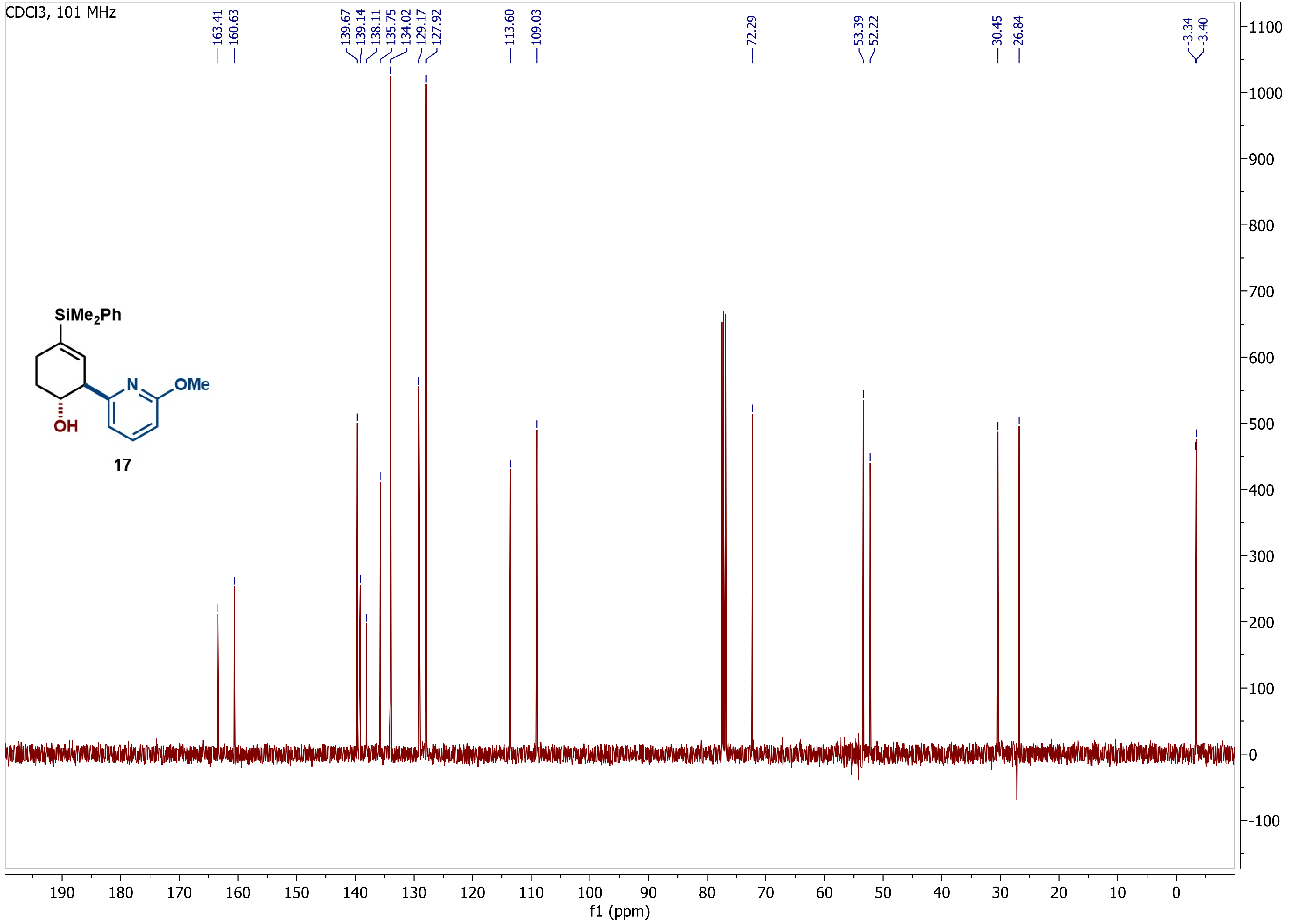

CDCl3, 101 MHz
163.41
160.63
139.67
139.14
138.11
135.75
134.02
129.17
127.92
113.60
109.03
72.29
53.39
52.22
30.45
26.84
-3.34
-3.40
SiMe2Ph
N
OMe
OH
17
f1 (ppm)

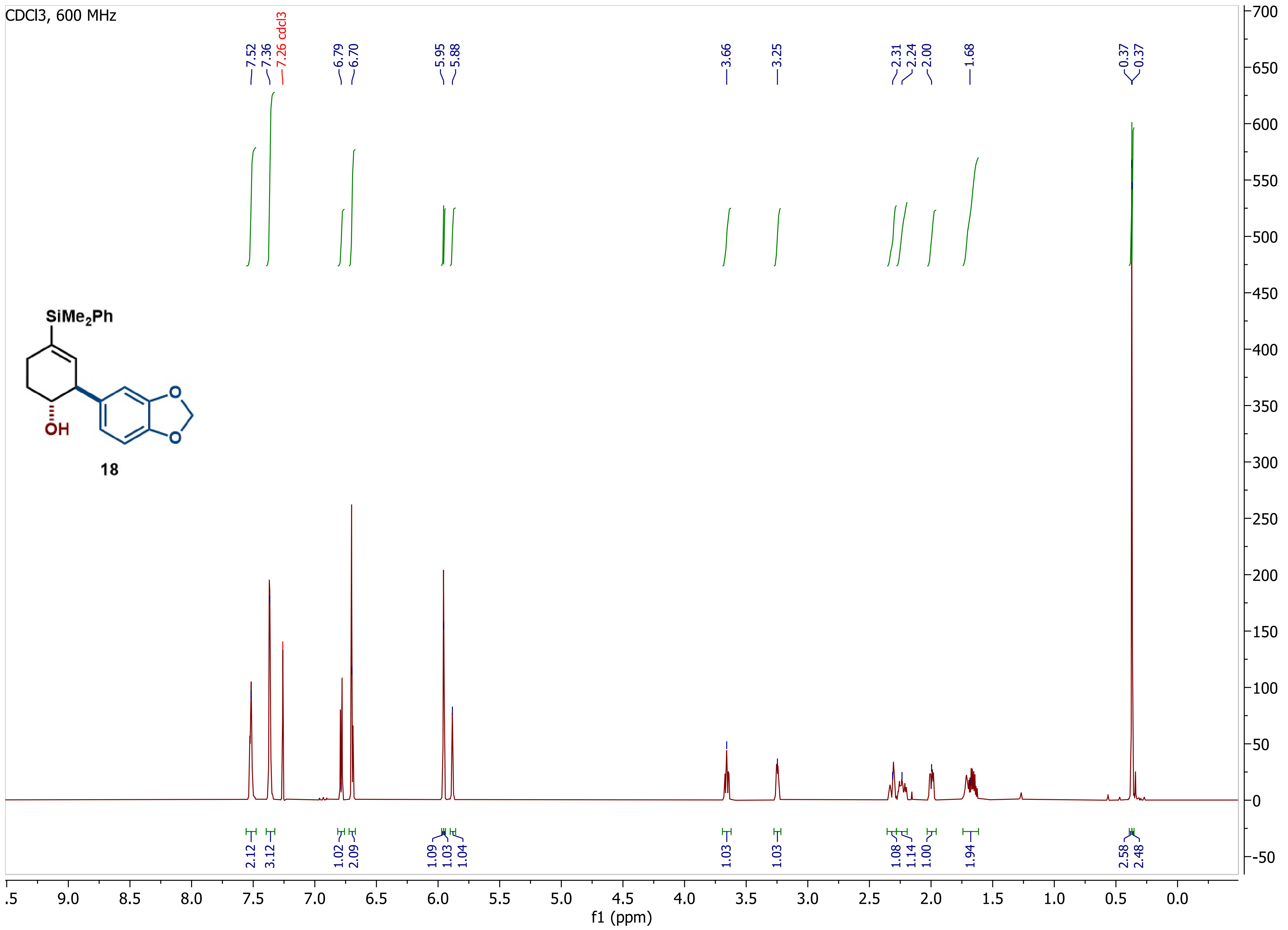

CDCl3, 600 MHz
7.52
7.36
7.26 cdcl3
6.79
6.70
5.95
5.88
3.66
3.25
2.31
2.24
2.00
1.68
0.37
0.37
SiMe2Ph
OH
O
O
18
2.12
3.12
1.02
2.09
1.09
1.03
1.04
1.03
1.03
1.08
1.14
1.00
1.94
2.58
2.48
f1 (ppm)

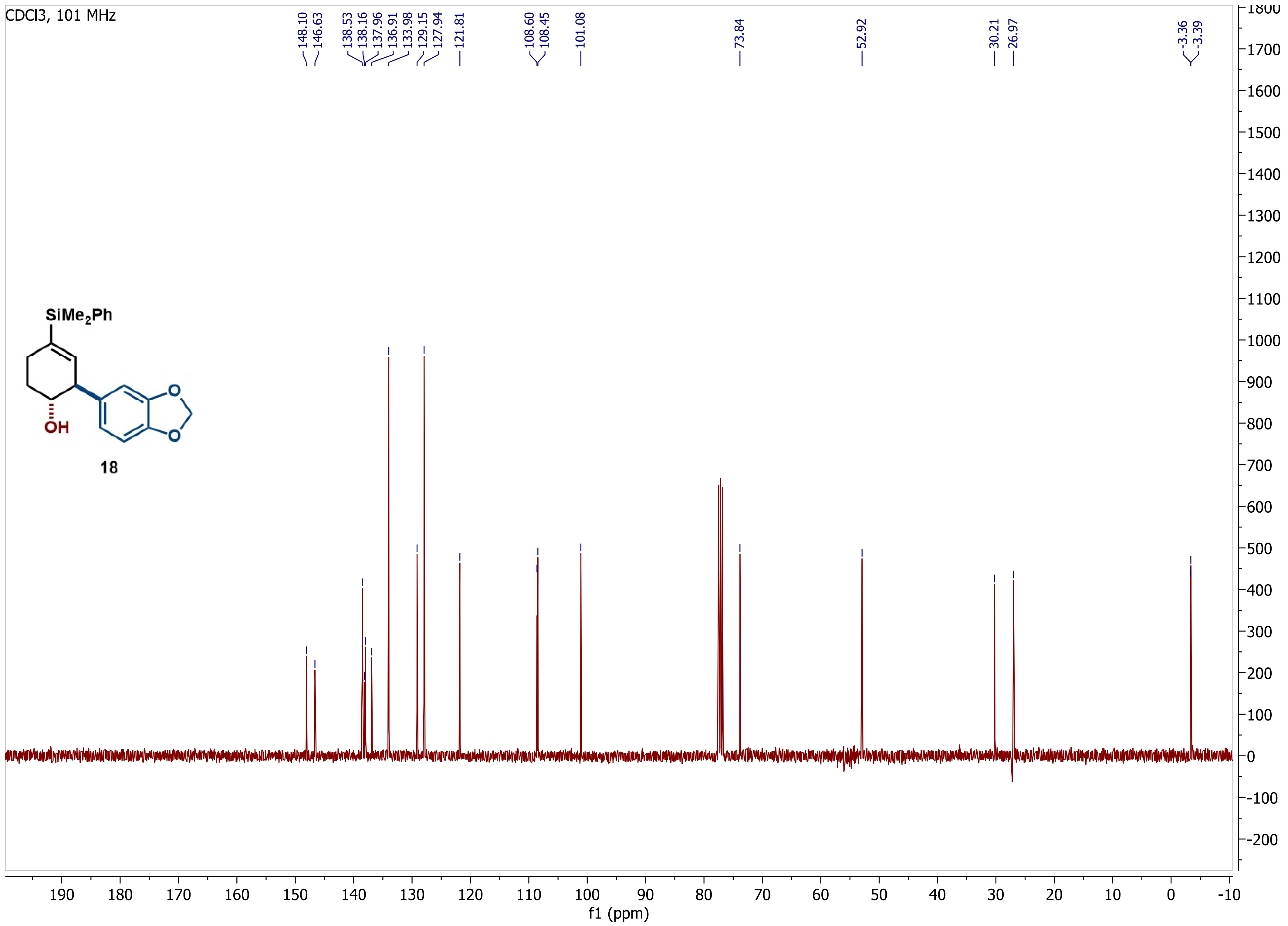
CDCl3, 101 MHz
148.10
146.63
138.53
138.16
137.96
136.91
133.98
129.15
127.94
121.81
108.60
108.45
101.08
73.84
52.92
30.21
26.97
-3.36
-3.39
SiMe2Ph
OH
18
f1 (ppm)

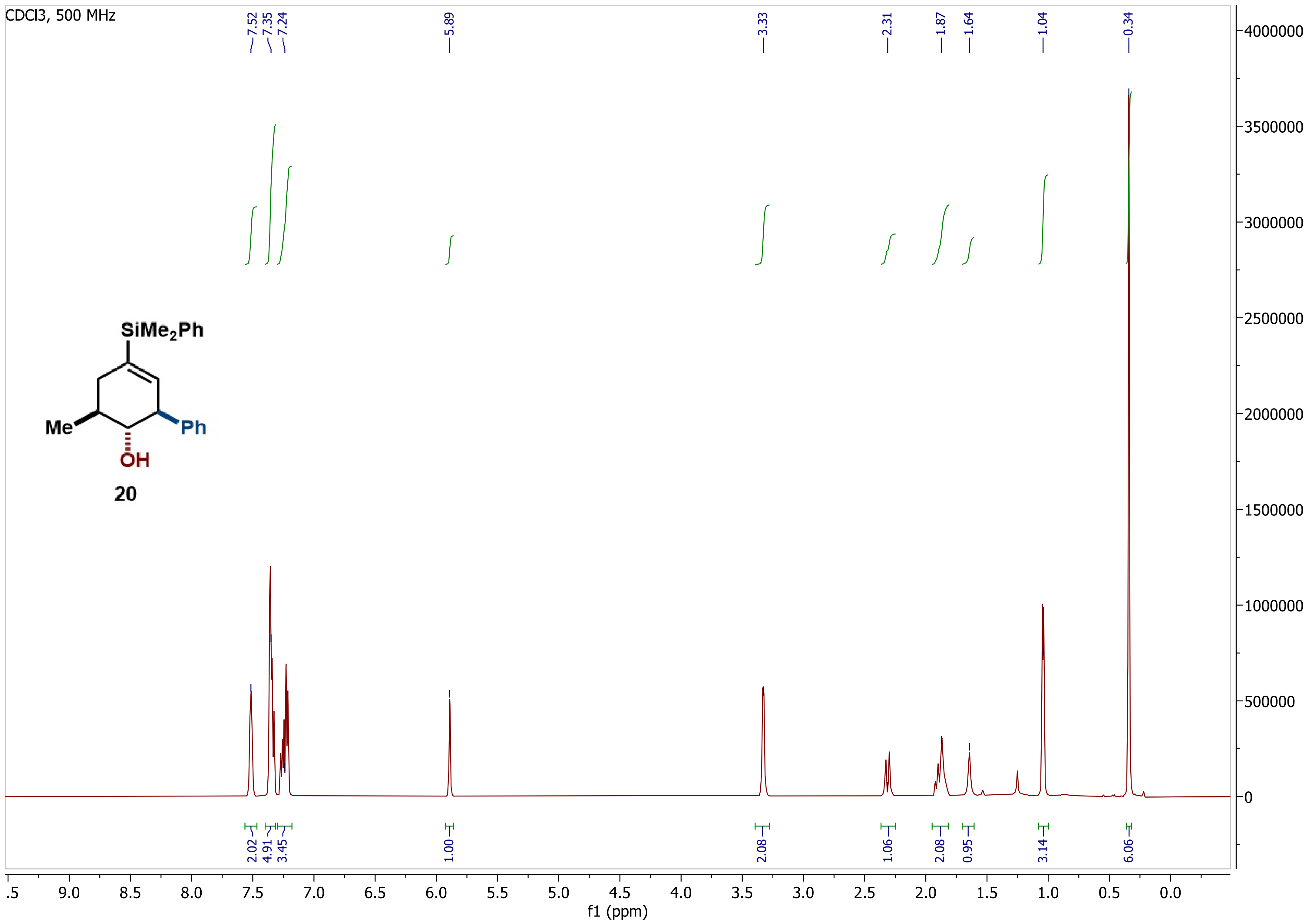

CDCl3, 500 MHz
7.52
7.35
7.24
5.89
3.33
2.31
1.87
1.64
1.04
0.34
SiMe2Ph
Me
Ph
OH
20
2.02
4.91
3.45
1.00
2.08
1.06
2.08
0.95
3.14
6.06
4000000
3500000
3000000
2500000
2000000
1500000
1000000
500000
0
.5
9.0
8.5
8.0
7.5
7.0
6.5
6.0
5.5
5.0
4.5
4.0
3.5
3.0
2.5
2.0
1.5
1.0
0.5
0.0
f1 (ppm)

CDCl3, 126 MHz

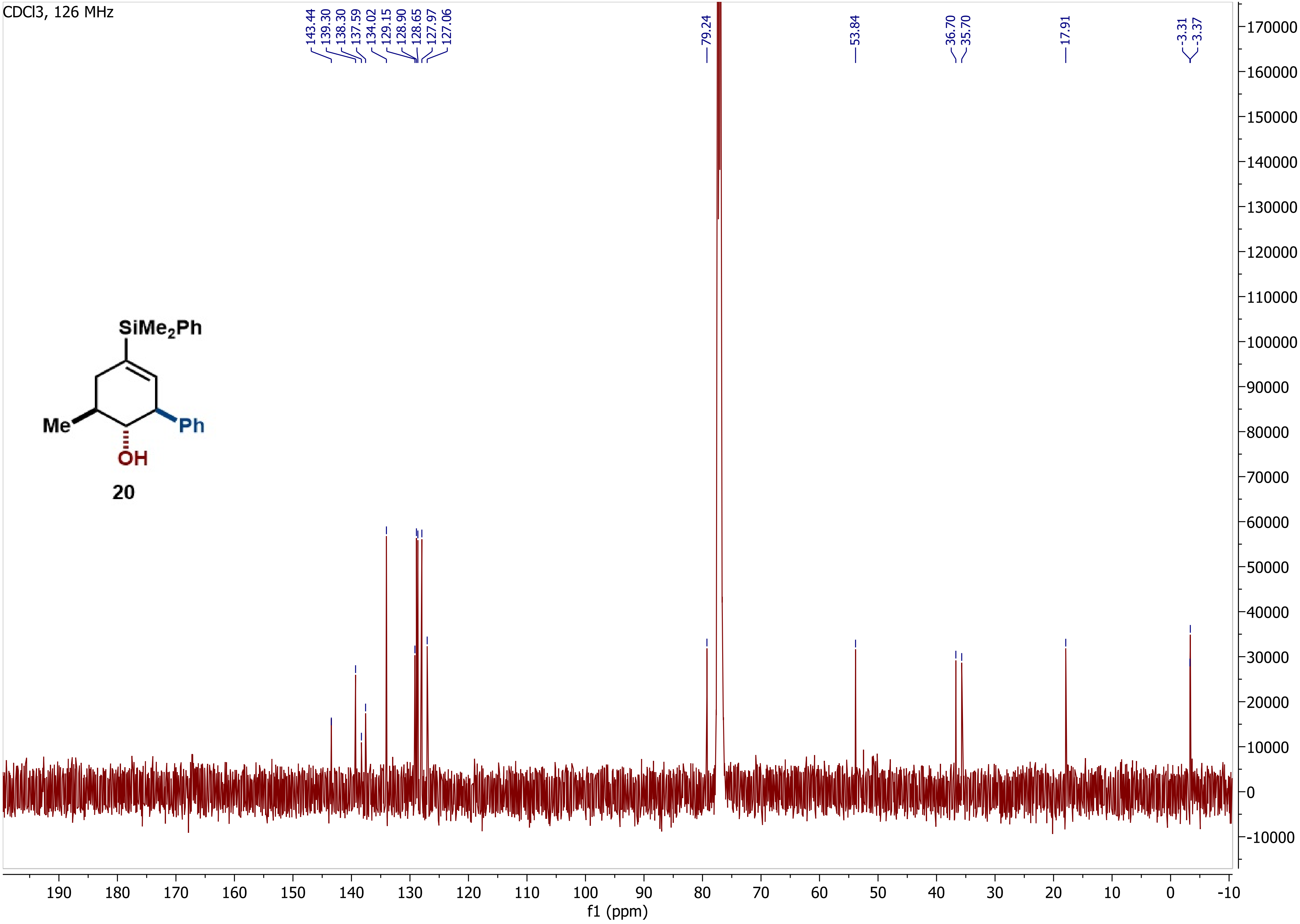

20

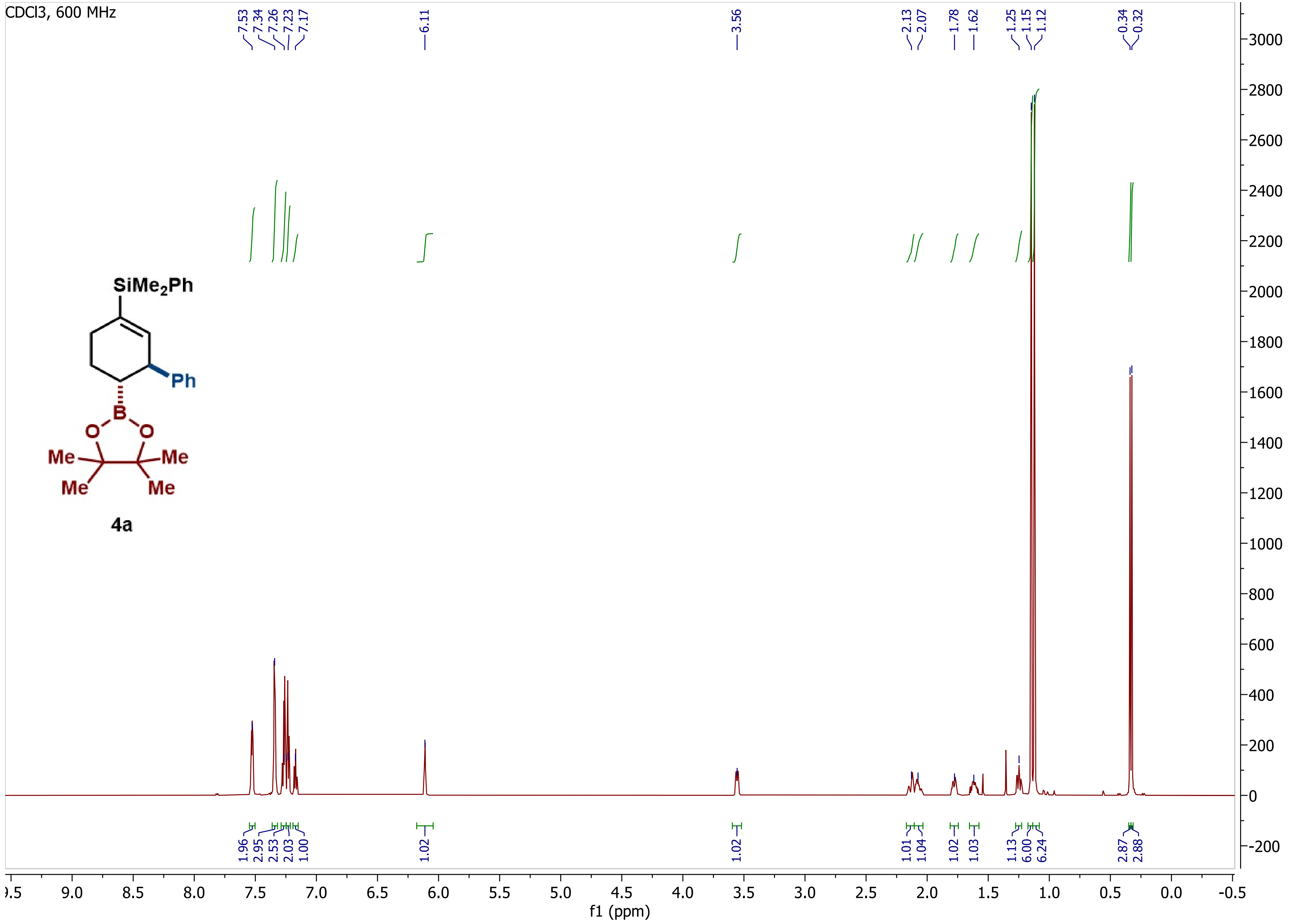
CDCl3, 600 MHz
7.53
7.34
7.26
7.23
7.17
6.11
3.56
2.13
2.07
1.78
1.62
1.25
1.15
1.12
0.34
0.32
SiMe2Ph
Ph
B
O
O
Me
Me
Me
Me
4a
1.96
2.95
2.53
2.03
1.00
1.02
1.02
1.01
1.04
1.02
1.03
1.13
6.00
6.24
2.87
2.88
f1 (ppm)

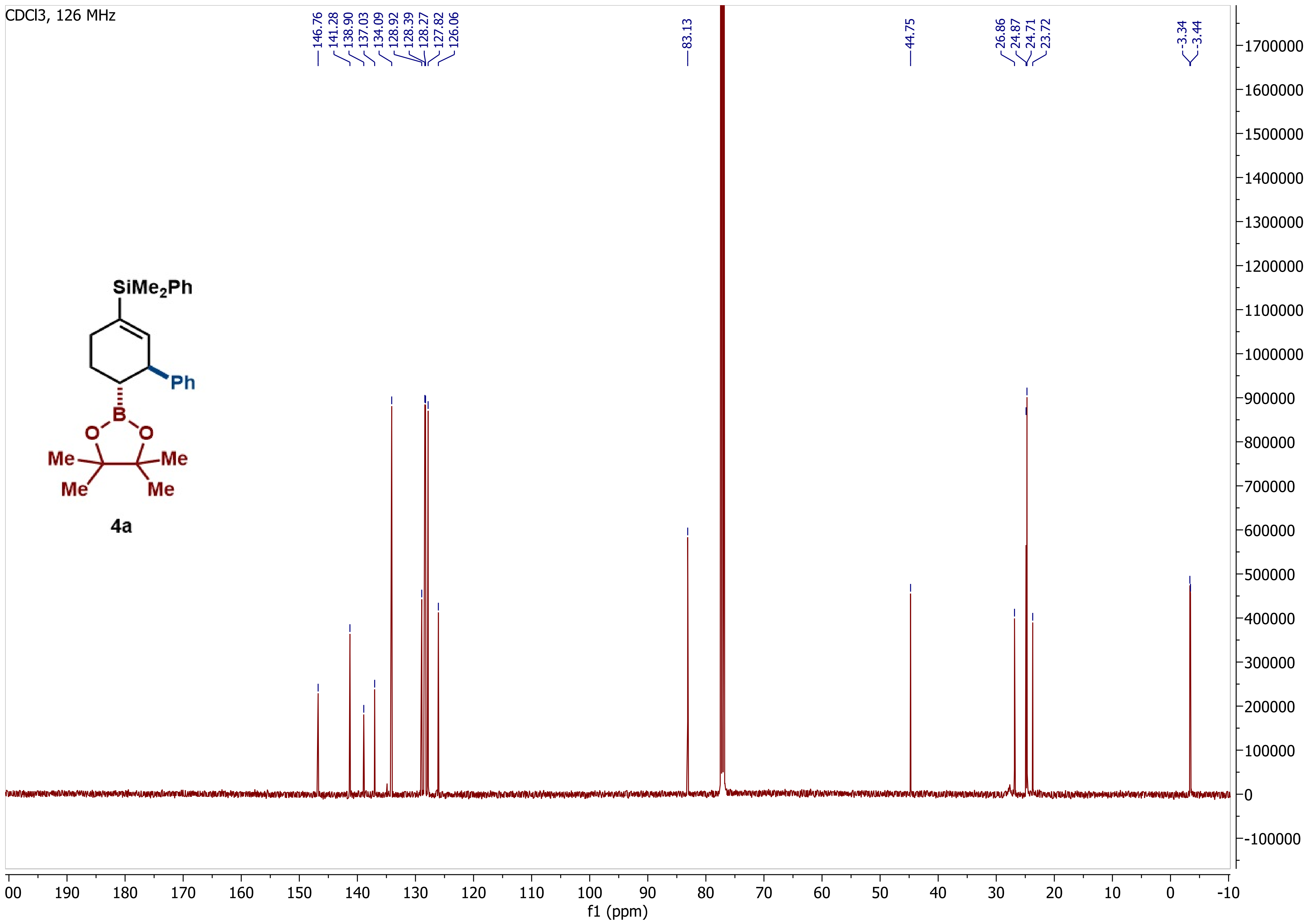

CDCl3, 126 MHz
146.76
141.28
138.90
137.03
134.09
128.92
128.39
128.27
127.82
126.06
83.13
44.75
26.86
24.87
24.71
23.72
-3.34
-3.44
SiMe2Ph
Ph
B
O
O
Me
Me
Me
Me
4a
f1 (ppm)

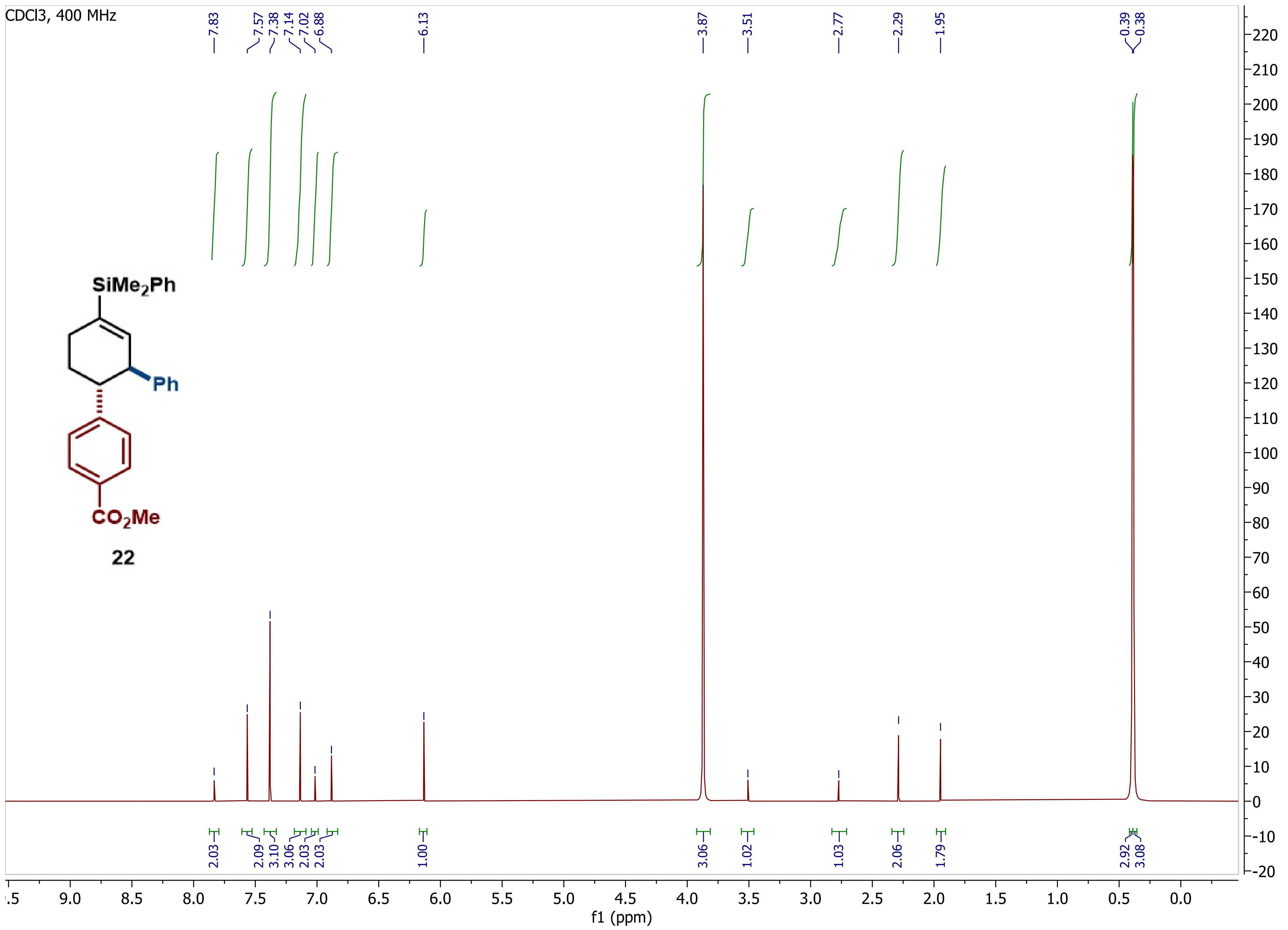

CDCl3, 400 MHz
7.83
7.57
7.38
7.14
7.02
6.88
6.13
3.87
3.51
2.77
2.29
1.95
0.39
0.38
SiMe2Ph
Ph
CO2Me
22
2.03
2.09
3.10
3.06
2.03
2.03
1.00
3.06
1.02
1.03
2.06
1.79
2.92
3.08
f1 (ppm)

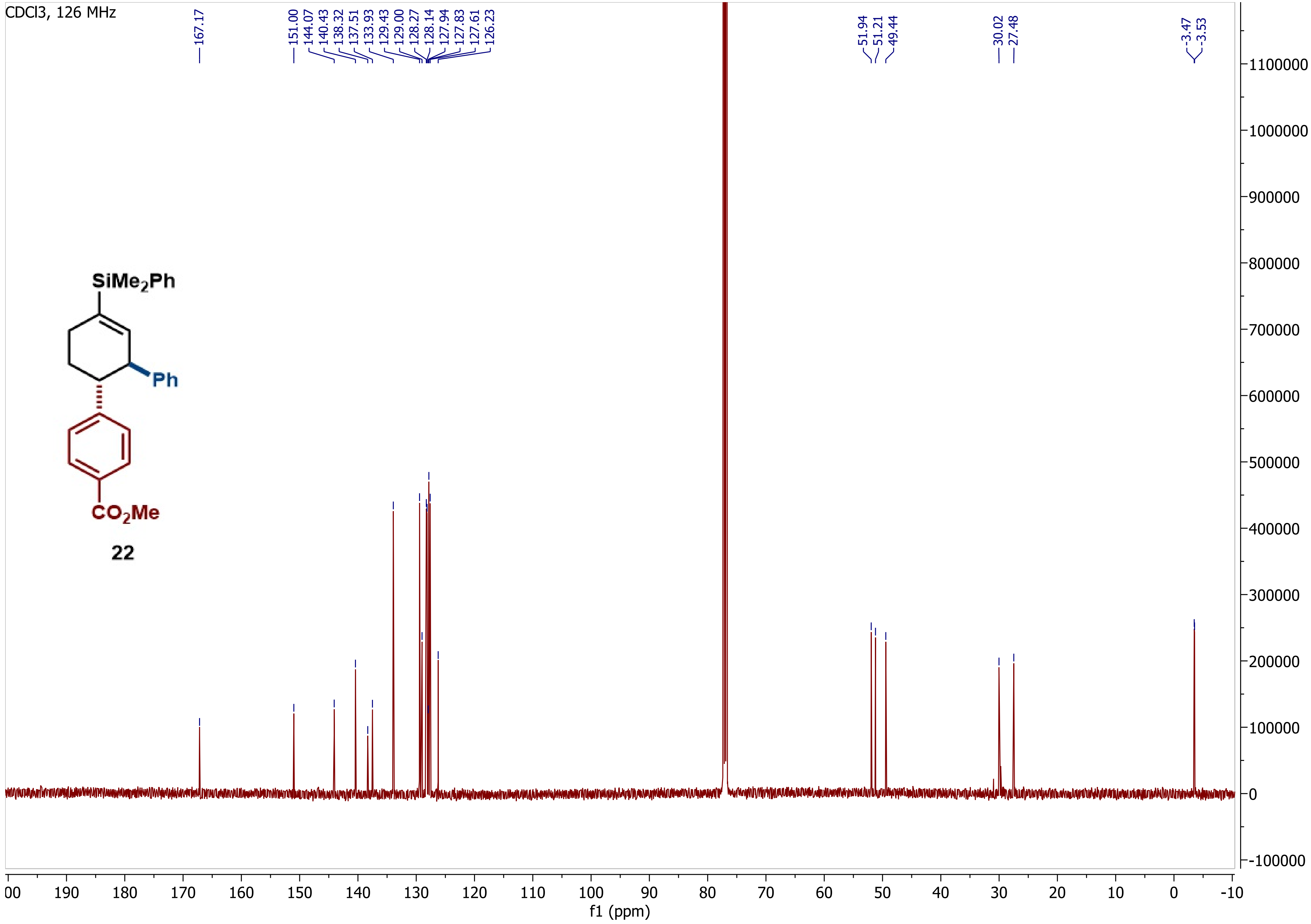

CDCl3, 126 MHz
167.17
151.00
144.07
140.43
138.32
137.51
133.93
129.43
129.00
128.27
128.14
127.94
127.83
127.61
126.23
51.94
51.21
49.44
30.02
27.48
-3.47
-3.53
SiMe2Ph
Ph
CO2Me
22
f1 (ppm)

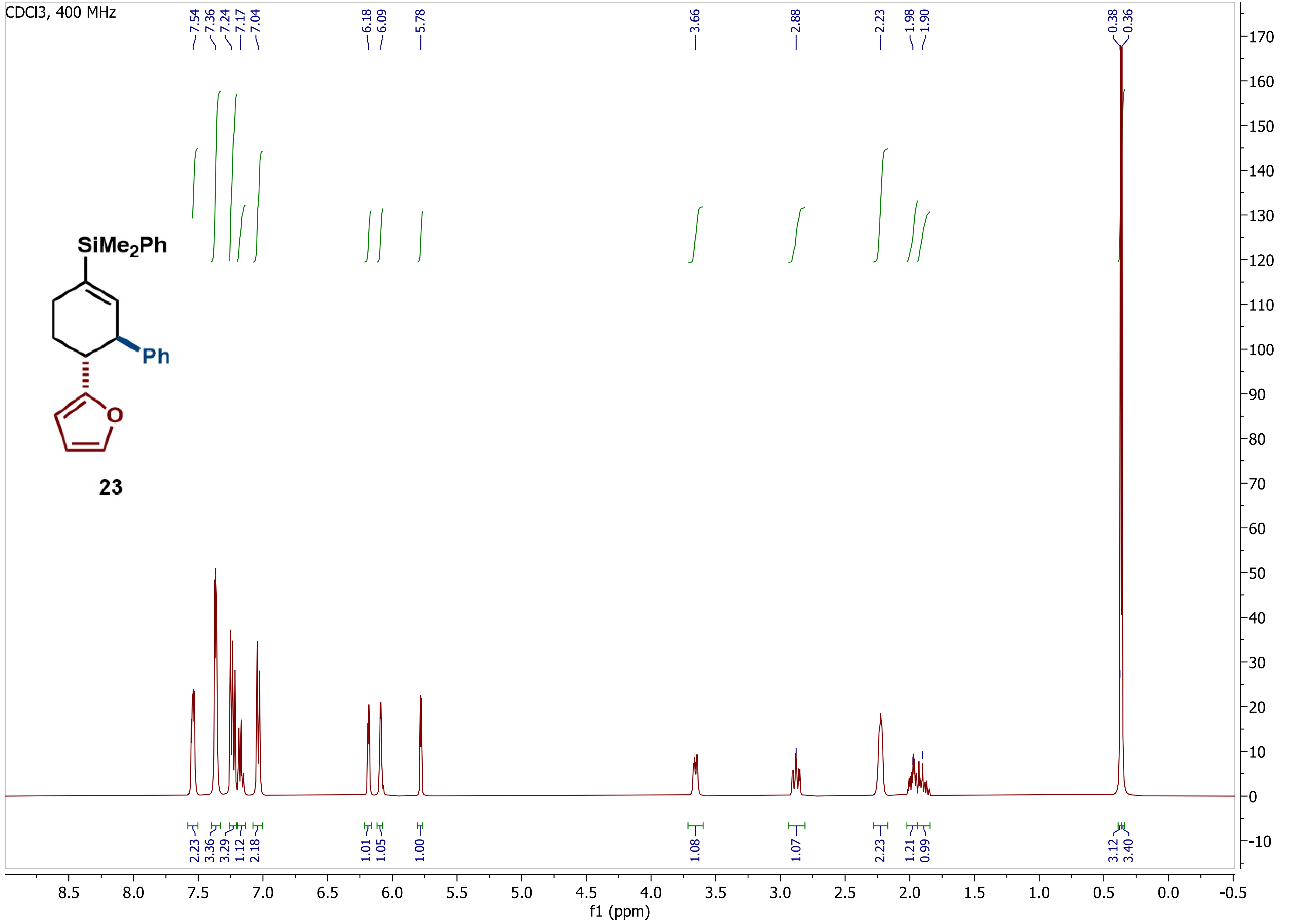

CDCl3, 400 MHz
7.54
7.36
7.24
7.17
7.04
6.18
6.09
5.78
3.66
2.88
2.23
1.98
1.90
0.38
0.36
SiMe2Ph
Ph
O
23
2.23
3.36
3.29
1.12
2.18
1.01
1.05
1.00
1.08
1.07
2.23
1.21
0.99
3.12
3.40
f1 (ppm)

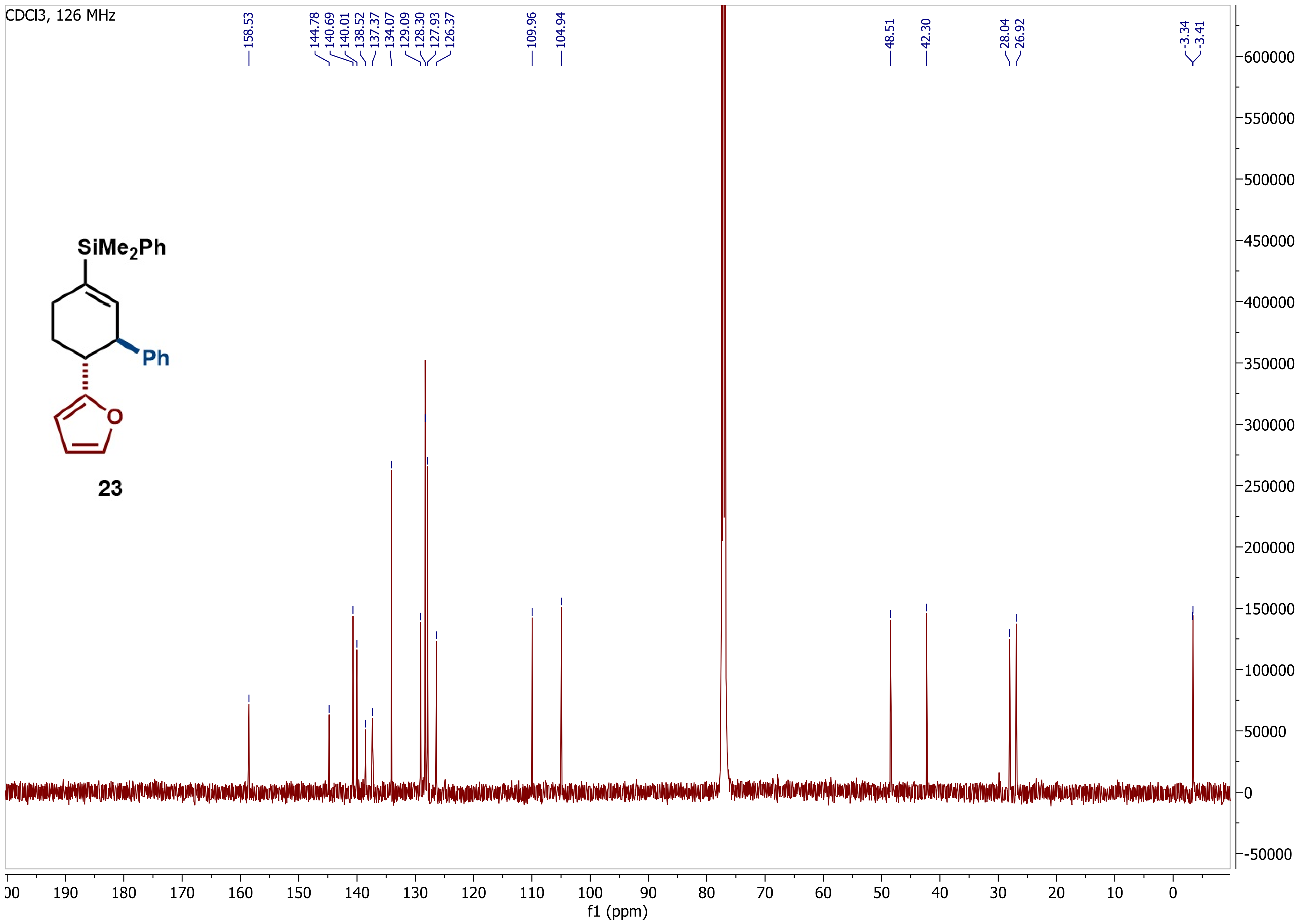

CDCl3, 126 MHz
158.53
144.78
140.69
140.01
138.52
137.37
134.07
129.09
128.30
127.93
126.37
109.96
104.94
48.51
42.30
28.04
26.92
-3.34
-3.41
SiMe2Ph
Ph
O
23
f1 (ppm)

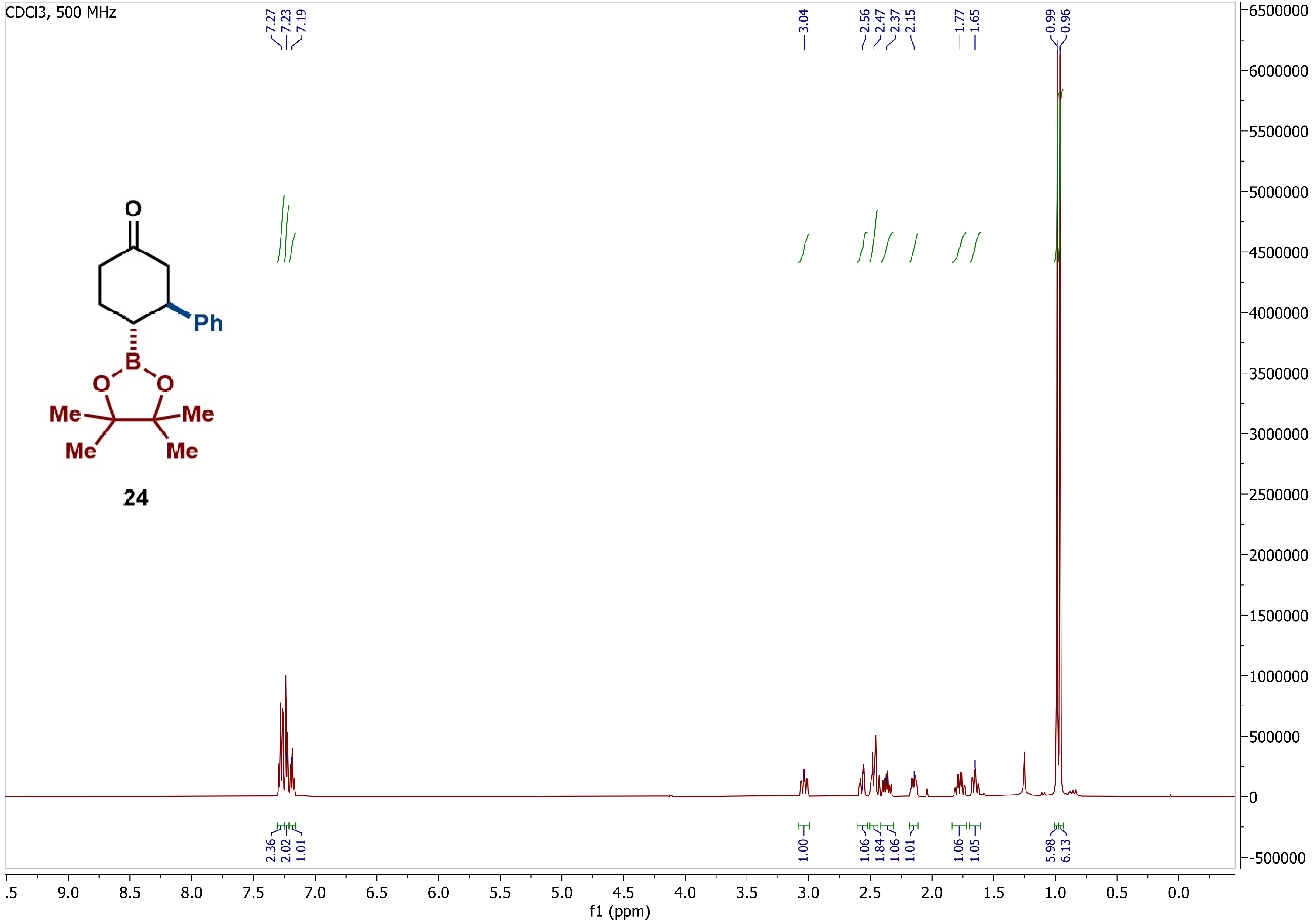

CDCl3, 500 MHz
7.27
7.23
7.19
3.04
2.56
2.47
2.37
2.15
1.77
1.65
0.99
0.96
Ph
O
B
Me
2.36
2.02
1.01
1.00
1.06
1.84
1.06
1.01
1.06
1.05
5.98
6.13
f1 (ppm)

24

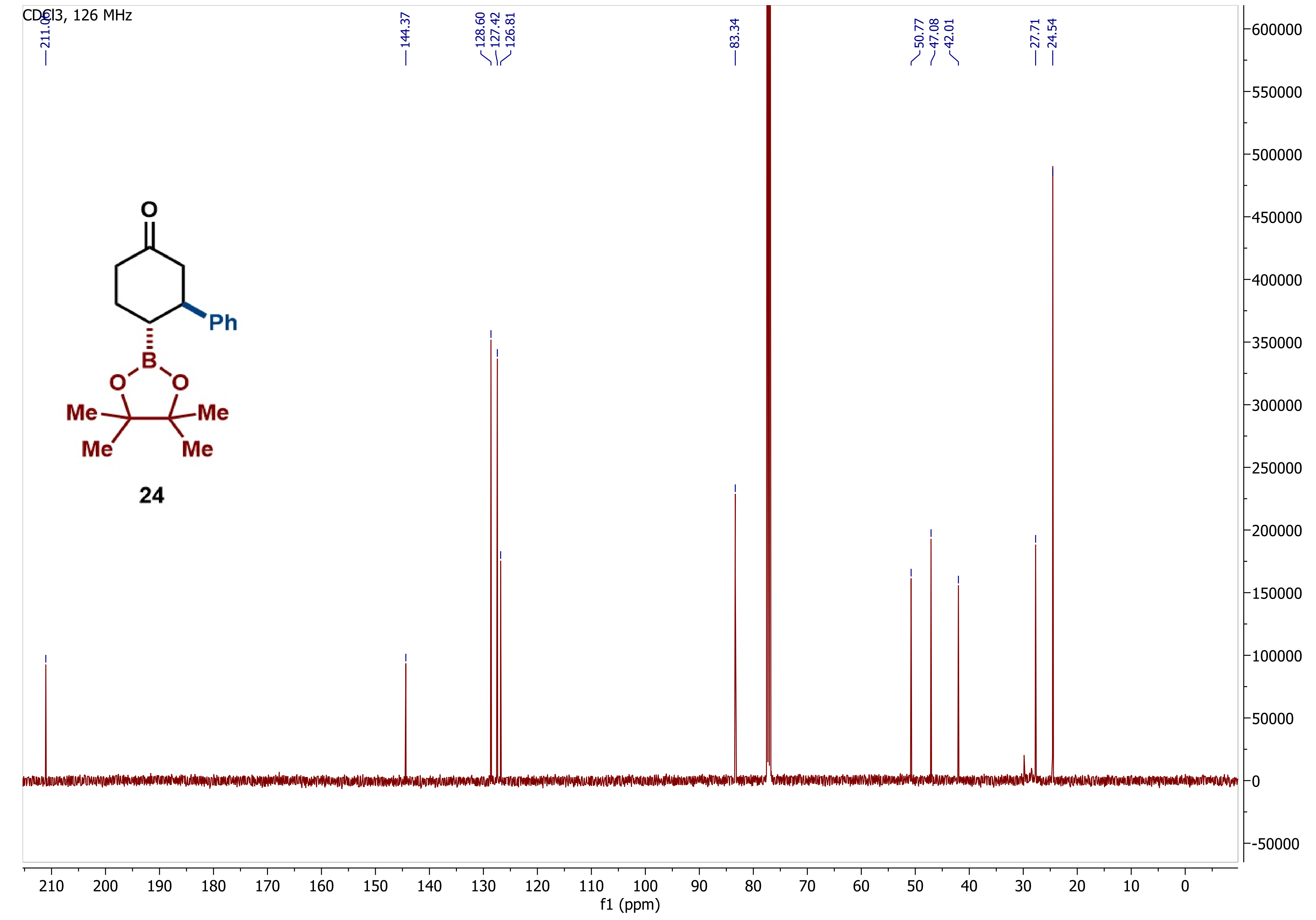

CDCl3, 126 MHz
211.0
144.37
128.60
127.42
126.81
83.34
50.77
47.08
42.01
27.71
24.54
O
Ph
B
O
O
Me
Me
Me
Me
24
600000
550000
500000
450000
400000
350000
300000
250000
200000
150000
100000
50000
0
-50000
210 200 190 180 170 160 150 140 130 120 110 100 90 80 70 60 50 40 30 20 10 0
f1 (ppm)

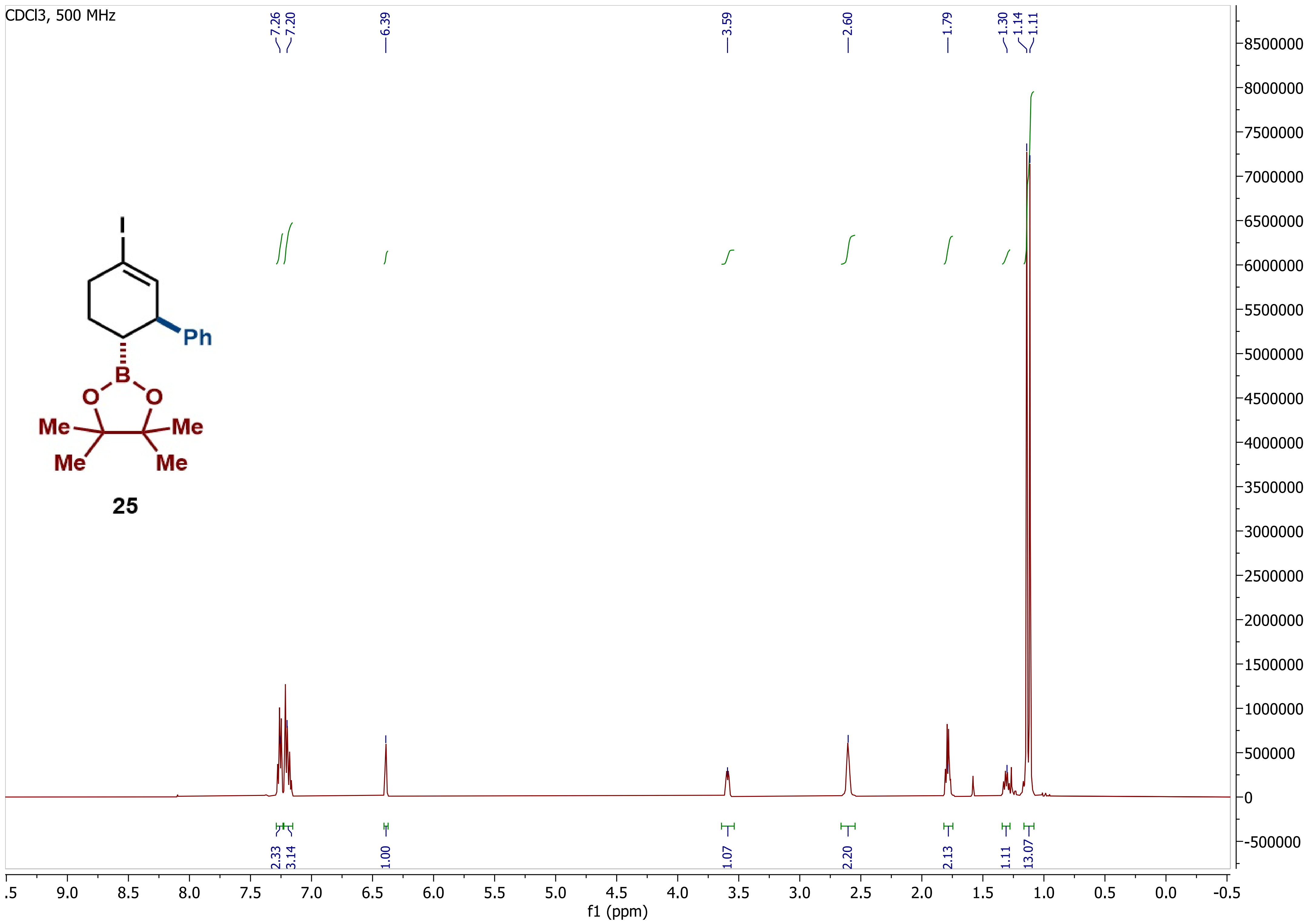

CDCl3, 500 MHz
7.26
7.20
6.39
3.59
2.60
1.79
1.30
1.14
1.11
Ph
B
O
O
Me
Me
Me
Me
25
2.33
3.14
1.00
1.07
2.20
2.13
1.11
13.07
f1 (ppm)

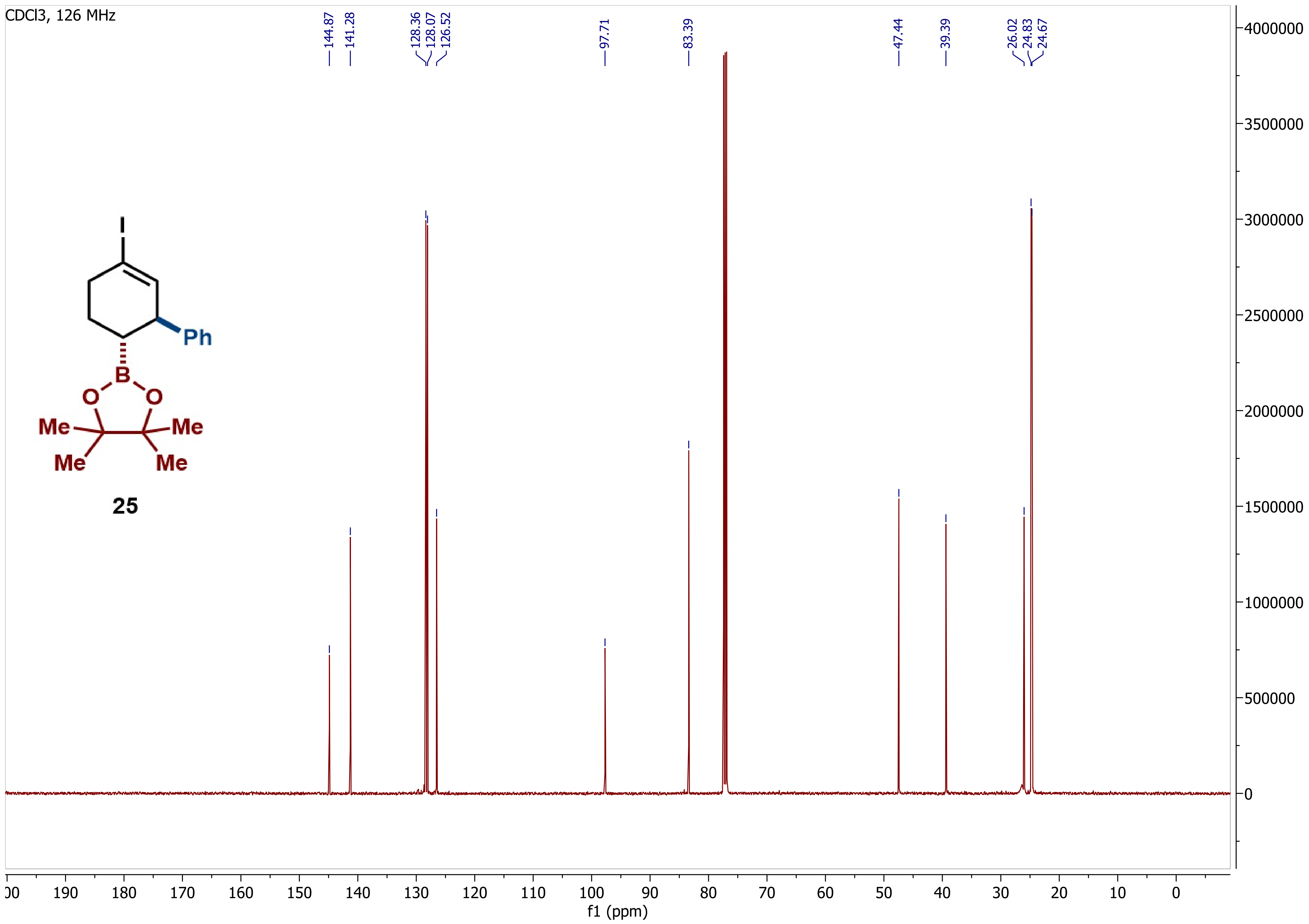

CDCl3, 126 MHz
144.87
141.28
128.36
128.07
126.52
97.71
83.39
47.44
39.39
26.02
24.83
24.67
I
Ph
B
O
O
Me
Me
Me
Me
25
4000000
3500000
3000000
2500000
2000000
1500000
1000000
500000
0
190
180
170
160
150
140
130
120
110
100
90
80
70
60
50
40
30
20
10
0
f1 (ppm)

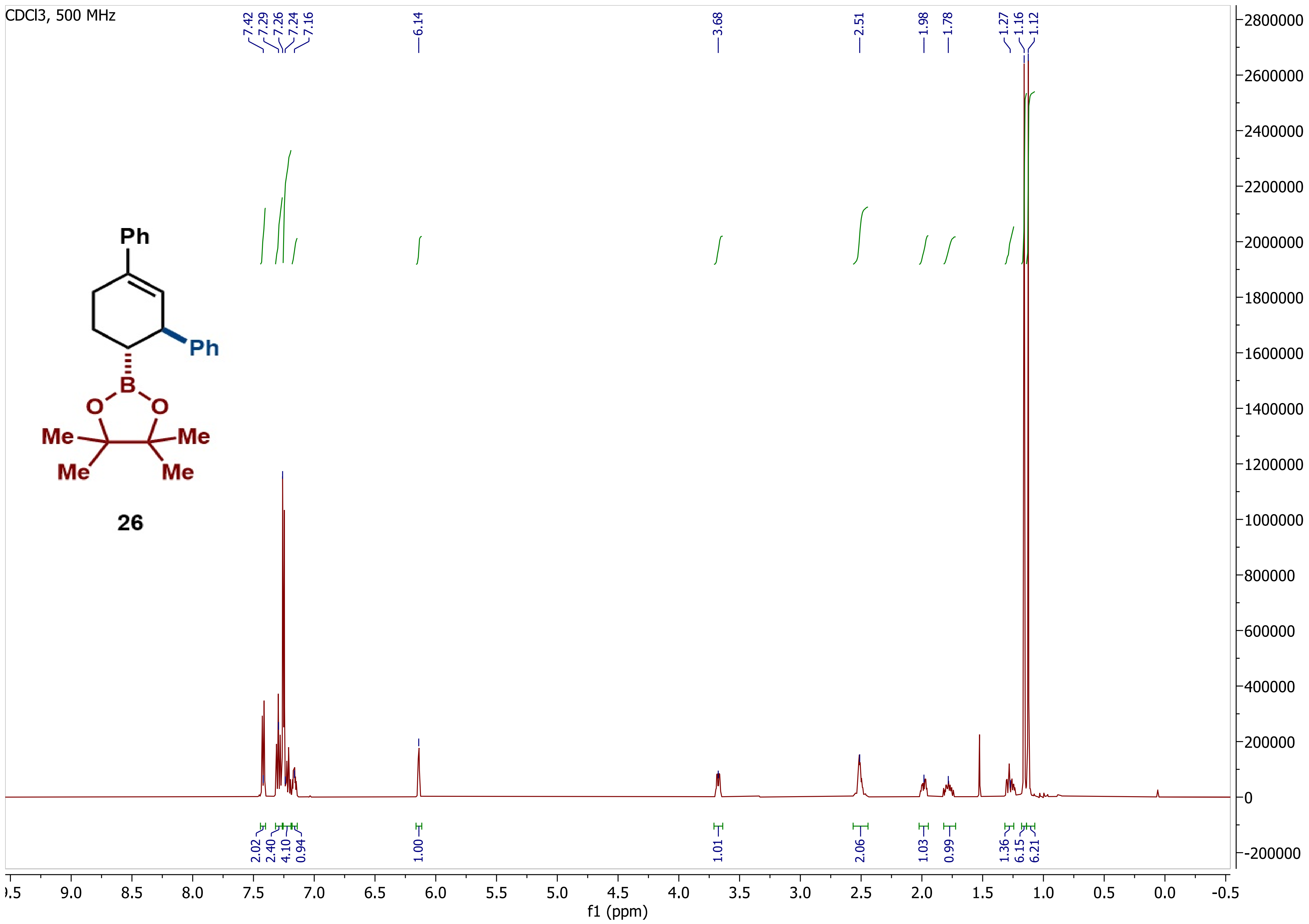
CDCl3, 500 MHz
7.42
7.29
7.26
7.24
7.16
6.14
3.68
2.51
1.98
1.78
1.27
1.16
1.12
Ph
Ph
B
O
O
Me
Me
Me
Me
26
2.02
2.40
4.10
0.94
1.00
1.01
2.06
1.03
0.99
1.36
6.15
6.21
f1 (ppm)

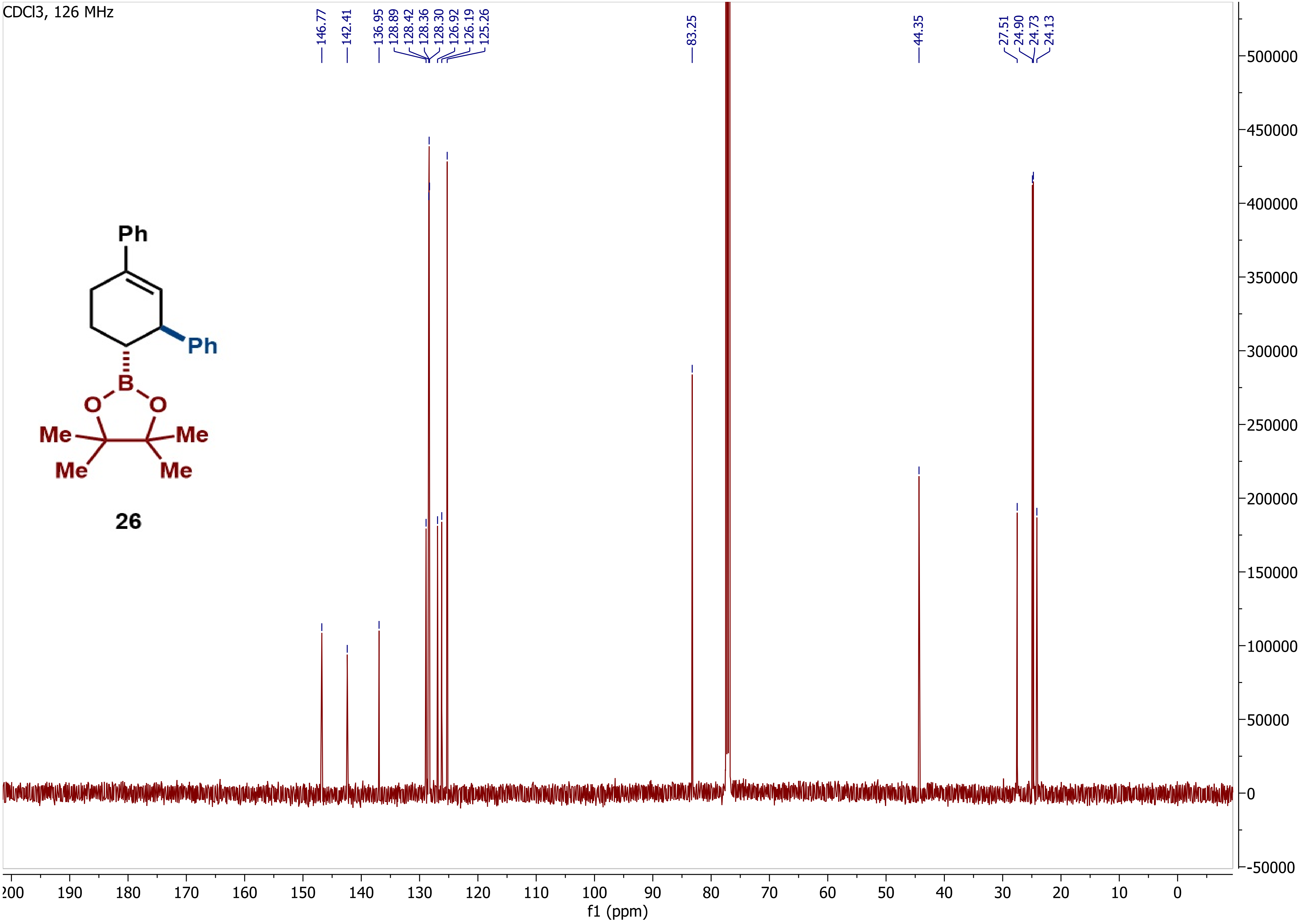
CDCl3, 126 MHz
146.77
142.41
136.95
128.89
128.42
128.36
128.30
126.92
126.19
125.26
83.25
44.35
27.51
24.90
24.73
24.13
Ph
Ph
B
O
O
Me
Me
Me
Me
26
f1 (ppm)

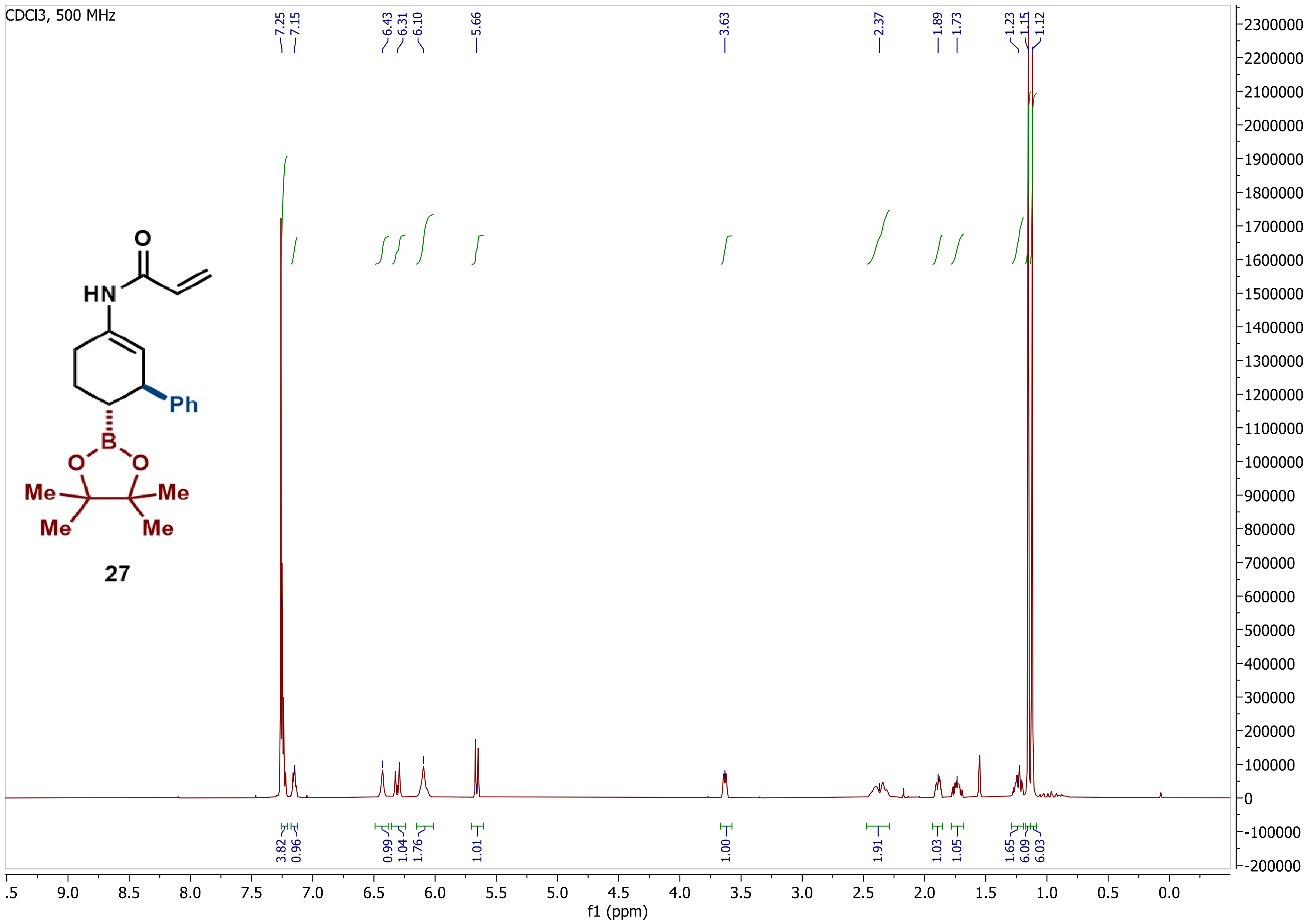

CDCl3, 500 MHz
7.25
7.15
6.43
6.31
6.10
5.66
3.63
2.37
1.89
1.73
1.23
1.15
1.12
HN
O
Ph
B
O
O
Me
Me
Me
Me
27
3.82
0.96
0.99
1.04
1.76
1.01
1.00
1.91
1.03
1.05
1.65
6.09
6.03
f1 (ppm)

CDCl3, 126 MHz

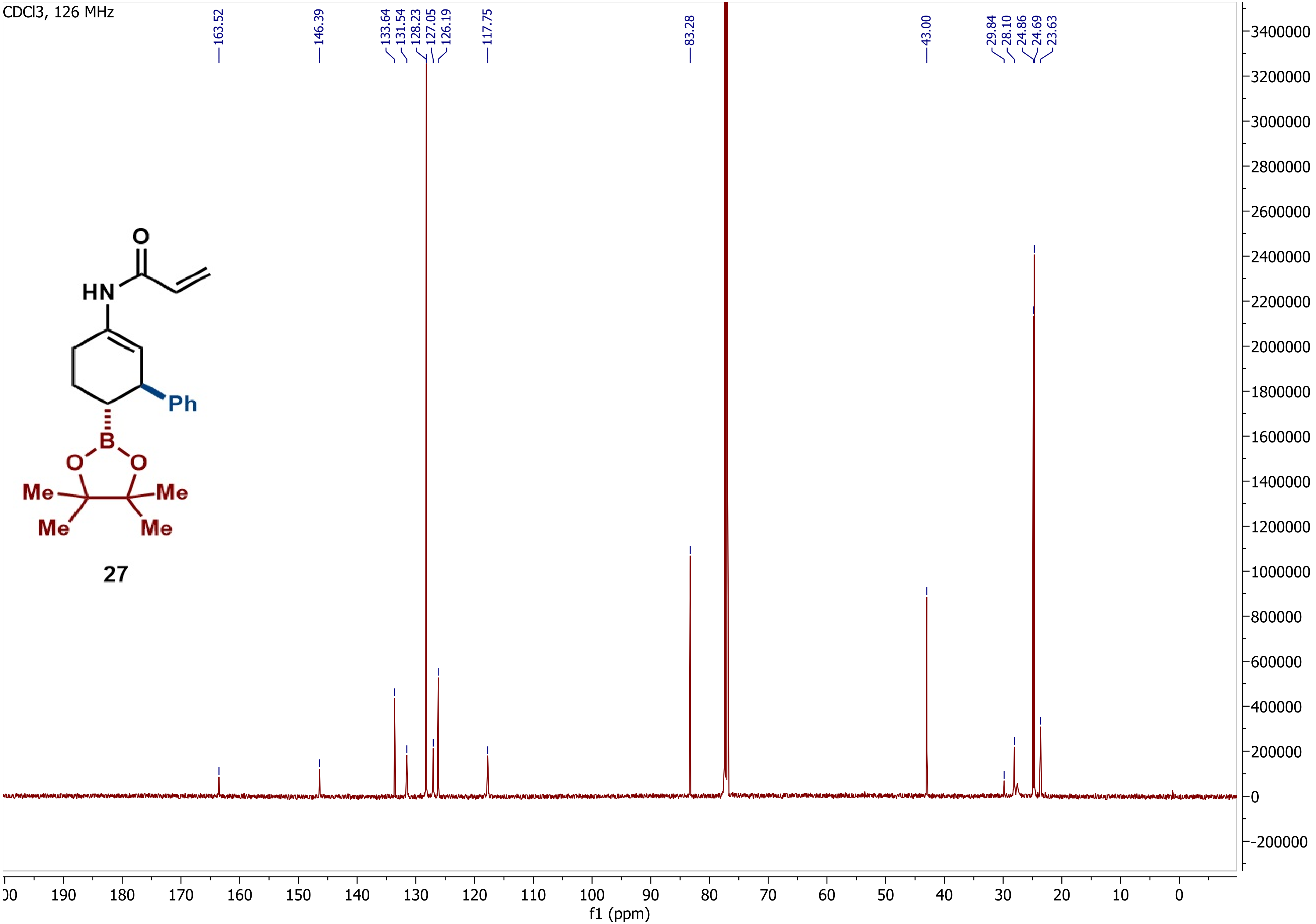

27

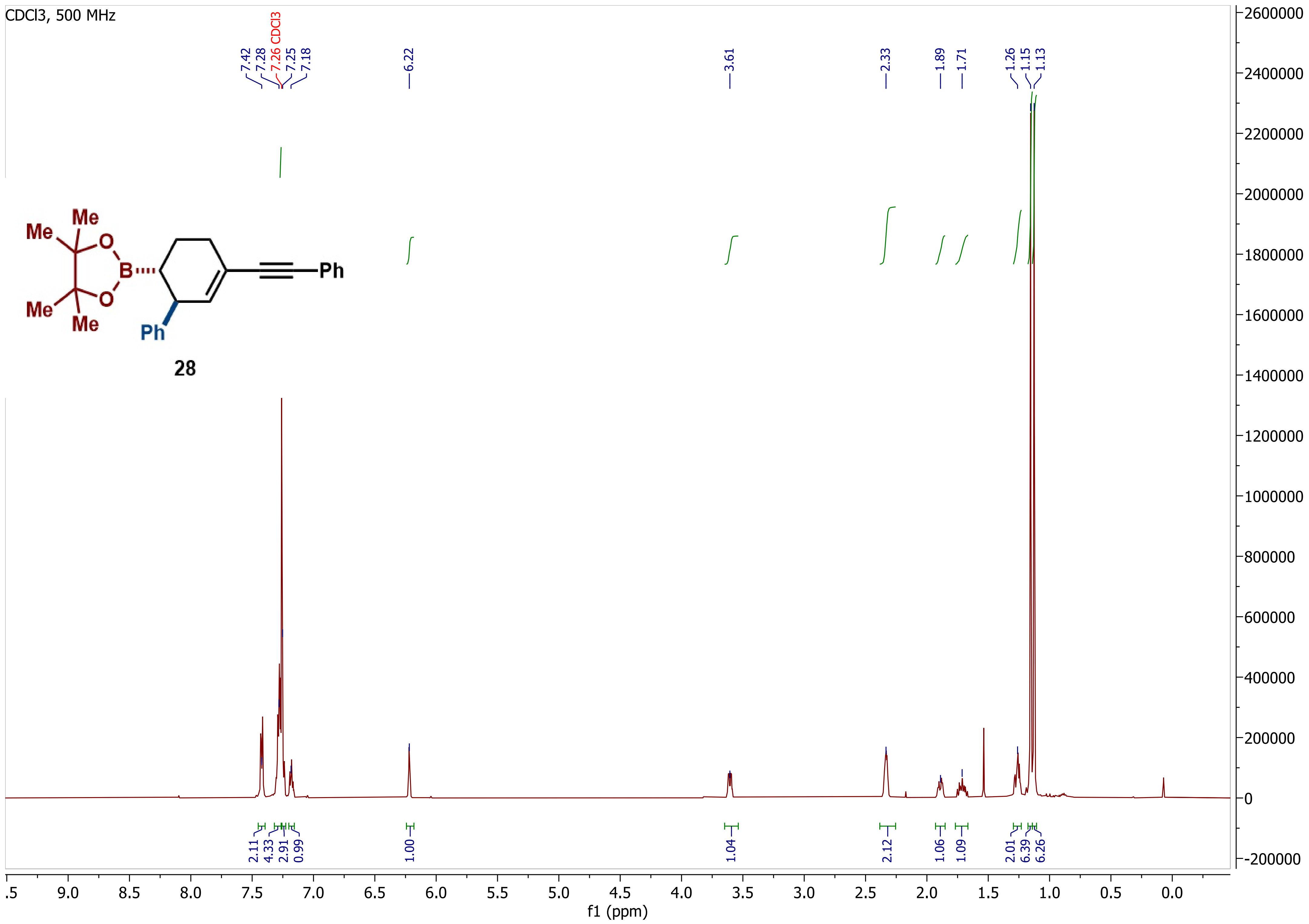

CDCl3, 500 MHz
7.42
7.28
7.26 CDCl3
7.25
7.18
6.22
3.61
2.33
1.89
1.71
1.26
1.15
1.13
Me
Me
O
B
O
Me
Me
Ph
Ph
28
2.11
4.33
2.91
0.99
1.00
1.04
2.12
1.06
1.09
2.01
6.39
6.26
f1 (ppm)

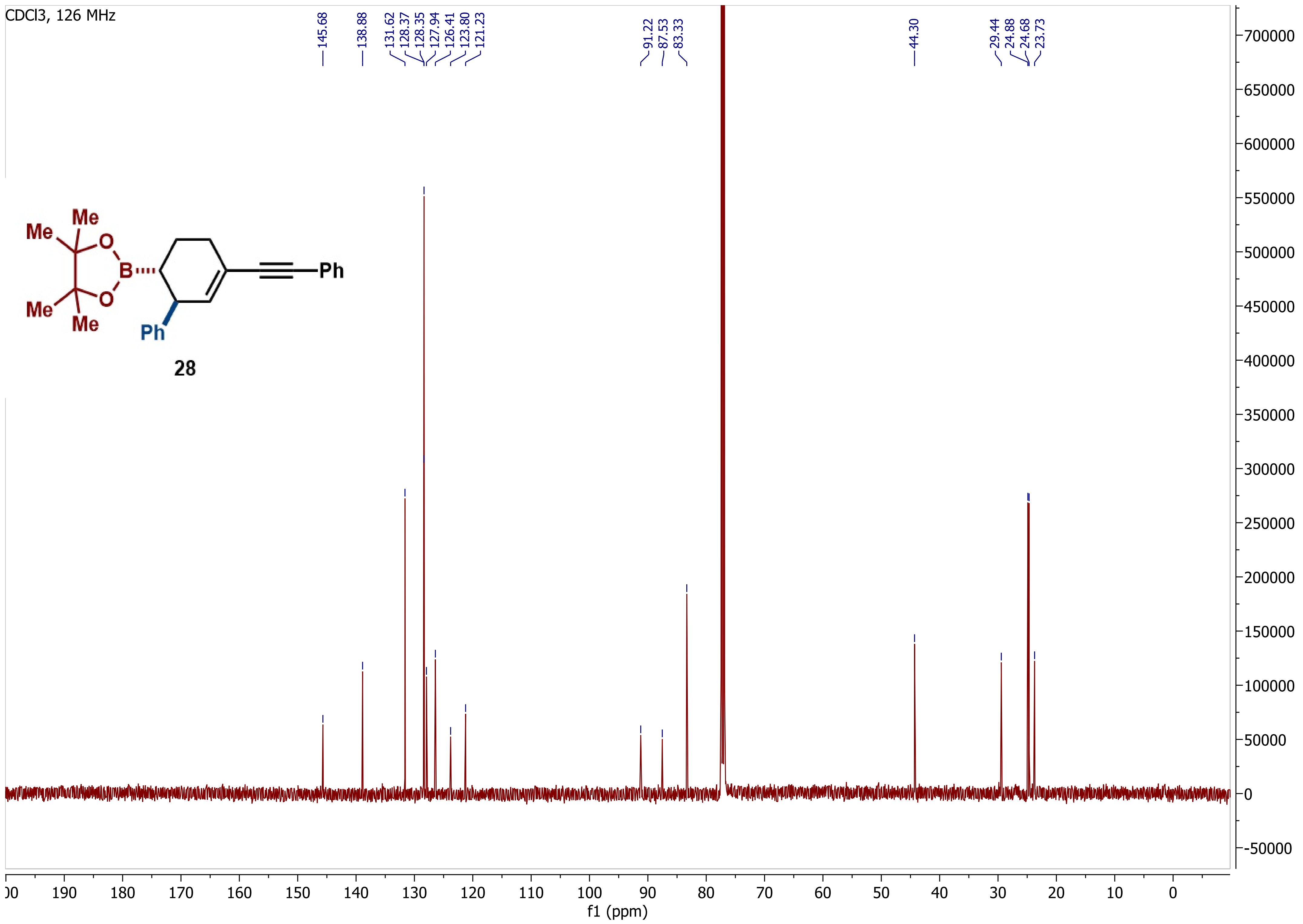
CDCl3, 126 MHz
145.68
138.88
131.62
128.37
128.35
127.94
126.41
123.80
121.23
91.22
87.53
83.33
44.30
29.44
24.88
24.68
23.73
Me
Me
Me
Me
O
O
B
Ph
Ph
28
700000
650000
600000
550000
500000
450000
400000
350000
300000
250000
200000
150000
100000
50000
0
-50000
190
180
170
160
150
140
130
120
110
100
90
80
70
60
50
40
30
20
10
0
f1 (ppm)

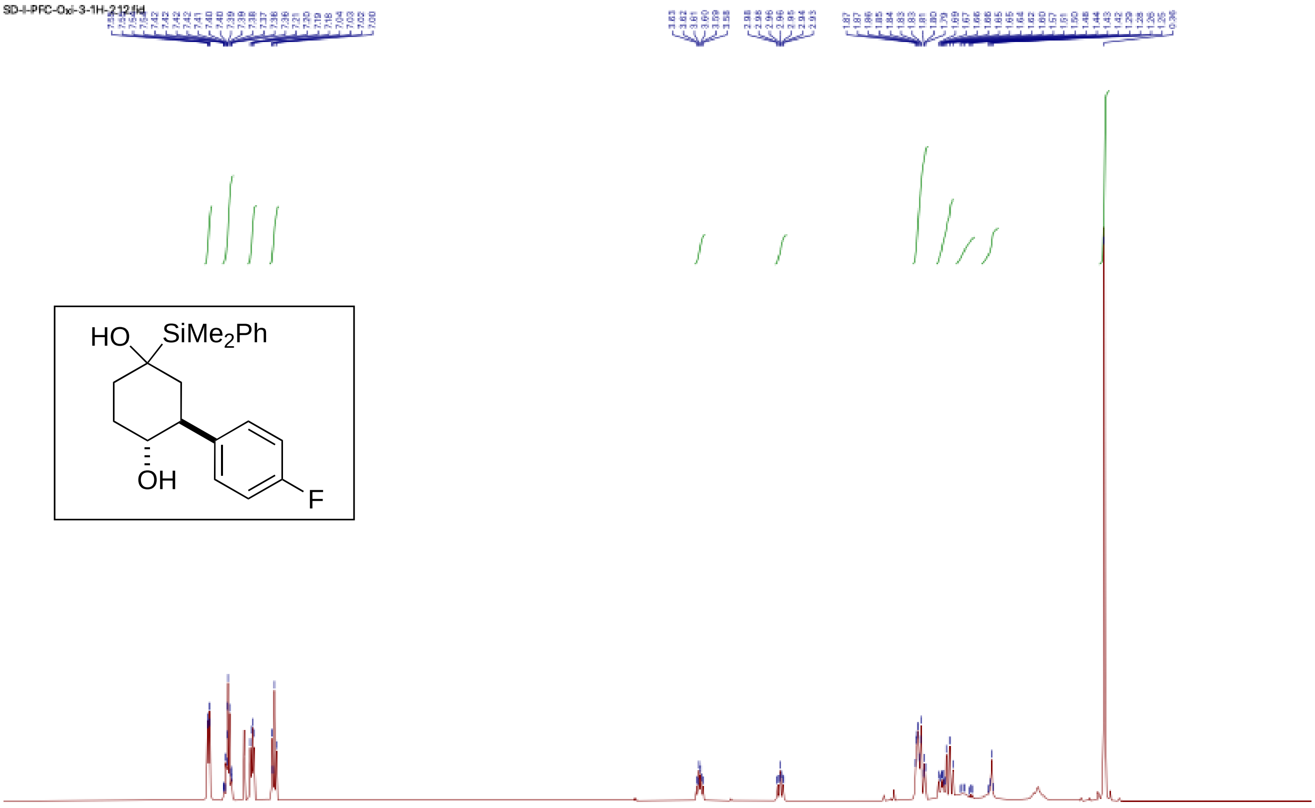
SD-I-PFC-Oxi-3-1H-2.12.fid
HO
SiMe2Ph
OH
F

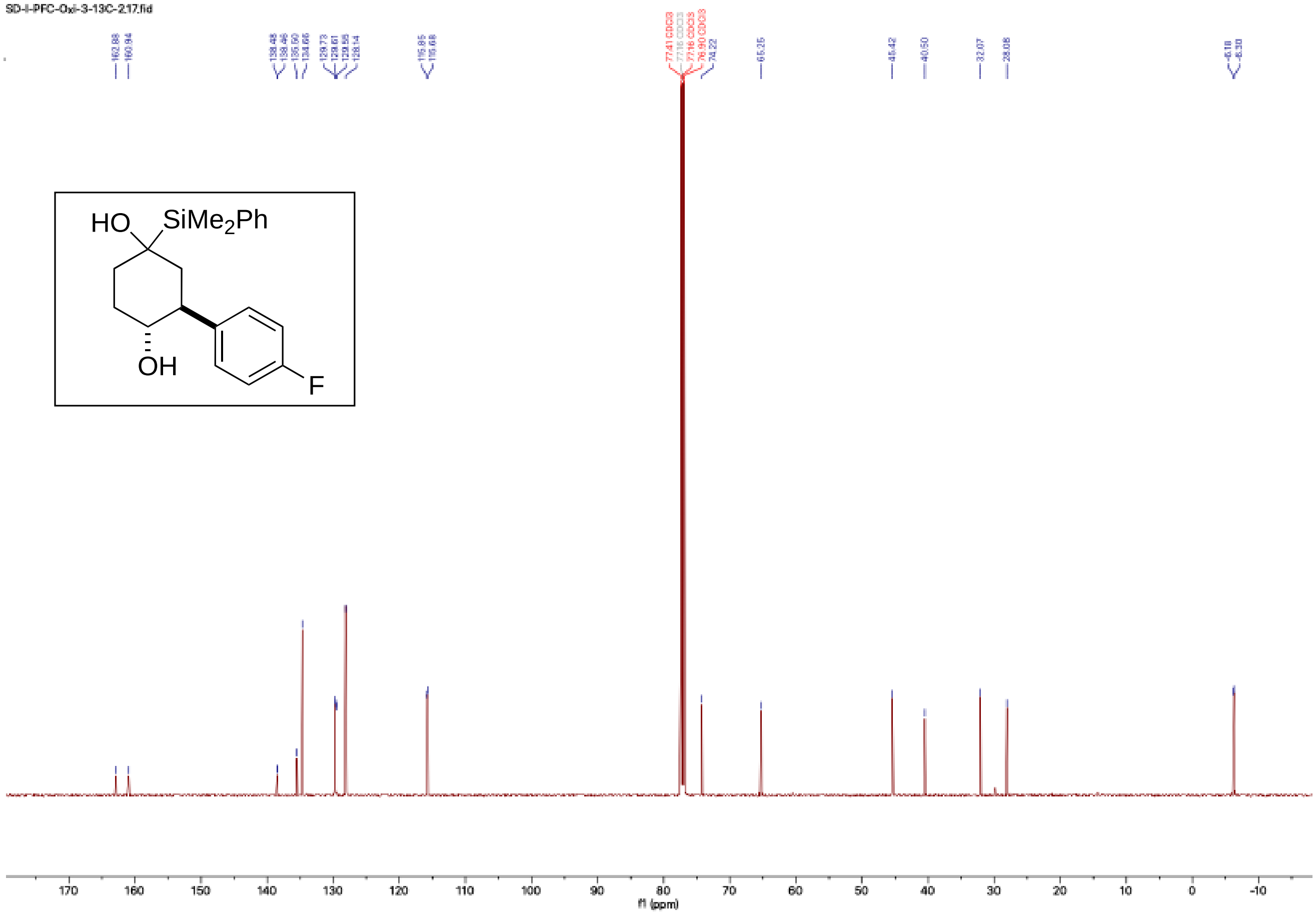
SD-I-PFC-Oxi-3-13C-2.17.fid
162.88
160.94
138.48
138.46
135.50
134.66
129.73
129.61
129.55
128.14
115.85
115.68
77.41 CDCl3
77.16 CDCl3
76.90 CDCl3
74.22
65.25
45.42
40.50
32.07
28.08
-6.18
-6.30
HO
SiMe2Ph
OH
F
170
160
150
140
130
120
110
100
90
80
70
60
50
40
30
20
10
0
-10
f1 (ppm)

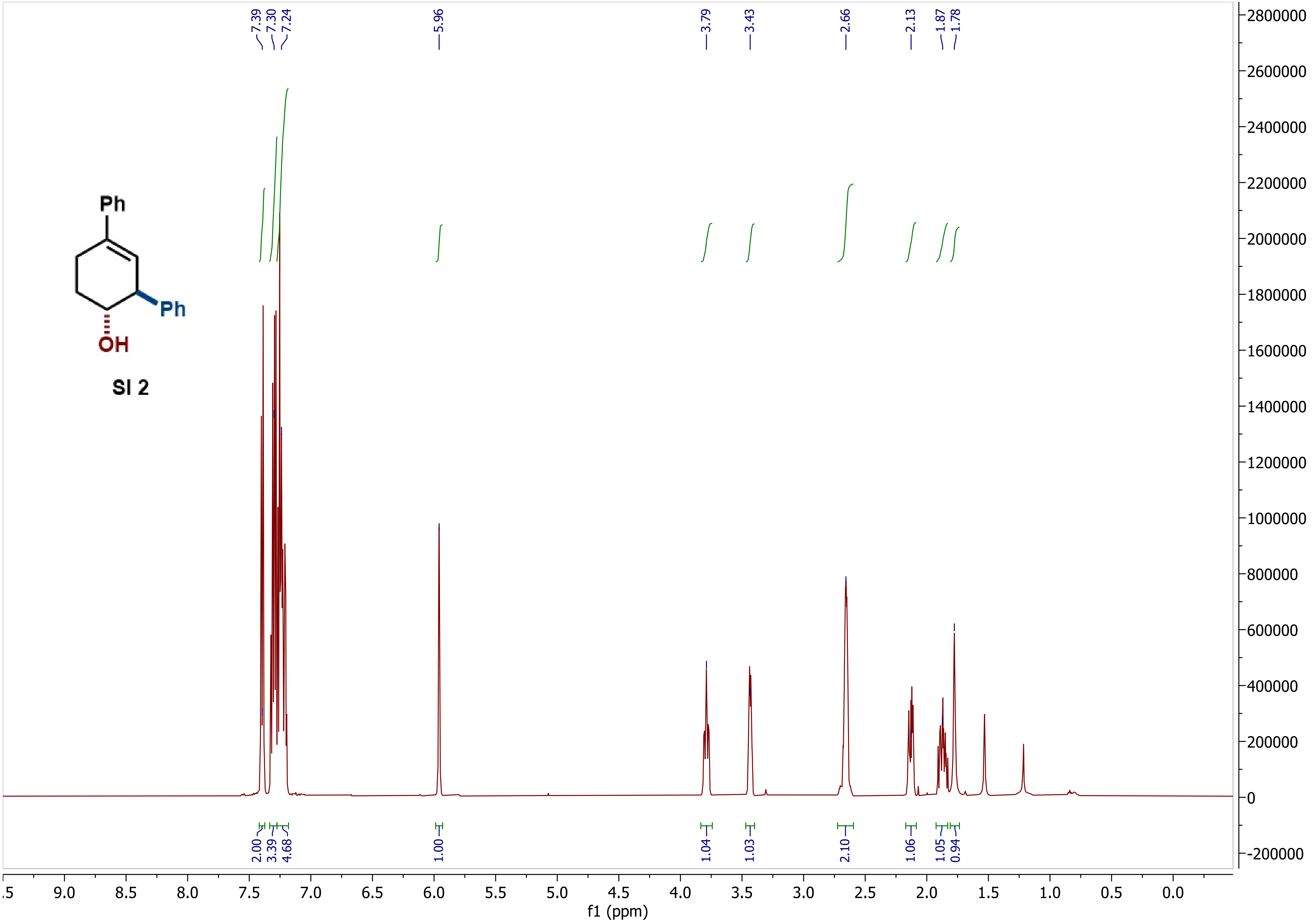

7.39
7.30
7.24
5.96
3.79
3.43
2.66
2.13
1.87
1.78
Ph
Ph
OH
2.00
3.39
4.68
1.00
1.04
1.03
2.10
1.06
1.05
0.94
f1 (ppm)

SI 2

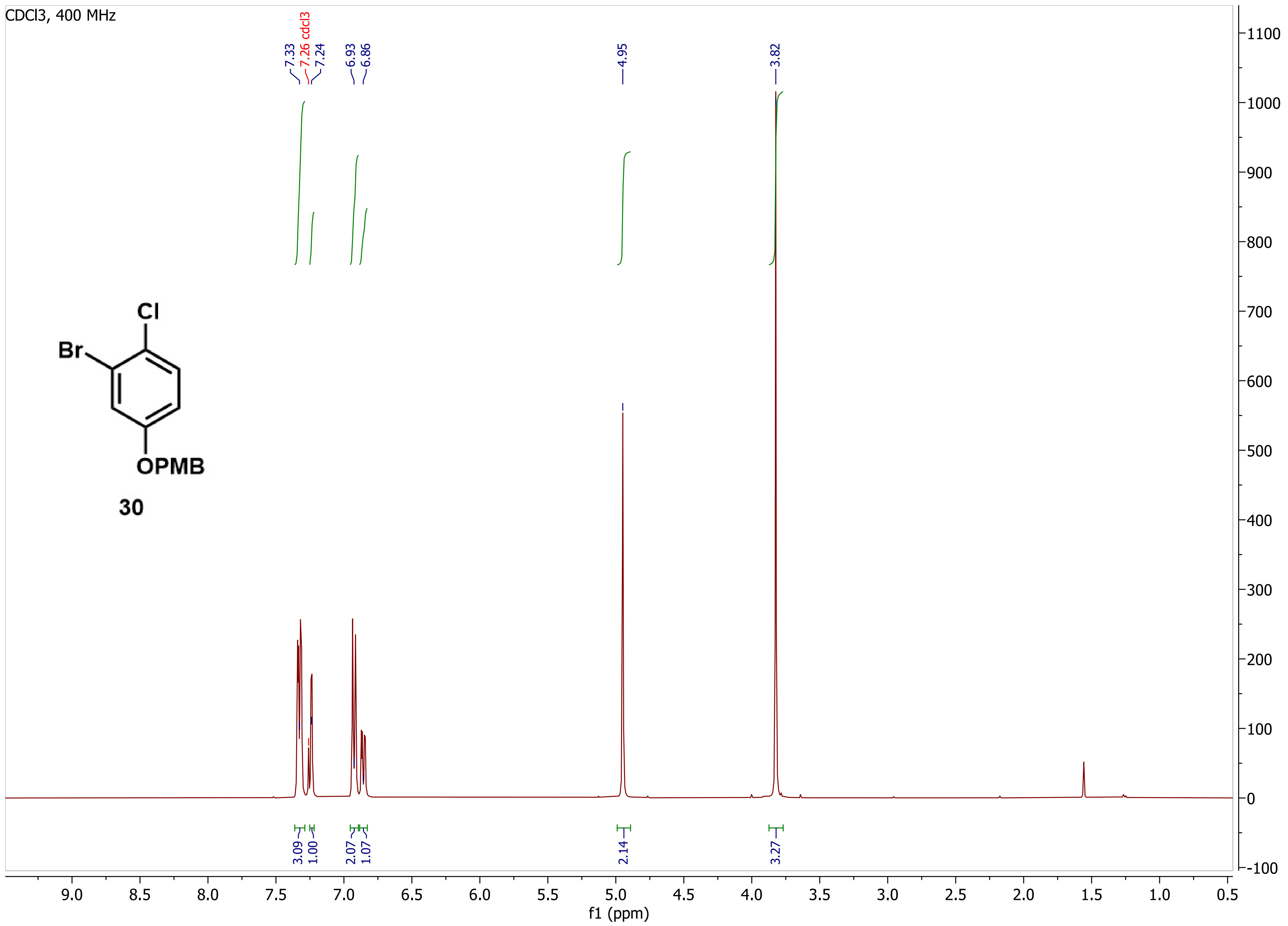

CDCl3, 400 MHz
7.33
7.26 cdcl3
7.24
6.93
6.86
4.95
3.82
Cl
Br
OPMB
30
3.09
1.00
2.07
1.07
2.14
3.27
f1 (ppm)

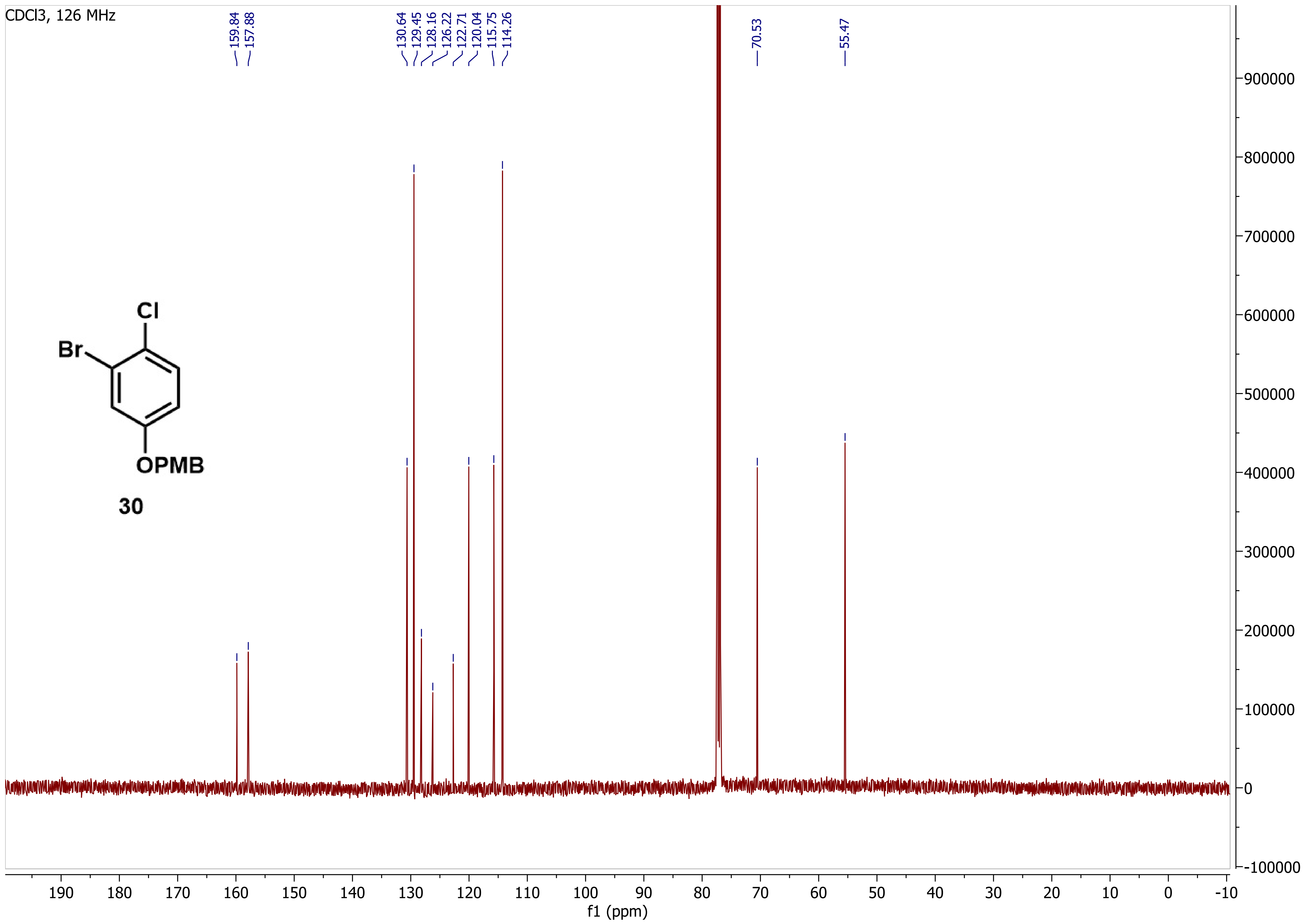
CDCl3, 126 MHz
159.84
157.88
130.64
129.45
128.16
126.22
122.71
120.04
115.75
114.26
70.53
55.47
Cl
Br
OPMB
30
f1 (ppm)

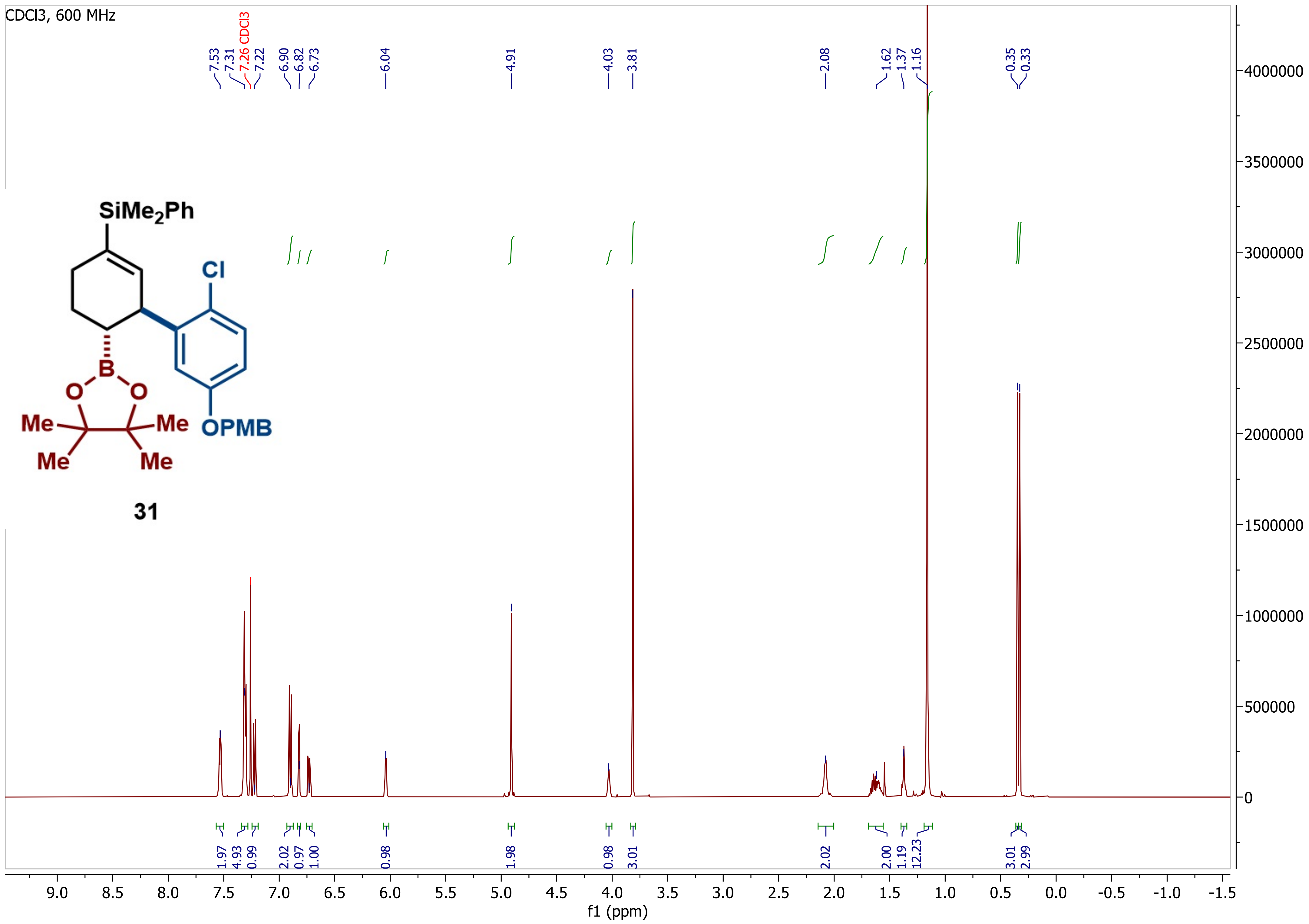

CDCl3, 600 MHz
7.53
7.31
7.26 CDCl3
7.22
6.90
6.82
6.73
6.04
4.91
4.03
3.81
2.08
1.62
1.37
1.16
0.35
0.33
1.97
4.93
0.99
2.02
0.97
1.00
0.98
1.98
0.98
3.01
2.02
2.00
1.19
12.23
3.01
2.99
SiMe2Ph
Cl
B
O
O
Me
Me
Me
Me
OPMB
31
f1 (ppm)

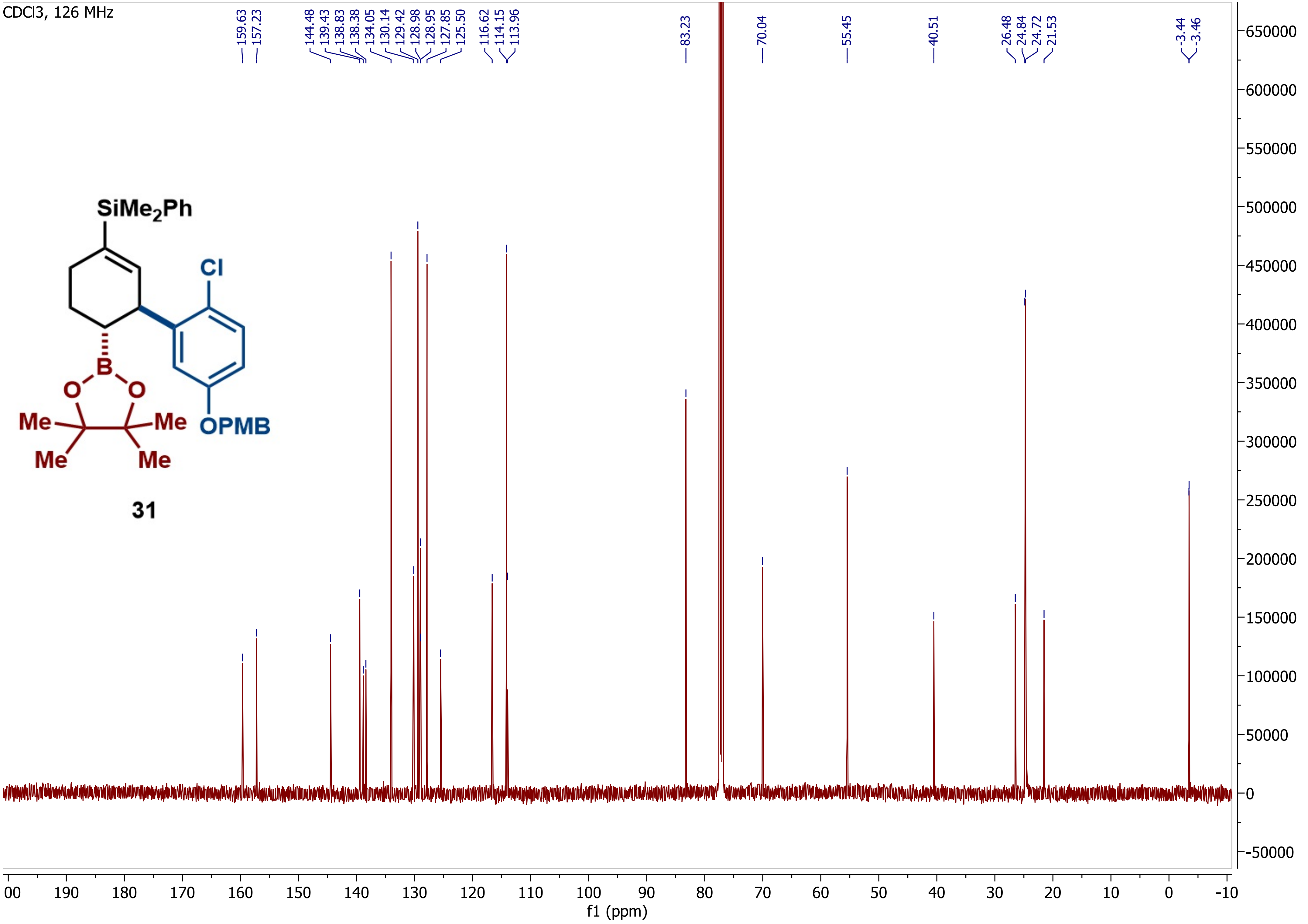
CDCl3, 126 MHz
159.63
157.23
144.48
139.43
138.83
138.38
134.05
130.14
129.42
128.98
128.95
127.85
125.50
116.62
114.15
113.96
83.23
70.04
55.45
40.51
26.48
24.84
24.72
21.53
-3.44
-3.46
f1 (ppm)
SiMe2Ph
Cl
B
O
O
Me
Me
Me
Me
OPMB

31

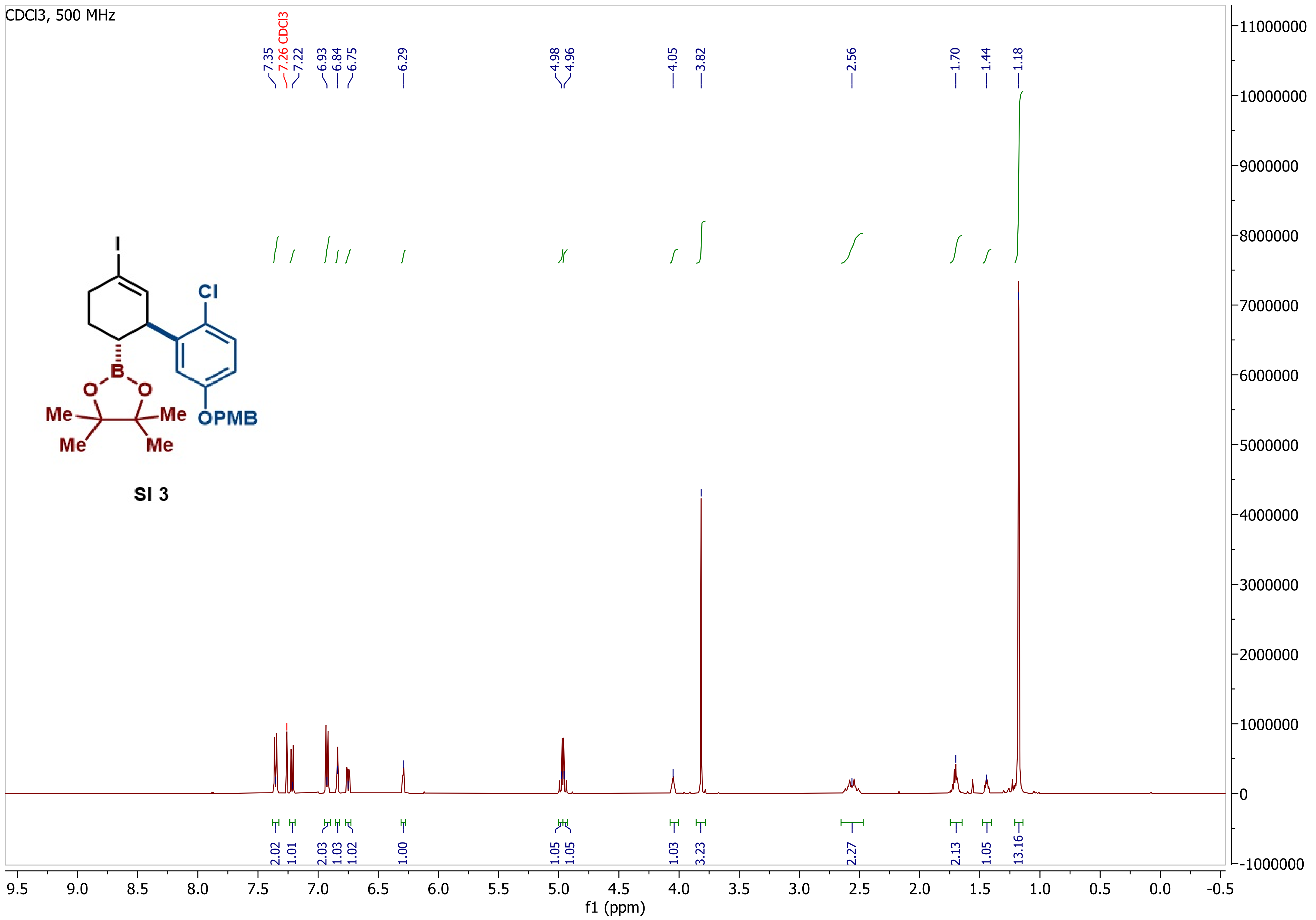
CDCl3, 500 MHz
7.35
7.26 CDCl3
7.22
6.93
6.84
6.75
6.29
4.98
4.96
4.05
3.82
2.56
1.70
1.44
1.18
I
Cl
B
O
O
Me
Me
Me
Me
OPMB
SI 3
2.02
1.01
2.03
1.03
1.02
1.00
1.05
1.05
1.03
3.23
2.27
2.13
1.05
13.16
f1 (ppm)

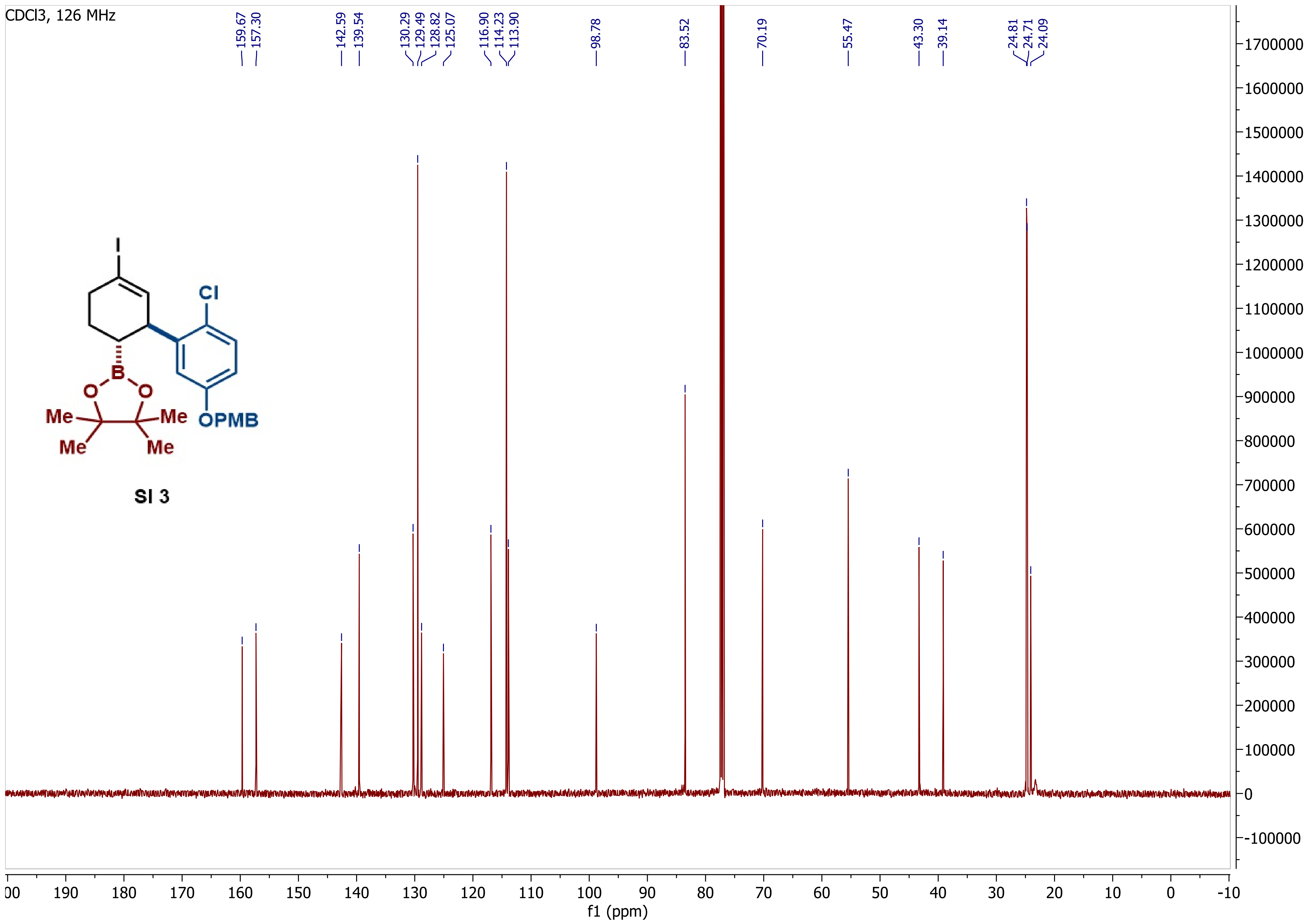
CDCl3, 126 MHz
159.67
157.30
142.59
139.54
130.29
129.49
128.82
125.07
116.90
114.23
113.90
98.78
83.52
70.19
55.47
43.30
39.14
24.81
24.71
24.09
I
Cl
B
O
O
Me
Me
Me
Me
OPMB
SI 3
f1 (ppm)

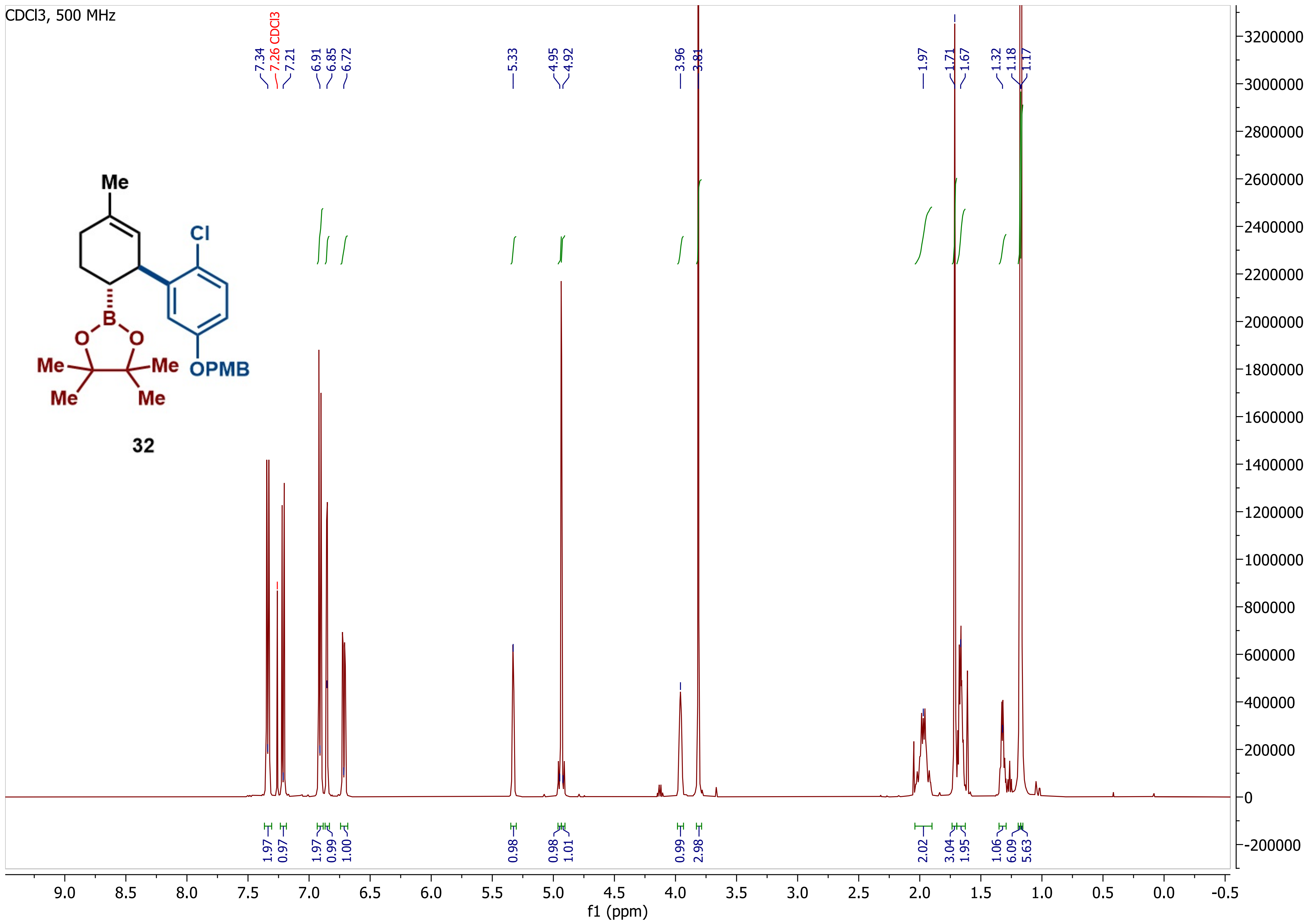

CDCl3, 500 MHz
7.34
7.26 CDCl3
7.21
6.91
6.85
6.72
5.33
4.95
4.92
3.96
3.81
1.97
1.71
1.67
1.32
1.18
1.17
Me
Cl
B
O
O
Me
Me
Me
Me
OPMB
32
1.97
0.97
1.97
0.99
1.00
0.98
0.98
1.01
0.99
2.98
2.02
3.04
1.95
1.06
6.09
5.63
f1 (ppm)

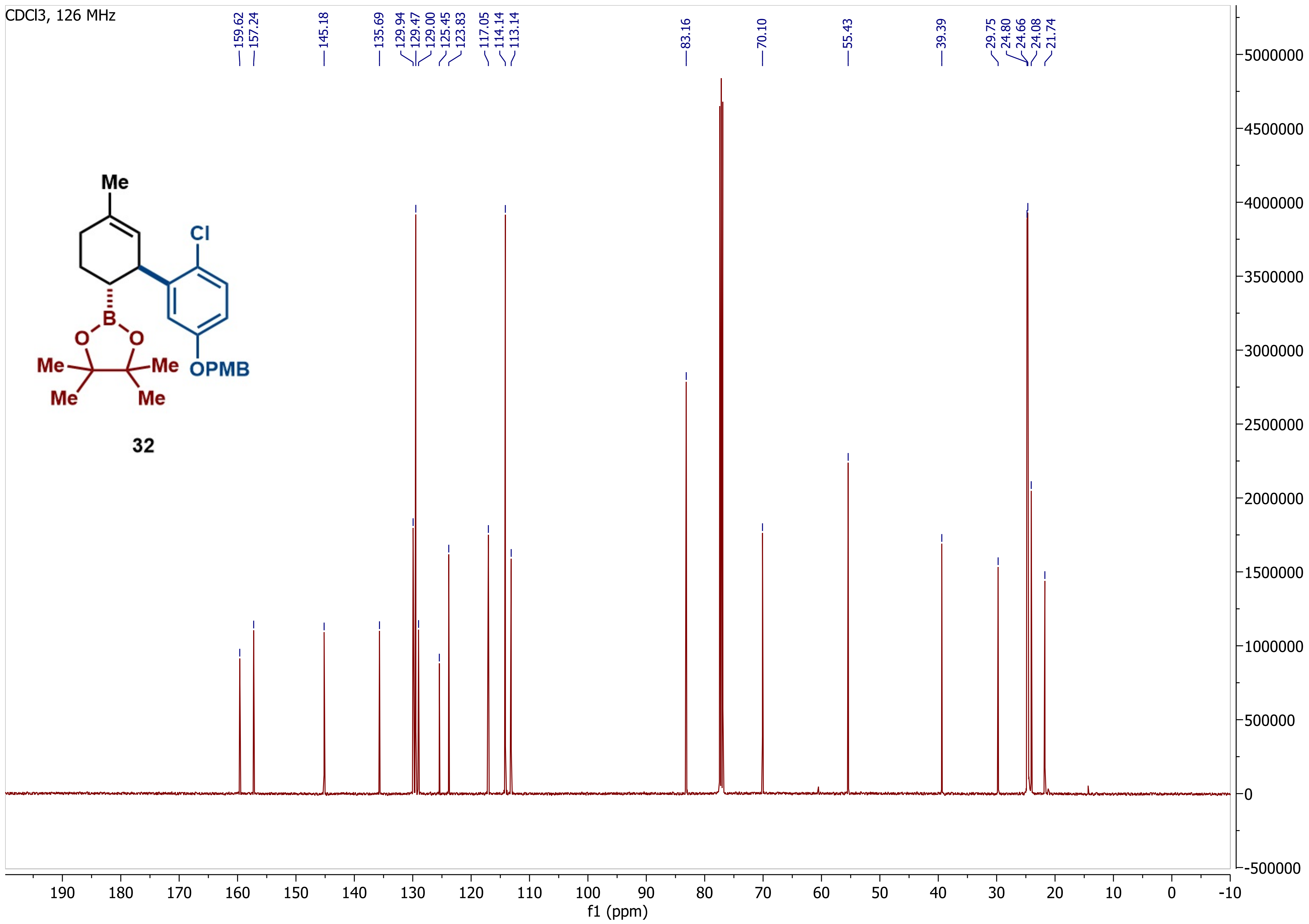
CDCl3, 126 MHz
159.62
157.24
145.18
135.69
129.94
129.47
129.00
125.45
123.83
117.05
114.14
113.14
83.16
70.10
55.43
39.39
29.75
24.80
24.66
24.08
21.74
Me
Cl
B
O
O
Me
Me
Me
Me
OPMB
32
f1 (ppm)

CDCl3, 400 MHz

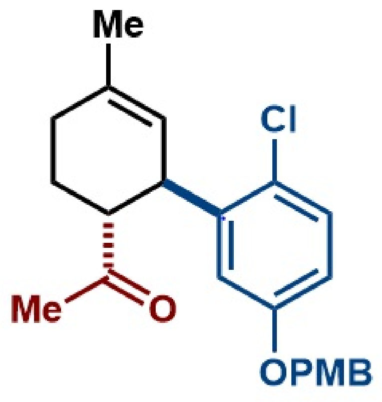

33

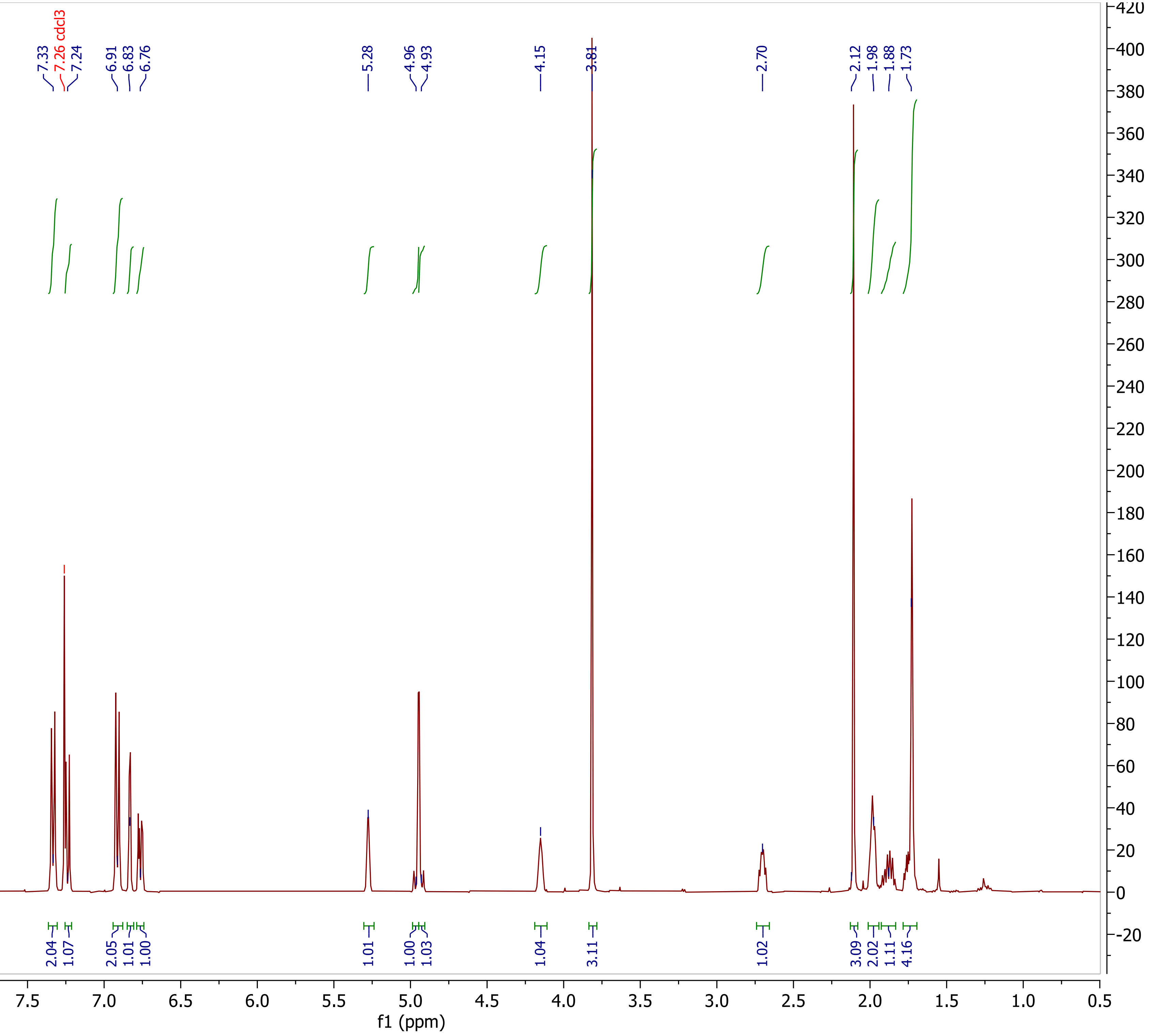

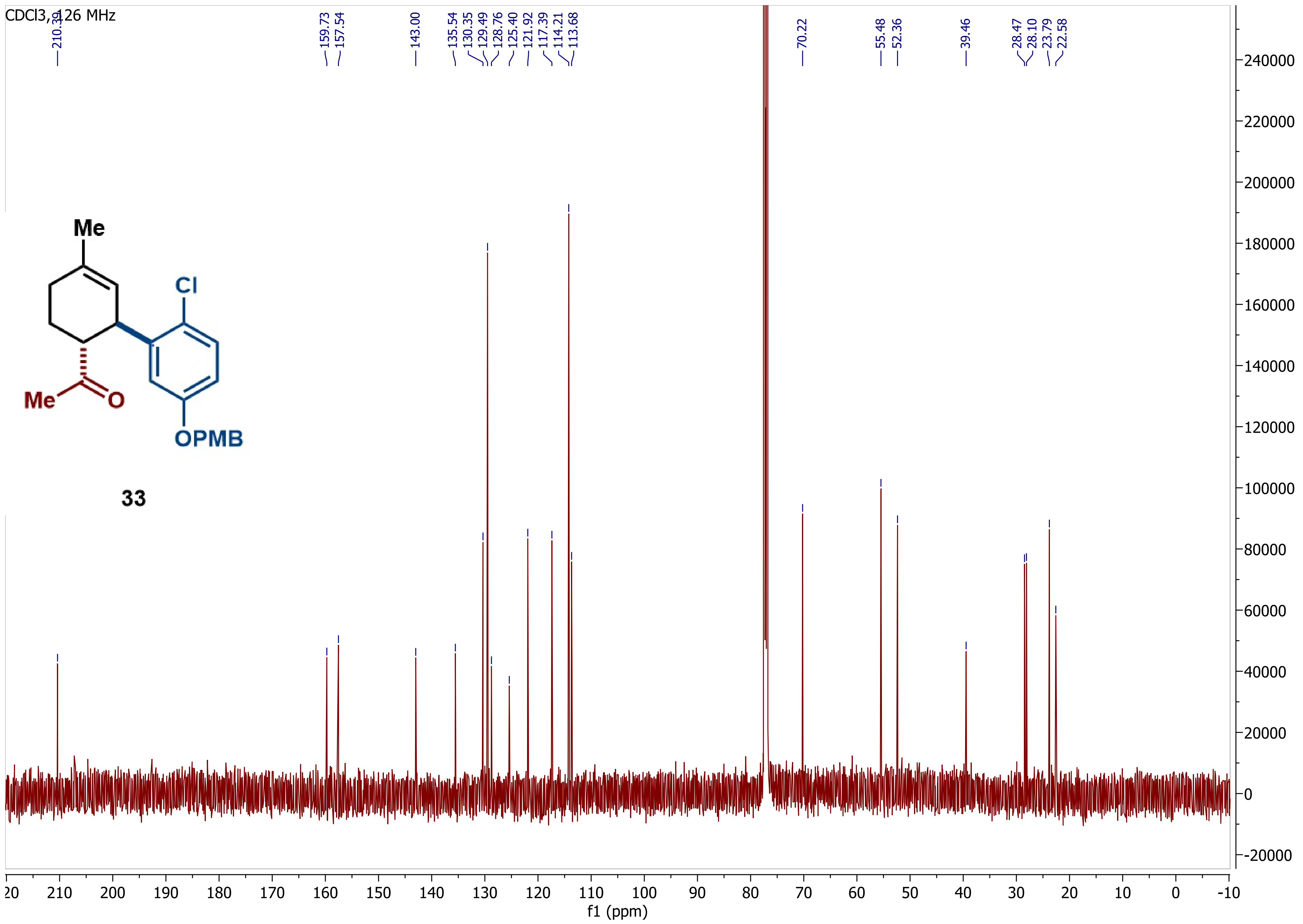

CDCl3, 126 MHz
210.39
159.73
157.54
143.00
135.54
130.35
129.49
128.76
125.40
121.92
117.39
114.21
113.68
70.22
55.48
52.36
39.46
28.47
28.10
23.79
22.58
Me
Cl
Me
O
OPMB
33
f1 (ppm)
